# The ancient mammalian KRAB zinc finger gene cluster on human chromosome 8q24.3 illustrates principles of C2H2 zinc finger evolution associated with unique expression profiles in human tissues

**DOI:** 10.1186/1471-2164-11-206

**Published:** 2010-03-26

**Authors:** Peter Lorenz, Sabine Dietmann, Thomas Wilhelm, Dirk Koczan, Sandra Autran, Sophie Gad, Gaiping Wen, Guohui Ding, Yixue Li, Marie-Françoise Rousseau-Merck, Hans-Juergen Thiesen

**Affiliations:** 1 Institute of Immunology, University of Rostock, Schillingallee 70, 18055 Rostock, Germany; 2 Institute of Bioinformatics, GSF - Research Institute for Environment and Health, Ingolstaedter Landstr. 1, 85764 Neuherberg, Germany; 3 Theoretical Systems Biology, Institute of Food Research, Norwich Research Park, Norwich NR4 7UH, UK; 4 Inserm U830, Institut Curie, 26 rue d'Ulm, 75248 Paris cedex 05, France; 5 Service de Génétique Oncologique, Institut Curie, 26 rue d'Ulm, 75248 Paris cedex 05 Paris, France; 6 Department of Genome Analysis, Leibniz Institute for Age Research-Fritz Lipmann Institute, Beutenbergstr 11, 07745 Jena, Germany; 7 Current address: Institut für Agrar- und Ernährungswissenschaften Martin-Luther-Universität Halle-Wittenberg, Von-Danckelmann-Platz 2, 06120 Halle (Saale), Germany; 8 Bioinformatics Center, Key Lab of Systems Biology, Shanghai Institutes for Biological Sciences, Chinese Academy of Sciences, Yueyang Road, Shanghai 200031, PR China

## Abstract

**Background:**

Expansion of multi-C2H2 domain zinc finger (ZNF) genes, including the Krüppel-associated box (KRAB) subfamily, paralleled the evolution of tetrapodes, particularly in mammalian lineages. Advances in their cataloging and characterization suggest that the functions of the KRAB-ZNF gene family contributed to mammalian speciation.

**Results:**

Here, we characterized the human 8q24.3 ZNF cluster on the genomic, the phylogenetic, the structural and the transcriptome level. Six (ZNF7, ZNF34, ZNF250, ZNF251, ZNF252, ZNF517) of the seven locus members contain exons encoding KRAB domains, one (ZNF16) does not. They form a paralog group in which the encoded KRAB and ZNF protein domains generally share more similarities with each other than with other members of the human ZNF superfamily. The closest relatives with respect to their DNA-binding domain were ZNF7 and ZNF251. The analysis of orthologs in therian mammalian species revealed strong conservation and purifying selection of the KRAB-A and zinc finger domains. These findings underscore structural/functional constraints during evolution. Gene losses in the murine lineage (ZNF16, ZNF34, ZNF252, ZNF517) and potential protein truncations in primates (ZNF252) illustrate ongoing speciation processes. Tissue expression profiling by quantitative real-time PCR showed similar but distinct patterns for all tested ZNF genes with the most prominent expression in fetal brain. Based on accompanying expression signatures in twenty-six other human tissues ZNF34 and ZNF250 revealed the closest expression profiles. Together, the 8q24.3 ZNF genes can be assigned to a cerebellum, a testis or a prostate/thyroid subgroup. These results are consistent with potential functions of the ZNF genes in morphogenesis and differentiation. Promoter regions of the seven 8q24.3 ZNF genes display common characteristics like missing TATA-box, CpG island-association and transcription factor binding site (TFBS) modules. Common TFBS modules partly explain the observed expression pattern similarities.

**Conclusions:**

The ZNF genes at human 8q24.3 form a relatively old mammalian paralog group conserved in eutherian mammals for at least 130 million years. The members persisted after initial duplications by undergoing subfunctionalizations in their expression patterns and target site recognition. KRAB-ZNF mediated repression of transcription might have shaped organogenesis in mammalian ontogeny.

## Background

Evolution of tetrapodes coincides with the expansion of Krüppel-type C2H2 zinc finger (ZNF) genes leading to the largest gene family involved in transcriptional gene regulation [1-7]. Members of the most prominent subfamily contain the Krüppel-associated box (KRAB) transcriptional repressor domain at their N-terminus [8-10]. The Krüppel-type zinc finger domain was originally identified in *Xenopus laevis *TFIIIA [11] and the Krüppel mutant of *Drosophila melanogaster *[12]. It is known as nucleic acid interaction domain but can also contribute to protein-protein interactions [13-16]. The classical zinc finger fold consists of an approximately 30-amino acid unit of two antiparallel β strands linked to an amphipathic α-helix with two cysteines and two histidines coordinating a zinc ion to stabilize the structure [17]. DNA-binding-specificity relies on amino acids within the α-helix reaching into the major groove of DNA [18]. According to the crystal structure of DNA bound EGR1/Zif268, the residues most crucial for DNA binding are localized at positions -1, 3 and 6 with respect to the start of the α-helix. Position 2 is also involved in DNA binding, but makes contact to the complementary strand [13]. Zinc finger domains usually occur in arrays of multiple C2H2 zinc finger modules comprising from only a few up to more than thirty units [19]. The individual units are separated by a conserved sequence (consensus TGEKP) called HC link.

The KRAB domain was originally described as heptad repeat of leucines in KOX1/ZNF10 [8] and shown to be evolutionarily conserved [9], for review see [20]. The KRAB domain of KOX1 consists of a KRAB-A and a KRAB-B subdomain of which the KRAB-A subdomain mediates transcriptional repression [21-23] and the KRAB-B part enhances the repression in conjunction with KRAB-A [24]. Later on, different KRAB-B subdomains with different properties have been discovered [19,25,4]. The KRAB domain, early postulated as a protein-protein interaction domain [8], has been shown to interact with the RBCC domain of TRIM28 (tripartite motif-containing 28, also known as KAP-1, KRIP-1, TIF1β; reviewed in [20]). This protein is considered essential for KRAB-mediated transcriptional repression and recruits various chromatin-modifying protein complexes, thus leading to a repressive chromatin state [26-28]. It is most likely that all KRAB C2H2 zinc finger proteins mediate transcriptional repression in a sequence-specific manner. It is currently unresolved how many target genes are regulated by an individual KRAB zinc finger protein. In case of KOX1/ZNF10, initial knockdown experiments with KOX1 specific antisense oligonucleotides combined with transcriptome analysis argued that a KRAB-ZNF protein might modulate the expression of 50 to 80 target genes in a direct or indirect manner [29].

Interestingly, functions of most KRAB-ZNF genes remain elusive so far. Recent reports showed evidence that KRAB-ZNF genes and TRIM28 are involved in differentiation and development [30-35]. Furthermore, genetic studies linked members of the KRAB-ZNF family to human disease [36-39].

The PFAM protein family database states that KRAB-ZNF genes occur in all tetrapodes from amphibians to birds and in all mammalian species (see http://pfam.sanger.ac.uk/family?entry=krab&type=Family), whereas fish species like *Fugu *do not appear to encode any KRAB-ZNF genes [3,6]. Noteworthy, less than 20 KRAB-ZNF genes are found in the genome of amphibians (*Xenopus laevis/tropicalis*) compared with the human genome for which comprehensive studies list 300-400 KRAB-ZNF protein-coding genes [4,40,6]. Thus, KRAB-zinc finger genes presumably coevolved with or occurred shortly after the appearance of tetrapodes and underwent a huge expansion during mammalian evolution. It was hypothesized that KRAB-like sequences in the histone methyltransferase Meisetz date the origin of this domain back to the last common ancestor of chordates and echinoderms [41]. However, this KRAB-like domain is closely related to the KRAB-like domain of the SSX proteins that does not interact with TRIM28 and consequently does not initiate transcriptional repression [42].

Initial mapping data on KOX zinc finger genes already indicated that most ZNF genes are clustered in the human genome [43]. This was confirmed by detection of 23 chromosomal KOX gene ZNF loci [44]. Numerous ZNF gene clusters have been defined over the years [45,3,49] and catalogued to a total of about 60-90 genomic loci [4,5,40], depending on definition. Diversification of ZNF genes during evolution is reflected by duplication and deletion of zinc finger domains thereby modifying recognition specificities for RNA/DNA binding [50,2,51]. Individual degenerate non-functional zinc finger domains do occur within a sequence as well as after truncation by introduction of an in-frame stop codon (reviewed for KRAB-ZNFs in [52]). Lineage-specific expansions and losses within ZNF clusters contribute to evolutionary adaptation [2,3,49,51,5,6].

In this manuscript we focus on the human ZNF cluster at 8q24.3 that had not been investigated in detail. It was chosen because of the presence of several KOX ZNF genes (ZNF7/KOX4, ZNF16/KOX9 and ZNF34/KOX32) for which we have a longstanding interest [44]. We sequenced the locus as part of the german HUGO initiative on chromosome 8 and identified seven ZNF genes. These genes form a paralog group well separated from other ZNF subfamilies. We show here that subfunctionalization of the individual members occurred through the modification of structural properties (KRAB protein interaction domain; ZNF DNA binding domain) as well as through fine-tuned tissue expression patterns. Phylogenetic analysis in several mammalian species indicated strong conservation and purifying selection of the KRAB and ZNF domains of these genes on one hand. On the other hand, gene loss and potential protein truncations in some species also denoted ongoing evolution. RNA expression was found to prevail in tissues with high degree of differentiation, most notably in fetal brain. Our investigation of the human 8q24.3 ZNF locus illustrates principles of ZNF gene evolution. The ZNF gene family provides a rich repertoire of transcription factors with distinct RNA/DNA binding specificities. In particular ZNF genes encoding the KRAB transcriptional repression domain represent a great regulatory potential to tune expression of numerous target genes thus contributing to the biodiversity seen in tetrapod evolution.

## Results

### Organization of the ZNF cluster at chromosome 8q24.3

**Figure 1:**
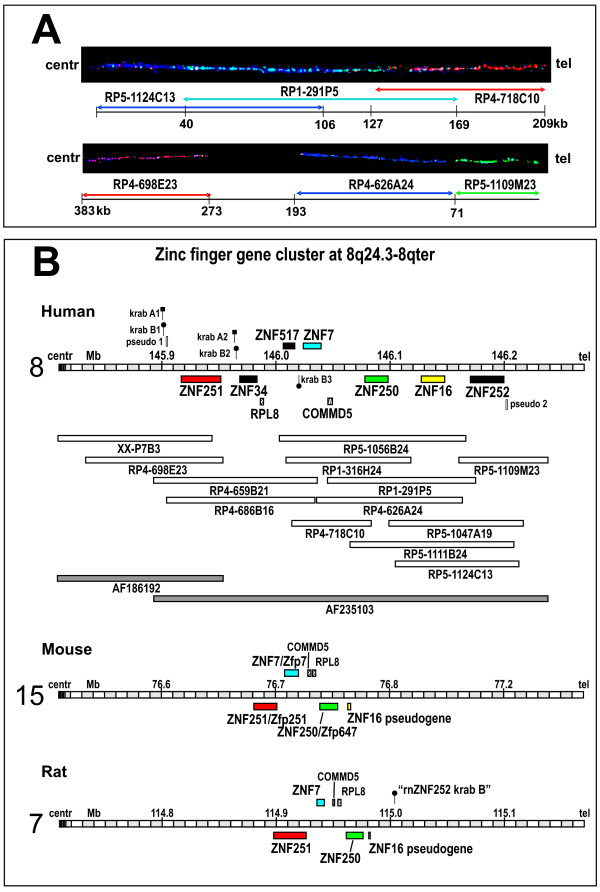
**Genomic organization of the human ZNF cluster at chromosomal region 8q24.3 and its syntenic regions in mouse and rat**. A. FISH of ZNF gene PAC clones on combed DNA. Orientation is given by indicating the centromeric (centr) and telomeric (tel) directions. Numbers on the depicted DNA indicate sizes within the piece of combed DNA. B. Map of the sequenced individual PAC clones at the 8q24.3-8qter locus (open bars) and their alignment/assembly within the whole contig (genomic assemblies with respective GenBank accessions shown as grey bars). Range, size and chromosomal position of the whole contig is reflected in the depicted chromosomal region on top. The ZNF gene models (see Table 1, Figure 2 and text) are indicated as colored boxes encompassing the sequence stretch from first to last exon. Genomic regions syntenic to human 8q24.3 in mouse and rat are depicted at the bottom. Same coloring designates orthologs. The three human genes depicted in black lack orthologs in mouse and rat (exception: presumable KRAB-B remnant of ZNF252 ortholog in rat, see text). Isolated, often degenerate sequences for KRAB-A ("pin" with square head), KRAB-B ("pin" with round head) and C2H2 zinc fingers (open rectangle with label "pseudo") are displayed at their respective position. Two other well characterized genes (RPL8, COMMD5) in the region are shown by striped boxes. The coding strand of each element is indicated by positioning above or below the depicted chromosomal region. Based on genome assemblies UCSC hg18 (human), mm9 (mouse) and rn4 (rat).

The ZNF cluster was initially determined by chromosome mapping of sixteen different ZNF PAC clones to genomic locus 8q24.3 of which thirteen were specific for this locus [44] whereas three clones showed signals of comparable intensities on other chromosomes as well. Fluorescence in situ hybridization (FISH) on interphasic nuclei and molecular combing techniques allowed to approximate the size and the organization of the 8q24.3 ZNF cluster. A first approach on interphasic nuclei was employed to select the closer co-localizing PAC clones from the more distant mapping ones. Two Pac clones, RP5-1109M23 and RP4-698E23 were clearly delimiting the borders of the cluster. FISH on combed DNA, performed with two series of three PAC clones, confirmed the close proximity of the ZNF PAC clones all along the cluster and allowed to estimate an approximate size for each probe taking into account a 10% variability for the resolution. Overall alignment lengths of 209 kb and 383 kb were obtained for RP5-1124C13/RP1-291P5/RP4-718C10 and RP5-1109M23/RP4-626A24/RP4-698E23, respectively (Figure 1A).

The 16 different PAC clones were aligned to genomic sequence information confirming that 13 ZNF PAC clones belong to one contig. The sequence of the contig is formed by the PAC clones XX-P7B3, RP4-659B21, RP5-1056B24 and RP5-1109M23 over a total size of 430 kb (Figure 1B). The other nine ZNF PACs cover the same region. Sequence data analysis confirmed the exclusion of the three ambiguously mapping PAC clones from the 8q24.3 ZNF contig. The respective lengths of the thirteen overlapping PACs have been established and are in agreement with the data obtained with the combed DNA. The whole 8q24.3 ZNF contig has been filed under GenBank accession number AF235103. Individual sequence information of the PAC clones can also be accessed at the Genome Analysis center in Jena http://genome.imb-jena.de under human chromosome 8q24.3 locus. The information obtained from sequencing of the PAC clones led to the identification of seven C2H2 Krüppel-type zinc finger genes in the 8q24.3 contig within a distance of nearly 300 kb (Figure 1B). With respect to orientation, two genes, ZNF517 and ZNF7, show a transcriptional direction towards the telomere whereas the five others, namely ZNF251, ZNF34, ZNF250, ZNF16 and ZNF252 are encoded on the complementary strand and are transcribed towards the centromere direction.

### Gene models and protein domain organization of the ZNF genes on contig 8q24.3

**Figure 2:**
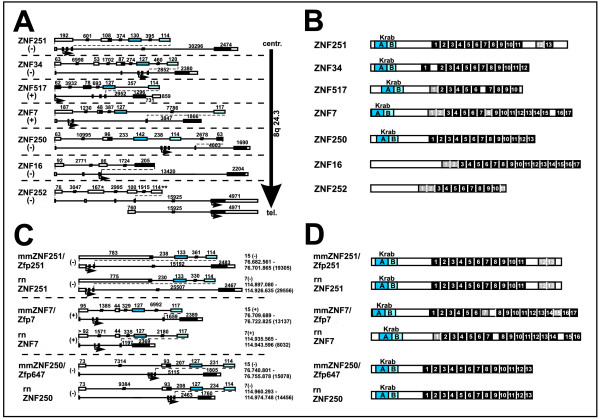
**Gene models (A, C) and protein domain organization (B, D) of the seven human ZNF genes at 8q24.3 (A, B) and their murine orthologs (C, D)**. In the gene models (A, C) exons are shown as boxes (white parts are untranslated, filled parts are translated), introns as solid lines. KRAB-A, KRAB-B and non-KRAB domain encoding sequences are shaded in dark blue, light blue and black, respectively. Numbers above exons/introns indicate their size (bp). Genes are drawn to scale with smaller exons additionally magnified for more detail. Introns interrupted by two perpendicular bars are not fully drawn out. Names of genes and proteins are given on the left of each model. Mouse and rat orthologs are indicated by prefixes mm (*Mus musculus*) or rn (*Rattus norvegicus*). * in the ZNF252 5-exon model indicates out of frame KRAB-A coding sequence, ** designates a degenerate KRAB-B box disrupted by a stop codon. The genes' direction of transcription is given by "+" or "-" (towards telomere or centromere, respectively). Genomic order of the human genes is indicated by the arrow in A. Exact genomic locations are displayed for mouse and rat genes (based on UCSC assemblies mm9 and rn4). Small arrows denote positions of presumable translational starts. Drawn to scale protein architectures (B, D) depict KRAB-A (dark blue), KRAB-B (light blue) and C2H2 ZNF domains (numbered boxes; black boxes represent complete, grey boxes degenerate or non-canonical fingers). Only fingers with at least two of the four conserved C2H2 residues were considered.

BLAST analysis of the ZNF sequences against human transcribed sequences and ESTs as well as evaluation of the Acembly and ENSEMBL databases resulted in the definition of transcript sequences for the seven ZNF genes. The focus was the definition of the longest possible open reading frame, not taking into account the possibility of different splice forms. All the gene models together encompass an approximately 282 kB piece of the qter region. The characteristics of the ZNF transcripts are given in Table 1. Out of the seven longest open reading frames for each gene five transcripts encode potentially functional Krüppel-associated box (KRAB) domains in the setting KRAB-A and KRAB-B. The comparison of the defined transcripts with the human genome (see Methods section) resulted in the description of the individual genomic organization for each ZNF gene. The individual cDNA sequences of the gene models can be found in the supplementary material (Additional file 1). A common theme among the seven ZNF genes are 5' untranslated exons, the coding of the C2H2 zinc finger modules all in one exon and, in the case of the five KRAB-containing ZNF genes, the typically separated KRAB-A and KRAB-B exons. Concerning ZNF252, we have been able to confirm both transcripts (see Figure 2A), the one with four 5' untranslated upstream exons as well as the one with only one 5' untranslated exon, by RT-PCR with specific primers that resulted in exon spanning products (data not shown). Both transcripts encode the same amino acid sequences from within the last exon. Interestingly, the amino terminus of the largest open reading frame contains a KRAB-B domain disrupted by a stop codon and exon 2 of the 5-exon form encodes a KRAB-A peptide. In between resides an additional exon, unusual for KRAB-ZNF genes. However, there is currently no evidence for a transcript that links KRAB-A and zinc finger domains in the same open reading frame. Noteworthy, the ZNF252 3' untranslated region contains a row of eleven complete as well as degenerate zinc fingers motifs after the stop codon of the longest open reading frame (see Additional file 2 for sequence). The stop codon is confirmed in the human genome sequence (e.g. in our PAC clone RP5-1109M23 GenBank AC087815), in cDNA and EST sequences (e.g. AK128723, BX505655, CD251662).

**Table 1:** ZNF genes and transcripts^a ^in the human 8q24.3 ZNF cluster on contig AF235103 (chr 8:145.893.231 -- 146.238.749)

Gene name (HUGO)	**Location**^**b**^	Strand	**Nucleotide sequence**^**c**^	**RefSeq**^**d**^	**Gene ID**^**e**^	ENSEMBL transcript ID.^f^	No of exons	cDNA (bp)	Amino acids (aa)	Remarks
**ZNF251**	145 917 103 -- 145 951 786 (34 684 bp)	**-**	AK000435 AK091638 XM_291262 BC006258	NM_138367	90987	ENST00000 292562	5	3018	671	KRAB-A KRAB-B

**ZNF34**	145 968 415 -- 145 983 530 (15 116 bp)	**-**	AK096508 AL833814 BC028136 BC004480	NM_030580	80778	ENST00000 343459	6	2830	539	KRAB-A KRAB-B alias Kox32

**ZNF517**	145 995 065 -- 146 006 265 (11 201 bp)	**+**	AK097278 AK096527 AK131440 XM_291261 AX721108	NM_213605	340385	ENST00000 359971	6	>2536	>527	KRAB-A KRAB-B

**ZNF7**	146 023 815 -- 146 039 409 (15 595 bp)	**+**	M29580 AK096025	NM_003416	7553	ENST00000 325241	5	2345	686	KRAB-A KRAB-B alias: Kox4

**ZNF250**	146 077 336 -- 146 097 650 (20 315 bp)	**-**	AK054888 BC017091 AK095705 X16282	NM_021061 NM_001103159	58500	ENST00000 292579	6	2168	560	KRAB-A KRAB-B alias: ZNF647

**ZNF 16**	146 126 577 -- 146 147 078 (20 502 bp)	**-**	BC010996 AK096815 AF244088 X52340 AL834451	NM_006958 NM_001029976	7564	ENST00000 276816	4	>2501	>715	no KRAB alias: Kox9

**ZNF252**	146 169 773 -- 146 191 428 (21 656 bp) 146 169 773 -- 146 199 082 (29 310 bp)	-	BC016287 BC019922 AK128723 BC065734	NR_023392	286101	ENST00000 355436	2 5	5731 5428	461 461	KRAB-A not in ZNF ORF; KRAB-B with stop codon after stop in frame more zinc fingers

^a^ Description of the largest transcript of the respective gene model

^b^ Chromosomal position according to human genome build hg18 (UCSC browser at http://genome.ucsc.edu; derived from NCBI genome assembly 36.1)

^c^ Selected NCBI GenBank and

^d^ Reference sequence collection accession numbers of representative cDNA/EST sequences

^e^ Entrez GeneID

^f^ Transcript proposed at http://www.ensembl.org

Searches for further KRAB-A, KRAB-B and C2H2 sequences on contig AF235103, besides the ones in the above described genes, revealed (Figure 1B) a pseudogene with potentially a complete set of KRAB-A, KRAB-B and zinc finger sequences at the centromeric end of the cluster (krab A1, krab B1, pseudo 1), truncated KRAB-A and KRAB-B sequences near each other (krab A2, krab B2), an isolated KRAB-B sequence (krab B3) and zinc finger sequences at the telomeric end of the cluster (pseudo 2). With the exception of krabA1 and krabB1, all these sequences are characterized by numerous stop codons and degenerate domain structure (nucleic acid sequences included in Additional file 1).

The domain organization of the proteins predicted from the gene models is presented in Figure 2B (see Additional file 2 for amino acid sequences). The KRAB-A and -B boxes of the five KRAB zinc finger proteins are located near the amino terminus followed by non-conserved linker sequences and numerous C2H2 zinc finger modules. Most of them are in a consecutive order joined by the conserved HC link sequences and equipped with the two cysteines and two histidines in the correct spacing. In some circumstances the consecutive zinc finger array is broken by gaps, e.g in the case of ZNF251, in such a way that the twelfth finger is isolated. In addition, some motifs are more degenerated, i.e. they lack one of the hallmark cysteine or histidine amino acid residues at the right position or are not complete (see e.g. ZNF7). Comparison of the KRAB-B domains of the 8q24.3 ZNF proteins to several other KRAB-B domains defining the KRAB AB or Ab subfamilies [19] clearly indicated that they belong to the KRAB AB subfamily (data not shown). ZNF252 and the KRAB-less ZNF16 are characterized by considerable stretches of peptide sequence without known conserved domain at the amino terminus followed by consecutive zinc finger arrays of seventeen or eleven zinc fingers, respectively.

### Phylogeny of the ZNF genes in the human 8q24.3 cluster in mammals

As a first step to analyze evolutionary conservation of the human 8q24.3 locus we searched for orthologs in other mammalian species by reciprocal BLAST searches and inspection of the syntenic regions. The region syntenic to human 8q24.3 (AF235103) in chimpanzee (*Pan troglodytes*, PanTro2) contains all seven ZNF genes defined in the human and spans about 355 kB on chromosome 8 (bases 149.153.255-149.508.639; still numerous gaps). The gene structures of the chimpanzee ZNF orthologs are identical to the human ones. As expected sequence similarities between the human and chimpanzee orthologs are very high, ranging from 96 to 99% identity on the amino acid level (data not shown).

The murine syntenic regions of the human 8q24.3 ZNF region are on mouse chromosome 15 between approximately 76.682 and 76.766 Mb and on rat chromosome 7 between approximately 114.897 and 114.983 Mb. Using various search tools like BLAST, BLAT and the ENSEMBL database (as described in Material and Methods) and the human sequences as input, we were able to define three likely functional orthologs for the human ZNF genes from 8q24.3 in mouse and rat (Figures 1B, 2C). The mouse genes were well supported by cDNA and EST sequences. With the help of the mouse sequences we defined the corresponding rat orthologs from genomic sequences and adjusted the predicted gene models in GenBank for further phylogenetic examinations (see below). The rat gene models have the disadvantage not to be built on cDNA sequences due to the rat cDNA/EST databases being far less comprehensive in comparison to mouse or human. The nucleotide and peptide sequences we introduced in the analysis are again given in Additional files 1 and 2 and cornerstones of mouse/rat 8q24.3 ZNF orthologs are summarized in Additional file 3.

The three mouse and rat ZNF genes in the syntenic region of human 8q24.3 are all KRAB-A and -B box-containing genes. Like in human, KRAB-A and KRAB-B as well as the C2H2 coding genomic sequences are organized in separate exons. Gene organization and protein structure (Figure 2B, D) clearly reflect the closely related mouse/rat ortholog pairs and also correspond to the human ortholog. In addition to the three functional ZNF genes we found sequences homologous to human ZNF16 in the mouse and rat regions syntenic to human 8q24.3 by BLAT searches and HMMER search of C2H2 domains: There are continuous stretches on the reverse strand of the respective chromosome that are homologous to sequences of the zinc finger exon of ZNF16. If translated, the mouse as well as the rat genomic pieces would result in "broken" protein sequences of canonical mixed with degenerate C2H2 zinc fingers, disrupted by stop codons and jumping between reading frames. These findings support the notion, that the ZNF16 relatives in mouse and rat are pseudogenes without functional polypeptides. HMMER searches did not unravel any other KRAB box or C2H2 zinc finger fragments in these mouse and rat genomic regions syntenic to human 8q24.3. The only exception was an isolated sequence at the 3'-end of the ZNF locus in the rat genome that would potentially encode KRAB-B-like amino acid sequences. Since the best hit was the dog ZNF252 KRAB-B box using BLASTp against mammalian sequences, this might be the rat remnant of ZNF252 (designation "rnZNF252_krab_B"; see Figure 1B; sequences in Additional files 1, 2). Interestingly, it resides on the opposite strand with respect to the ZNF16 pseudogene. Because of its shortness the sequence was not included in further analyses.

Comparison of locus organization in mouse/rat and human shows that the mouse/rat region encompassed by the conserved non-ZNF genes RPL8 and COMMD5 is inverted in human such that the two genes reside on the opposite strand with ZNF7 and the human specific ZNF517 in between (Figure 1B).

The phylogenetic analysis was extended to all orthologs of the human 8q24.3 ZNF genes detected in respective genome assemblies of other mammalian species, namely, rhesus monkey, dog, cow and opossum (for nucleotide and amino acid sequences see Additional files 1 and 2; genome assembly references given in Methods). With respect to ZNF16 orthologs, rhesus monkey, dog as well as cow appear to have, like the primates, fully functional genes in contrast to mouse and rat (see above). Since ZNF34, ZNF517 and ZNF252 orthologs are likely to exist in other mammals (e.g. dog and cow for ZNF34, dog for ZNF517, dog and opossum for ZNF252), it seems that these genes were lost in the murine genomes.

### ZNF252 in the evolution of eutheria

**Figure 3:**
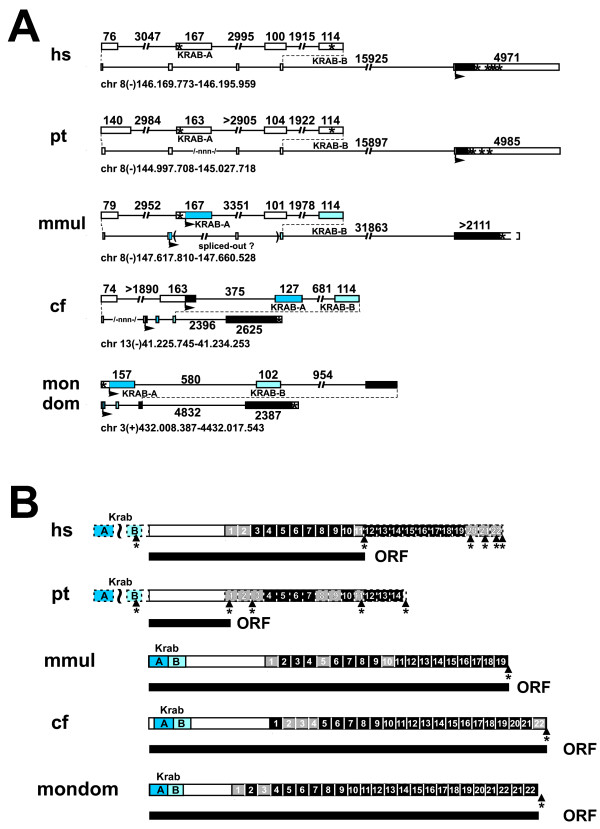
**Evolutionary changes in the ZNF252 ortholog group**. Gene (A) and protein domain (B) organization depicted for the mammalian ZNF252 ortholog group (see Additional files 1 and 2 for sequences). For legend see Figure 2. Gaps in the genomic sequence are indicated by --nnn-. An asterisk indicates a stop codon. Dashed lines in (B) illustrate potential coding sequences that are probably untranslated because of stop codons. The tilde denotes a shift in the open reading frame. The presumably longest predicted ORF are shown as black bars under the protein domain depiction. Species abbreviations: hs, *Homo sapiens*; pt, *Pan troglodytes*; mmul, *Macaca mulatta*; cf, *Canis familiaris*; mondom, *Monodelphis domestica*.

The peculiar open reading frame with broken KRAB domain and premature stop codon of human ZNF252 inspired us to carry out a more detailed comparison of the mammalian ZNF252 orthologs (Figure 3). The chimpanzee has the same 5' exon structure as the human ZNF252, i.e. out-of-frame KRAB-A and B exons with an additional exon inserted in between as well as a stop codon. However, the transcript apparently does not even encode any complete C2H2 zinc finger since a stop codon disrupts already the first would-be C2H2 domain between the C2 and H2 parts. Additional stops can be found just before sequences that would encode zinc fingers 9, 10 and 11. Thus, the chimpanzee ZNF252 protein, if expressed at all, is devoid of KRAB effector and ZNF DNA binding domains.

By using the tool RepeatMasker (A.F.A. Smit, R. Hubley & P. Green at http://repeatmasker.org) we found sequences homologous to Long and Short Interspersed Elements (LINE/SINE repeats) in the KRAB-A and the following exon and the interjacent intron of the human and chimpanzee ZNF252 transcripts (data not shown). This raises the possibility, that insertion of repeat sequences was responsible for the frame shift between the KRAB-A and the more downstream protein-encoding sequences. In contrast, the ZNF252 orthologs in rhesus monkey, dog (cDNA experimentally defined; GenBank: AJ388557) and opossum clearly harbor a fully developed KRAB-AB domain in the same open reading frame as the zinc fingers. In addition, the zinc fingers of ZNF252 extend to a number of 19-22 without disruption by any stop codon. The unusual additional exon between the KRAB-A and -B exons appears to be primate-specific. In conclusion, ZNF252 is an example for ongoing evolution in the mammalian lineage from fully functional KRAB zinc finger protein (e.g. dog) to residual protein fragment without KRAB and zinc fingers (chimpanzee) with recent considerable changes occuring after the divergence from the common ancestor of rhesus monkey and human/chimpanzee.

### Phylogenetic relationship of the human 8q24.3 cluster ZNF genes

In order to characterize the evolutionary relationships, we constructed phylogenetic trees after alignment of the seven human 8q24.3 ZNF genes along with their mouse and rat predicted orthologs. The analysis also contained the *Xenopus *KRAB-ZNF gene Xfin as outlier and human KRAB zinc finger genes from different genomic locations. The latter included seven ZNFs from locus 19q13.2, recently analyzed in detail [3], as reference group for comparison and in order to assess the stability of resulting clades. Alignments were performed with the cDNA sequences, the total amino acid sequences, the overall zinc finger DNA binding domains and the KRAB effector domains of the proteins. Phylogenetic relationships were then predicted by tree construction methods (see Methods).

**Figure 4:**
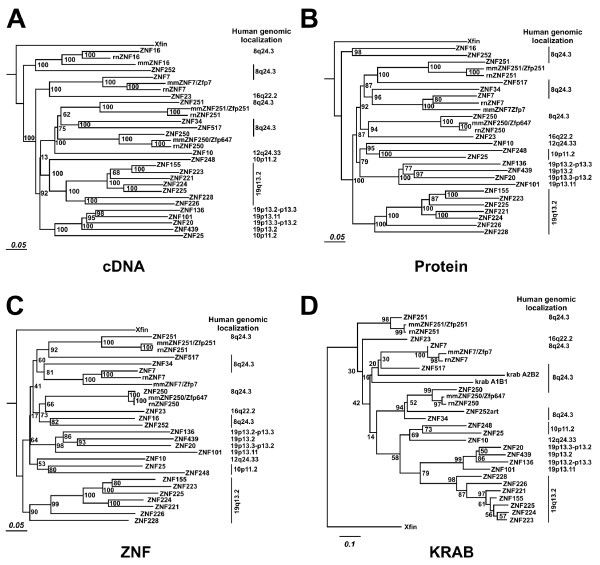
**Phylogenetic relationships between the human ZNF genes from 8q24.3, their murine orthologs and other human ZNF genes**. The analysis relied on alignments of full nucleotide cDNA sequences (A), of whole polypeptide sequences (B), of the array of all C2H2 zinc finger domains of each protein and of the KRAB domains using the neighbor-joining method. The analysis also included *Xenopus *Xfin as a distant outlier and as reference group seven KRAB-ZNFs from human 19q13.2 and eight KRAB-ZNFs from other genomic locations. Numbers indicate bootstrap values in percent based on 1000 replicates. To the right of the different clades the genomic localizations of the human genes are given. Note that the two KRAB domains of presumable pseudogenes (krab A1B1, krab A2B2) within 8q24.3 as well as an artificially combined human ZNF252 KRAB domain (labeled "art") have been added; see text for more details. Since the ZNF16 ortholog cDNAs from mouse and rat do most likely not give rise to a functional protein, protein sequences were not included in the analyses. The full nucleotide and protein sequences are given in Additional files 1 and 2, respectively.

The analysis of the 8q24.3 members revealed that, in general, the genes disperse into well separated clades/subclades with long branch lengths and often with many nodes in between (Figure 4), indicating relatively large phylogenetic distances and thus divergence times. This was the case for trees based on all four different alignments. In particular ZNF16 and ZNF252 exhibit a very distant relationship to the other 8q24.3 ZNF genes and stay closest to each other but well separated by long branches. This behavior of the 8q24.3 ZNFs is different from the members of the human ZNF locus at 19q13.2 that form a closed clade with much narrower relationships. We also did a second series of analyses with extended mammalian ortholog sequences of the human 8q24.3 ZNF genes as well as the above mentioned pseudogene sequences at our 8q.24.3 locus. The results were qualitatively similar to the one described above in that the different 8q24 ZNF genes with their respective orthologs were always clearly separated in their own clades and not intermingling, indicating significant phylogenetic distance from each other (see Additional file 4). With respect to the residual ZNF sequences on the pseudogenes named pseudo 1 and pseudo 2, the phylogenetic analysis did not indicate close relationships to any 8q24.3 ZNF gene.

The 8q24.3 genomic sequences encoding isolated or disrupted KRAB domains (krab A1B1, A2B2) separated in the phylogenetic analysis of the KRAB domain from the other genes of that locus (Figure 4D and Additional file 4D). However, the krab A1B1 putative KRAB domain displayed the highest score with sequence identities of 56% (36/64) with ZNF251 when using TBLASTN. The krab A2B2 8q24.3 piece that represents a more degenerated KRAB domain showed best matches with two predicted ZNF genes (XM_498167 30/73 = 41%; XM_938315 28/67 = 41%) and then with ZNF7 (28/72 = 38%). Thus, these KRAB domains, especially krab A1B1 with the nearby pseudo1 ZNF sequence appear to be distant relatives of the other 8q24.3 KRAB domains. They might originally have been part of ancient locus members that lost their function long ago.

Despite the distant relationship TBLASTN searches of the reference human mRNA database with the different 8q24.3 ZNF protein sequences indicated that another member of the 8q24.3 locus was in general the closest relative based on overall sequence similarity. The closest human paralog of ZNF34 was ZNF250 (identities 268 out of 554 residues = 48%), that of ZNF517 was ZNF251 (identities 220/499 = 44%), and ZNF16's closest human relative was ZNF252 (identities 333/677 = 49%). The most similar paralog based on TBLASTN of ZNF250, ZNF251 and ZNF7 was ZNF184 at 6p21.3, that of ZNF252 was ZNF167 at 3p22.3-p21.1. Still, 8q24.3 members were not far off with respect to score (data not shown).

**Figure 5:**
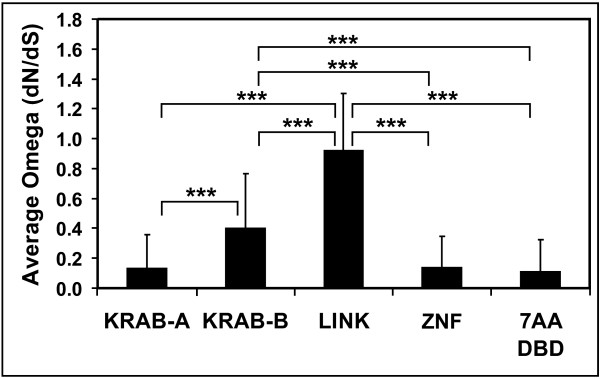
**Estimation of the evolutionary selection pressure on the different domains of the ZNF genes/proteins from 8q24.3 and their orthologs**. The histogram plots the average ω (= ratio between dN, the number of non-synonymous substitutions per non-synonymous site, and dS, the number of synonymous substitutions per synonymous site) for each domain, KRAB-A, KRAB-B, linker (LINK) and zinc finger array (ZNF, the consecutive sequence of all C2H2 zinc fingers of the gene/protein) of the ZNF genes/proteins. The data labeled 7AA DBD only consider the sequences -2 to 6 with respect to the a-helix of each C2H2 zinc finger for analysis, except for conserved position 4. The ω values of smaller than 1, equal to 1 or larger than 1 refer to negative purifying selection, neutral evolution or positive selection, respectively. Error bars represent the respective standard deviations. The means of the different domains were statistically compared by a T-test, the asterisks indicate the high significance (p < 0.0001).

As part of the phylogenetic analysis, synonymous and nonsynonymous substitution rates within an ortholog group of each 8q24.3 ZNF gene were determined with focus on the analysis of differences between the rates for the different domains, KRAB-A, KRAB-B, non-conserved linker and zinc finger array. Estimation of these rates was carried out using maximum likelihood (software PAML, [53,54] see Methods). The results for the individual pairwise comparisons of the four domains for each ortholog group are listed in Additional file 5. In order to generalize the findings, the average non-synonymous/synonymous substitution rate ratios were calculated for each domain (Figure 5). Overall, the zinc finger array of each ortholog group showed strong purifying selection (ω = dN/dS around 0.1) with mostly highly significant p-values. This result was not surprising since the conserved zinc finger framework residues need to be maintained in order to adopt the correct 3D structure. The KRAB domain appeared to be under purifying selection as well. However, our calculation argues that the purifying evolutionary pressure was generally stronger on the KRAB-A (ω around 0.15) compared to the KRAB-B domain (ω around 0.4). The linker part of the ZNF genes/proteins, which in case of the 8q24.3 ZNF sequences and their orthologs does not contain any conserved domain, followed neutral evolution (ω near 1). When we restricted the analysis to a stretch of sequence encoding the amino acids that mainly determine DNA binding specificity (see next paragraph), again, the values obtained showed strong purifying selection as for whole zinc fingers.

### Comparative analysis of zinc finger DNA binding domains

**Figure 6:**
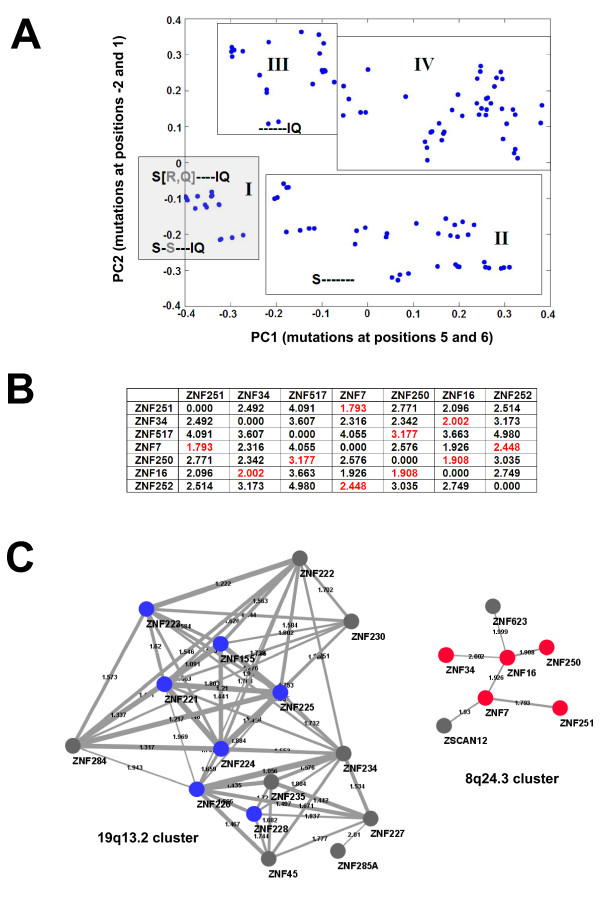
**Evaluation of the conservation of the C2H2 ZNF DNA binding domains**. (A) Principal component analysis of ZNF domains for conservation based on the 8-amino acid region from -2 to 6 with respect to the start of the α-helix of each finger (see text). Included are all individual fingers from the 8q24.3 ZNF proteins and their mouse and rat orthologs. The plot shows the first (PC1) against the second (PC2) principal component representing the variation in position 5 and 6 or -2 and 1, respectively. Negative values are indicative of lower variability and thus higher conservation. Plot areas that contain the same or similar 8-residue regions are boxed. Conserved amino acids are highlighted in single letter code at respective positions. Dashes indicate non-conserved residues. Additional file 7 details all peptides and their values/coordinates based on the boxed areas. (B) Pairwise ZNF matrix similarities between the 8q24.3 ZNF locus members (see text). Numbers in red indicate lowest values, i.e. highest similarities of each ZNF gene. (C) Detail of a paralog network founded on pairwise ZNF sequence similarities between all human C2H2 ZNF genes (see text). Nodes represent the individual genes (labeled by name), the edges describe their similarity. The thickness of the edges is proportional to the similarity and the value is given as label. A decrease in the value means an increase in similarity. Shown are the isolated 8q24.3 and 19q13.2 clusters of the network with 8q24.3 ZNF member nodes in red, and nodes of 19q13.2 members included in Figure 2 in blue. Network restricted to similarity values ≤ 2.01.

Specific amino acid residues positioned in the C2H2 zinc finger domain play a key role in determining their nucleic acid binding specificities. Based on the EGR1/Zif268 protein-DNA crystal structure, helical positions -1, 3 and 6 with respect to the start of the α-helix are especially important for DNA-binding specificity [17]. When comparing these important residues along each group of orthologs (from opossum to human, as available), it became immediately evident, that they are highly conserved (see graphical depiction in Additional file 6). Analysis of the mutational trends in this region of the α-helix between the 8q24.3 ZNF paralogues should help to clarify likely duplication scenarios and functional, i.e. nucleic acid binding residue, variability. To that purpose, principal component analysis of the conservation profile of the individual zinc finger sequences was performed. The analysis is based on the multiple alignment of the region encompassing the 8-residue long stretches from positions -2 to 6 with respect to the α-helix of each C2H2 zinc finger. Principal component analysis identifies new axes in a multiple alignment matrix by weighting positions with high co-variation and deemphasizing positions that show little co-variation with other positions. Positions in the stretch of binding residues most tightly connected with one another thus reflect correlated mutations that are under evolutionary selection and are more likely to be important for nucleic acid binding. The first principal component, plotted on the x-axis of Figure 6A, contains position 6 (weight -0.82) and the correlated position 5 (weight -0.44). The second principal component, plotted on the y-axis, is based on position -2 (-0.92) and 1 (-0.27). For both axes, zinc-finger domains sharing the prevalent amino acids in the respective positions are in the negative and genes with mutations in these positions in the positive regions. The sequences assigned to the different regions with the plotted matrix values are given in Additional file 7. Region I contains all zinc finger domains with a significantly overrepresented motif S [Q, R]S---IQ (a dash stands for any amino acid) with frequencies S(49%), [Q(35%), R(19%)], S(45%), I(44%), and Q(49%). S-----IQ is found in nearly all 8q13.4 genes (only ZNF7 has the mutational variant R-----IQ). The subgroups in region I represent the motif SQ----IQ present in individual zinc fingers of human/mouse/rat ZNF250, human ZNF16, ZNF34 and ZNF252, and its relative, the motif SR----IQ present in human/mouse/rat ZNF251 as well as human ZNF517 zinc fingers. Region I also includes the motif S-S---IQ found in C2H2 domains of human/mouse/rat ZNF251, and human ZNF34 and ZNF16. In zinc finger domains of region II the serine is still at position -2; however, mutations in positions 5 and 6 have occurred (e.g. zinc fingers ZNF7-12 SQ----I**Y**, ZNF7-2 SD----KH, while in region III the serine at -2 is lost, but the IQ at position 5 and 6 is conserved (e.g. ZNF7-4 **R**L----IQ). As expected, residues in the binding region of the 8q24.3 zinc finger domains are frequently modified to alter or refine their binding specificity; yet, specific amino acids at positions -2, 1 and 5, 6 are significantly overrepresented in the 8q23.4 sub-family of zinc fingers pointing to a common conserved framework of DNA binding.

Multiple alignments are a means to compare and relate zinc finger domains. However, the search for the best alignment is often complicated if different numbers of the highly conserved zinc finger motif are present in the sequences to be aligned. Therefore, we sought an alternative way to compare zinc finger sequence similarity and thus DNA binding specificity: We computed ZNF motif matrices for each 8q24.3 ZNF gene based on the codon-aligned individual zinc finger sequences (see Methods). These matrices were then used to calculate a pairwise similarity table between the genes (Figure 6B). The results argued that the closest relatives with respect to their DNA binding domain were ZNF7 and ZNF251. Yet several other similarities were not that far off, e.g. those between ZNF250 and ZNF16, ZNF7 and ZNF16 and ZNF34 and ZNF16. In contrast ZNF252 and ZNF517 exhibited the most distantly related matrices. In order to define the closest paralogs in human with respect to the ZNF domain we extended the analysis to all human C2H2 genes in the SysZNF database [40]. The computed pairwise similarity tables were used to construct ZNF similarity networks. The graphical display allowed us to visualize the closest paralogs with respect to ZNF matrix similarity in a cluster-wise fashion. In order to avoid overcrowding and pinpoint the closest relationships between ZNF genes we limited the network by chosen thresholds. With a threshold of 2.01 we found five of the seven 8q24.3 genes (ZNF7, ZNF16, ZNF34, ZNF250, ZNF251) within one sparse isolated cluster of the network construction (Figure 6B). In this cluster another member of the locus was always nearest neighbor as indicated by the similarity values. Thus, no other human ZNF gene was found that was more closely related in the ZNF domain. The cluster contained two additional ZNF genes with close similarities, ZSCAN12 from chromosome 6 (NCBI GeneID:9753) and ZNF623 (GeneID:9831) that, interestingly, is located upstream not far from our 8q24.3 locus at approximately 144.8 Mb. ZNF517 and ZNF252 are missing from the cluster and have closest relationships in their ZNF matrix with ZNF324B (GeneID:388569, at 19q13.43; similarity 2.466) and ZNF184 (GeneID:7738, at 6p21.3; similarity 2.189). As reference group we looked at the genes from cluster 19q13.2 again. They too were grouped within one cluster (Figure 6C) and displayed higher ZNF matrix similarities among themselves than the 8q24.3 sequences. The closer relationship within the group was also obvious from the higher interconnectivity, i.e. the gene nodes had a higher number of connecting edges. Thus, despite clear divergence, the analyses of the ZNF DNA binding domains provide evidence for a common evolutionary history of the 8q24.3 zinc finger genes.

### Tissue expression profiles of the ZNF genes in the 8q24.3 cluster

**Figure 7:**
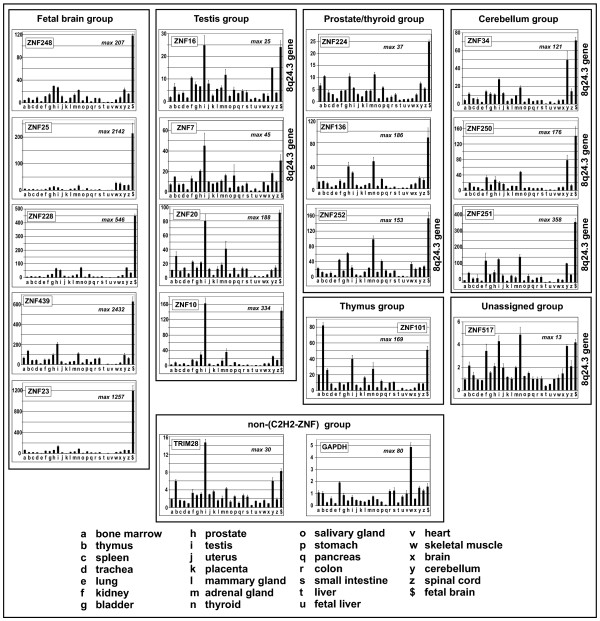
**Human tissue expression profiles of the 8q24.3 ZNF genes**. RNA expression profiles (labeled by gene names) of the seven 8q24.3 zinc finger genes compared to those of ten other C_2_H_2 _zinc finger genes, the KRAB domain-interacting partner TRIM28 and of the housekeeping gene GAPDH. Relative expression levels with the respective value for heart tissue set to 1 were determined with gene-specific assays by quantitative RT-PCR. The ordinate displays fold changes compared to heart. The different tissues as source for total RNA are labeled with letters and are indicated on the bottom of the bar plots. The maximal fold change between the tissue with the highest and the lowest values is indicated in the histograms ("max"). Bars represent means of three replicates ± SD within one assay. The profiles are arranged in subgroups: Strong overrepresentation in fetal brain compared to all other tissues ("fetal brain group"); values in testis similar or larger to those in fetal brain ("testis group"), prominent thyroid and prostate expression ("prostate/thyroid group"); highest expression in thymus ("thyroid group"); at least 50fold increased expression in cerebellum compared to heart and ratio fetal brain/cerebellum < 3.5 ("cerebellum group"). ZNF517 was unassigned, TRIM28 and GAPDH were put into a non-ZNF group.

The profiling of expression signatures provides a means to functionally compare the transcription regulatory sequences of the 8q24.3 ZNF genes. Therefore we recorded the gene expression profiles of the 8q24.3 ZNF genes in twenty-seven human tissues by quantitative RT-PCR and compared them to those of ZNF genes at other chromosomal localizations (Figure 7, see also individual data in Additional file 8). We also included the analysis of the KRAB-ZNF co-repressor gene TRIM28 and the housekeeping gene GAPDH. Each gene-specific assay gives a measure of the relative expression in the different tissues (fold changes with respect to the value in heart tissue that was set to 1). Transcripts of all 8q24.3 ZNF genes turned out to be detectable in all tissues, though, the results indicated overall strong expression differences in individual tissues for most of the genes. For the 8q24.3 genes the smallest overall deviations between tissues were seen with ZNF517 (maximal factor of 13.5: lung 0.36 and thyroid 4.86), whereas the highest differences were observed with ZNF251 (maximal factor of 358: heart 1 and fetal brain 358.31). Interestingly, in most cases the maximal relative expression levels of the 8q24.3 ZNF genes were reached in fetal brain, testis, cerebellum and thyroid. On the other hand, the 8q24.3 ZNF genes were generally the least abundant in heart, liver, fetal liver and lung. Noteworthy, similar expression profiles were also observed for the non-8q24.3 ZNF genes tested but not for the housekeeping gene GAPDH. Visual inspection of the profiles led us to subgroup the ZNF genes into 3 groups, based on tissues with the highest expression. ZNF7 and ZNF16 fell into the testis group, ZNF34, ZNF250 and ZNF251 into the cerebellum group and ZNF252 into the prostate/thyroid group. These expression signatures may hint at more prominent roles of these genes in the respective tissues. ZNF517 was left unassigned since overall expression changes were relatively small compared to the other genes. With respect to TRIM28 we recorded a profile that parallels the testis subgroup of ZNF expression signatures, yet the absolute expression changes between tissues were smaller (maximal factor ~ 30: liver 0.49, testis 14.65). Since relative quantification of the number of transcript molecules was performed, absolute differences in expression levels between different genes could only be estimated by the values of the respective threshold cycles (Ct; lower values mean higher number of starting RNA molecules; note the exponential relationship). Assuming similar PCR efficiencies, the RT-PCR data indicate that transcripts of all 8q24.3 ZNF genes were much less abundantly expressed than those of TRIM28 or GAPDH (see Additional file 8 for the primary quantitative PCR data). In heart tissue, e.g., most 8q24.3 ZNF gene transcripts were detectable at Ct values between 30 and 31 whereas TRIM28 and GAPDH displayed values of 22.9 and 19.13, respectively. In testis, the 8q24.3 ZNF RNAs were detected earlier at Ct values of about 24-26, demonstrating the higher expression in this tissue. Here, TRIM28 and GAPDH were detectable at Ct 19 and 19.6, respectively.

**Figure 8:**
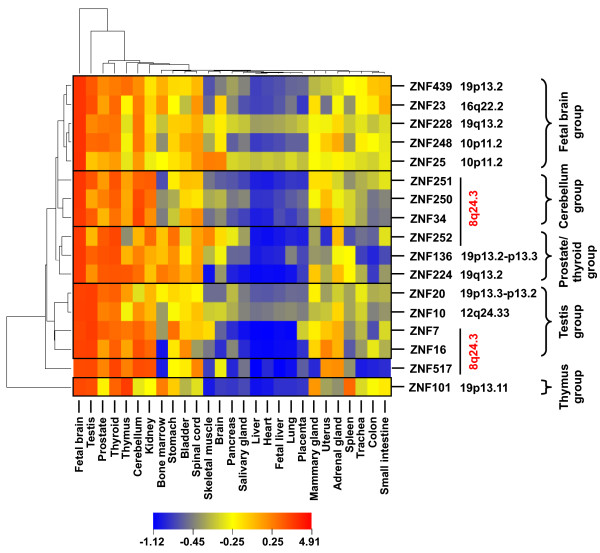
**Comparison of the tissue expression patterns of the seven 8q24.3 ZNF genes and those of ten ZNF genes located on other human chromosomes by hierarchical cluster analysis**. The RNA expression profiles are based on quantitative RT-PCR with total RNA from the indicated tissues as input (see Figure 7). The trees were calculated with the original relative expression data. The gene tree is based on the Pearson correlation distance and the tissue tree on Euclidian distance, both with single linkage. For the visual heatmap the data were standardized in order to fit them into the same scale, however, without changing the trees. Pseudo-color dark blue marks lowest, dark red highest expression. In addition to the gene names chromosomal localizations are indicated. The assignment of ZNF genes into tissue subgroups is based on subclades of the gene clustering and is consistent with Figure 7.

The different gene expression patterns were compared using hierarchical cluster analysis. Clustering was done for the genes under study as well as for the different tissues (values for distance measures are given in Additional files 9 and 10). As noted above, we observed overall high similarities of the ZNF expression profiles. Expression of 8q24.3 and non-8q24.3 ZNF genes is similar, but different from a group of unrelated genes (see Additional file 10). The heatmap in Figure 8 visualizes the results for the ZNF genes. Based on gene expression similarity clustering different subgroups can be identified, corresponding to the manually compiled ones in Figure 7. The genes of our 8q24.3 locus spread into several well-separated clades. Among the locus members, ZNF250 and ZNF34 displayed the closest tissue expression profiles. Furthermore, ZNF251 was the next related ZNF gene of 8q24.3. Similarly, ZNF16 and ZNF7 were closest neighbors. ZNF252 is the most distant with respect to the other 8q24.3 ZNF genes. This more distant relationship is indicated by a lower expression in cerebellum and a higher expression in prostate and thyroid. This places ZNF252 into a clade with ZNF136 and ZNF224. The data implicate that many KRAB-ZNF genes display similar tissue expression profiles. Genes from the same genomic locus with close profiles like ZNF250/ZNF34 or ZNF16/ZNF7 might share common regulatory sequences.

Considering tissue clustering, fetal brain and testis separate most from the other tissues. The latter fall into three tissue groups: Group I with usually high expression comprises prostate, thyroid, thymus, cerebellum and kidney. Group II with usually low expression consists of pancreas, salivary gland, liver, heart, fetal liver, lung and placenta. Group III shows moderate expression compared to the others. In terms of the three germ layers there does not seem to be overrepresentation of a particular one in a group. Still, the tissues are made up of different cell types and it remains unknown which cell types contribute most to the ZNF expression levels.

### Sequence similarity implies expression profile similarity

We compared the four different sequence similarity matrices with the expression profile similarity matrices. We concentrated on those KRAB domain encoding genes that are also included in the phylogenetic sequence comparisons (Figure 4). The results are summarized in Table 2. The three columns correspond to all sixteen ZNF genes, 8q24.3 and non-8q24.3 genes, respectively. Generally, there is a moderate, but nevertheless significant, positive correlation between sequence and expression profile similarities. Considering all sixteen genes, the ZNF domain sequence similarity correlates best with gene expression similarity (0.42). Interestingly, the correlation between sequence and expression similarity is highest for the six 8q24 KRAB-ZNF genes. This holds for all four different sequence similarities (all four rows), but is most striking for the KRAB domain sequence. So we conclude that KRAB-ZNF genes from the 8q24.3 locus with similar KRAB domain sequence have a very similar expression profile implying involvement in connected biological processes.

**Table 2:** Correlation between sequence and expression profile similarities

	Tissue	expression	**matrix**^**b**^
**Sequence similarity matrix**^**a**^	**all KRAB-ZNF**	**8q24.3 KRAB-ZNF**	**Non-8q24.3 KRAB-ZNF**

**cDNA**	0.27	0.62	0.15
**Protein**	0.39	0.62	0.38
**ZNF**	0.42	0.56	0.42
**KRAB**	0.22	0.71	0.16

^a ^Sequence similarity matrices are based on ClustalW alignments (IUB matrix for DNA or Gonnet matrix for protein sequences) and distance calculations using the Tamura-Nei method on DNA or the Poisson correction on the amino acid level, respectively, with the pairwise deletion option; calculated with MEGA4 (see Methods). Based on whole cDNA or full protein sequence or the ZNF or KRAB protein domains only.

^b ^Tissue expression matrices were calculated from the relative quantitative RT-PCR data using Pearson correlation distance.

Values represent Pearson correlation coefficients of matrix similarity comparisons (see Methods). All correlations are statistically significant (T-test, p < 0.0005), except cDNA and KRAB versus non-8q24.3 KRAB-ZNF (p = 0.068 and 0.062). Note that only ZNF genes with expressed KRAB domain from Figure 3 and available gene expression data were included.

### Computational comparison of the promoter regions of the seven human 8q24.3 ZNF genes

To investigate if expression profile similarities were due to promoter similarities (same transcription factor binding sites (TFBS)), we analyzed the proximal promoter regions of the seven ZNF genes for (i) general properties and especially (ii) common TFBS (see Methods for details).

The basic characteristics of the seven promoter regions are summarized in Table 3 (promoter sequences are given in Additional file 11): With the exception of ZNF34 the ZNF genes exhibit more than one major transcriptional start site (TSS) in the analyzed regions, three of them even four. The promoter regions overlap with CpG islands and experimentally defined binding regions of the RNA polymerase II core enzyme (see Additional file 12). Concerning classical core promoter elements, the seven ZNF promoter regions do neither contain classical Initiator (INR) elements, nor a TATA-box, a downstream promoter element (DPE) or a TFIIB recognition element (BRE) in a typcial configuration in relation to a TSS. Interestingly, an INR-DPE module can be found in the ZNF34 promoter region, although in an atypical constellation upstream of the TSS. The motif ten element (MTE), originally identified in *Drosophila *appears to act synergistically with INR [55]. Though lacking the INR, the ZNF34 promoter region displays this element in a typical position downstream of the TSS. Also the promoter regions of ZNF7, ZNF16 and ZNF517 show such an element less than 50 bp from the default location (for a detailed list of the sequence elements see Additional file 13). Finally, an X core promoter element (XCPE1) is found properly spaced to a TSS in the ZNF16 and ZNF252 promoter regions. This element can utilize free TATA-binding protein or the complete TFIID complex, usually in TATA-less genes and in conjunction with sequence-specific transcriptional activators [56].

**Table 3:** Characteristics of the proximal gene promoters^a ^of the seven human 8q24.3 ZNF genes

Properties		ZNF7	ZNF16	ZNF34	ZNF250	ZNF251	ZNF252	ZNF517
**Genomic location **^**b**^	Start	146023180	146147578	145984033	146098150	145952275	146199589	145994565
	End	146023872	146146948	145983392	146097511	145951559	146198940	145995192

**TSS **^**c**^		528	501	505	501	501	501	501
		569	514		531	617	505	528
		590	531		535		544	
		593			540		550	

**CpG island **^**d**^		yes	yes	yes	yes	yes	yes	yes

**Core promoter elements **^**e, f**^	Position^**g**^							

**INR**	-2	no	no	no	no	no	no	no
**TATA**	-31	no	no	no	no	no	no	no
**BRE**	-37	no	no	no	no	no	no	no
**INR-DPE**	-2/+28	no	no	atypical	no	no	no	no
**MTE**	+18	atypical	atypical	yes	no	atypical	no	yes
**XCPE**	-8	no	yes	no	no	no	yes	no

^a ^Promoters determined by Genomatix Gene2Promoter (see Methods); sequences given in Additional file 11

^b ^Based on human genome hg18

^c ^TSS positions relative to first nucleotide in the defined promoter sequence (= 1)

^d ^As annotated in the UCSC genome browser http://genome.ucsc.edu, see Additional file 12

^e ^Elements predicted with Genomatix MatInspector (see Methods)

^f ^Scoring: yes = within 10 bp of usual position; atypical = not at typical position, but within 50 bp; no = absent or far (>50 bp) from typical position

^g ^Usual default starting position of the element with respect to TSS (= +1), [55]

Next we looked for common sequence-specific TFBS among the promoter regions of the seven ZNF genes. We searched for predefined functional transcription factor modules using Genomatix ModelInspector (see Methods). This approach is based on the occurrence of usually two combined TFBS that have been experimentally shown to be functionally connected [57]. This strategy increases the likelihood to detect TFBS of biological relevance. Using a simple counting strategy on the thus obtained data we found eight module families that were shared in at least three of the seven ZNF promoter regions (Table 4; further details in Additional file 14). The lowest number of common modules was found in the ZNF34 promoter region. The eight module families cover 46 out of a total of 93 defined individual modules. The module families ETSF_SP1F, EGRF_SP1F and SP1F_ETSF occurred in six of the seven analyzed sequences. Certain families of TFBS like SP1-, ETS- und EGR-families occurred with high frequency in these modules. In order to pinpoint the involved elements we separated the eight module families into TFBS families and individual TFBS (Table 5). The most frequent TFBS from the modules, found in all seven promoter regions, were SP1 recognition sites. Almost as prominent was a motif for EGR1 that occurred in six regions. TFBS recognized by the ETS family of transcription factors were also frequent. However, the motifs belonged to three different family members, SPI1, ELK1 and Ets-1. Finally, modules contained recognition sites for AP-2 alpha and IKZF1 in five and four promoter regions, respectively. A graphical overview of the TFBS in the modules as well as the core promoter elements is shown in Additional file 15.

**Table 4:** TFBS module families with occurences in proximal promoter regions of at least three 8q24.3 ZNF genes

**Module family**^**a**^	ZNF7	ZNF16	ZNF34	ZNF250	ZNF251	ZNF252	ZNF517	Total	**Promoter count**^**b**^
**ETSF_SP1F**	2	1	0	2	1	3	2	11	6
**EGRF_SP1F**	3	0	1	2	2	1	1	10	6
**ETSF_ETSF**	0	1	1	0	3	1	0	6	4
**SP1F_ETSF**	1	1	0	1	1	1	1	6	6
**IKRS_AP2F**	1	0	0	0	1	1	1	4	4
**KLFS_SP1F**	1	0	0	0	1	1	0	3	3
**NFKB_SP1F**	0	0	0	1	1	0	1	3	3
**SP1F_AP2F**	1	0	0	0	0	0	1	3	3

^a ^Modules are functional combinations of (most often 2) TFBS [57]. Subcategories were combined to "module families" for counting. For more detailed lists see Additional file 14

^b ^Number of 8q24.3 ZNF promoter regions with matches of a given TFBS module family

Summarizing, our analysis shows that the proximal promoter regions of the seven 8q24.3 ZNF genes share core promoter properties like association with CpG islands and being TATA-less. Common TFBS modules could partly explain expression similarity of the ZNF genes.

## Discussion

Transcription factors are key elements in orchestrating gene expression programs with respect to development and differentiation and in response to the environment. A recent census of human transcription factors stated that out of approximately 1700-1900 transcription factor genes roughly 700 encode C2H2 zinc finger domains including, with a number of about 400, the largest subgroup of all, the KRAB-ZNF proteins [7]. There is evidence to suggest that KRAB zinc finger genes and thus KRAB-mediated transcriptional repression initially accrued 360 million years ago at a time when the first tetrapod/amphibian genomes were established [3]. The ZNF family continuously grew during phylogenesis with particular emphasis on the mammalian and therein the primate lineage, with often lineage-specific expansions [2,58,51,5,6,60]. The expansions are considered the result of repeated tandem gene duplication followed by diversification. Gene duplication is a key mechanism in driving evolution by providing opportunities for the selection of new phenotypes. Upon duplication the gene copies might diversify, thus developing new functionalities (neo- or subfunctionalization), they might contribute to genetic robustness or one of the copies might be lost (for review see [61]). Neo- and subfunctionalization can be brought about on different levels: Mutations in protein coding sequences may confer novel properties e.g. for transcription factors altered DNA binding sites and thus modified target gene lists. Changes in regulatory regions may lead to quantitative and qualitative expression differences like altered tissue expression profiles. A recent study on a large set of human transcription factors supports these notions [62]. Both, positive selection between paralogs for altered C2H2 zinc finger DNA binding domains [59,6] as well as diversification of the expression patterns between paralogs [51] has been shown for the (KRAB) ZNF family. In a study of coding sequence polymorphisms identified in humans compared to chimpanzee, the KRAB zinc finger gene family was classified as having an excess of rapidly evolving genes, with an enrichment for positively selected genes [63].

Here we focused on the human 8q24.3 zinc finger cluster comprising seven members near the telomere. Our phylogenetic analysis in mammals revealed both, considerable evolutionary pressure to keep effector domain structure as well as ongoing evolution: Purifying selection on the KRAB and the zinc finger domains, including the major residues influencing DNA binding specificity were indications of the conservation in mammals and thus a likely functional importance of the 8q24.3 ZNF genes in biological processes. Evolutionary constraints on the zinc finger region and purifying selection between orthologs has been generally noted for the C2H2 family [6]. In contrast, the lack of functional ZNF16, ZNF34, ZNF252 and ZNF517 ortholog genes in the rodent lineages pointed to persisting evolutionary dynamics. ZNF252 is of particular interest. It appears that even within the primate lineage it can encode a fully functional KRAB-ZNF protein (in rhesus monkey), a protein with a disrupted KRAB domain and truncated C2H2 zinc fingers (in human) or only a remnant without KRAB and ZNF sequences (in chimpanzee). Sequences homologous to LINE/SINE repeats in the human and chimpanzee orthologs might be responsible for the disruption of the open reading frame in the 5' part. ZNF16 remained the only 8q24.3 locus member without evidence of containing a KRAB domain in a mammalian species.

Since human C2H2 ZNF genes probably originated from common ancestors as products of gene duplication, they most likely retained common structural and transcriptional regulatory features that should be apparent in the family members of established ZNF clusters. Our alignments/tree building, reciprocal database searches and ZNF domain characteristics revealed that the ZNF genes from 8q24.3 generally share higher similarities to members of their own locus than to ZNF genes in other genomic loci. Thus, the seven ZNF genes comprise the closest paralogs for each other. They appear to form a rather remote genomic locus without close ties to other ZNF clusters. Furthermore, in contrast to other clusters, e.g. the one at the 19q13.2 locus described before [3] and used as an outlier group in the present study, the 8q24.3 ZNF genes show considerably less phylogenetic relatedness within the cluster. One explanation for this relative distance within 8q24.3 could be that duplication events leading to the paralogs happened quite early in mammalian evolution, most likely more than 130 million year ago before the split of *Theria *and *Eutheria*. The high degree of conservation in dog, cow, mouse/rat and human in combination with the location in syntenic regions argues that the 8q24.3 ZNF locus is as old as the *Eutheria*. The robustness of at least three members of this locus (ZNF7, ZNF250, ZNF251) is probably due to essential functions that are conserved during mammalian evolution. The ZNF252 ortholog found in the marsupial opossum raises the likelihood that the locus existed even before in the *Theria*. The fact that we were currently unable to define other orthologs in opossum is probably due to the preliminary state of the genome assembly of this species. Without data from other phylogenetically older species than mammals it is difficult to assess which gene might have been descended from a more ancestral gene. Furthermore, it remains unclear from which ancestral locus the 8q24.3 locus originally derived.

Expression profiles are indirect means for the comparison of regulatory regions of different genes. The recorded tissue expression profiles of the 8q24.3 ZNF genes showed overall relatively similar patterns in that mostly the same tissues displayed the highest or lowest relative expressions, respectively. This implies common regulatory principles, e.g. similar cis-acting elements or transacting factors. Yet, subgroups could be distinguished, too. A possible subspecialization after duplication, while still showing overlap or even redundancy, was conceivable. In order to gain insights into the gene control regions of the seven 8q24.3 ZNF genes we performed a computational comparison of their proximal promoter regions with focus on common properties and elements. There was evidence that the promoters can be classified as TATA-less and CpG island-associated as well as (with the exception of ZNF34) displaying multiple start points for transcriptional initiation. Thus, they would mostly fit a class of core promoters dubbed "dispersed" that are evolutionary younger and more common in vertebrates and whose exact mechanisms of regulation by transcription factors are less understood than those of the class termed "focused" [55]. The most prominent individual TFBS module discovered in six of the seven ZNF promoter regions (not in that of ZNF16), was made up of EGR1 and SP1, both C2H2 zinc finger proteins. The two factors have been shown to be able to act reciprocally on promoters in this module configuration, i.e. SP1 as transactivator and EGR1 as repressor [64]. The interrelationship between these two factors was also shown by *in vivo *occupancy changes in genome-wide studies during monocytic differentiation [65]. SPI1, an ETS-domain transcription factor also known as PU.1 and with essential functions during hematopoiesis [66], was found as part of modules in five ZNF promoter regions. Interestingly, genome-wide experimental investigation of SP1, EGR1 and SPI1 binding sites in the above mentioned cell model of monocytic differentiation [65,67] corroborated the potential functionality of most of our bioinformatically determined sites: We accessed the data through the online genome explorer of the Genome Network Platform http://genomenetwork.nig.ac.jp/. The transcription factor occupancies represented there indicate e.g. binding of SP1, EGR1 and SPI1 to the ZNF251, ZNF250 and ZNF252 promoter regions, but not to that of ZNF16. This coincides with our predictions. SP1 is a ubiquitously expressed transcription factor involved in many processes through transcriptional regulation of numerous genes [68]. The immediate-early response gene EGR1/Zif268 also has been implicated in many processes. In light of the strong expression of 8q24.3 ZNF genes in fetal brain and also cerebellum it is noteworthy that EGR1 was described to also play a role in spatial memory [69]. Since transcription factors have a high potential for functional pleiotropy based on broadly defined DNA binding specificity, crosstalk with each other and dependence on cell and tissue specific influences, it will be pivotal to determine the roles of the TFBS detected in the 8q24.3 ZNF genes experimentally. The closest pairs with respect to their expression patterns were ZNF34/ZNF250 and ZNF16/ZNF7 (see cluster analysis in Figure 8 and expression similarity calculations in Additional file 10). Our analyses of the proximal promoter regions revealed that ZNF34 shares one TFBS module with ZNF250 and ZNF16 shares two TFBS modules with ZNF7 (see Table 4). This could partly explain expression similarity, but regulatory elements from outside the proximal promoter regions might also contribute. Furthermore, other regulatory principles like chromatin organization or regulation by small RNAs might also play a role. A next step for further elucidation would be an analysis of the expression profiles of the corresponding transcription factors.

Surprisingly, KRAB-ZNF genes from other genomic loci exhibited similar tissue signatures as well, raising the possibility that there are underlying causes for these patterns of ZNF genes. It is generally assumed that higher expression of a gene in one compared to another tissue points to a more important function in the tissue with the more prominent transcript levels and may be connected to tissue function [70,71]. Thus, our recorded profiles especially emphasize fetal brain and, for particular genes of the familiy, also testis, cerebellum, prostate and thyroid as tissues in which the examined ZNF genes might serve important roles (see Figures 7, 8). Interestingly, tissues like heart and liver display consistently low levels of expression of the seventeen tested ZNF genes. Therefore, one might infer that (KRAB) ZNF proteins are predominantly influencing morpho-/organogenic processes of organs such as the brain that have been more strongly modified during tetrapode to primate evolution than liver and heart.

Fetal brain is a tissue undergoing complex developmental differentiation processes, notably neurogenesis. Testis as well is characterized by a major differentiation program, spermatogenesis. Both tissues display, compared to other tissues, above average features of transcriptional regulation: Fetal brain and testis belong to the tissues with the highest number of alternative splicing events [72], testis tissue expresses a large number of tissue-specific exons [73] and has the highest frequency of tissue-specific putative alternative promoters [74]. A recent detailed global transcriptome analysis of human mid-fetal brain regions revealed a high percentage of expressed genes with a large number of specific gene expression and alternative splicing patterns [75]. The transcript patterns corresponded to anatomical and functional subdivisions of brain. C2H2 ZNF genes were sometimes also found to be enriched in some fetal brain regions. With respect to the 8q24.3 ZNF locus, most members were not scored as over/underrepresented in a particular brain region. Only ZNF250, along with at least 15 other C2H2 ZNF genes, was underexpressed in thalamus tissue compared to average expression in other regions. Prominent cell types of testis comprise the epithelial Sertoli cells, the androgen producing Leydig cells and the developing germ cells, the spermatocytes. The latter cells display transcriptional properties that are conceptually different from somatic cells: Distinctive features include use of alternative promoters, alternate starts sites, use of alternate transcription factors, altered genome packaging and arrest of transcription during spermatogenesis [76]. Involvement of KRAB-ZNF proteins in spermatogenesis can be inferred from the fact that their co-repressor protein TRIM28 was shown to be required for the maintenance of this process in mouse [31]. In addition to reports on prominent expression of KRAB-ZNF genes in testis in embryos and adults [77-81], there is accumulating evidence that KRAB-ZNF genes play a role in sex determination, spermatogenesis and imprinting [35,81]. We therefore assume that KRAB zinc finger genes/proteins play especially important roles in differentiation processes in fetal brain and testis, e.g. by switching off distinctive target genes in distinct temporal and spatial patterns. In this respect it is noteworthy that KRAB-ZNF genes can be found in expression signatures of stem cells and change in response to reprogramming and Oct4 knock-down [82-84]. A recent publication provided even evidence for the mouse KRAB-ZNF protein ZFP809 as a stem-cell-specific retroviral restriction factor [85].

Comparison of sequence similarities with expression pattern similarities uncovered higher positive correlations for the 8q24.3 locus KRAB-ZNF group than for the more heterogeneous non-8q24.3 group (see Table 2). They probably reflect the closer phylogenetic relationships in the 8q24.3 group. Notably, cDNA and KRAB domain similarity correlations deviate a lot between the two groups. These findings imply that sequences within the cDNA and in particular those encoding the KRAB domain of the 8q24.3 genes are somehow involved in specifying the expression profiles observed. It is probably more likely that cis-regulatory sequences near the KRAB-encoding exons rather that the KRAB exon sequences themselves contribute to this phenomenon. Such surrounding sequences might form an evolutionary linked unit with the KRAB exons.

Expression profiling is constrained by the samples and methodologies that are being used. As in many published studies on human tissue-specific expression analyses (e.g. [70,51,72]) we depended on commercial RNA samples from human materials that represented pools from different individuals. With respect to the methods, the amplicons our quantitative PCR relied on do not likely interrogate all possible transcripts of a gene nor do they necessarily measure the same isoforms that are measured by microarray applications. A polyA-based priming step in many labeling protocols for microarray hybridization is an example: It leads to a considerable 3' bias [29]. Sensitivity of quantitative PCR exceeds that of the classical microarray platforms [86]. High sensitivity is of particular interest for the robust detection of (KRAB) ZNF transcripts which are often low abundant. Therefore, despite the wealth of published gene expression data, problems of bias and sensitivity affect the expression profiling of (KRAB) ZNF genes and result in an incomplete picture of their transcript patterns in cells and tissues. Detailed, high sensitive profiling of all transcripts of a (KRAB) ZNF gene will be instrumental in understanding their spatial and temporal patterns of expression. As for most other members of the ZNF superfamily, information on the proteins and function of the 8q24.3 ZNF genes is scarce. One study discovered a complex of human ZNF7 protein with autoantigen L7 and ribosomal protein S7 [87]. ZNF16 was proposed to have a role in erythroid and megakaryocytic differentiation and to harbor a transactivation domain N-terminally to the zinc finger domain [88,89]. Another report associated ZNF250 with cell proliferation [90]. Recently, a localization study on endogenous mouse ZNF250/Zfp647 uncovered a novel type of nucleoplasmic body containing KRAB-ZNF proteins and TRIM28 in differentiated cells [91].

## Conclusions

In summary, our analysis characterizes the six KRAB and one KRAB-less members of the human 8q24.3 C2H2-ZNF locus as an old mammalian paralog group that probably already existed around the split of *theria *and *eutheria *130 million years ago. Subfunctionalization of a more ancient presumable ancestor(s) included modified DNA binding specificities and qualitative and quantitative expression differences. Ongoing evolution is evident from the loss of four orthologs in the murine lineage and truncations of one member in primate species. This raises the question what the primary functions of the lost/truncated genes might be that turned out to be dispensable in some species. The measured gene expression profiles highlight in particular fetal brain as a primary tissue that utilizes KRAB-ZNF gene functions. We assume that expanded and altered target gene repertoires in conjunction with diversified expression patterns of the KRAB zinc finger protein family in general contributed to amelioration of differentiation and developmental programmes during speciation. The expansion of KRAB-ZNF genes in mammalian evolution resulted in an increase in diversity and complexity of transcriptional gene regulation by modulating and modifying gene expression signatures from cell type to cell type in space and time.

## Methods

### Fine-mapping of ZNF gene cluster on human chromosome 8q24.3

FISH techniques were employed to fine-map PAC clones assigned to human chromosome 8q24.3 [44]: The apparent colocalization of the PAC clones on chromosome 8q24.3 was analyzed using two or three differently labeled probes on interphasic nuclei to obtain a relative order. In a second approach using molecular combing technologies, genomic DNA was obtained from a control lymphoblastoid cell line. Cells were embedded in low-melting agarose blocks (10^6 ^cells per block equivalent to 6 μg of DNA)[92]. DNA was diluted to approximately 1.5 μg/ml in 400 mM MES-NaOH pH5.5 (Sigma). Combing was performed using the Molecular Combing Apparatus™ (Institut Pasteur, Paris, France and Genomic Vision) on silanized surfaces[92]. Each probe (1 μg) was labeled by random priming (Bioprime DNA labeling system, Invitrogen) either with fluorescein-11-dUTP (FluoroGreen, Amersham), digoxigenin-11-dUTP (Roche Diagnostics) or biotin-14-dCTP (Invitrogen) and purified with the QIAquick PCR purification kit (Qiagen). Nine hundred nanograms of each probe were competed with 12-fold excess human Cot-1 DNA (1mg/ml; GibcoBRL). Hybridisation was performed as previously described [92,93]. Biotinylated, digoxigenin- and fluorescein-labeled probes were detected with AMCA, Texas Red and fluorescein respectively, using four or five successive layers of antibodies as follows: (1) and (3) AMCA-avidin (diluted 1:10; Vector Laboratories) + Texas Red-conjugated mouse anti-digoxigenin (1:50; Jackson ImmunoResearch) + rabbit anti-fluorescein (1:10; Molecular Probes); (2) and (4) biotinylated goat anti-avidin (1:50; Vector Laboratories) digoxigenin-conjugated goat anti-mouse IgG (1:10; Roche Diagnostics) + fluorescein-conjugated goat anti-rabbit IgG (1:10; Valbiotech); (5) AMCA-avidin (diluted 1:10; Vector Laboratories) + Texas-Red-conjugated mouse anti-digoxigenin (1:50; Jackson ImmunoResearch). Signals were observed under an epifluorescence Leica DMRB microscope and captured with IPLab Spectrum-SU2 software (Vysis, Downers Grove, IL, USA) using an NU 200 CCD camera (Photometrics, Tucson, AZ, USA). Image analyses were performed with CartographiX software (X. Michalet, Institut Pasteur, Paris, France and Genomic Vision).

### Sequencing and genomic sequence assembly

The complete sequences of 13 ZNF PAC clones belonging to the chromosome 8q24.3 ZNF contig (see Figure 1) are available in the Jena databases http://genome.imb-jena.de/. For genomic sequencing, the PACs were nebulized and subcloned into M13mp18 vector [94]. At least 3000 plaques were selected from each clone library and shotgun sequenced using dye-termination, ThermoSequenase (Amersham) and universal M13-primer (MWG Biotech). The gels were run on ABI-377 sequencers and data were assembled and edited using the GAP4 Program [95]. Initial genomic DNA sequence analysis was performed using the automated sequence annotation system RUMMAGE [96]. The final assembly is represented in GenBank accession numbers AF186192 and AF235103.

In order to look for all KRAB-A, KRAB-B and C2H2 zinc finger domains in the human 8q24.3 region the sequence from GenBank accession AF23513 was subjected to HMMER version 2 search http://hmmer.janelia.org/ with the matrices defining the KRAB-A and C2H2 zinc finger domains taken from PFAM http://www.sanger.ac.uk/Software/Pfam/; [97]) and KRAB-B by the HMMBUILD part of the HMMER program with an alignment of KRAB-B taken from known sequences [19]. Only hits with scores higher than 5 and E-values smaller than 0.0001 were considered.

### Analysis of gene models and orthologs

Originally defined gene sequences and gene models obtained during sequence assembly (see above) were used to do in-depth searches in various public databases. NCBI BLASTn http://www.ncbi.nlm.nih.gov/BLAST/ was used to find cDNA and EST sequences in GenBank that belong to a particular gene model and to find exon/intron borders in the genomic sequences of the NCBI genome build 36.1. In parallel using either previously defined cDNA sequences or GenBank accession numbers the resources ENSEMBL http://www.ensembl.org and Acembly http://www.ncbi.nlm.nih.gov/IEB/Research/Acembly/ were accessed to compare gene models. Furthermore the BLAT search algorithm of the UCSC genome browser was used to find the best alignments in the human genome http://genome.ucsc.edu/cgi-bin/hgBlat. All the information was integrated into building gene models for seven C2H2 zinc finger genes on 8q24.3 that represented in general the longest open reading frame supported by cDNAs and ESTs. The exact position of the gene models within the genome was determined by mapping the sequence to human reference sequence assembly hg18, March 2006 (UCSC browser) that is based on NCBI Build 36.1 http://genome.ucsc.edu/. The search for orthologs in chimpanzee, mouse and rat was done in a similar way using BLASTn, BLASTp, BLAT (nucleic acid and protein levels) and information on syntenic regions (ENSEMBL). Alignments of presumable orthologs using T-Coffee http://www.tcoffee.org/[98] served to inspect the similarities. Reciprocal BLAST searches against the human database were done to confirm the closest relationship with the presumable ortholog. Since the mouse sequences were far better supported by cDNAs and ESTs, they also served to define the respective rat genes and the transcript they presumably encode by searching for homologies in the rat genomic sequence. Genomic annotation was again based on the UCSC browser (for chimpanzee pantro2, March 2006 based on GSC build 2.1; for mouse mm9 July 2007 based on NCBI 37; for rat rn4 November 2004 based on HGSC build 3.4; see UCSC website for more details). Furthermore, we searched the mouse and rat genomic regions that are syntenic to our human 8q24.3 ZNF locus for remnants of KRAB-A, KRAB-B and C2H2-ZNF encoding sequences using HMMER as described above for the human sequence. After setting up the gene models in human, chimpanzee and, if available, also in mouse and rat, we used all the information to find orthologs in other mammalian species by the means described above. Not surprisingly, in many cases the gene models in rhesus monkey (UCSC genome assembly rheMac2), dog (canFam2), cow (bosTau4) and opossum (monDom5) suffer from the early stage of genome assembly and the lack of known cDNAs. In most cases we were left with deduced coding sequences inferred from homology comparisons. All transcript and protein sequences derived from these combined efforts are given in Additional files 1 and 2, respectively.

### Phylogenetic analyses, alignments and clustering

Multiple alignments of protein and cDNA sequences were generated with ClustalW.1.83 using Gonnet/IUB matrices and default parameters [99]. Phylogenetic trees were calculated by the neighbor-joining algorithm [100]. Bootstrap values of the trees were calculated for 1000 trials using the MEGA4 program package [101]. Given the close evolutionary relationship of duplicated genes, a maximum parsimony analysis was performed for protein sequences using the program PROTPARS of the PHYLIP package [102]. Since internal rearrangements of zinc finger domains were expected from domain organization and initial neighbor-joining tree inspections, this analysis was best suitable for the KRAB domain. In addition, all zinc finger domains were analyzed by principal component analysis individually (see below). Other human C2H2 zinc finger genes and distant relative *Xenopus laevis *Xfin [GenBank:X06021, GenBank:EU277665] were included in some analyses as outgroups (see respective figures and Additional files 1 and 2 for accession numbers and sequences).

### Calculation of substitution rates between ortholog gene sequences

To estimate selective evolutionary pressure on different parts of the gene sequences encoding the different domains we employed the YN00 module of the PAML software package version 3.14 [53,54]. The codon-based alignments of the domains/regions KRAB-A, KRAB-B, linker ("LINK") and zinc fingers ("ZNF") were done separately using ClustalW 1.83 [99]. We calculated the ratio ω of the number of non-synonymous (dN) and the number of synonymous (dS) substitutions per site for each pairwise comparison in a multiple alignment of ortholog sequences. Values of ω < 1, = 1 and > 1 refer to negative purifying selection, neutral evolution or positive selection, respectively. Z scores as output of the Z-test of selection were calculated from: Z score = (dN-dS)/sqrt (dN_SE^2 ^+ dS_SE^2^) with SE being the standard deviation of the mean. The accompanying p-value was determined through: p = 1-normsdist (abs(Z score)).

### ZNF motif matrix analysis

DNA sequences encoding zinc finger motifs of individual ZNF genes were compared to all ZNF sequences in the SysZNF data base ([40]) and ranked according to their similarity with the tool ZNFMotifMatrix (see SysZNF webpage at http://lifecenter.sgst.cn/Utilities2007/znfMotifMatrix/: First, ZNF motif DNA sequences of each ZNF gene were aligned on the codon level. Then, a position-specific scoring matrix (PSSM) was constructed for this multi-alignment. Fortunately, the ZNF motifs have a fixed length of ~ 72 nucleic acid residues, facilitating comparisons of PSSMs. We built PSSMs for all human and mouse ZNF genes and obtained genome-wide PSSMs datasets. Thus, we can compare the PSSM of a selected ZNF gene with the PSSMs in the pre-constructed PSSM datasets and find the most similar matrices (Euclidean distance), i.e the closest relative with respect to C2H2 zinc finger sequences. For visualization we employed Cytoscape 2.6.2 (http://www.cytoscape.org, [58])

### Analysis of binding residues

Principal component analysis (PCA) was applied to the multiple sequence alignment of the 8-residue stretch of individual C2H2 zinc finger domains starting after the highly conserved phenylalanine and ending before the first zinc coordinating histidine. This region is involved in determining DNA/RNA-binding specificities as confirmed by 3-D structure analysis [13]. These residues are numbered -2, -1, 1, 2, 3, 4, 5, and 6 with respect to the start of the α-helix of a C2H2 zinc finger. Here, an N × 8 matrix was computed containing explicitly the degree of conservation at each position of the aligned 8-residue binding stretch for each of the N zinc finger domains. For example, at position -2 42% of the domains contained a serine, thus the conservation matrix was filled with 0.42 for all domains with serine at this position. Principal component analysis of the conservation matrix was then performed using the Matlab Statistics toolbox (The Mathworks Inc., MA, USA). Pre-analysis revealed that the positions 2 (73% S) and 4 (93% L) are highly conserved and thus uninformative with respect to functional changes; these were removed from the conservation matrix before principal component analysis. The plot of the first against the second principal component representing the variation in position -2 and 1 or 5 and 6, respectively, was used for evolutionary analysis of functional residues.

### Tissue expression profiling of zinc finger genes

Total RNA from twenty-seven different human tissues was purchased from Clontech. First strand synthesis was performed with 5 μg total RNA and the Superscript II reverse transcriptase (Invitrogen) primed with random hexamer oligodeoxynucleotides according to the recommendations of the supplier. Quantitative real-time PCR was performed with the TaqMan 7700 cycler (Applied Biosystems) with an input of first strand sythesis product equivalent to 25 ng total RNA. The probe/primer combinations for the gene-specific amplicons were either designed by the PrimerExpress 1.0 software (Applied Biosystems) or purchased in a ready-to use format (Applied Biosystems TaqMan Gene expression assays). The assays for ZNF251, ZNF34, ZNF517, ZNF7, ZNF250 as well as ZNF10, ZNF136, ZNF439, ZNF248 and ZNF25 detected sequences in the KRAB A and B exons, thus monitoring all KRAB-containing transcripts. The assays for the KRAB-ZNF genes ZNF23 and ZNF101 probe the zinc finger exon sequences and for ZNF20 the 3'UTR. The assay for ZNF16 spans two exons (exon 2, 3) as does the one for ZNF252 (exons 1,2 and 4,5, respectively, depending on gene model: see Figure 2A). Order numbers of the commercial as well as probe/primer sequences of the assays we developed are given in Additional file 8. Real-time PCR runs were performed with the TaqMan PCR core reagent kit (part N808-0228, Applied Biosystems) under the supplier's recommended standard conditions. Relative RNA expression levels are proportional to [1/(2 exponent Ct] (see Applied Biosystems 777802-002: ABI prism 7700 sequence detection system user bulletin 2) with Ct being the threshold cycle of the reaction. Ct was determined from a log-linear plot of the PCR signal (fluorescence) versus the cycle number by the instrument's software. Relative expression values were computed for each gene and referred to the value in heart tissue which was set 1, since heart tissue showed the lowest expression signals in the majority of assays. Since the expression levels of the housekeeping gene GAPDH changed considerably (as much as by a factor of ~ 80; lowest relative expression 0.06 in pancreas, highest 4.87 in skeletal muscle) normalization based on its expression was omitted and comparisons were based on RNA input.

For cluster analysis of the tissue expression profiles of the various ZNF genes the original relative quantitative RT-PCR values were used to calculate trees: The ZNF gene tree is based on Pearson correlation distances and the tissue tree on euclidean distances, both with single linkage, using R software http://www.r-project.org. The corresponding heatmap (Figure 8) was drawn with CIMminer http://discover.nci.nih.gov/cimminer.

### Comparison of expression and sequence similarities

The analysis of sequence similarities for the different ZNF DNA/protein sequences was based on distance matrices obtained with the MEGA4 software package [101]. Alignments were made with ClustalW 1.83 [99] using IUB (DNA) or Gonnet (protein) matrices. Distance calculations employed the Tamura-Nei method [103] on DNA or Poisson correction on the amino acid level, both with the pairwise deletion option of missing data. Four different similarities based on the sequences of cDNAs, of whole proteins, and of only the ZNF- and the KRAB domains, respectively, were computed. Similarities of expression profiles were quantified by Pearson correlation coefficients. In order to examine whether sequence similarity was associated with expression profile similarity, we quantified the similarities between the different similarity matrices by the Pearson correlation of all corresponding matrix elements. We considered all 16 ZNF genes with available expression data and KRAB domains. Since only the underlying similarity matrices were analyzed, this approach is independent of any tree-reconstruction method [104]. All correlations were calculated using Mathematica (Wolfram Research Ltd.).

### Computational analysis of promoter regions

The proximal promoter sequences of the seven ZNF genes were extracted with the Gene2Promoter software module of the GenomatixSuite (Genomatix, Germany; sequences given in Additional file 11). The software takes into account known transcripts as well as recorded CAGE (cap analysis of gene expression) tags that correspond to the 5'-end of capped transcripts and thus provide a picture of TSS. The promoter regions encompass 500 bp upstream and 100 bp downstream of a TSS. If more than one TSS was present the sequence stretch was calculated from the most upstream and most downstream sitting sites, respectively. For comparison, we annotated the extracted regions with the UCSC Genome Browser using tracks for known promoter features. Examples are CpG islands, RNA polymerase II chromatin immunoprecipitation footprints and TSS predictions by other software (see visualizations in Additional file 12). Core promoter elements were predicted by Genomatix MatInspector 8.0 with default settings using position weight matrices for respective TFBS [105] and are listed in Additional file 13. Then the promoter regions were subjected to the Genomatix Suite tool ModelInspector version 5.6.5 using default conditions. This tool employs predefined functional modules that usually combine two TFBS in a conserved configuration and with known synergistic, additive or antagonistic relationship [57]. The current promoter module library (version 5.1) consists of 657 vertebrate regulatory modules that on average detect less than 1 match per 10000 bp in genomic sequence. The modules combine redundant elements since they group TFBS into families covering closely related transcription factors. For simplicity, we further consolidated modules into module families based on the presence of the same TFBS families and used the data for a simple counting strategy: A module family was taken into account if it was predicted in at least 3 of the 8q24.3 ZNF promoter regions. Such modules are listed in detail with their TFBS in Additional file 14 and served as input to generate the summarized data of Tables 4 and 5.

**Table 5:** Frequent individual elements from TFBS modules in proximal promoter regions of the 8q24.3 ZNF genes ^a^

**TFBS family**^**b**^	**Individual TFBS**^**b**^	**Factor**^**c**^	ZNF7	ZNF16	ZNF34	ZNF250	ZNF251	ZNF252	ZNF517	Total	**Promoter count**^**d**^
**AP2F**	AP2.02	AP-2α	2	0	0	1	1	1	2	7	5

**EGRF**	EGR1.02	EGR1	3	0	1	2	2	1	1	10	6

**ETSF**	CETS1 P54.01	c-ETS1 (p54)	1	0	0	0	0	1	1	3	3
	ELK1.02	ELK1	1	4	0	0	3	1	0	9	4
	SPI1_ PU1.02	SPI1	0	0	1	2	2	3	1	9	5

**IKRS**	IK2.01	IKZF1	1	0	0	0	1	1	1	4	4

**SP1F**	SP1.01	SP1	3	1	1	4	3	3	3	18	7
	SP1.03	SP1	2	0	0	3	3	3	1	12	5

		Total	13	5	3	12	15	14	10	72	

^a ^Only TFBS module families that occur in promoter regions of at least three 8q24.3 ZNF genes served as input for counting. Listed are only TFBS that were found in at least three different promoter regions. The position in a module was not considered. It does not exlude the presence of additional binding sites within the promoter sequences.

^b ^TFBS family and individual TFBS matrices based on Genomatix Matrix Library 8.1. Derived from the results using Genomatix ModelInspector. Detailed listing and sequences in Additional file 14

^c ^Transcription factor recognizing the respective TFBS

^d ^Number of 8q24.3 ZNF promoter regions with matches of a given TFBS

## Abbreviations

KRAB: Krüppel-associated box; ZNF: zinc finger; TFBS: transcription factor binding sites; TSS: transcriptional start site; FISH: fluorescence in situ hybridization

## Authors' contributions

PL performed the gene expression analysis, compiled the sequence information, participated in the bioinformatic analyses and wrote the draft of the manuscript including preparation of figures, tables and additional files. Genomic clone preparation, sequencing and initial assignments and annotations of the human 8q24.3 locus was done by DK, GW, MFRM, SA, and SG. SD, TW, GD and YL performed bioinformatic analyses and contributed to the writing of the manuscript. HJT designed and coordinated the study, participated in bioinformatic analyses and in writing the manuscript. All authors read and approved the final version of the paper.

## Supplementary Material

Additional file 1 **Nucleotide sequences**. Compendium of all nucleotide sequences used for analysis. Click here for file

Additional file 2 **Amino acid sequences**. Listing of all amino acid sequences used for analysis. Click here for file

Additional file 3 **Orthologs of human 8q24.3 ZNF genes at syntenic genomic regions in mouse and rat**. Table summarizing accession numbers and genomic localization of the mouse and rat orthologs. Click here for file

Additional file 4 **Phylogeny of the human 8q24.3 ZNF genes and their mammalian orthologs**. Extended phylogenetic trees of the human 8q24.3 ZNF genes and their mammalian orthologs that were constructed using cDNA, whole protein, zinc finger region and KRAB domain sequences. Click here for file

Additional file 5 **Calculations of synonymous and non-synonymous substitution rates**. Table containing the values from the domain-wise calculations of synonymous and nonsynonymous substitution rates within an ortholog group of each 8q24.3 ZNF gene (done with the YN00 module of the PAML software package version 3.14 [106,54]). Click here for file

Additional file 6 **Zinc finger DNA-binding region comparison**. "Choo & Klug plot" for the C2H2 zinc finger regions of the human 8q24.3 ZNF genes and all their orthologs. Zinc finger region alignment of the proteins in each ortholog group based on individual zinc fingers represented by residues -1, 3 and 6 that are thought to be essential in determining DNA binding specificity. Click here for file

Additional file 7 **Principal component analysis of individual zinc finger peptide sequences for functional and evolutionary conservation**. List of the individual sequences and their values based on the alignment of the 8-amino acid region from positions -2 to 6 with respect to the start of the α-helix of a C2H2 zinc finger that is known to be involved in determining DNA binding specificity. Only human, mouse and rat orthologs were considered. Click here for file

Additional file 8 **Tissue expression profiling**. Tables with primary data and derived relative expression values of ZNF quantitative RT-PCR and details on the gene expression assays and samples. Click here for file

Additional file 9 **Tissue expression distances inferred from ZNF gene expression**. Euclidian distance between 27 human tissues based on the expression analysis of 17 human ZNF genes (7 genes from 8q24.3, 10 from other loci). Click here for file

Additional file 10 **Gene expression similarities based on tissue expression profiles**. Pairwise distances between all genes based on their tissue expression profiles. Included are the seven 8q24.3 ZNF genes, ten ZNF genes from other loci, TRIM28 and twelve non-ZNF genes. Calculation: Distance = 1 - abs (Pearson correlation coefficient). Click here for file

Additional file 11 **Proximal promoter sequences of the seven human 8q24.3 locus ZNF genes**. Sequences of the proximal promoter regions of the seven human 8q24.3 ZNF genes extracted by Genomatix Gene2Promoter. Click here for file

Additional file 12 **UCSC Genome Browser visualizations of the proximal promoter regions of the seven human 8q24.3 ZNF genes**. Visualization along with associated features using the UCSC Genome Browser (human genome hg18). Genome Browser tracks included CpG islands, occupancy with RNA polymerase II core enzyme (Pol2) in four cell lines, occupancy with pre-initiation complex general transcription factor TAF1 and third party TSS predictions. Arrowheads in the depiction of the promoter regions indicate the direction of transcription. Click here for file

Additional file 13 **Core promoter elements in the proximal promoter regions of the seven human 8q24.3 genes**. Detailed listing and sequences of core promoter elements based on Genomatix MatInspector software. Click here for file

Additional file 14 **TFBS modules occuring in at least three of the seven promoter regions of the human 8q24.3 ZNF genes**. Listing of the module families, the modules, their associated individual TFBS and the respective nucleotide sequences. Click here for file

Additional file 15 **Graphical view of TFBS and TSS within the proximal promoter regions of the seven human 8q24.3 ZNF genes**. Visualization of core promoter elements and those TFBS that are derived from module families that occur in at least three promoters. The symbols representing core promoter elements have ovoid shapes, those depicting TFBS from modules are rectangles. Strand orientation is reflected in the placement of the symbols above ("sense" strand) or below ("antisense" strand) the promoter DNA depiction. The width of the symbols reflects the length of the respective DNA elements. Click here for file
