# Supplementary material for: The ancient mammalian KRAB zinc finger gene cluster on human chromosome 8q24.3 illustrates principles of C2H2 zinc finger evolution associated with unique expression profiles in human tissues
Source: BMC Genomics. 2010 Mar 26;11:206. doi: 10.1186/1471-2164-11-206 (PMC2865497; doi:10.1186/1471-2164-11-206)
Supplement: Additional file 1 — Nucleotide sequences. Compendium of all nucleotide sequences used for analysis. [file 1471-2164-11-206-S1.RTF]

Additional file 1									

Note: The list contains the sequences that were used for the analyses and relate to the gene models defined in this publication. Given accession numbers state respective database entries corresponding to these sequences but not necessarily matching exactly to them. For example, the information of several transcripts/cDNA sequences might be combined. Sequences manually curated from automatic gene predictions in GenBank (i.e. accession numbers starting with XM) were given this accession number with the added label “alt” for “altered”. Sequences without a given accession number were derived from genomic sequences using the tools described in Methods.
Species assignment: The human sequences are given with the gene names only whereas the names of the other species are preceded by abbreviations: Chimpanzee (pt, Pan troglodytes), rhesus monkey (mmul, Macaca mulatta and mfas, Macaca fascicularis), dog (cf, Canis familiaris), cow (bt, Bos taurus), mouse (mm, Mus musculus), rat (rn, Rattus norvegicus) and opossum (mondom, Monodelphis domestica).


cDNA sequences of human 8q24.3 ZNF genes:

>ZNF251 [RefSeq:NM_138367]
gaggcgtactgaggccccgcagggggcggggaagcctgcgtgtgcgcagctcggcccggcccgccctcttcatcctagcccgccccctccccggctctggacccggttctgtgggagggtccgttccgggctcggtgcgactgcgcagctcctcggcgcttcctcggtggcttccccgggtcgagcaaacaggtaagttcggggagacttcaggccagggcagtctcctgtcttctcttggaaaccacagcagcccatgcacacagaatggcagccacattccagcttccagggcaccaggagatgccgctgaccttccaggatgtggccgtgtacttctctcaggcggaggggcggcagctgggcccccagcagcgggcgctctaccgggatgtgatgctggagaactatgggaacgtggcctctctgggattccctgtccctaagccggagttgatctcccagctggagcaggggaaggaactttgggtcctgaatcttctgggagctgaggaaccagatatcttgaaaagctgccagaaagattctgaggttgggaccaagaaggaactatctattttaaaccaaaaattttccgaagaagtaaaaaccccagaatttgtatcaagaagactcttaagggataatgcacaggccgctgagtttcgggaagcatggggccgtgagggcaaactcaaagagcgcgtgggaaattctgccgggcagagtttgaacaaacccaatattcacaagagagttttaacagaagctaccgtgggcagggaaagatctttgggagaaagaacccaagagtgtagtgcatttgatagaaacttgaatctggaccaaaatgttgttagacttcaaagaaataaaacaggagagagggtctttaaatgtgatatatgcagcaaaaccttcaaatataattcagacctaagtagacaccagagaagtcacactggggagaagccgtacgaatgtggccggtgtgggcgagcctttactcacagctcaaatcttgttctgcaccatcacattcacactggaaataaaccatttaaatgtgatgaatgtgggaaaacttttggactcaattctcacctccgtcttcatcggagaattcacactggagaaaaaccctttggctgtggtgagtgtgggaaggctttcagtcgaagctcaactcttattcaacatcggatcattcacacaggagagaaaccctacaagtgtaatgaatgtggaagaggctttagccagagcccccagttaactcagcatcagagaattcacactggagagaagccgcatgaatgcagtcactgtgggaaggccttcagtcgaagctccagccttattcagcatgagagaattcacactggagagaagccccataaatgcaatcagtgtgggaaggccttcagtcagagctcaagccttttcctccatcatcgggttcatactggagagaaaccctatgtatgtaatgaatgcggcagagcctttggttttaactctcatcttactgaacacgtaaggattcacacaggagaaaaaccctatgtttgtaatgagtgcggcaaagcctttcgtcggagttccactcttgttcagcatcgaagagttcacactggggagaagccctaccagtgcgttgaatgtgggaaagctttcagccagagctcccagctcaccctacatcagcgagttcacactggagagaagccctatgactgtggtgactgtgggaaggccttcagccggaggtcaaccctcattcagcatcagaaagttcacagcggagagactcgtaagtgcagaaaacatggtccagcctttgttcatggctccagcctcacagcagatggacagattcccactggagagaagcacggcagagcctttaaccatggtgcaaatctcattctgcgctggacagttcacactggtgagaaatcctttggatgtaatgaatatggaaaagctttcagtcccacctcacgacccactgaagatcagataatgcatgctggggaaaagccctataaatgtcaagaatgtggaaacgccttcagtggaaagtcaacccttattcaacatcaggtaactcacactggtcagaaaccatgtcattgcagtgtgtatgggaaagccttcagccagagttcacagctcacaccacctcagcagactcgtgttggagagaaacctgctttaaatgatggctctaaaagatactttattcatatcaagaagattttccaagaaagacatttttaatgtgataaatgcagaagacagtttagcaactgttcacttgacattagaagataagatggcataatgaaagatatataaggtctaaatattactggcaaagtaaaataaatagttcagatgactactaaagtcaaagtcattaaatctggaagtaaacacgagaattcattctgggaaatcaggctgtctgtgtaaaggttactgctgtgctcaggagttgaaccgtgtggtgctgtatccggatactcatgacgaatggatggagggcgtgaaaagtgagcccagctggtgctctgggtctaccctacctgacatccttccagtcttatcctttgtttcctatccaggcccaggcttgtggctgagaacatccactttcagtcccatatacctgcctccaagtgtggtacagagaacttgggcctgctgggggcgcttagccttactctctccaccacctctcccaccaacccccagatgaactgcaggtagacgtttcttccttgcttggagccccagtttttgcatttcattttcattaaaatgaaaggtggtttggttttggttctaaggagctctacagtttaacagagagagggaccttaggggccaagaaagcaggggcctaccaagtatctccacttttgaaaattgtaattactgataaaaaattttttaaaggcaggtttattgaagcataatttataaacaataaaatttactctttttagtgta

>ZNF34 [RefSeq:NM_030580]
attggcggggagggagggccgaacgggcgaggttgggctgccgtgctgctcggcggcgctgaggccaaatagttgcatcacatgtatctaatccgagagtctcatgcttctggtagctcctcagtgaccagctcctgctcactgccctcagaaagcccaaccctcaggcaatggcggccttgttcctgtctgccccaccccaggccgaggtgaccttcgaggacgtggctgtgtacctctcccgggaggaatggggccgcctgggccctgctcagaggggcctctacagggacgtgatgctggagacctacgggaacctagtctcactgggagtaggacctgcaggccccaagcctggagtgatctcgcagttggagcgaggggatgagccctgggtcctggatgttcagggcacctctgggaaagagcacctgagagtcaacagcccagctcttgggaccagaactgagtacaaggagttgacttcacaggagacatttggtgaggaagatccccagggatctgagccagtagaagcctgtgaccacatcagtaagtcagaggggagcctggaaaagctagtggagcagagaggccccagggcagtcacactgaccaacggggagagcagcagggagtctgggggaaacctcaggttgctgtcaagacctgttcctgatcagagacctcacaaatgtgatatatgtgagcaaagttttgaacagagatcatatctcaacaaccataagcgtgtacacaggtcaaaaaaaacaaatacagttcgtaactctggggaaatcttcagtgcaaacttagttgttaaagaagatcagaaaattcctactgggaaaaaattgcattattgcagttactgtgggaaaacattcaggtacagtgccaaccttgtcaagcatcagcggcttcacactgaagagaagccctacaaatgtgatgagtgtgggaaagccttcagccagagctgcgagttcatcaatcaccgaaggatgcactcaggagagattccctaccggtgtgacgagtgtgggaagacattcacccggaggcccaacctcatgaagcaccagaggattcacactggggagaaaccctacaagtgtggggagtgtgggaagcactttagcgcctactcttccctgatttatcaccagagaatccacaccggagagaaaccctataaatgtaatgactgcgggaaagccttcagtgatggctcaatccttatccgacatcgtcggactcacaccggagagaagccatttgagtgcaaggaatgtggcaaaggctttacacaaagttctaaccttatccaacatcagagaattcacactggagagaaaccctataaatgtaatgaatgtgagaaagctttcattcaaaaaaccaaactcgtggaacatcagagaagccacactggagagaagccctatgaatgcaatgactgtggcaaagttttcagccaaagcacacacctcatccagcaccagagaatccacacaggagagaagccctacaagtgcagcgagtgtgggaaggccttccacaacagttccagactcatccaccaccagaggctgcaccacggagagaaaccctacagatgcagcgattgcaagaaagccttcagccagagcacgtacttgattcagcaccggaggatccacaccggggagaagccctacaagtgcagcgagtgtgggaaggccttccggcacagttccaacatgtgtcagcatcagcggattcacctccgggaggacttctccatgtaacagtggcgcggtgtccgagggcagagtccagctgagcacttcctgcatgcgcccccggcacctgactctgccctttatgtattatccacacgatgttttcacagagtgaaaggacgtttctcattaaacaaacctcttttcttaaatcaaaagcagtgcatgttcattttagagaaattgagacagaaaagtgggcaaaaaagaagcttctgtgatctctcccacttagaaaaaaaattcattgctgctgatttacgaatattttcgagacatactggagagcattttaaaaagaaaataccgtaatgcttgtcattgcagcacctaggatcttcacttttgctatttcctcaatatcagaaaattcacactgaagagagcaccatatattttaacatttttacatattgtaaatttaaaagcatcggccaggtgtggtggctcatgcctgtaataccagcattttgagaggccaaggtgggtggatcacttgaggtcaggagtttgagaccagcctggccaacatggtgaaaccccgtctctactaaaaatacaaaaattagccaggtgtggtggtgggtgcctgtaatcccagctactcgggaggctgaagcaggagcattgcttgaacctgggaggcggaggttgcagtgagccaagactgtgccactgtactccagcctgggtgacagagcgagacgctgtctcaaaaaataaatgaaacaaacttaacattcatactttttatgtcaatagagttgaagttgcctttaactgtggtgaaataacataatatgaaatttacaattttaaccatttttaagtgtacaattcagtggcattaagtacattcatggtgttgtgtaaccatcatcactgtgtatttccagaactgttttattactccaaacagaaaccctgtacctattaaataataactcttcatttcccccttctccctactgctggtaacctcaattctattttctgtctctttgaatttgcctattctaggaacataaatatgtgtggaatcatgc

>ZNF517 [RefSeq:NM_213605]
ggaaccggagcctgagagccgggcgccGTGCGCTCCTCCCCGCGCTGTCTCGGCGGCCCAGGAATTCACTGTCTGTAGCATCTGCTCCTCCACAGAGGGACCCTGGAATGGCGATGGCACTCCCGATGCCTGGACCTCAGGAGGCGGTTGTGTTCGAGGATGTGGCTGTGTACTTCACAAGGATAGAGTGGAGTTGCCTGGCCCCCGACCAGCAGGCACTCTACAGGGACGTGATGCTGGAGAACTATGGGAACCTGGCCTCACTAGGCTTTCTTGTTGCCAAACCAGCACTGATCTCCCTATTGGAGCAAGGAGAGGAGCCGGGGGCCTTGATTCTGCAGGTGGCTGAACAGAGCGTGGCCAAAGCCAGCCTGTGCACAGATTCCAGGATGGAGGCTGGGATCATGGAGTCTCCTCTGCAGAGAAAGCTCTCCAGGCAGGCAGGACTGCCGGGCACCGTGTGGGGGTGCCTCCCCTGGGGGCACCCTGTGGGGGGGCACCCTGCACCACCCCACCCGCATGGCGGTCCTGAGGACGGGTCAGATAAACCCACCCACCCCCGGGCTCGGGAGCACAGCGCCTCCCCAAGGGTTCTGCAGGAAGACCTGGGCCGGCCTGTGGGGAGCTCAGCCCCCCGCTACAGGTGCGTGTGCGGCAAGGCGTTCAGATACAACTCGCTGCTTCTCAGGCACCAGATCGTCCACACCGGCGCCAAGCCCTTCCAGTGCACAGAGTGCGGGAAGGCCTTCAAGCAAAGCTCCATCCTGCTGCGGCACCAGCTGATCCACACTGAGGAGAAGCCGTTCCAGTGCGGCGAGTGCGGGAAGGCCTTCCGGCAGAGCACGCAGCTGGCTGCCCACCACCGCGTCCACACCCGCGAGCGGCCCTACGCATGCGGCGAGTGCGGCAAGGCCTTCAGCCGCAGCTCCCGGCTGCTGCAGCACCAGAAGTTCCACACCGGGGAGAAGCCCTTCGCGTGCACAGAGTGCGGCAAGGCGTTCTGCCGCAGGTTCACCCTCAACGAGCACGGCCGCATCCACAGCGGGGAGCGGCCCTACCGGTGCCTGCGGTGTGGGCAGCGCTTCATCCGAGGGTCCTCGCTCCTGAAGCACCACCGGCTGCACGCGCAGGAGGGTGCCCAGGACGGCGGCGCGGGGCAGGGCGCCCTGCTCGGAGCTGCGCAGAGGCCCCAGGCGGGGGACCCGCCCCACGAGTGCCCGGTGTGCGGGAGGCCGTTCCGACACAACTCCCTGCTGCTGCTGCACCTGCGCCTACACACGGGCGAGAAGCCGTTCGAGTGCGCGGAGTGCGGCAAGGCCTTCGGTCGCAAGTCCAACCTCACTCTGCACCAGAAGATCCACACCAAGGAGAAGCCCTTCGCGTGCACCGAGTGCGGCAAGGCGTTCCGCAGGAGCTACACGCTGAACGAGCACTACCGGCTCCACAGCGGCGAGAGGCCATACCGGTGCCGCGCCTGCGGGAGGGCCTGCAGCCGGCTGTCCACCCTCATCCAGCACCAGAAGGTGCACGGCCGCGAGCCCGGGGAGGACACAGAGGGCAGGCGGGCGCCCTGTTGGGCTTCCTGATGACGGGGACGACAGGCCGAGGATTCACGCTGGAAGCCCACCCAAGCCGGCGGGGCCCTAGCGCAGAAATTCAGAACCCCCTGTCCTGAAGGATCTCTCTGTCAGCCAGCCTGGAGTGCAATGGCGCAATCATGGCTTACTGCAGCCTCCAGCTCCCAGGCTCAAGCGTTTCTCCCACCTTAGCCTCCTGAGTAGCTGGGACTAAGGCACACACACTGTACCTGGCAGCTGGCGAAGCATTGTTTCTGAGTGTGTGAGGATGTTTTCAGTGGACTCAGTGAAGATCTGCCCTCAGCGTGGGCAGGCACATCCACTTGGTTGGGGACCCAGATGGAACACAAAGGGAGAAGACAAGCAAATTCTCTTTTCTGGAGGGAGACACCCATCTCCTGCCCTTGGACATCAGGACTCCAGGTTCTTCGGCCTTTGGACTCAGGCTTGCCACAGAGGCCTCCCAGGGCTCTCGGCCAGTCAGCCTCAGAATGAGAGTTACACCACTGGCTTCCTTGGTTCAACCACCTTCTTACCTGGACTGAGCCTCACTTACAGCTTCTCTAGGTCTCCAGCTTGCAGACAGCCTATGGGAGGACTTCTCAGCCTCCATAAGTGTGTGGGCCAGTTCGCCTAATAAATCCCCTCTCCTGGCCGGGCGCGGTAGCTCTCCCCTGTAATCTCAGCATTTTGGGAGGCAGAGGTAGGTGGATCACCTGAGGTCAGGAGTTCAAGACCAGCCTGGCCAACATGGTGAGACCCCCGTCTCTACTAAAAGTACAAAAAGTAGCTGGGTGTGGTGCTGGGTGCCTGTAATCCCAGCTACTCGGGAGGCTGAAGCAGGAGAATCACTTCGACCTGGGAGGTAGAGGTTGCAGTGAGCCGAGATCGAGCCACTGCACTCCAGCCTGGGTGACAGGGCAAGACTCTGTCTCAAACAAATAAAAATCCCCTCTCCT


>ZNF7 [RefSeq:NM_003416]
GGCGCGGACTCGGGTTGCCCTCGGTCCGAGTGATCCCTGGTCGCTTCCTTAGCCCTCCCGCCTTCGGCATTGGGGTCCCCGCGTCCCCCGGGCCTCCAGGCGGGAAAGCGCGGGGGCTTTGCGGGGCCTTGAGCGCCTGGTGTGGGAGGTGGTCGAGCCCAGCCACCCTCCCCCGCGGCGGCGCGAGGTCTCTCGGCCAGAACACGTGGATGCCCACCCACCACTGAGCCTCATGGAGGTGGTAACATTTGGCGATGTGGCTGTGCACTTCTCTCGGGAGGAGTGGCAGTGTCTGGACCCTGGCCAGAGGGCCCTCTACAGGGAAGTGATGCTGGAGAACCACAGCAGTGTGGCTGGACTAGCAGGATTCCTGGTTTTCAAGCCTGAGCTGATCTCTCGGCTGGAGCAGGGAGAAGAGCCATGGGTCCTCGACCTGCAGGGAGCAGAGGGGACAGAGGCACCAAGGACCTCCAAGACAGATTCTACGATTAGGACTGAAAATGAGCAGGCCTGTGAGGACATGGACATCCTAAAATCAGAATCCTATGGGACAGTGGTCAGAATCTCCCCACAGGACTTTCCTCAGAATCCTGGCTTTGGAGACGTTTCTGATTCTGAGGTCTGGTTAGACAGTCATCTGGGCAGTCCCGGGCTGAAAGTGACAGGCTTTACCTTCCAAAATAACTGTTTGAATGAGGAGACTGTGGTTCCCAAGACCTTCACCAAGGACGCACCCCAGGGATGTAAGGAGCTGGGAAGCAGCGGCCTGGATTGTCAGCCTCTTGAAAGTCAGGGAGAGAGTGCGGAAGGGATGTCCCAGAGATGCGAGGAGTGTGGCAAAGGCATCAGAGCCACTTCAGATATCGCTCTGCATTGGGAAATTAATACACAGAAAATTAGCAGATGTCAAGAATGCCAAAAAAAGTTATCTGACTGCTTGCAGGGGAAACATACAAATAACTGCCATGGAGAGAAGCCGTACGAATGTGCAGAGTGTGGGAAAGTCTTCAGGCTCTGCTCGCAGCTTAATCAGCATCAGAGAATCCACACGGGAGAGAAACCCTTTAAATGCACTGAGTGTGGAAAAGCCTTCCGCCTGAGCTCAAAACTTATTCAGCATCAAAGAATCCACACTGGGGAGAAGCCCTACAGATGTGAGGAATGTGGAAAAGCTTTTGGTCAGAGCTCAAGCCTCATCCACCATCAGAGAATCCACACAGGAGAGAGGCCCTATGGTTGTCGTGAGTGTGGGAAAGCCTTCAGCCAGCAGTCGCAGCTGGTTAGACACCAGAGAACTCACACTGGGGAGAGGCCCTACCCTTGCAAGGAGTGTGGGAAGGCCTTCAGCCAGAGCTCCACCCTAGCCCAGCATCAAAGGATGCATACTGGGGAGAAAGCTCAAATTCTAAAAGCCTCAGACAGTCCAAGCCTTGTTGCACATCAGAGAATTCACGCTGTAGAGAAACCATTTAAGTGTGATGAGTGTGGGAAAGCTTTTAGGTGGATCTCTCGCCTGAGTCAGCATCAGCTGATTCACACTGGAGAGAAGCCTTATAAATGCAACAAGTGTACAAAAGCCTTTGGTTGTAGTTCACGGCTTATTCGCCATCAGAGAACTCACACTGGAGAAAAACCATTTAAATGTGATGAGTGTGGCAAAGGCTTTGTTCAGGGCTCACACCTTATTCAGCATCAGCGAATCCACACTGGAGAGAAACCCTATGTGTGTAATGACTGTGGAAAAGCCTTCAGTCAGAGTTCCAGCCTTATTTACCATCAGAGAATCCATAAAGGAGAGAAGCCCTACGAATGCCTCCAATGCGGAAAAGCCTTCAGTATGAGCACACAGCTTACAATACATCAAAGGGTTCACACTGGAGAGAGGCCCTATAAATGTAATGAATGTGGGAAAGCCTTCAGTCAAAACTCAACCCTTTTCCAACACCAGATAATTCATGCAGGGGTGAAGCCCTATGAGTGCAGTGAGTGTGGAAAAGCCTTCAGCCGGAGCTCATATCTTATTGAACACCAGAGAATACACACTAGGGCCCAGTGGTTTTACGAATATGGGAATGCCCTGGAAGGGTCCACCTTTGTGAGCCGTAAAAAGGTTAATACTATAAAGAAACTGCATCAGTGTGAAGACTGTGAGAAGATATTTAGGTGGCGTTCACACCTAATTATACACCAGAGAATTCACACCGGGGAGAAGCCTTATAAATGCAATGACTGTGGCAAAGCTTTTAATCGTAGCTCAAGGCTTACCCAGCATCAAAAAATTCACATGGGATAGACCACTTACATATAAATGTGTATATATGTGAATAAACCTATAGCCTTAACTT


>ZNF250 [RefSeq:NM_021061]
agccctttgtctgaaggtgctgcgggatgccgttccttcgcgcgtgaggctgcggctctgacgccccataggctccttcaatttccgtgatcctcggagtccccaggagaccaggtgATGGCAGCAGCCAGACTCCTGCCAGTGCCGGCAGGACCCCAGCCCCTGTCGTTCCAGGCCAAGCTGACCTTCGAGGATGTGGCTGTGCTCCTCTCCCAGGATGAATGGGACCGCCTGTGCCCTGCTCAGAGGGGTCTCTACAGAAATGTGATGATGGAAACCTATGGGAATGTAGTCTCATTGGGACTTCCAGGATCCAAGCCTGACATAATCTCCCAGCTGGAGCGAGGGGAAGATCCCTGGGTCCTGGACAGGAAGGGGGCTAAGAAGAGCCAGGGCCTGTGGAGTGACTACTCAGACAACCTCAAATATGACCACACTACAGCCTGTACACAACAAGACAGTTTATCTTGTCCATGGGAATGTGAAACCAAGGGAGAGAGTCAAAATACAGACTTGAGTCCGAAGCCATTAATTTCAGAGCAAACAGTGATTCTGGGGAAAACACCCTTGGGGAGGATTGATCAAGAAAATAATGAAACAAAGCAAAGCTTCTGTCTGAGTCCAAACTCTGTTGACCACCGTGAAGTTCAGGTCTTAAGCCAAAGCATGCCACTCACTCCGCACCAGGCAGTGCCTAGTGGAGAGAGGCCCTACATGTGTGTTGAGTGTGGGAAGTGCTTTGGCCGGAGTTCCCACCTCCTTCAGCATCAGCGTATCCACACTGGAGAGAAGCCCTATGTGTGCAGTGTATGTGGGAAGGCCTTCAGCCAGAGCTCAGTCCTTAGTAAACACAGGAGAATTCACACAGGTGAGAAGCCCTATGAGTGTAATGAGTGTGGAAAAGCCTTTAGAGTGAGCTCAGATCTTGCTCAGCATCACAAGATACATACAGGAGAGAAGCCTCACGAATGTCTTGAGTGTCGGAAAGCCTTCACTCAACTCTCACATCTCATTCAGCACCAGCGGATCCACACGGGAGAAAGGCCATATGTGTGTCCGTTGTGTGGGAAAGCCTTCAACCATAGCACTGTTCTGCGGAGCCACCAGAGGGTACACACTGGGGAGAAGCCTCACAGGTGCAATGAGTGTGGGAAAACCTTCAGTGTGAAGAGGACACTGCTGCAGCACCAGAGGATCCACACCGGGGAGAAGCCCTACACGTGCAGCGAGTGTGGGAAGGCCTTCAGCGACCGCTCAGTCCTCATTCAGCACCACAACGTGCACACCGGGGAGAAGCCCTATGAGTGCAGTGAGTGTGGGAAGACCTTCAGCCACCGCTCCACACTGATGAATCACGAGCGGATCCACACCGAGGAAAAGCCCTATGCATGCTACGAATGTGGGAAGGCCTTCGTTCAGCACTCACACCTGATCCAGCACCAGAGAGTCCACACTGGGGAGAAGCCCTATGTGTGTGGTGAATGTGGGCACGCCTTCAGTGCACGCCGGTCTCTGATCCAGCATGAGAGAATCCACACAGGTGAAAAGCCCTTCCAGTGCACAGAATGTGGCAAAGCCTTCAGCCTGAAAGCAACTCTGATTGTGCACCTGAGGACCCACACGGGCGAGAAGCCATATGAGTGCAATAGCTGCGGGAAGGCCTTCAGCCAGTACTCAGTGCTCATCCAGCACCAGCGGATCCACACAGGCGAGAAGCCCTATGAGTGCGGGGAGTGTGGGCGTGCCTTCAACCAGCATGGCCACCTAATCCAGCACCAGAAAGTGCACAGAAAGTTGTGAcccatggctgacacaagaatccattctcacagaaactgcatgtggaaccacaagcagccttcagcccaagagaagtctctgttaactctataggaagcttttctttggcgattcagtgtcacaaaataactccagaaagaagcacttagcgtgctgttcctgtggaaaaacttcagagactacctgttttattttcctcaacatcttgaagttatgttggagagtaatcatacaattgtagagaattttggtaaaaaacagccataattctttaacattagtttatttgaactaagggaatttaaggcataagaaccattatcccaataaaatcttacattccaaataaagttctttttctaagaaca


>ZNF16 [RefSeq:NM_006958]
AATTCGGGGCGGGACTTCCGGGGGTCAGCCGGCGTTGGCTGAGACGTCTTCGTGCCACGGTGCTGCCTCCTTTCCAAGCGCGACCCGTTGAGGTCCTTGTCATGCCCAGCCTCAGAACTCGCCGTGAGGAGGCAGAGATGGAGCTCTCAGTTCCAGGACCATCCCCCTGGACCCCTGCAGCCCAGGCCCGTGTGAGAGATGCTCCTGCTGTGACCCACCCTGGATCTGCAGCCTGTGGTACCCCCTGCTGTAGTGATACTGAGCTGGAAGCCATCTGCCCTCACTATCAGCAGCCAGATTGTGACACCAGGACTGAAGACAAGGAGTTTCTTCACAAGGAAGACATTCATGAAGATTTGGAATCACAGGCAGAAATATCAGAAAACTATGCTGGTGATGTTTCCCAGGTACCCGAGCTTGGAGATCTGTGTGATGATGTATCAGAAAGAGACTGGGGAGTCCCCGAAGGCAGGAGGCTGCCACAGTCCCTCTCCCAGGAGGGGGACTTCACACCAGCTGCCATGGGGCTCCTTAGGGGCCCCTTAGGGGAGAAAGATCTGGACTGTAATGGTTTTGACAGTCGCTTCAGTCTGAGCCCAAACCTGATGGCATGTCAGGAAATCCCTACAGAAGAGAGGCCACATCCATATGACATGGGTGGCCAGAGTTTCCAGCACAGTGTGGACCTAACTGGTCATGAGGGGGTTCCCACAGCTGAAAGTCCACTCATATGTAATGAGTGTGGGAAAACCTTCCAAGGAAATCCTGACCTTATTCAGCGTCAAATAGTCCACACTGGGGAGGCTTCCTTTATGTGTGATGATTGTGGGAAAACCTTCAGCCAGAACTCAGTTCTTAAAAACCGTCATCGATCTCATATGAGTGAGAAAGCTTACCAGTGCAGCGAATGTGGGAAAGCCTTCCGAGGGCACTCAGACTTTTCTAGGCATCAGAGTCACCACAGCAGTGAGAGGCCTTATATGTGTAATGAATGTGGAAAAGCCTTCAGCCAGAACTCGAGCCTTAAAAAGCACCAAAAGTCTCACATGAGTGAGAAGCCCTATGAATGCAATGAATGTGGGAAGGCTTTTAGGCGGAGCTCAAACCTCATCCAACATCAAAGAATCCATTCTGGGGAGAAACCGTATGTGTGCAGTGAGTGTGGGAAGGCCTTCAGGCGAAGCTCAAACCTCATCAAACACCACAGGACTCACACAGGAGAGAAGCCTTTTGAGTGTGGCGAGTGTGGGAAAGCCTTCAGCCAGAGTGCACACCTGAGGAAGCACCAGAGGGTCCACACTGGAGAGAAGCCTTATGAGTGTAATGATTGTGGCAAGCCCTTCAGTCGGGTCTCCAACCTCATTAAGCACCACAGGGTTCACACTGGAGAGAAGCCCTATAAGTGCAGTGACTGTGGGAAAGCATTTAGTCAGAGCTCCAGCCTTATTCAGCATCGGAGAATTCACACTGGAGAAAAGCCTCACGTGTGTAATGTATGTGGAAAAGCCTTTAGTTATAGCTCAGTGCTCCGAAAGCACCAGATCATCCACACGGGAGAGAAGCCGTACAGATGCAGTGTCTGTGGGAAGGCCTTCAGCCACAGCTCAGCCCTCATTCAGCACCAGGGCGTGCACACAGGCGACAAGCCCTACGCCTGCCACGAGTGTGGGAAGACCTTTGGTCGCAGCTCCAACCTCATCCTTCACCAGCGAGTCCACACTGGAGAGAAGCCCTATGAATGTACTGAATGTGGAAAAACCTTCAGCCAGAGCTCAACCCTCATTCAGCATCAGAGGATTCATAATGGGCTGAAGCCCCATGAATGTAACCAGTGTGGTAAAGCCTTCAACCGAAGCTCAAATCTCATTCACCACCAGAAAGTTCATACTGGGGAAAAACCCTACACCTGTGTTGAATGTGGTAAGGGCTTCAGCCAGAGCTCACACCTCATTCAGCATCAGATAATCCACACGGGCGAGCGCCCCTACAAATGCAGTGAGTGTGGGAAAGCCTTCAGTCAGCGTTCGGTCCTCATCCAGCACCAGAGGATTCACACTGGGGTGAAGCCCTATGACTGTGCTGCTTGTGGGAAAGCCTTCAGCCAGCGATCAAAGTTGATCAAACACCAGTTGATTCACACCAGGGAATAGGCTGTTGGGCTGGCAGGAGTGAAACCGAGCATAGTTTCCTCTCCAACTCCTCTTTGGCCATTGTCTCAGCCTCTGCCACTTCCCAGACCGAAAGCCTGGACTCAGCTCACTGCCTACATCCAGTGGCCACATAGCCTGGGACCCTCCTTTGAAACATCCTTTACAGGCTCCCTTCTCACTGCCACTGCTCTTGCCTCTTGTGATGTCTTGGGTTTGGGCTTGTTTTTACAGCTTGTGAACGGACTGCACGTTTGTTACAAGGGAGAAGATTATGGAGGCTGCATATTCATGAATAGAGTTTATTATTATAATGAAAAGTTACCAAAAAAGTAAAGTGACGTTTGGTTTGAG


>ZNF252exon2 [GenBank:BC016287]
ggtatctgccctgtttccttgctctcaaaacacacttcaaacatgtcataatcttcagtggtaaaggcaaatttccccttggttgtattctttttggagtagaaaatatggccagcagaatctgtgatcttgaggtggctgcacaggccaccagtgcccccagactggtcagagatctcgtatgtgccagtcactagtaggtccttgtggatctcctcatggaagcagttgtgagaattaatggacagatggaaggagatggcgaggaccaagctggggcccagcaggaacaaaagcagcaacgccgatggacaagggccatgccgggctggtgggccagacaaaccagacatggtgctggagactcattccccctttagcccttctgctggggatcaacacccactactatgagtcctctaccatgatgtgttcaggccccaacactgtcctatctatgctccccgtcctcctgaaaagctagttttgacctttcctcagcttaggcagaggcctgtgtctttctccacggttagagaccaggtggcagggaaggacacaaccagcacaaagactcagaccacaggtccatcctgccctctacctgggatgacttttcaggtcttgcttcctttctctggagcagGACTTCAAGCTTCCAAACCTGATGTCATCTCCAGGCTGGAGCGGGGGGACGAACCATAGACCCCTCACATCCTGAGAACTCAGGGGAGCTGGAGCTGGAGGCACAAGAGAGAAGGCTGTGATTCTAGGATTGAAAAAGAGGAAATGATTCCAAAGCAGGACATTTCTGAAGAATTGGAATCCCAGAGAGCAAAATCAGAAGATCATGTAAGGAATATTTTTAAGGAAACTGAAGAGATGAGTAAAACTGAGGGAAAGTTAGAGAATTGCTGGAGAAAATATGCAGTAGAAGGAGTTAAGAACTCATTCTCCCAGAAGAGCAATTTCAGAGAAATTACCATGAGGTATGTGAAAACCCTCTCTAGAGAGAATGGCCAGAAGTTCAATGCTGTTGGAGAAAACTGCATTACAGACTCAAATCCTGCCAAACATCTTAGAGGGTCTAGAGAGGAGAGTCTCCATCCAAGTGTGTCAAGTGTAGAAAACTTGCAACAACATGAGGACCTCATTAACCTCCAGAGTTTCCAATTAGGAGAAAGAGCCTATCAGACAGATGTGTTAGTGAAAGTGCCCAGACAGAGCTCAGTTCTTAGTGAGAATCAGAGGATGAATAATCCAGAGAGATGGTTTGAGAGTACTGGGTGTGGAAAAACTTACAATCAGAACAGAGCTTTTAACCAGCACCAGAGATTTCATAGTGGAGAGAAGACCTATGAGCACAATGAATGTGGAAAAGCTTTCAGTTGGCCCTCTATCCTCAGTAAACATCAGAGAATCCACACTGGTAAGAAACTCTACACATGTGAGGATTGTGGCAAATCTTTCAGTGTTCACTCATACTTTATTCAGCATTGTAAAATTCACACTAGAGAAAAACCCTATGAGTGTATTAAATGTGGGAAAGCTTTTAGTACACATTCATCTTATGTTCAACATCTAAAAATTCATACAGGAGAGAAACATCATGAGTGTAATCAGTGTGGGAAAGCCTTTAGTCATAGCTCTAATCTAATTCATCATCAGAGAATTCATAGTGGAGAGAAACCTTACAAGTGCAAAGAATGTGGGAAAGCCTTCAACAGACAATCAAACCTTATTCAGCATCAGAGAATTCATTCTGGAGAGAAACCTTATGACTGTAAGGAGTGTGGCAAAGCCTTCAGTACACAATTATTTCTCATTCAGCATCAGAGAATTCATACAGGAGAAAAGCCCTATGAATGTAATGAATGTGCAAAATCCTTTAGCCTGAACCGAACTCTTACTGTCCATCAGAGAATTCACACTGGAGAGAAACCTTATAGGTGTAATGAATGTGGGAAATCCTTTAGTCAATGCTCACAAGTTATTCAACATAAGAGAATTCACACTGGAGAGAAACCTTATATCTGCAATGAGTGTGGAAAATCATTTGGTGCTCGTCTATCCCTTATCCAGCATCAGAGAATTCACACTGGAGAGAAACCTTATGGTTGTAGTGTGTGTGGGAAAACCTTTAGTCAAAAGGGACATCTTATTCAGCATCAGTGAattcacacaggagagaaaccctatgaatgtagtgagtgtggaaaagctttcagccagagttttaatcttattcaccatcaaagaacacacaatggtgagaagtcctatgaatgtaatgaatgtgataaagccttcagtttgctttcttcccttgttcaacatcagagaatacataatggagacaaaccctatgagtgtcacaaatgtgggaaggcctttagccaggggtcacaccttattcagcatcagaggagtcacattggtgagaaaccctatgagtgtaatgagtgtgggaaaacctttgggcagatatccaccctaattaagcatgagagaacacacaatggagagaagccctatgagtgcagtgactgtgggaaggccttcagccagagtgcacaccttatccaccatcaaagaattcacactggagagaatccctatgagtgcagtgaatgtgggaaggccttcaatgtttgttcctctctcattcagcatcacagaattcatactggtgagaaaccttatgaatgtagtgactgtggcaaggcgttcagtcagcattcacaatttatccaacatcagagaattcacactggagagaaaccctacatgtgcaatgagtgtgagaaatccttcagtgcatgcttatcccttatccaacacaagagaattcacactggagagaaaccctatgtatgtgccaaatgtggaaaatccttctgacaaagctctcaccttattcaacatcagagaattcacagtggggagcaacctcatacgtgtaatcgatgtgaaaaaaccttcagttagagaataactcttagtagtcatgagaaaatccatacaattcacattagagagcaagtctatgaatgtagtaagtgtggggaactctagcgcacagtcatctttcattcaacattgtacagttcacagtggagagtaactgattagtctataaattcttcacagttatacattatatgagagtcatactcgtgagaaatgagtcactttatggttttccttttggagattcagctcttaagggatttcactctcccagtgagctgctaactaaaggcaggctgctgaagtatatagcagcaatcccacaatagcacaccttgcttcagattcagaggatgagcccccttcttgagccctgtagatatgtgaaacttaattatttagatgcatttcatggcaccagaccctaaggtacagttaggtatttcactggagaccacttactttgctttgctttgcttttttttttttttttttttgtatgatttatttcaacttcaaagaaagtagttgagatccttgcacttccaagtgtgatggaaccaggctagggttttctttgtttgtttgggcctcttcaaaacgtttttattctgttataaggaatatgataccttaagcttcaattcacagggaagagaaacatgattataagcaaaagtttgtcaaggaataaggtgagtctttgtgtagattaaaatagttgagaccttgctaaaaagtcagataacactgtaatccacctccaggtttccaagagttaagacgctgccagcaaggagcttgcagtcttgtagctggcccaatatatccaactaattcaaaatgggaaattgtacattttacttaaacaggatttggtagatgggtcttctagatatgttgtcatgaagaaaaatcaggaaaatgtccaagggtgggagcctgtggcttatggcttcacttacccatcacagatagctatgttacactgcaagaacagagttcatggcttttcctgaggtggctggaaaccttaatctcctgaagttaagtcaaaacttaaatgtcaattcacccagctctcttgcctgcaggctgatccagggacttagagcttgttacccagttacaacttcttctagagaagaacttttaagaaggaagcaaaaagaaattcagtttcccccaaggatctcttctttgaggttccatcaagagatgacaaaaggaggctgggcgcggtgtctcacacctgtaatcccagcactttgggaggctgaagtgggcagatcacgaggtcaagagatcaagaccatcctggccaacatggtgaaaccacatttctacaaaatgcaaaaattagctgggcatggtggtgcacgcctgtagtcccagctacttgggaggctgaggcaggagaattgcttgaacccaggaggcggaggtagcagtgagccaagattgcaccactgcattccagcatggccacaaagcaagactccaaaaaatatatatatatatgacaaaagggttaggaagcttttcctctgaggcccacgattacacaaccaaatcagaaaataatattttttttgttttaaacctgtttacataagatggaggtgtttacttaactaagaacctcggtatttactcaaatccggttttgggttttatggctaaaacatttctccagattaaccttatcagtttggaatatgtttttattttccattaaatatttcatccaaatcatactgaattttatttatcatggcttttaatgttctctattcaagtcaaatttctgtttcctaattacttggcttggtgatggcattaatattggttgggagctagatggtctctgctctatttgatgattggcatagagcaatcatacacttggtacttcctagcatttttttttgagacagtatcatgctgtatcatgttgcacagggtggagtgcagtggtgcagtcatagctcactgtaaccttgaactcctgggctcaagtgattcccctgcctcaacttcatgagtagcagggactacagctgtgtaccaccacaccggcctctcctggcttcttaaccacttacattaaaattgagaggagaaaggcattttcagtttctttagttaataaaaagaagccatttctggaggagttttatgcctgtaccagcagaggttcagccttccaggaatctcatcatgatccatactgctgacacaggcctttgtcacctgaagcattcttaaaataaggagactgacattaaacaggacaattgtgaactccactttgtaagcatcatacatatcttacaactcattctgaagactcctttattcttgctcttcccagagagctactgtgtttagtcatgctcactgcactcacaagagaaaaagagtaagtggcatatttgcaggtgttgatggttacaagagcatttttactttttcctccatgtttgttctgcagcatttccattgatgcatgctattgtagacttcttaggttgaccttggccttgacctccaccactagctcatgacccaaaactgaagatgtgcatcagaaagggacagatttggtctttctgctgtaaaagatattctgccttagtgcctgctaatttgttgtgcatagtcctgtgctgccaatcttccccaccaattgttaaggttaatgtgtgttttatatgtgtattcacaaacattaaaatgtaatcagtggattccattattg


>ZNF252exon5 [GenBank:BC019922]
tccggcccagggggcttggctccccgcggcacgggatttagcgttcgcgctccttcccttcccgtggtcgagccgagtcctgacctgagggctgcatcaagatcttgtcattccacatcgtggtttcctttgaggatgtggctgtacccctctcccaggaggagtgggactgtctgatccctgctcagaggggcctctacaaggatgtgatgatggggacctatgggaacctactctcattagtaggtgaatggttaagcaaactgtggtacatccataccatgggatacgactcaacaatcaaaaggaactgcccagacttcaccacgatgcaatatatgcagacttcaagcttccaaacctgatgtcatctccaggctggagcggggggacgaaccatagacccctcacatcctgagaactcaggggagctggagctggaggcacaagagagaaggctgtgattctaggattgaaaaagaggaaatgattccaaagcaggacatttctgaagaattggaatcccagagagcaaaatcagaagatcatgtaaggaatatttttaaggaaactgaagagatgagtaaaactgagggaaagttagagaattgctggagaaaatatgcagtagaaggagttaagaactcattctcccagaagagcaatttcagagaaattaccatgaggtatgtgaaaaccctctctagagagaatggccagaagttcaatgctgttggagaaaactgcattacagactcaaatcctgccaaacatcttagagggtctagagaggagagtctccatccaagtgtgtcaagtgtagaaaacttgcaacaacatgaggacctcattaacctccagagtttccaattaggagaaagagcctatcagacagatgtgttagtgaaagtgcccagacagagctcagttcttagtgagaatcagaggatgaataatccagagagatggtttgagagtactgggtgtggaaaaacttacaatcagaacagagcttttaaccagcaccagagatttcatagtggagagaagacctatgagcacaatgaatgtggaaaagctttcagttggccctctatcctcagtaaacatcagagaatccacactggtaagaaactctacacatgtgaggattgtggcaaatctttcagtgttcactcatactttattcagcattgtaaaattcacactagagaaaaaccctatgagtgtattaaatgtgggaaagcttttagtacacattcatcttatgttcaacatctaaaaattcatacaggagagaaacatcatgagtgtaatcagtgtgggaaagcctttagtcatagctctaatctaattcatcatcagagaattcatagtggagagaaaccttacaagtgcaaagaatgtgggaaagccttcaacagacaatcaaaccttattcagcatcagagaattcattctggagagaaaccttatgactgtaaggagtgtggcaaagccttcagtacacaattatttctcattcagcatcagagaattcatacaggagaaaagccctatgaatgtaatgaatgtgcaaaatcctttagcctgaaccgaactcttactgtccatcagagaattcacactggagagaaaccttataggtgtaatgaatgtgggaaatcctttagtcaatgctcacaagttattcaacataagagaattcacactggagagaaaccttatatctgcaatgagtgtggaaaatcatttggtgctcgtctatcccttatccagcatcagagaattcacactggagagaaaccttatggttgtagtgtgtgtgggaaaacctttagtcaaaagggacatcttattcagcatcagtgaattcacacaggagagaaaccctatgaatgtagtgagtgtggaaaagctttcagccagagttttaatcttattcaccatcaaagaacacacaatggtgagaagtcctatgaatgtaatgaatgtgataaagccttcagtttgctttcttcccttgttcaacatcagagaatacataatggagacaaaccctatgagtgtcacaaatgtgggaaggcctttagccaggggtcacaccttattcagcatcagaggagtcacattggtgagaaaccctatgagtgtaatgagtgtgggaaaacctttgggcagatatccaccctaattaagcatgagagaacacacaatggagagaagccctatgagtgcagtgactgtgggaaggccttcagccagagtgcacaccttatccaccatcaaagaattcacactggagagaatccctatgagtgcagtgaatgtgggaaggccttcaatgtttgttcctctctcattcagcatcacagaattcatactggtgagaaaccttatgaatgtagtgactgtggcaaggcgttcagtcagcattcacaatttatccaacatcagagaattcacactggagagaaaccctacatgtgcaatgagtgtgagaaatccttcagtgcatgcttatcccttatccaacacaagagaattcacactggagagaaaccctatgtatgtgccaaatgtggaaaatccttctgacaaagctctcaccttattcaacatcagagaattcacagtggggagcaacctcatacgtgtaatcgatgtgaaaaaaccttcagttagagaataactcttagtagtcatgagaaaatccatacaattcacattagagagcaagtctatgaatgtagtaagtgtggggaactctagcgcacagtcatctttcattcaacattgtacagttcacagtggagagtaactgattagtctataaattcttcacagttatacattatatgagagtcatactcgtgagaaatgagtcactttatggttttccttttggagattcagctcttaagggatttcactctcccagtgagctgctaactaaaggcaggctgctgaagtatatagcagcaatcccacaatagcacaccttgcttcagattcagaggatgagcccccttcttgagccctgtagatatgtgaaacttaattatttagatgcatttcatggcaccagaccctaaggtacagttaggtatttcactggagaccacttactttgctttgctttgcttttttttttttttttttttgtatgatttatttcaacttcaaagaaagtagttgagatccttgcacttccaagtgtgatggaaccaggctagggttttctttgtttgtttgggcctcttcaaaacgtttttattctgttataaggaatatgataccttaagcttcaattcacagggaagagaaacatgattataagcaaaagtttgtcaaggaataaggtgagtctttgtgtagattaaaatagttgagaccttgctaaaaagtcagataacactgtaatccacctccaggtttccaagagttaagacgctgccagcaaggagcttgcagtcttgtagctggcccaatatatccaactaattcaaaatgggaaattgtacattttacttaaacaggatttggtagatgggtcttctagatatgttgtcatgaagaaaaatcaggaaaatgtccaagggtgggagcctgtggcttatggcttcacttacccatcacagatagctatgttacactgcaagaacagagttcatggcttttcctgaggtggctggaaaccttaatctcctgaagttaagtcaaaacttaaatgtcaattcacccagctctcttgcctgcaggctgatccagggacttagagcttgttacccagttacaacttcttctagagaagaacttttaagaaggaagcaaaaagaaattcagtttcccccaaggatctcttctttgaggttccatcaagagatgacaaaaggaggctgggcgcggtgtctcacacctgtaatcccagcactttgggaggctgaagtgggcagatcacgaggtcaagagatcaagaccatcctggccaacatggtgaaaccacatttctacaaaatgcaaaaattagctgggcatggtggtgcacgcctgtagtcccagctacttgggaggctgaggcaggagaattgcttgaacccaggaggcggaggtagcagtgagccaagattgcaccactgcattccagcatggccacaaagcaagactccaaaaaatatatatatatatgacaaaagggttaggaagcttttcctctgaggcccacgattacacaaccaaatcagaaaataatattttttttgttttaaacctgtttacataagatggaggtgtttacttaactaagaacctcggtatttactcaaatccggttttgggttttatggctaaaacatttctccagattaaccttatcagtttggaatatgtttttattttccattaaatatttcatccaaatcatactgaattttatttatcatggcttttaatgttctctattcaagtcaaatttctgtttcctaattacttggcttggtgatggcattaatattggttgggagctagatggtctctgctctatttgatgattggcatagagcaatcatacacttggtacttcctagcatttttttttgagacagtatcatgctgtatcatgttgcacagggtggagtgcagtggtgcagtcatagctcactgtaaccttgaactcctgggctcaagtgattcccctgcctcaacttcatgagtagcagggactacagctgtgtaccaccacaccggcctctcctggcttcttaaccacttacattaaaattgagaggagaaaggcattttcagtttctttagttaataaaaagaagccatttctggaggagttttatgcctgtaccagcagaggttcagccttccaggaatctcatcatgatccatactgctgacacaggcctttgtcacctgaagcattcttaaaataaggagactgacattaaacaggacaattgtgaactccactttgtaagcatcatacatatcttacaactcattctgaagactcctttattcttgctcttcccagagagctactgtgtttagtcatgctcactgcactcacaagagaaaaagagtaagtggcatatttgcaggtgttgatggttacaagagcatttttactttttcctccatgtttgttctgcagcatttccattgatgcatgctattgtagacttcttaggttgaccttggccttgacctccaccactagctcatgacccaaaactgaagatgtgcatcagaaagggacagatttggtctttctgctgtaaaagatattctgccttagtgcctgctaatttgttgtgcatagtcctgtgctgccaatcttccccaccaattgttaaggttaatgtgtgttttatatgtgtattcacaaacattaaaatgtaatcagtggattccattattg


>ZNFpseudo1
CATGGATGCCATGTGTATGGGAAGTCTTTCAGCAAGAACTCACACCTTACTTGGCATCAGATAATTCATACTACAGAGAAGCCCTGTGTATGTATTGAATGTGGGGAAAAAAACACAACTCAAGCCTTATACATCATCAGAAAATTCACCATGGATGGTGAAACCCTATCTCTACTAAAAACACAAAAATTATCTGGGTGTGGTGGTGTATGCTGGTAATCCTGGCTACTCAGGAGGCTGAGGCAGAAGAATTGCTTGAACCAGGGAGGCGGAGGTTGCATTGAGCTGAGATTGCGGCACTGCACTCCAGCCTGGGCGACAGAGTGAGACTCCATCTCAAAAGAAAAGAAAAGAAAAGAAAATTCATGGTGGAGAAAAACCCTGTGTGTGTAGTGAATGTGGTAAGGGCTTTAGGGAGACATCAAAGCTTGTTAAACATCAGAGAATTCATACTGGAGAAAAGCCCTATAGGTGGGATGAATGTGACAAAGCCTTTAGTGGGAATTCAAACCTTATTAAACACCAACTGATACATACTGGAGAGAAGCCCTATAAATGTATTGAGTGCGGGAAAGCCTTTAATCCGAAAGCAAATCCCATGCAATATCAGAGAATTCATACAGGAGAGAAACTTTTTGAATGTCAAGAGTGTGGAAAAAGCTTCAGTCAGCCATCACACGTGATTCATAATCAAAGGAAGCATGCTTGAGAGAAGCCCTACAAATGCAGTGAGTGTGGAAGGGGCTTCAAACATCACACCTGATTGATCATCAAAGAATACACACTGGAGAGAAAACTTATGTGTGTGTCATGTGGGAGAGGCTTTATTCAGATGTCACACTTGATTCATTGCCACAGAACACATTCTGTAGAGAAGCCACATGCATGTAGTGTATGTGGGAAATGCTTCAGCCAGAGCTCAACCCTTATTAGACATTAGGTTGTTCACATTATGGATAGGAAGTATAAGTTAAAGGAATGTGGGCAGGCTTTCAGTGTGAGCTAGCTTCTCAGTCACTATCTCACAAATTCTACTGGAGAGGAACCC

>ZNFpseudo2
gtgtagtgtagtgaacatggtaagccttaagaaggactaacacaatttagaaaacaccagagatttcactataaagacaagttctatgaaggcagagtgtgggaaaaccttcaactacagctctaatcttgtgtaacaccaaagaatccacattggaaataagccttatcagtgtaacaaacgtggaaaggcctttgtttaaagttctaacctaatttcccatcacagaactcacactggacagaaaccctatgaatgtaatgagtgtgaaagggcattctctctgggatcaaccattatgaagcatcaggggaatactactggtgagcaaccctacacacatgtaaaaaatgtggaaaggtctttggtcagcatccagcctttattcgacatctggaagttcacacagggtagaaatcttatgaacatactggatgtggaaaaccctaaaaataacagttctgccccgttagccatggaagagcagactggtgaaagcagtgt

>krab_A1
ggcacagtgatgtttgaggaggtggccatgtacctcacacaggaggaggggcagcacctgggaccccctcagagagcactctaccaggatgtgatgctcgagaaccactgcaccctaccagctctg

>krab_A2
gggtcagtgacatttgatgacgtggctgcatatttcacaaggaggaaatggatgcatctagctcttcaccaaaaggcggctggagaactgtgggaacttgac


>krab_B1
ggtttttcagtgtccacactcagggttatctcccagcaggagcaggggaaaatgccatgggtcattgctctgccagggcctccctgcaca

>krab_B2
gaattttcaggcctcaaacctgacctgatcttctagctggagagagaggaagagccatggctgccagatgttccagggactgaaaatacagacaca

>krab_B3
ggatttccagcctgcaaacctgagctaatctccctgctggaatgaggggaggcgccctggatcctcacccggccagaaacgggggagagtgctccc


cDNA sequences of chimpanzee (Pan troglodytes) ZNF genes from the chromosomal region syntenic to human 8q24.3:

>ptZNF251
GAGGCGcACTGAGGCCCCGCAGGGGGCGGGGAAGCCTGCGTGTGCGCAGCTCGGCCCGGCCCGCCCTCTTCATCCTAGCCCGCCCCCTCCCCGGCTCTGGACCCGGTTCTGTGaGAGGGTCCGcTCCGGGCTtGGTGCGACTGCGCAGCTCCTCGGCGCTTCCTCGGTGGCTTCCCCGGGcCGAGCAAACAGGTAAGTTCGGGGAGACTTCAGGCCAGGGCAGTCTCCTGTCTTCTCTTGGAAACCACAGCAGCCCATGCACACAGAATGGCAGCCACATTCCAGCTTCCAGGGCACCAGGAGATGCCGCTGACCTTCCAGGATGTGGCCGTGTACTTCTCTCAGGCGGAGGGGCaGCAGCTGGGCCCtCAGCAGCGGGCGCTCTACCGaGAcGTGATGCTGGAGAACTATGGGAACGTGGCCTCTCTGGGATTCCCTGTCCCTAAGCCGGAGTTGATCTCCCAGCTGGAGCAGGGGAAGGAACTTTGGGTCCTGAATCTTCTGGGAGCcGAGGAACCAGATATCTTGAAAAGCTGCCAGAAAGATTCTGAGGTTGGGACCAAGAAGGAACTATCTATTTTAAACCAAAAATTTTCCGAAGAAGTAAAAACCCCAGAATTTGTATCAAGAAGACTCTTAAGGGATAATGCACAGGCCGCTGAGTTTCGGGAAGCATGGGGCCGTGAGGGCAAACTCAAAGAGtGCGTGGGAAATTCTGCCGGGCAGAGTTTGAACAAACCCAATATTCACAAGAGAGTTTTAACAGAAGCTACCGTGGGCAGGGAAAGATCTTTGGGAGAAAGAACCCAAGAGTGTAGTGCATTTGATAGAAACTTGAATCTGGACCAAAATGTTGTTAGACTTCAAAGAAATAAAACAGGAGAGAGGGTCTTTAAATGTGATATATGCAGCAAAACCTTCAAATATAATTCAGACCTAAGTAGACACCAGAGAAGTCACAgTGGGGAGAAGCCGTACGAATGTGGCCGGTGTGGGCGAGCCTTTACTCACAGCTCAAATCTTGTTCTGCACCATCACATTCACACTGGAAATAAACCATTTAAATGTGATGAATGTGGGAAAACTTTTGGACTCAATTCTCACCTCCGTCTTCATCGGAGAATTCACACTGGAGAAAAACCCTTTGGCTGTGGTGAGTGTGGGAAGGCTTTCAGTCGAAGtTCAACTCTTATTCAACATCGGATCATTCACACAGGAGAGAAACCCTACAAGTGTAATGAATGTGGAAGAGGCTTTAGCCAGAGCCCCCAGTTAACTCAGCATCAGAGAATTCACACTGGAGAGAAGCCGCATGAATGCAGTCACTGTGGGAAGGCCTTCAGTCGAAGCTCCAGCCTTATTCAGCATGAGAGAATTCACACTGGAGAGAAGCCCCATAAATGCAATCAGTGTGGGAAGGCCTTCAGTCAGAGCTCAAGCCTTTTCCTCCATCATCGGGTTCATACTGGAGAGAAACCCTATGTATGTAATGAATGCGGCAGAGCCTTTGGTTTTAACTCTCATCTTACTGAACACGTAAGGATTCACACAGGAGAAAAACCCTATGTTTGTAATGAGTGCGGCAAAGCCTTTCGTCGGAGTTCCACTCTTGTTCAGCATCGAAGAGTTCACACTGGGGAGAAGCCCTACCAGTGCGTTGAATGTGGGAAAGCTTTCAGCCAGAGCTCCCAGCTCACCCTACATCAGCGAGTTCACACTGGAGAGAAGCCCTATGACTGTGGTGACTGTGGGAAGGCCTTCAGCCGGAGGTCAACCCTCATTCAGCATCAGAAAGTTCACAGCGGAGAGACTCGTAAGTGCAGAAAACgTGGTCCAGCCTTTGTTCATGGCTCCAGCCTCACAGCAGATGGACAGATTCCCACTGGAGAGAAGCACaGCAGAGCCTTTAACCATGGTGCAAATCTCATcCTGCGCTGGACAGTTCACACTGGTGAGAAATCCTTTGGATGTAATGAATATGGAAAAGCTTTCAGTCCCACCTCACGACCCACTGAAGATCAGATAATGCATGCTGGGGAAAAGCCCTATAAATGTCAAGAATGTGGAAACGCCTTCAcTGGAAAGTCAACCCTTATTCAACATCAGGTAACTCACACTGGTagGAAACCATGTCATTGCAGTGTGTgTGGGAAAGCCTTCAGCCAGAGTTCACAGCTCACACCACCTCAGCAGACTCGTGTTGGAGAGAAACCTGCTTTAAATGATGGtTCTAAAAGATACTTTATTCATATCAAGAAGATTTTCCAAGAAAGACATTTTTAATGTGATAAATGCAGAAGACAGTTTAGCAACTGTTCACTTGACATTAGAAGATAAGATGGCATAATGAAAGATAAGcTCTAAATATTACTGGCAAAGTAAAATAAATAGTTCAGATGACcACTAAAGTCAAAGTCATTAAATCTGGAAGTAAACACGAGAATTCATTCTGGGAAATCAGGCTGTCTGTGTAAAGGTTACTGCTGTGCTCAGGAGTTGAACCGTGTGGTGCTGTATCCGGATACTCATGACGAATGGATGGAGGGCGTGAAAAGTGAGCCCAGCTGGTGCTCTGGGTCTACCCTACCTGACATCCTTCCAGTCTTATCCTTTGTTTCCTATCCAGGCCCAGGCTTGTGGCTGAGAACATCCACTTTCAGTCCCATATACCTGCCTCCAAGTGTGGTACAGAGAACTTGGGCCTGCTGGGGGCGCTTAGCCTTACTCTCTCCACCACCTCTCCCACCAACCCCCAGATGAACTGCAGGTAGACGTTTCTTCCTTGCTTGGAGCCCCAGTTTTTGCATTTCATTTTCATTAAAATGAAAGGTGGTTTGGTTTTGGTTCTAAGGAGCTCTACAGTTTAACAGAGAGAGGGACCTTAGGGGCCAAGAAAGCAGGGGCCTACCAAGTATCTCCACTTTTGAAAATTGTAATTACTGATAAAAAATTTTTTAAAGGCAGGTTTATTGAAGCATAATTTATAAACAATAAAATTTACTCTTTTTAGTGTA


>ptZNF34
ATTGGCGGGGAGGGAGGGCCGAACGGGCGAGGTTGGGCTGCCGTGCTGCTCGGCGGCGCTGAGGCCAAATAGTTGCATCACATGTATCTAATCCGAGAGTCTCATGCTTCTGGTAGCTCCTCAGTGACCAGCTCCTGCTCACTGCCCTCAGAAAGCCCAACCCTCAGGCAATGGCGGCCTTGTTCCTGTCTGCCCCACCCCAGGCCGAGGTGACCTTCGAGGACGTGGCTGTGTACCTCTCCCGGGAGGAATGGGGCCGCCTGGGCCCTGCTCAGAGGGGCCTCTACAGGGACGTGATGCTGGAGACCTACGGGAACCTAGTCTCACTGGGAGTAGGACCTGCAGGCCCCAAGCCTGGAGTGATCTCGCAGTTGGAGCGAGGGGATGAGCCCTGGGTCCTGGATGTTCAGGGCACCTCTGGGAAAGAGCACCTGAGAGTCAACAGCCCAGCTCTTGGGACCAGAACTGAGTACAAGGAGTTGACTTCACAGGAGACATTTGGTGAGGAAGATCCCCAGGGATCTGAGCCAGTAGAAGCCTGTGACCACATCAGgAAGTCAGAGGGGAGCCTGGAAAAGCTAGTGGAGCAGAGAGGCCCCAGGGCAGTCACACTGACCAAtGGGGAGAGCAGCAGGGAGTCTGGGGGAAACCTCAGGTTGCTGTCAAGACCTGTTCCTGATCAGAGACCTCACAAATGTGATATATGTGAGCAAAGTTTTGAACAGAGATCATATCTCAACAACCATAAGCGTGTACACAGGTCAAAAAAAACAAATACAGTTCGTgACTCTGGGGAAATCTTCAGTGCAAACgTAGTTGTTAAAGATCAGAAAATTCCTACTGGGAAAAAATTGCATTATTGCAGTTACTGTGGGAAAACATTCAGGTACAGTGCCAACCTTGTCAAGCATCAGCGGCTTCACACTGAAGAGAAGCCCTACAAATGTGATGAGTGTGGGAAAGCCTTCAGCCAGAGCTGCGAGTTCATCAATCACCGAAGGATGCACTCAGGAGAGATTCCCTACCGGTGTGACGAGTGTGGGAAGACATTCACCCGGAGGCCCAACCTCATGAAGCACCAGAGGATTCACACTGGGGAGAAACCCTACAAGTGTGGGGAGTGTGGGAAGCACTTTAGCGCCTACTCTTCCCTGATTTATCACCAGAGAATCCACACCGGAGAGAAACCCTATAAATGTAATGACTGCGGGAAAGCCTTCAGTGATGGCTCAATCCTTATCCGACATCGTCGGACTCACACCGGAGAGAAGCCATTTGAGTGCAAGGAATGTGGCAAAGGCTTTACACAAAGTTCTAACCTTATCCAACATCAGAGAATTCACACTGGAGAGAAACCCTATAAATGTAATGAATGTGAGAAAGCTTTCATTCAAAAAACCAAACTCGTGGAACATCAGAGAAGCCACACTGGAGAGAAGCCCTATGAATGCAATGACTGTGGCAAAGTTTTCAGCCAAAGCACACACCTCATCCAGCACCAGAGAATCCACACAGGAGAGAAGCCCTACAAGTGCAGCGAGTGTGGGAAGGCCTTCCACAACAGTTCCAGACTCATCCACCACCAGAGGCTGCACCACGGAGAGAAACCCTACAGATGCAGCGATTGCAAGAAgGCCTTCAGCCAGAGCACGTACTTGATTCAGCACCGGAGGATCCACACCGGGGAGAAGCCCTACAAGTGCAGCGAGTGTGGGAAGGCCTTCCGGCACAGTTCCAACATGTGTCAGCATCAGCGGATTCACCTCCGGGAGGACTTCTCCATGTAACAGTGGCGCGGTGTCCGAGGGCAGAGTCCAGCTGAGCACTTCCTGCATGCGCCCCCGGCACCTGACTCTGCCCTTTATGTATTATCCACACGATGTTTTCACAGAGTGAAAGGACGTTTCTCATTAAACAAACCTCTTTTCTTAAATCAAAAGCAGTGCATGTTCATTTTAGAGAAATTGAGACAGAAAgGTGGGCAAAAAAGAAGCTTCTGTGATCTCTCCCACTTAGAAAAAAAATTCATTGCTGCTGATTTACGAATATTTTCGAGACATACTGGAGAGCATTTTAAAAAGAAAATACCGTAATGCTTGTCATTGCAGCACCTAGGATCTTCACTTTTGCTATTTCCTCAATATCAGAAAATTCACACTGAAGAGAGCACCATATATTTTAACATTgTTACATATTGTAAATTTAAAAGCATCGGCCAGGTGTGGTGGCTCAcGCCTGTAATcCCAGCATTTTGAGAGGCCAAGGTGGGTGGATCACTTGAGGTCAGGAGTTTGAGACCAGCCTGGCCAACATGGTGAAACCCCGTCTCTACTAAAAATACAAAAATcAGCCAGGTGTGGTGGcGGGTGCCTGTAATCCCAGCTACTCGGGAGGCTGAGGCAGGAGCATTGCTTGAACCTGGGAGGCGGAGGTTGCAGTGAGCCtAGACTGTGCCACTGTACTCCAGCCTGGGTGACAGAGCGAGACGCTGTCTCAAAAAATAAATGAAACAAACTTAtCATTCATACTTTTTATGTCAATAGAGTTGAAGTTGCCTTTtttACTGTGGTGAAATAACATAAcatg


>ptZNF517
GGAACCGGAGCCTGAGAGCCGGGCGCCGTGCGCTCCTCCCCGCGCTGTCcCGGCGGCCCAGGAATTCACTGTCTGTAGCATCTGCTCCTCCACAGAGGtACCCTGGAATGGCGATGGCACTCCCGATGCCTGGACCTCAGGAGGCtGTTGTGTTgGAGGATGTGGCTGTGTACTTCACgAGGATAGAGTGGAGTTGCCTGGCCCCCGACCAGCAGGCACTCTACAGGGACGTGATGCTGGAGAACTATGGGAACCTGGCCTCACTAGGCTTTCTTGTTGCCAAACCAGCgCTGATCTCCCTATTGGAGCAAGGAGAGGAGCCGGGGGCCTTGATTCTGCAGGTGGCTGAACAGAGCGTGGCCAAAGCCAGCCTGTGCACAGATTCCAGGATGGAGGCTGGGATCATGGAGTCTCCTCTGCAGAGAAAGCTCTCCAGGCAGGCAGGACTGCCGGGCACCGTGTGGGGGTGCCTCCCCTGGGGGCACCCTGCACCACCCCACCCGCATGGCGGTCCTGAGGgCGGGTCAGATAAACCCACCCACCCCCGGGCTCGGGAGCACAGCGCCTCCCCAAGGGTTCTGCAGGAAGACCcGGGCCGGCCTGTGGGGAGCTCAGCCCCCCGCTACAGGTGCGTGTGCGGCAAGGCGTTCAGATACAACTCGCTGCTTCTCAGGCACCAGATCaTCCACACCGGCGCCAAGCCCTTCCAGTGCACAGAGTGCGGGAAGGCCTTCAAGCAAAGCTCCATCCTGCTGCGGCACCAGCTcATCCACACTGAGGAGAAGCCGTTCCAGTGCGGCGAGTGCGGGAAGGCCTTCCGGCAGAGCACGCAGCTGGCTGCCCACCACCGCGTCCACACCCGCGAGCGGCCCTACGCATGCGGCGAGTGCGGCAAGGCCTTCAGCCGCAGCTCCCGGCTGCTGCAGCACCAGAAGTTCCACACCGGGGAGAAGCCCTTCGCGTGCACcGAGTGCGGCAAGGCGTTCTGCCGCAGGTTCACCCTCAACGAGCACGGCCGCATCCACAGCGGGGAGCGGCCCTACCGGTGCCTGCGGTGTGGGCAGCGCTTCATCCGAGGGTCCTCGCTCCTGAAGCACCACCGGCTGCACGCGCAGGAGGGcGCCCAGGACGGCGGCGCGGGaCAGGGCGCCCTGCTCGGAGCTGCGCAGAGGCCCCAGGCGGGGGACCCGCCCCACGAGTGCCCGGTGTGCGGGAGGCCGTTCCGACACAACTCCCTGCTGCTGCTGCACCTGCGCCTACACACGGGCGAGAAGCCGTTCGAGTGCGCGGAGTGCGGCAAGGCCTTCGGTCGCAAGTCCAACCTCACTCTGCACCAGAAGATCCACACCAAGGAGAAGCCCTTCGCGTGCACCGAGTGCGGCAAGGCGTTCCGCAGGAGCTACACGCTGAACGAGCACTACCGGCTCCACAGCGGCGAGAGGCCATACCGGTGCCGCGCCTGCGGGAGGGCCTGCAGCCGGCTGTCCACCCTCATCCAGCACCAGAAGGTGCACGGCCGCGAGCgCaGGGAGaACACAGAGGGCAGGCGGGCGCCCTGTTGGGCTTCCTGATGACGGGGACGACAGGCCGAGGATTCACGCTGGAAGCCCACCCAAGCCGGCGGGGCCCTAGCGCAGAAATTCAGAACCCCaTGTCCTGAAGGATCTCTgTGTCAGCCAGCCTGGAGTGCAATGGCaCAATCATGGCTTACTGCAGCCTCCAGCTCCCAGGCTCAAGCGcTTCTCCCACCTTAGCCTCCTGAGTAGCTGGGACTAAGGCACACACACTGcACCTGGCAGCTGGCGAAGCATTGTTTCTGAGTGTGTGAGGATGTTTTCAGTGGACTCAGTGAAGATCTGCCCTCAGCGTGGGCAGGCACATCCACTTGGTTGGGGgCCCAGATGGAACACAAAGGGAGAAGAgAAGCAAATTCTCTTTTCTGGAGGGAGACACCCATCTCCTGCCCTTGGACATCAGGACTCCAGGTTCTTCGGCCTTTGGACTCAGGCTTGCCACAGAGGCCTCCCAGGGCTCTCGGCCAGTCAGCCTCAGAATGAGAGTTACACCACTGGCTTCCTTGGTTCAACCgCCTTCTTACCTGGACTGAGCCTCACTaACAGCTTCTCTAGGTCTCCAGCTTGCAGACAGCCTATGGGAGGACTTCTCAGCCTCCATAAGTGTGTGGGCCAGTTCcCCTAATAAATCCCCTCTCCTGGCCGGGCGCGGTAGCTCTtgCCTGTAATCTCAGCATTTTGGGAGGCAGAGGTAGGcGGATCACCTGAGGTCAGGAGTTCgAGACCAGCCTGGCCAACATGGTGAGAcccccCCCCCGTCTCTACTAAAAaTACAAAAAGTAGCTaGGTGTGGTGCTGGGTGCCTGTAATCCCAGCTACTtGGGAGGCTGAAGCAGGAGAATCACTTCGACCTGGGAGGTAGAaGTTGCAGTGAGCCGAGATCGAGCCACTGCACTCCAGCCTGGGTGACAGGGCAAGACTCTGTCTCAAACAAATAAAAATCCCCTCTCCT


>ptZNF7
GGCGCGGACTCGGGTTGtCCTCGGTCCGAGTGATCCCTGGTCGCTTCCTgcGCCCTCCCGCCTTCGGCAcTGGGGTCCCCGCGTCCCCCGGGCCTCCAGtCGGGAAAGCGCGGGGGCTTTGCGGGGCCTTGAGCGCCTGGcGTGGGAGGTGGTCGAGCCCAGCCACCCTCCCCCGCGGCGGCGCGAGGTCTCTCGGCCAGAACACGTGGATGCCCACCCACCACTGAGCCTCATGGAGGTGGTAACATTTGGCGATGTGGCTGTGCACTTCTCTCGGGAGGAGTGGCAGTGTCTGGACCCTGGCCAGAGGGCCCTCTACAGGGAAGTGATGCTGGAGAACCACAGCAGTGTGGCTGGACTAGCAGGATTCCTGGTTTTCAAGCCTGAGCTGATCTCTCGGCTGGAGCAGGGAGAAGAGCCATGGGTCCTCGACCTGCAGGGAGCAGAGGGGACAGAGGCACCAAGGACCTCCAAGACAGATTCTACGATTAGGACTGAAAATGAGCAGGCCTGTGAGGACATGGACATCCTAgAATCAGAATCCTATGGGACAGTGGTCAGAATCTCCCCACAGGACTTTCCTCAGAATCCTGGCTTTGGAGACGTTTCTGATTCTGAGGTCTGGTTAGACAGTCATCTGGGCAGTCCtGGGCTGAAAGTGACAGGCTTTACCTTCCAAAATAACTGTTTGAATGAGGAGACTGTGGTTCCCAAGACCTTCACCAAGGACGCAgCCCAGGGATGTAAGGAGCTGGGAAGCAGCGGCCTGGATTGTCAGCCTCTTGAAAGTCAGaGAGAGAGTGCaGAAGGGATGTCCCAGAGATGCGAGGAGTGTGGCAAAGGCATCAGAGCCACTTCAGAcATCGCTCTGCATTGGGAAATTAATACACAGAAAATTAGCAGATGTCAAGAATGCCAAAAgAAGTTATCTGACTGCTTGCAGGGGAAACATcCAAATAACTGCCATGGAGAGAAGCCaTACGAATGTGCAGAGTGTGGGAAAGTCTTCAGGCTCTGCTCGCAGCTTAATCAGCATCAGAGAATCCACACGGGAGAGAAACCCTTTAAATGCACTGAGTGTGGAAAAGCCTTCCGCCTGAGCTCAAAACTTATTCAGCATCAAAGAATCCACACTGGGGAGAAGCCCTACAGATGTGAGGAATGTGGAAAAGCTTTTGGTCAGAGCTCAAGCCTCATCCACCATCAGAGAATCCACACAGGAGAGAGGCCCTATGGTTGTCaTGAGTGTGGGAAAGCCTTCAGCCAGCAGTCGCAGCTGGTTAGACACCAGAGAACTCACACTGGGGAGAGGCCCTACCCTTGCAAGGAGTGTGGGAAGGCCTTCAGCCAGAGCTCCACCCTAGCCCAGCATCAAAGGATGCATACTGGGGAGAAAGCTCAAATTCTAAAAGCCTCAGACAGTCCAAGCCTTGTTGCACATCAGAGAATTCACGCTGTAGAGAAACCATTTAAGTGTGATGAGTGTGGGAAAGCTTTTAGGTGGATCTCTCGCCTGAGTCAGCATCAGCTGATTCACACTGGAGAGAAGCCTTATAAATGCAACAAGTGTACAAAAGCCTTTGGTTGTAGTTCACGGCTTATTCGCCATCAGAGAACTCACACTGGAGAAAAACCATTTAAATGTGATGAGTGTGGCAAAGGCTTTGTTCAGGGCTCACACCTTATTCAGCATCAGCGAATCCACACTGGAGAGAAACCCTATGTGTGTAATGACTGTGGAAAAGCtTTCAGTCAGAGTTCCAGCCTTATTTACCATCAGAGAATCCATAAAGGAGAGAAGCCCTACGAATGCCTCCAATGCGGAAAAGCCTTCAGcATGAGCACACAGCTTACAATACATCAAAGGGTTCACACcGGAGAGAGGCCCTATAAATGTAATGAATGTGGGAAAGCCTTCAGTCAAAACTCAACCCTTTTCCAACACCAGATAATTCAcGCAGGGGTGAAGCCCTATGAGTGtAGTGAGTGTGGAAAAGCCTTCAGCCGGAGCTCATATCTTATTGAACACCAGAGAATACACACTAGGGCCCAGTGGTTTTACGAgTATGGGAATGCCCTGGAAGGGTCCACCTTTGTGAGCCGTAAAAAGGTTAATACTATAAAGAAACTGCATCAGTGTGAAGACTGTGAGAAGATATTTAGGTGGCGTTCACACCTAATTATACACCAGAGAATTCACACCGGcGAGAAGCCTTATAAATGCAATGACTGTGGCAAAGCTTTTAATCGTAGCTCAAGGCTTACCCAGCATCAAAAAATTCACAcGGGATAGACCACTTACATATAAATGTGTATATATGTGAATAAACCTAcAGCCTTAACTT


>ptZNF250
AGCCCTTTGTCTGAAGGTGCTGCGGGATGCCGTTCCTTCGCGCGTGAGGCTGCGGCgCTGACGCCCCATAGGCTCCTTCAATTTCCGTGATCCTCGGAGTCCCCAGGAGACCAGGTGATGGCAGCAGCCAGACTCCTGCCAGTGCCGGCAGGACCCCAGCCCCTGTCGTTCCAGGCCAAGCTGACCTTCGAGGATGTGGCTGTGCTCCTCTCCCAGGATGAATGGGACCGCCTGTGCCCTGCTCAGAGGGGcCTCTACAGAAATGTGATGATGGAAACCTATGGGAATGTAGTCTCATTGGGACTTCCAGGATCCAAGCCTGACATAATCTCCCAGCTGGAGCGAGGGGAAGATCCCTGGGTCCTGGACAGGAAGGGGGCTAAGAAGAGCCAGGGCCTGTGGAGTGACTACTCAGACAACCTCAAATATGACCACACTACAGCCTGTACACAACAAGACAGTTTATCTTGTCCATGGGAATGTGAAACCAAGGGAGAGAGTCAAAATACAGACTTGAGTCCGAAGCCATTAATTTCAGAGCAAACAGTGATTCTGGGGAAAACACCCTTGGGGAGGATTGATCAAGAAAATAATGAAACAAAGCAAAGCTTCTGTCTGAGTCCAAACTCTGTTGACCACCGTGAAGTTCAGGTCTTAAGCCAAAGCATGCCACTCACTCCGCACCAGGCAGTGCCTAGTGGAGAGAGGCCCTACATGTGTGTTGAGTGTGGGAAGTGCTTTGGCCGGAGTTCCCACCTCCTTCAGCATCAGCGTATCCACACTGGAGAGAAGCCCTATGTGTGCAGTGTATGTGGGAAGGCCTTCAGCCAGAGCTCAGTCCTTAGTAAACACAGGAGAATTCACACAGGTGAGAAGCCCTATGAGTGTAATGAGTGTGGAAAAGCCTTTAGAGTGAGCTCAGATCTTGCTCAGCATCACAAGATACATACAGGAGAGAAGCCTCACGAATGTCTTGAGTGTCGGAAAGCCTTCACTCAACTCTCACATCTCATTCAGCACCAGCGGATCCACACGGGAGAAAGGCCATATGTGTGTCCGTTGTGTGGGAAAGCCTTCAACCATAGCACTGTTCTGCGGAGCCACCAGAGGGTACACACTGGGGAGAAGCCTCACAGGTGCAgTGAGTGTGGGAAAACCTTCAGTGTGAAGAGGACACTGCTGCAGCACCAGAGGATCCACACCGGGGAGAAGCCCTACACGTGCAGCGAGTGTGGGAAGGCCTTCAGCGACCGCTCAGTCCTCATTCAGCACCACAACGTGCACACCGGGGAGAAGCCCTATGAGTGCAGTGAGTGTGGGAAGACCTTCAGCCACCGCTCCACACTGATGAATCACGAGCGGATCCACACCGAGGAAAAGCCCTATGCATGCTACGAATGTGGGAAGGCCTTCGTTCAGCACTCACACCTGATCCAGCACCAGAGAGTCCACACTGGGGAGAAGCCCTAcGTGTGTGGTGAATGTGGGCACGCCTTCAGTGCACGCCGGTCTCTGATCCAGCATGAGAGAATCCACACAGGTGAAAAGCCCTTCCAGTGCACAGAATGTGGCAAAGCCTTCAGCCTGAAAGCAACTCTGATTGTGCACCTGAGGACCCACACGGGCGAGAAGCCATATGAGTGCAATAGCTGCGGGAAGGCCTTCAGCCAGTACTCAGTGCTCATCCAGCACCAGCGGATCCACACAGGCGAGAAGCCCTATGAGTGCGGGGAGTGTGGGCGTGCCTTCAACCAGCATGGCCACCTAATCCAGCACCAGAAAGTGCACAGAAAGTTGTGACCCATGGCTGACACAAGAATCCATTCTCACAGAAACTGCATGTGGAACCACAAGCAGCCTTCAGCCCAAGAGAAGTCTCTGTTAACTCTATAGGAAGCTTTTCTTTGGCGATTCAGTGTCACAAAATAACTCCAGAAAGAAGCACTTAGCGTGCTGTTCCTGTGGAAAAACTTCAGAGACTACCTGTTTTATTTTCCTCAACATCTTGAAGTTATGTTGGAGAGTAATCATACAATTGTAGAGAATTTTGGTAAAAAACAGCCATAATTCTTTAACATTAGTTTATTTGAACTAAGGGAATTTAAGGCATAAGAACCATTATCCCAATAAAATCTTACgTTCCAAATAAAGTTCTTTTTCTAAGAACA


>ptZNF16
AATTCGGGGCGGGACTTCCGGGGGTCAGCCGGCGTTGGCTGAGACGTCTTCGTGCCACGGTGCTGCCTCCcTTCCAAGCGCGACCCGTTGAGGTCCTTGTCATGCCCAGCCTCAGAACTCGCCGTGAGGAGGCAGAGATGGAGCTCTCAGcTCCAGGACCATCCCCtTGGACCCCTGCAGCCCAGGCCCGTGTGAGtGATGCTCCTGCTGTGACCCACCCTGGATCTGCAGCCTGTGGTACCCCCTGCTGTAGTGATACTGAGCTGGAAGCCATCTGCCCTCACTATCAGCAGCCAGATTGTGACACCAGGACTGAAGACAAGGAGTTTCTTCACAAGGAAGACATTCATGAAGATTTGGAATCACAGGCAGAAATATCAGAAAACTATGCTGGTGATGTTTtCCAGGTACCCaAGCTTGGAGATCTGTGTGATGATGTATCAGAAAGAGACTGGGGAGTCCCCGAAGGCAGGAGGCTGCCACAGTCCCTCTCCCAGGAGGGGGACTTCACACCAGCTGCCATGGGGCTCCTTAGGGGCCCCTTAGGGGAGAAAGATCTGGACTGTAAcGGTTTTGACAGTCGCTTCAGTCTGAGCCCAAACCTGATGGCATGTCAGGAAATCCCTACAGAAGAGAGGCCACATCCATATGACATGGGTGGCCAGAGTTTCCAGCACAGTGTGGACCTAACTGGTCATGAGGGGGTTCCCACAGCTGAAAGTCCACTCATATGTAATGAGTGTGGGAAAACCTTCCgAGGAAATCCTGACCTTATTCAGCGTCAAATAGTCCACACTGGGGAGGCTTCCTTTATGTGTGATGATTGTGGGAAAACCTTCAGCCAGAACTCAGTTCTTAAAAACCGTCATCGATCTCATATGAGTGAGAAAGCcTACCAGTGCAGCGAATGTGGGAAAGCCTTCCGAGGGCACTCAGACTTTTCTAGGCATCAGAGTCACCACAGCAGTGAGAGGCCTTATAcGTGTAcTGAATGTGGAAAAGCCTTCAGCCAGAACTCGAGCCTTAAAAAGCACCAAAAGTCTCACATGAGTGAGAAGCCCTATGAATGCAATGAATGTGGGAAGGCTTTTAGGCGGAGCTCAAACCTCATCCAACATCAAAGgATCCATTCTGGGGAGAAACCGTATGTGTGCAGTGAGTGTGGGAAGGCCTTCAGGCGAAGCTCAAACCTCATCAAACACCACAGGACTCACACAGGAGAGAAGCCTTTTGAGTGTGGCGAGTGTGGGAAAGCCTTCAGCCAGAGTGCACACCTGAGGAAGCACCAGAGGGTCCACACTGGAGAGAAGCCTTATGAGTGTAATGATTGTGGCAAGCCCTTCAGTCGGGTCTCCAACCTCATTAAGCACCACAGGGTTCACACTGGAGAGAAGCCCTATAAGTGCAGTGACTGTGGGAAAGCgTTTAGTCAGAGCTCCAGCCTTATTCAGCATCGGcGAATTCACACTGGAGAAAAGCCTCAtGTGTGTAATGTATGTGGAAAAGCCTTTAGTTATAGCTCAGTGCTCCGAAAGCACCAGATCATCCACACGGGAGAGAAGCCGTACAGATGCAGTGTCTGTGGGAAGGCCTTCAGCCACAGCTCAGCCCTCATTCAGCACCAGGGCGTGCACACAGGCGACAAGCCCTACGCCTGCCACGAGTGTGGGAAGACCTTTGGTCGCAGCTCCAACCTCATCCTTCACCAGCGAGTCCACACTGGAGAGAAGCCCTATGAATGTACTGAATGTGGAAAAACCTTCAGCCAGAGCTCAACCCTCATTCAGCATCAGAGGATTCATAATGGGCTGAAGCCCCAcGAATGTAACCAGTGcGGTAAAGCCTTCAACCGAAGCTCAAATCTCATTCACCACCAGAAAGTTCATACTGGGGAAAAACCCTACACCTGTGTTGAATGTGGTAAGGGCTTCAGCCAGAGCTCACACCTCATTCAGCATCAGATAATCCACACtGGCGAGCGCCCCTACAAATGCAGTGAGTGTGGGAAAGCCTTCAGTCAGCGTTCtGTCCTCATCCAGCACCAGAGGATTCACACTGGGGTGAAGCCCTAcGACTGTGCTGCTTGTGGGAAAGCCTTCAGCCAGCGATCAAAGTTGATCAAACACCAGTTGATTCACACCAGGGAATAGGCTGTTGGGCTGGCAGGAGTGAAACtGAGCATAGTTTCCTCTCCAgCTCCTCTgTGGCCATTGTCTaAGCCTCTGCCACTTCCCAGACCGAAAGCCTGGACTCAGCTCACTGCCTACATCCAGTGGCCACATAGCCTGGGACCCTCCTTTGAAACATCCTTTACAGGCTCCCTTCTCACTGCCACTGCTCTTGCCTCTTGTGATGTCTTGGGTTTGGGCTTGTTTTTACAGCTTGTGAACGGACTGCACGTTTGTTACAAGGGAGAAGATTATGGAGGCTGCATATTCATGAATAGAGTTTATTATTATAAccAAAAGTTACCAAAAAAGTAAAGTGACaTTTGGTTTGAG


>ptZNF252exon2
GGTATCcGCCCTGTTTCCTTGCTCTCAAAACACACTTCcAACATGTCATAATCTTCAGTGGTAAAGGCAAATTTCCCCTTGGTTGTATTCTcTTTGGAGTAGAAAATATGGCCAGCAGAATCTGTGATCTTGAGGTGGCTGCACAGGCCACCAGTGCCCCCAGACTGcTCAGAGATCTCGTATGTGCCAGTCACTAGTAGGTCCTTGTGGATCTCCTCATGGAAGCAGTTGTGAGAATTAATGGgCAGATGGAAGGAGATGGCGAGGACCAAGCTGGGGCCCAGCAGGAgCAAAAGCAGCAACGCCGAcGGACAAGGGCCATGCCaGGCTGcTGGGCCAGACAAACCAGACATGGTGCTGGAGACTCATTCCCCCTTTAGCCCTTCTtCTGGGGATCAACACCCcCTACTATGAGTCCTCTACCATGAcGTGTTCAGGCCCCAACgCTGTCCTATCTATGCTCCCCGTCCcCCTGAAAAGCTAGTTTTGACCTTTCtTCAGCTTAGGCAGAGGCCTGTGTCTTTCTCCACGGTTAGAGAgCAGGTGGCAGGGAAGGACACAACCAGCACAAAGACTCAGACCACAGGaCCATCCTGCCCTCTACCTGGGATGAaTTTTCAGGTCTTGCTTCCTTTCTCTGGAGCAGGACTTCAAGCTTCCAAACCTGATGTCATCTCCAGGCTGGAGCGGGGGGACGAACCATAGACCCCTCACATCCTGAGAACTCgGGGGAGCTGGAGCTGGAGGCACAAGgGAGAAGGCTGTGATTCTAGGATTGAAAAAGAGGAAATGATTCCAAAGCAGGACATTTCTGAAGAATTGGAATCCCAGAGAGCAAAATCAGAAGATCATGTAAGGAATATTTTTAAGGAAACTGAAGAGATcAGTAAAACTGAGGGAAAGTTAGAGAATTGCTGGAGAAAATATGCAGTAGAAGGAGTTAAGAACTCATTCTCCCAGAgGAGCAATTTCAGAGAAATTACCATGAGGTATGTGAAAACCCTCTCTAGAGAGAATGGCCAGAAGTTCAATGCTGTTGGAGAAAACTGCATTACAGACTCAAATCCTGCCAAACATCTTAGAGGGTCTAGAGAGGAGAGTCTCCATCCAAGTGTGTCAAGTGTAGAAAACTTGCAACAACATGAGGACCTCATTAACCTCCAGAGTTTCCAATTAGGAGAAAGAGCCTATCAGACAGATGTGTTAGTGAAAGTGCCCAGACAGAGtTCAGTTCTTAGTGAGAATCAGAGGATGAATAATCCAGAGAGATGGTTTGAGAGTACTGGGcaTGGAAAAACTTAgAATCAGAACAGAGCTTTTAACCAGCACCAGAGATTTCATAGTGGAGAGAAGcCCTATGAGCACAATGAATGTGGAAAAGCTTTCAGTcaGCCCTCTATCCTCAGTAAACATCAGAGAATCCACACTGGTAAGAAACcCTACACATGaGAGGATTGTGGCAAATCTTTCAGTGTTCACTCATACTTTATTCAGCATTGTAAAATTCACACTAGAGAAAAACCCTATGAGTGTATTAAATGTGGGAAAGCTTTTAGTACACATTCATCTTATGTTCAACATCTAAAAATTCATACAGGAGAGAAACATCATGAGTGTAATCAGTGTGGGAAAGCCTTTAGTCATAGCTCTAATCTgATTCATCATCAGAGAATTCATAGTGGAGAGAAACCTTACAAGTGCAAAGAATGTGGGAAAGCCTTCAACAGACAATCAcACCTTATTCAGCATCAGAGAATTCATTCTGGAGAGAAACCTTATGACTGTAAGaAGTGTGGCAAAGCCTTCAGTACgCAATTATcTCTCATTCAGCATCAGAtAATTCATACAGGAGAAAAGCCCTATGAATaTAATGAATGTGgAAAATCCTTTAGCCTGAACCGAACTCTTACTGTCCATCAGAGAATTCACACTGGAGAGAAACCTTATAGGTtTAATGAATGTGGGAAATCCTTTAGTCAATGCTCACAAGTTATTCAACATAAGAGAATTCACACTGGAGAGAAACCTTATATCTGCAATGAGTGTGGAAAATCATTTGGTGCTCGcCTATCCCTTATCCAGCATCAGAGAATTCACACTGGAGAGAAACCTTATGGTTGTAGTGTGTGTGGGAAAACCTTTAGTCAAAAGGGACATCTTATTCAGCATCAGTGAATTCACACAGGAGAGAAACCCTATGAATGTAGTGAGTGTGGAAAAGCTTTCAGCCAGAGTTTTAATCTTATTCACCATCAAAGAACACACAATGGTcAGAAGcCCTATGAATGTAATGAATGTGATAAAGCCTTCAGTgTGCTTTCTTCCCTTGTTCAACATCAGAGAATACATAATGGAGAgAAACCCTATGAGTGTCACAAATGTGGGAAGGCCTTTAGCCAGGtGTCACACCTTATTCAGCATCAGAGGAGTCACAcTGGTGAGAAACCCTATGGTGTAATGAGTGTGGGAAAACCTTTGGGCAGATATCCACCCTAATTAAGCATGAGAGAACACACAATGGAGAGAAGCCCTATGAGTGCAGTGACTGTGGGAAGGCCTTCAGCCAGAGTGCACACCTTATCCgCCATCAAAGAATTCACACTGGAcAGAATCCCTATGAGTGCAGTGAcTGTGGGAAGaCCTTCAATGTTcGTTCCTCTCTCATTCAGCATCACAGAATTCATACTGGTGAGAAACCTTATGAAgGTAGTGACTGTGGCAAGGCGTTCAGTCAGCATTCACAATTTATCCAACATCAGAGAATTCACACTGGAGAGAAACCCTACATGTGCAATGAGTGTGAGAAATCCTTCAGTGCAcGCTTATCCCTTATCCAACACAAaAGAATTCACACTGGAGAGAAACCCTATGTATGcGCCAAATGTGGAAAATCCTTCTGACAAAGCTCTCACCTTATTCAACATCAGAGAATTCACAGTGGGGAGCAACCTtATACGTGTAATCaATGTGgAAAAACCTTCAGTcAGAGAATAACTCTTAGTAGTCATGAGAAAATCCATACAATTCACATTAcAGAGCAAGTCTATGAATGTAGTAAGTGTGGGGAACTCTAGCGCACAGTCATgTTTCATTCAACATTGTACAGTTCACAGTGGAGAGTAACTGATTAGTCTATAAATTtTTCACAGTTATACATTATATGAGAGTCAcACTgGTGAGAAATGAGTCACTTTATGGTTTTCCTTTTGGAGATTCAaCTCTTAAGGGATTTCACTCTCCCAGTGAGCTGCTAACTAAAGGCAGGCTGCTGAAGTATATAGCAGCAATCCCACAATAGCACACCTTGCTTCAaATTCAGAGGATGAGCCCCCTTCTTGAGCCCTGTAGATATGTGAAACTTAATTATTTAGATGCATTTCATGGCACCAGACCtTAAGGTACAGTTAGGTATTTCACTGGAGACCACTTACTTTGCTTTGCTTTGCtttgctttgtcTTTTTTTTTTTTTTTTTTTTgGTATGATTTATTTCAACTTCAAAGAAAGTAGTTGAGATCCTTGCACTTCCAAGTGTGATGGAAtCAGGCTAGGGTTTTCTTTGTTTGTTTGGGCCTCTTCAAAACGTTTTTATTCTGTTATAAGGAATATGgTACCTTAAGCTTCAATTCACAGGGAAGAGAAACATGATTATAAGCAAAAGTTTGTCAAGGAATAAGGTGAGTCTTTGTGTAGATTAAAATAGTTGAGACCTTGCTAAAAAGTCAaATAACACTGTAATCCACCTCCAGGTTTCCAAGAGTTAAGAtGCTGCCAGCAAGGAGCTTGCAGTCTTGTAGCTGGCCCAATATATCCAACTAATTCAAAATGGGAAATTGTACATTTTACtTTAAACAGGATTTGGTAGATGGGTCTTCTAGATATGTTGTCATGAAGAAAAAcCAGGAAAATGTCCAAGGGTGGGAGCCTGTGGCTTATGGCTTCACTTACCCATCACAGATAGCTATGTTcCACcGCAAGAACAGAGTTCATGGCTTTTCCTGAGGTGGCTGGAAACCTTAATCTCCTGAAGTTAAGTCAAAACTcAAATGTCAATTCACCCAGCTCTCTTGCCTGCAGGCTGATCCAGGGACTTAGAGCTTGTTACCCAGTTACAACTTCTTCTAGAGAAGAACTTTcAAGAAGGAAGCAAAAAGAAATTCAGTTTCCCCCAAGGATCTCTTCTTTGAGGTTCCATCAAGAGATGACAAAAGGgGGCTGGGtGCGGTGTCTCACACCTGTAATCCCAGCACTTTGGGAGGCTGAAGcGGGCAGATCACGAGGTCAAGAGATCAAGACCATgCTGGCCAACATGGTGAAACCACATTTCTACAAAATGCAAAAATTAGCTGGGCATGGTGGTGCACGCCTGTAGTCCCAGCTACTTGGGAGGCTGAGGCAGGAGAATTGCTTGAACCCAGGAGGCGGAGGTAGCAGTGAGCCAAGATTGCACCACTGCATTCCAGCcTGGCCACAAAGCAAGACTCCAAAAAATATATATATATATGACAAAAGGGTTAGGAAGCTTTTCCTCTGAGGCCCACGATTACAgAACCAAATCAGAAAATAATAaTTTTTTTGTTTTAAACCTGTTTACATAAGATGGAGGTGTTTACTTAACTAAGAACCTCGGTATTTACTCAAATCCGGTTTTGGGTTTTATGGCTAAAACATTTCTCCAGATTAACCTTATCAGTTTGGAATATGTTTTTATTTTCCATTAAATATTTCATCCAAATCATACTGAATTTTATTTATCATGGCTTTTAATGTTCTCTATTCAAGTCagAAATTTCTGTTTCCTAATTACTTGGCTTGGTGATGGCATTAATATTGGTTGGGAGCTAGATGGTCTCTGCTCTATTTGATGATTGGCATAGAGCAATCATACACTTGGTACTTCCTAGCAtTTTTTTTTTGAGACAGTATCATGCTGTATCATGTTGtACAGGGTGGAGTGCAGTGGTGCAGTCATAGCTCACTGTAACCTTGAACTCCTGGGCTCAAGTGATTCCCCTGCCTCAACTTCATGAGTAGCAGGGACTACAGCTaTGTACCACCACACCGGCCTCTCCTGGCTTCTTAACCACTTACATTAAAATTGAGAGGAGAAAGGCATTTTCAGTTgCTTTAGTTAATAAAAAGAAGCCATTTCTGGAGGAGTTTTATGCCTGTACCAGCAGAGGTTCAGCCTTCCAGGAATCTCATCATGATCCATACTGCTGACACAGGCCTTTGTCACCTGAAGCATTCTTAAAATAAGGAGACTGACATTAAACAGGACAATTGTGAACTCCACTTTGTAAGCATCATACATATCTTACAACTCATTCTGAAGACTCCTTTATTCTTGCTCTTCCCAGAGAGCTACTGTGTTTAGTCATGCTCACTGtACTCACAAaAGAAAAAGAGTAAGTGGCATATTTGCAGGTGcTGATGGTcACAAGGCATTTTTACTTTTTCCTCCATGTcTGTTCTGCAGCATTTCCATTGATGCATGCTATTGTAGACTTCTTAGGTTGACCTTGGCCTTGACCTCCACCACTAGCTCtTGACCCAAAACTGAAGATGTGCATCAGAAAGGGACAGATTTGGTCTTTCTGCTGTAAAAGATATTCTGCCTTAGTGCCTGCTAATTTGTTGTGCATAGTCCTGTGCTGCCAATCTTCCCCACCAATTGTTAAGGTTAATGTGTGTTTTATgTGTGTATTCACAAACATTAAAATGTAATCAGTGGATTCCATTATTG

>ptZNF252exon5
tccggcccagggggtttggctctccgcggctcggggtttcGCGTTCGCGCTCCTTCCCTTCCCGTGGTCGAGCCGAgtgagtcccgagctagggcgcctggtgcggaggtgccggagtggcgctggggcgggcagggacgGTCCTGACCTGAGGGCTGCATCAAGATCTTGTCATTCCACATCGTGGTTTCCTTTGAGGATGTGGCTGTACCCCTCTCCCAGGAGGAGTGGGACTGTCTGATCCCTGCTCAGAGGGGCCTCTACAAGGATGTGATGATGGaGACCTgTGGGAACCTACTCTCATTAGTAGGTGAATGGTTAAGCAAACTGTGGTACATCCATACCATGGaATACtACTCAgCAATCAAAAGGAACTGCCCAGACTTCACCACaATGCAATATATGCAGACTTCAAGCTTCCAAACCTGATGTCATCTCCAGGCTGGAGCGGGGGGACGAACCATAGACCCCTCACATCCTGAGAACTCgGGGGAGCTGGAGCTGGAGGCACAAGgGAGAAGGCTGTGATTCTAGGATTGAAAAAGAGGAAATGATTCCAAAGCAGGACATTTCTGAAGAATTGGAATCCCAGAGAGCAAAATCAGAAGATCATGTAAGGAATATTTTTAAGGAAACTGAAGAGATcAGTAAAACTGAGGGAAAGTTAGAGAATTGCTGGAGAAAATATGCAGTAGAAGGAGTTAAGAACTCATTCTCCCAGAgGAGCAATTTCAGAGAAATTACCATGAGGTATGTGAAAACCCTCTCTAGAGAGAATGGCCAGAAGTTCAATGCTGTTGGAGAAAACTGCATTACAGACTCAAATCCTGCCAAACATCTTAGAGGGTCTAGAGAGGAGAGTCTCCATCCAAGTGTGTCAAGTGTAGAAAACTTGCAACAACATGAGGACCTCATTAACCTCCAGAGTTTCCAATTAGGAGAAAGAGCCTATCAGACAGATGTGTTAGTGAAAGTGCCCAGACAGAGtTCAGTTCTTAGTGAGAATCAGAGGATGAATAATCCAGAGAGATGGTTTGAGAGTACTGGGcaTGGAAAAACTTAgAATCAGAACAGAGCTTTTAACCAGCACCAGAGATTTCATAGTGGAGAGAAGcCCTATGAGCACAATGAATGTGGAAAAGCTTTCAGTcaGCCCTCTATCCTCAGTAAACATCAGAGAATCCACACTGGTAAGAAACcCTACACATGaGAGGATTGTGGCAAATCTTTCAGTGTTCACTCATACTTTATTCAGCATTGTAAAATTCACACTAGAGAAAAACCCTATGAGTGTATTAAATGTGGGAAAGCTTTTAGTACACATTCATCTTATGTTCAACATCTAAAAATTCATACAGGAGAGAAACATCATGAGTGTAATCAGTGTGGGAAAGCCTTTAGTCATAGCTCTAATCTgATTCATCATCAGAGAATTCATAGTGGAGAGAAACCTTACAAGTGCAAAGAATGTGGGAAAGCCTTCAACAGACAATCAcACCTTATTCAGCATCAGAGAATTCATTCTGGAGAGAAACCTTATGACTGTAAGaAGTGTGGCAAAGCCTTCAGTACgCAATTATcTCTCATTCAGCATCAGAtAATTCATACAGGAGAAAAGCCCTATGAATaTAATGAATGTGgAAAATCCTTTAGCCTGAACCGAACTCTTACTGTCCATCAGAGAATTCACACTGGAGAGAAACCTTATAGGTtTAATGAATGTGGGAAATCCTTTAGTCAATGCTCACAAGTTATTCAACATAAGAGAATTCACACTGGAGAGAAACCTTATATCTGCAATGAGTGTGGAAAATCATTTGGTGCTCGcCTATCCCTTATCCAGCATCAGAGAATTCACACTGGAGAGAAACCTTATGGTTGTAGTGTGTGTGGGAAAACCTTTAGTCAAAAGGGACATCTTATTCAGCATCAGTGAATTCACACAGGAGAGAAACCCTATGAATGTAGTGAGTGTGGAAAAGCTTTCAGCCAGAGTTTTAATCTTATTCACCATCAAAGAACACACAATGGTcAGAAGcCCTATGAATGTAATGAATGTGATAAAGCCTTCAGTgTGCTTTCTTCCCTTGTTCAACATCAGAGAATACATAATGGAGAgAAACCCTATGAGTGTCACAAATGTGGGAAGGCCTTTAGCCAGGtGTCACACCTTATTCAGCATCAGAGGAGTCACAcTGGTGAGAAACCCTATGGTGTAATGAGTGTGGGAAAACCTTTGGGCAGATATCCACCCTAATTAAGCATGAGAGAACACACAATGGAGAGAAGCCCTATGAGTGCAGTGACTGTGGGAAGGCCTTCAGCCAGAGTGCACACCTTATCCgCCATCAAAGAATTCACACTGGAcAGAATCCCTATGAGTGCAGTGAcTGTGGGAAGaCCTTCAATGTTcGTTCCTCTCTCATTCAGCATCACAGAATTCATACTGGTGAGAAACCTTATGAAgGTAGTGACTGTGGCAAGGCGTTCAGTCAGCATTCACAATTTATCCAACATCAGAGAATTCACACTGGAGAGAAACCCTACATGTGCAATGAGTGTGAGAAATCCTTCAGTGCAcGCTTATCCCTTATCCAACACAAaAGAATTCACACTGGAGAGAAACCCTATGTATGcGCCAAATGTGGAAAATCCTTCTGACAAAGCTCTCACCTTATTCAACATCAGAGAATTCACAGTGGGGAGCAACCTtATACGTGTAATCaATGTGgAAAAACCTTCAGTcAGAGAATAACTCTTAGTAGTCATGAGAAAATCCATACAATTCACATTAcAGAGCAAGTCTATGAATGTAGTAAGTGTGGGGAACTCTAGCGCACAGTCATgTTTCATTCAACATTGTACAGTTCACAGTGGAGAGTAACTGATTAGTCTATAAATTtTTCACAGTTATACATTATATGAGAGTCAcACTgGTGAGAAATGAGTCACTTTATGGTTTTCCTTTTGGAGATTCAaCTCTTAAGGGATTTCACTCTCCCAGTGAGCTGCTAACTAAAGGCAGGCTGCTGAAGTATATAGCAGCAATCCCACAATAGCACACCTTGCTTCAaATTCAGAGGATGAGCCCCCTTCTTGAGCCCTGTAGATATGTGAAACTTAATTATTTAGATGCATTTCATGGCACCAGACCtTAAGGTACAGTTAGGTATTTCACTGGAGACCACTTACTTTGCTTTGCTTTGCtttgctttgtcTTTTTTTTTTTTTTTTTTTTgGTATGATTTATTTCAACTTCAAAGAAAGTAGTTGAGATCCTTGCACTTCCAAGTGTGATGGAAtCAGGCTAGGGTTTTCTTTGTTTGTTTGGGCCTCTTCAAAACGTTTTTATTCTGTTATAAGGAATATGgTACCTTAAGCTTCAATTCACAGGGAAGAGAAACATGATTATAAGCAAAAGTTTGTCAAGGAATAAGGTGAGTCTTTGTGTAGATTAAAATAGTTGAGACCTTGCTAAAAAGTCAaATAACACTGTAATCCACCTCCAGGTTTCCAAGAGTTAAGAtGCTGCCAGCAAGGAGCTTGCAGTCTTGTAGCTGGCCCAATATATCCAACTAATTCAAAATGGGAAATTGTACATTTTACtTTAAACAGGATTTGGTAGATGGGTCTTCTAGATATGTTGTCATGAAGAAAAAcCAGGAAAATGTCCAAGGGTGGGAGCCTGTGGCTTATGGCTTCACTTACCCATCACAGATAGCTATGTTcCACcGCAAGAACAGAGTTCATGGCTTTTCCTGAGGTGGCTGGAAACCTTAATCTCCTGAAGTTAAGTCAAAACTcAAATGTCAATTCACCCAGCTCTCTTGCCTGCAGGCTGATCCAGGGACTTAGAGCTTGTTACCCAGTTACAACTTCTTCTAGAGAAGAACTTTcAAGAAGGAAGCAAAAAGAAATTCAGTTTCCCCCAAGGATCTCTTCTTTGAGGTTCCATCAAGAGATGACAAAAGGgGGCTGGGtGCGGTGTCTCACACCTGTAATCCCAGCACTTTGGGAGGCTGAAGcGGGCAGATCACGAGGTCAAGAGATCAAGACCATgCTGGCCAACATGGTGAAACCACATTTCTACAAAATGCAAAAATTAGCTGGGCATGGTGGTGCACGCCTGTAGTCCCAGCTACTTGGGAGGCTGAGGCAGGAGAATTGCTTGAACCCAGGAGGCGGAGGTAGCAGTGAGCCAAGATTGCACCACTGCATTCCAGCcTGGCCACAAAGCAAGACTCCAAAAAATATATATATATATGACAAAAGGGTTAGGAAGCTTTTCCTCTGAGGCCCACGATTACAgAACCAAATCAGAAAATAATAaTTTTTTTGTTTTAAACCTGTTTACATAAGATGGAGGTGTTTACTTAACTAAGAACCTCGGTATTTACTCAAATCCGGTTTTGGGTTTTATGGCTAAAACATTTCTCCAGATTAACCTTATCAGTTTGGAATATGTTTTTATTTTCCATTAAATATTTCATCCAAATCATACTGAATTTTATTTATCATGGCTTTTAATGTTCTCTATTCAAGTCagAAATTTCTGTTTCCTAATTACTTGGCTTGGTGATGGCATTAATATTGGTTGGGAGCTAGATGGTCTCTGCTCTATTTGATGATTGGCATAGAGCAATCATACACTTGGTACTTCCTAGCAtTTTTTTTTTGAGACAGTATCATGCTGTATCATGTTGtACAGGGTGGAGTGCAGTGGTGCAGTCATAGCTCACTGTAACCTTGAACTCCTGGGCTCAAGTGATTCCCCTGCCTCAACTTCATGAGTAGCAGGGACTACAGCTaTGTACCACCACACCGGCCTCTCCTGGCTTCTTAACCACTTACATTAAAATTGAGAGGAGAAAGGCATTTTCAGTTgCTTTAGTTAATAAAAAGAAGCCATTTCTGGAGGAGTTTTATGCCTGTACCAGCAGAGGTTCAGCCTTCCAGGAATCTCATCATGATCCATACTGCTGACACAGGCCTTTGTCACCTGAAGCATTCTTAAAATAAGGAGACTGACATTAAACAGGACAATTGTGAACTCCACTTTGTAAGCATCATACATATCTTACAACTCATTCTGAAGACTCCTTTATTCTTGCTCTTCCCAGAGAGCTACTGTGTTTAGTCATGCTCACTGtACTCACAAaAGAAAAAGAGTAAGTGGCATATTTGCAGGTGcTGATGGTcACAAGGCATTTTTACTTTTTCCTCCATGTcTGTTCTGCAGCATTTCCATTGATGCATGCTATTGTAGACTTCTTAGGTTGACCTTGGCCTTGACCTCCACCACTAGCTCtTGACCCAAAACTGAAGATGTGCATCAGAAAGGGACAGATTTGGTCTTTCTGCTGTAAAAGATATTCTGCCTTAGTGCCTGCTAATTTGTTGTGCATAGTCCTGTGCTGCCAATCTTCCCCACCAATTGTTAAGGTTAATGTGTGTTTTATgTGTGTATTCACAAACATTAAAATGTAATCAGTGGATTCCATTATTG

>ptZNFpseudo1
CATGGATGCCATGTGTATGGGAAGTCTTTCAGCAAGAACTCACACCTTACTTGGCATCAGATAATTCATACTACAGAGAAGCCCTGTGTATGTATTaAATGTGGGGAAAAAAtCACAACTCAAGCCTTATACATCATCAGAAAATTCACCATGGATGGTGAAACCCTATCTCTACTAAAAACACAAAAATTATCTGGGTGTGGTGGTGTATGCTGGTAATCCcGGCTACTCAGGAGGCTGAGGCAGAAGAATTGCTTGAACCAGGGAGGCGGAGGTTGCATTGAGCTGAGATTGCGGCACTGCACTCCAGCCTGGGCGACAGAGTGAGACTCCATCTCAAAAAAAAGgAAAAGAAAAGaAAAATTCATGGTGGAGAAAAACCCTGTGTGTGTAGTGAATGTaGTAAGGGCTTTAGGGAGACATCAAAGCTTGTTAAACATCAGAGAATTCATACTGGAGAAAAGCCCTATAGGTGGGATGAATGTGACAAAGCCTTTAGTGGGAATTCAAACCTTATTAAACACCAACTGATACATACTGGAGgGAAGCCCTATAAATGTATTGAGTGtGGGAAAGCCTTTAATCCaAAAGCAAATCCCATGCAATATCAGAGAATTCATACAGGAGAGAAACTTTTTGAATGTaAAGAGTaTGGAAAAAGCTTCAGTCAGCCATCACACGTGATTCATAATCAAAGGAAGCATGCTTGAGAGAAGCCCTACAAATGCAGTGAGTGTGGAAGGGGCTTCAAACATCACACCTGATTGATCATCAAAGAATACACACTGGAGAGAAAACTTATGTGTGTGTCATGTGGGAGAGGCTTTATTCAGATGTCACACTTGATTCATTGCCACAGAACACATTCTGTAGAGAGCCACATGCATGTAGTGTATGTGGGAAATGCTTCAGCCAGAGCTCAACCCTTATTAGACATTAGGTTGTTCACATTATGGATAGcAAGTATAAGTTAAAGGAATGTGGGCAGGCTTTCAGTGTGAGCTAGCTTCTCAGTCACTATCTCACAAATTCTACTGGAGAGGAACCC

>ptZNFpseudo2
GTGTAGTGTAGTGAACATGGTAAGCCTTAAGAAGGACTAACACAATTTAGAAAACACCAGAGATTTCACTATAAAGACAAGTTCTATGAAGGCAGAGTGTGGGAAAACCTTCAACTACAGCTCTAATCTTGTGTAACACCAAAGAATCCACATTGGAAATAAGCCTTATCAGTGTAACAAAtGTGGAAAGGCCTTTGTTTAAAGTTCTAAcCCTAATTTCCCATCACAGAACTCACACTGGAgAGAAACCCTATGAATGTAATGAGTGTGAAAGGGCATTCTCTCTGGGATCAACCATTATGAAGCATCAGGGGAATACTACTGGTGAGCAACCCTACACACATGTAAAAAATGTGGAAAGGTCTTTGGTCAGCATCCAGCCTTTATTCGACATCTGGAAGTTCACACAGGGTAGAAATCTTATGAACATACTGGATGTGGAAAACCCTAAAAATAACAGTTCTGCCCCcTTAGCCATGGAAGAGCAGACTGcTGAAAGCAGaGT


cDNA sequences of rhesus monkey (Macaca mulatta; one from M. fascicularis) ZNF genes from the chromosomal region syntenic to human 8q24.3:


>mmulZNF251 [GenBank:XM_001105603alt]
gattctgagtttgggaccaagaaggaactatctattttaaaccaaaaattttccgaagAAGTAAAAACCCCAGAATTTGTATCAAGAAGTCTCTTAAGGGATAATGCACAGGCAGCTGAGTTTCGGGAAGCATGGTGCCATGAGGGTAAACTCGAAGAGCACCTGGGAAATTCTGCCGGGCAGAGTTTGAACAAACCCAATATTCACAAGAGAGTTTTAACAGAAGCTACCATGGGTAGGGAAAGATCTTTGGGAGAAAGACCCCAAGAGGGTAGTGCATTTGATAGAAACTTGAATCTGAACCAAAATGTTGTTAGACTTCAAAGAAATAAAACAGGAGAGAGGGTCTTTAAATGTGATATATGCAGCAAAACCTTCAAATACAATTCAGACCTAAGTAGACATCAGAGAAGTCACACTGGGGAGAAGCCATACCAATGTGGCCGGTGTGGACGAGCCTTTACTCACAGCTCAAATCTTGTTCTGCACCATCACATTCACACTGGAAATAAACCATTTAAATGTGATGAATGTGGGAAAACTTTTGGACTCAATTCTCACCTCCGTCTTCATCGGAGAATTCACACTGGAGAAAAACCCTTTGGCTGTGGTGAGTGTGGGAAGGCTTTCAGTCGAAGCTCAACTCTTATTCAACATCGGATAATTCACACAGGAGAGAAACCCTACAAGTGTAATGAATGTGGAAGAGGCTTTAGCCAGAGCCCCCAGTTAACTCAGCATCAGAGAATTCACACTGGAGAGAAGCCGCATGAATGCAGGCACTGTGGGAAGGCCTTCAGTCGAAGTTCCAGCCTTATTCAGCATGAGAGAATTCACACTGGAGAGAAGCCCCATAAATGCAATCAGTGTGGGAAGGCCTTCAGTCAGAGCTCAAGCCTTTTCCTCCATCATCGGGTTCATACTGGAGAGAAACCCTATGTATGTAATGAATGCGGTAGAGCCTTTGGTTTTAACTCTCATCTTACTGAACATGTAAGGATTCACACAGGAGAAAAACCCTATGTTTGTAATGAGTGCGGCAAAGCCTTTCGTCGGAGTTCCACTCTTGTTCAGCATCGAAGAGTTCACACTGGGGAGAAGCCCTACCAATGCGTTGAATGTGGGAAAGCTTTCAGCCAGAGCTCCCAACTCACCCTACATCAGCGAGTTCACACTGGAGAGAAGCCCTATGAATGTGGTGACTGTGGGAAGGCCTTCAGCCGGAGGTCAACCCTCATTCAGCATCAGAAAATTCACAGTGGAGAGACTCGCAAGTGCAGAAAACGTGGTCCAGCCTTTGTTCATGGCTCTAGCCTTACACCAGATGGACAGATTCCCACTGGAGAGAAGCACAGCAGAGCCTTTAGCCATGGTGCAAATCTCATTCTGCGCTGGACAGTTCACACTGGTGAGAAATCCTTTGGATGTAATGAATATGGAAAAGCTTTCAGTCCCACCTCACAACCCACGGAAGATCAGAAAATGCATGCTGGGGAAAAGCCCTATAAATGTCAAGAATGTGGAAACGCCTTCAGTGGAAATTCAGCCCTTATTCGACATCAGGTAACTCACACTGGTGGGAAACCCTGTCATTGCAGTGTGTGTGGGAAAGCCTTCAGCCAGAGTTCACAGCTCACACCACCTCAGCAGACTCATGTTGGAGAGAAACCTGCTTTAAATGATGGATCTAAAAGATACTTTATTCAAATCAAAAAGATTTTCCAAGAAAGACATTTTTAAAGTGATAAATGCAGAAGACAATTTAGCAACTGTTCACTTTACATTAGAAGATGGCATAATGAAAGATAAATAAGGTCTAAATATTCCTGGCAAAGTAAAATAAATAGTTCAGATGACTACTAAAAGTCATTTAAAGTCATTAAATCTGGGAGTAAACACAAGAGAATTCATTCTGGGAAATCAGGCTGTCTGTGTAAAGGCTACTGCCGTGCTCAGGAGTTGAACCGTGTGGTGCTGTGTCCGGATACTCATGATGAATGGATGGAGGGTGTGAAAAATGAGCCCAGCTGGTGCTCTGGGTCTACCCTGCCTGACATCCTTCCAGTCTTATCCTTTGTTTCCTATCCAGGCCCAGGCTTGTGGCTGAGAACATCCACTTTCAGTCCCATACATCTGCCTCCAGGCAGAGAACTTGCGCCTGCTGGTGGGGCTTAGCCTTATTCCTTCCACCACCTCTCCCACCAGCCCGCAGAGGAACTGCAGGTAGACGTTT


>mmulZNF34 [ENSEMBL:ENSMMUT00000038746]
ATGGCAGCCTGGTTCCTGTCTGCCCCACCCCAGGCCGAGGTGACCTTCGAGGATGTGGCTGTGTTCCTCTCTCGGGAGGAATGGGGCCGCCTGGGCCCTGCTCAGAGGGGCCTCTACAGGGACGTGATGCTGGAGACCTACGGGAACCTAGTCTCACTGGGAGTAGGACCTGCAGGCCCCAAGCCTGGAGTGATCTCGCAGTTGGAGCGAGGGGATGAGCCCTGGGTCCTGGATGTTCAGGGCACCTCTGGGAGAGAGCACCTGAAAGTCAACAGCCCAGCTCTTGGGACCAGAACTGAGTACAAGGAGTTGACTTCACGGGAGACATTTGGTAAGGAAGAACTAGGCCAGCTGAGGGGGATTTTTCCCCAGGGATCTGAGCCAGTAGAAGCCTGTGACCACATCAGGAAGGCAGAGGGGAGCCTGGAAAAGCTGGAGCAGAGAGGCCCCAGGGCAGTCACACTGACCAACGGGGAGAGCAGCAGGGGGTCTGGGGGAAGCCTCAGGTTGGTGTCACGACCTGTTCCCGATCAGAGCCCTCACAAATGTGATATATGCGAGCAAAGTTTTGAACAGAGATCTTATCTCAACAACCATAAGCGCATACACAGGTCAAAAAAAACAAAGACAGTTCGTGACTCTGGGGAAATCTTGAGTGCAAACTTAGTTGTTAAAGAAGATCAGAAAATTCCTACTGGGAAAAAATTGCATTATTGCAGTTACTGTGGGAAAACATTCAGGTACAGTGCCAACCTTGTCAAGCATCAGCGGCTTCACAGTGAAGAGAAGCCCTACAAATGTGATGAGTGTGGGAAAGCCTTCAGCCAGAGCTGCGAGTTCATCAACCACCGAAGGATGCACTCAGGAGAGATTCCCTACCGGTGTGACGAGTGTGGTAAGACATTCACCCGGAGGCCCAACCTCATGAAGCACCAGAGGATTCACACTGGGGAGAAACCCTACAAGTGTGGTGAGTGTGGGAAACACTTTAGCGCCTACTCTTCCCTTATTTATCACCAGAGGATCCACACTGGAGAGAAACCCTATAAATGTAATGACTGTGGGAAAGCCTTCAGTGATGGCTCAATCCTTATCCGCCATCGTCGGACTCACACTGGAGAGAAGCCATTTGAGTGCAAGGAATGTGGCAAGGGCTTTACCCAAAGTTCTAACCTTATCCAGCATCAGAGAATTCACACTGGAGAGAAACCCTATAAATGTAATGAATGTGAGAAAGCTTTCATTCAAAAAACCAAACTTGTGGAACATCAGAGAAGCCACACTGGAGAGAAGCCCTATGAATGCAATGACTGTGGCAAAGTTTTCAGCCAGAGCACACACCTCATCCAGCATCAGAGAATTCACACAGGAGAAAAGCCCTACAAGTGCAGCGAGTGTGGGAAGGCCTTCCACAACAGTTCCAGACTCATTCACCACCAGAGGTTGCACCACGGAGAGAAACCCTACAAATGCAGTGATTGCAAGAAAGCCTTCAGCCAGAGCACTTACTTGATTCAGCACCGGAGGATCCACACCGGGGAGAAGCCCTACAAGTGCAGTGAGTGTGGGAAGGCCTTCCGGCACAGTTCCAACATGTGTCAGCATCAGCGGATTCACCTCCGGGAGGACTTCTCCGTGTAA


>mmulZNF517 [ENSEMBL:ENSMMUT00000018806]
ATGACGATGGCACTTCCGATGCCTGGACCCCAGGAGGCTGTTGTGTTCGAGGATGTGGCTGTGTACTTCACGAGGATAGAGTGGAGTTGCCTGGCCCCCGACCAGCAGGCCCTCTACAGGGACGTGATGCTGGAGAACTATGGGAACCTGGCCTCGCTAGGCTTTCTTGTTGCCAAACCAGCACTGATCTCCCTCTTGGAGCAAGGAGAGGAGCCGGGGGCCTTGATTCTGCAGGTGGCTGAACAGAGCGTGGCCAAAGCCAGCCTGTGCACAGATTCCAGGATGGAGGCCGGGATCGTGGAGTCTCCTCTGCAGAGAAAGCTCTCCAGGCAGGCAGGACTGCCGGGCACCGTGTGGGGGCGCCTCCCCGGGGGGCACCCCGCACCACCCCACCCACGTGGCAGTCCTGAGGACGGGTCAGATAAACTCACCCGCCCCCGGGCTCGGGAGCGCAGCGCCTCCCCAAGGGTTCTGCAGGAAGACCCGGGCCGGCCCGTGGGGAGCTCAGCCCCCCGCTACAGGTGCGTGTGCGGCAAGGCGTTCAGATACAACTCGCTGCTGCTCAGGCACCAGGTCATCCACACCGGCGCCAAGCCCTTCCAGTGCACGGAGTGCGGGAAGGCCTTCAAGCAGAGCTCCATCCTGCTGCGGCACCAGCTGATCCACACCGAGGAGAAGCCGTTCCAGTGCGGCGAGTGCGGCAAGGCCTTCCGGCAGAGCACGCAGCTGGCTGCCCACCACCGCGTCCACACCCGCGAGCGGCCCTACGTGTGCGGCGAGTGCGGCAAGGCCTTCAGTCGCAGCTCCCGGCTGCTGCAGCACCAGAAGTTCCACACCGGGGAGAAGCCCTTCGCGTGCACCGAGTGCGGCAAGGCGTTCTGCCGCAGGTTCACCCTCAACGAGCACGGCCGCATCCACAGCGGGGAGCGGCCCTACCGGTGCCTGCGGTGTGGGCAGCGCTTCATCCGAGGGTCCTCGCTCCTGAAGCACCACCGGCTGCACGCCGAGGAGGGCCCTCAGGACGGCGGCGCGGGGCAGGGCGCCTTGCTCCGAGCGGCGCAGAGGCCCCAGGTGGGGGACCCGCCCCACGAGTGCCCGGTGTGCGGGAGGCCGTTCCGACACAACTCTCTGCTGCTCCTGCACCTGCGCCTACACACGGGCGAGAAGCCGTTCGAGTGCGCGGAGTGCGGCAAGGCCTTCGGTCGCAAGTCCAACCTCACTCTGCACCAGAAGATCCACACCAAGGAGAAGCCCTTCGCGTGCACCGAGTGCGGCAAGGCCTTCCGCAGGAGCTACACGCTGAACGAGCACTACCGGCTGCACAGCGGCGAGAGGCCATACCGGTGCCGCGCCTGTGGGAGGGCCTGCAGCCGGCTGTCCACCCTCATCCAGCACCAGAAGGTGCACGGCCGCGAGCGCCAGGAGGACACGGAGGGCAGGCGGGCGCCCCGCTGGGCTTCC


>mmulZNF7 [GenBank:XM_001106092]
ATGGCAGTACGGTCATCCCCAACGCACAGCAGAGCCTCCATCTCCAGGACCTCCGTGCCAGTCCCCCTCTGTGTGTGCGCAGCAGCCACCACTGCTGTTCCCAAAACCTCAGTCCCATGGCTGCTCTGGCGCTCATGGGTCTCTGTATGGCACATTGCCCAGGTGGGGCCCGCTGTCTGCCCCGGCCCTCTGCTCTTGTCCCTCGGGCCCCAGCCTCGTCCCTCCTCGGATCTGACTCCACCCCTTCTCCGGAGCCTGGGAGGCGGGGCTGAGAACCACAGAGATGGCCGGGCCTCCGAGCCGCCCAGGGCGGCCGGAAGTTTGCGGGGCGGGGCGGGGCAGACGCGGGCGGCCAAGGCCCGTTTCCGGCGGCGTCGCGCGTTTGCTATCCTCGGGTGGTCCTCAGGGAGGGTGTCTCGGCCAGAACACGTGGATGCCCACCCACCACTGAGCCTCATGGAGGTGGTAACATTTGGCGATGTGGCTGTGCACTTCTCTCGGGAGGAGTGGCAGTGTCTGGACCCTGGCCAGAGGGCCCTCTACAGGGAAGTGATGCTGGAGAACCACAGCAGTGTGGCTGGACTAGCAGGATTCCTAGTTTTCAAGCCTGAGCTGATCTCCCGGCTGGAGCAGGGAGAGGAGCCGTGGGTCCTTGACCTGCAGGGAGCAGAGGGGACAGAGGCACCAAGGACCTCCAAGACAGATTCTACGATTAGGACTGAAAATGAGCAGTCCTGTGAGGACATGGACATCCTAAAATCGGAATCCTATGGGACAGTCATCAGAATCTCCCCACAGGACTTTCCTCAGAATCCTGGCTTTGGAGACGTTTCTGATTCTGAGGTCTGGTTAGACAGTCATTTGGGCAGTCGTGGGCTGAGAATGACAGGCTCTACCTTTCAGAATAACTGTTTGAATGAGGAGACTGTGGTTCCAAAGACCTTCATCAAGGATGCTGCCCAGGGATGTAAGGAGCTGGGAAGCAGCATCCTGGATTGTCAGCCTCCTGAAAGTCAGAGAGAGAGTGCAGAAGGGACGTCCCAGAGATGCGAGGGGTGTGGGAAAGGCTTCAGAGCCACTTCAGACATTGCTCTGCATTGGGAAATTAATACACAGAAAATTAGCAGATGTCAAGAATGCCAAAAAAAGTTATCTGACTGCTTGCAGGGGAAACATCCAAATAACTGTCATGGAGAGAAGCCGTACGAATGTGCAGAGTGTGGGAAAGTCTTCAGGCTCTGCTCGCAGCTTAATCAGCATCAGAGAATCCACACGGGAGAGAAACCCTTTAAATGCACTGAGTGTGGAAAAGCCTTCCGCCTGAGCTCAAAACTTATTCAGCATCAAAGAATTCACACTGGGGAGAAGCCCTACAGATGTGAGGAATGTGGAAAAGCCTTTGGTCAGAGCTCAAGCCTCATCCACCATCAGAGAATCCACACAGGAGAGAGGCCCTATGGTTGTTGTGAATGTGGGAAAGCCTTCAGCCAGCAGTCGCAGCTGGTTAGACACCAGAGAACTCACACTGGGGAGAGGCCCTACCCTTGCAAGGAGTGTGGGAAGGCCTTCAGCCAGAGCTCCACCCTAGCCCAGCATCAAAGGATGCACACTGGGGAGAAAGCTCAAATTCTAAGAGCCTCAGACAGTCCAAGCCTTGTTGCACATCAGAGAATTCACGCTGTAGAGAAACCATTTAAGTGTGATGAGTGTGGGAAAGCTTTTAGGTGGATCTCTCGCCTGAGTCAGCATCAGCTGATTCACACTGGAGAGAAGCCTTATAAATGCAACAAGTGTACAAAAGCCTTTGGTTGTAGTTCACGACTTATTCGCCATCAGAGAACTCACACTGGAGAAAAACCATTTAAATGTGATGAGTGTGGCAAAGGCTTTGTTCAGGGTTCACACCTTATTCAGCATCAGCGAATCCACACTGGAGAGAAACCCTATGTGTGTAATGACTGTGGAAAAGCCTTCAGTCAGAGTTCTAGCCTTATTTACCATCAGAGAATCCATAAAGGAGAGAAGCCCTATGAATGCCTCCAGTGTGGAAAAGCCTTCAGCATGAGCACACAGCTTACAATACATCAAAGGGTTCACACTGGAGAGAGGCCCTATAAATGTAACGAATGTGGGAAAGCCTTCAGTCAAAACTCAACCCTTTTCCAGCACCAGATAATTCATGCAGGAGTGAAGCCCTATGAGTGCAGTGAATGTGGAAAAGCCTTCAGCCGGAGCTCGTATCTTATTGAACACCAGAGAATACACACTAGGGCCCAGTGGTTTTACGAATATGGGAATGCCCTGGAAGGGTCCACCTTTGTGAGCCGTAAAAAGGTTAATACTATAAAGAAACTGCATCAGTGTGAAGACTGTGAGAAAATATTTAGGTGGCGTTCACACCTAATTATACACCAGAGAATTCACACTGGGGAGAAGCCTTATAAATGCAATGACTGTGGCAAAGCTTTTAATCGGAGCTCACGGCTTACCCAGCATCAAAAAATTCACACAGGATAGACCACTTATGTATAAATGTGTATATATGTGAATAAACCTACAGCCTTAA


>mfasZNF7 [GenBank:AB169247]
GTTTCCGGCGGCGTCGCGCGTTTGCTATCCTCGGGTGGTCCTCAGGGAGGGTGTCTCGGCCAGAACACGTGGATGCCCACCCACCACTGAGCCTCATGGTAGGAAGCTTGACTGCCTCCTCCTCCTCGCTTGTCCTGTGAAGGCCCACCTCCTGCTCTGCCTCGGGGCCTGGGCTCAGACCCTACTTTGGCCCTTGCTGGGCTTAGGAAGACCCCCTTGCCCCCATCCAGTGAGCAAACCCCTGCCCAAACCAGGGCCTAATTGAGGCTCTTGAGGGGGGAGGGTTGTATGACCACCGTGGACTGGGTTCCTCCTCCCCGAGACTACCCCCCCCCAGAATATTAGAGGCAGAGGAGTCCTGGTGAGCGTCTCGCCTAACCCCGTGGTGCGCCTGCCTGAGGCATGTCATGAGGCAGTGCCAGAAGGGGGCTGGCACCCAGGGATTCCTGGGGTGCTGGTGTATGTCCTTTCAGGAGGTGGTAACATTTGGCGATGTGGCTGTGCACTTCTCTCGGGAGGAGTGGCAGTGTCTGGACCCTGGCCAGAGGGCCCTCTACAGGGAAGTGATGCTGGAGAACCACAGCAGTGTGGCTGGACTAGCAGGATTCCTAGTTTTCAAGCCTGAGCTGATCTCCCGGCTGGAGCAGGGAGAGGAGCCGTGGGTCCTTGACCTGCAGGGAGCAGAGGGGACAGAGGCACCAAGGACCTCCAAGACAGATTCTACGATTAGGACTGAAAATGAGCAGTCCTGTGAGGACATGGACATCCTAAAATCGGAATCCTATGGGACAGTCATCAGAATCTCCCCACAGGACTTTCCTCAGAATCCTGGCTTTGGAGACGTTTCTGATTCTGAGGTCTGGTTAGACAGTCATTTGGGCAGTCGTGGGCTGAGAATGACAGGCTCTACCTTTCAGAATAACTGTTTGAATGAGGAGACTGTGGTTCCAAAGACCTTCATCAAGGATGCTGCCCAGGGATGTAAGGAGCTGGGAAGCAGCATCCTGGATTGTCAGCCTCCTGAAAGTCAGAGAGAGAGTGCAGAAGGGACGTCCCAGAGATGCGAGGGGTGTGGGAAAGGCTTCAGAGCCACTTCAGACATTGCTCTGCATTGGGAAATTAATACACAGAAAATTAACAGATGTCAAGAATGCCAAAAAAAGTTATCTGACTGCTTGCAGGGGAAACATCCAAATAACTGTCATGGAGAGAAGCCGTACGAATGTGCAGAGTGTGGGAAAGTCTTCAGGCTCTGCTCGCAGCTTAATCAGCATCAGAGAATCCACACGGGAGAGAAACCCTTTAAATGCACTGAGTGTGGAAAAGCCTTCCGCCTGAGCTCAAAACTTATTCAGCATCAAAGAATTCACACTGGGGAGAAGCCCTACAGATGTGAGGAATGTGGAAAAGCCTTTGGTCAGAGCTCAAGCCTCATCCACCATCAGAGAATCCACACAGGAGAGAGGCCCTATGGTTGTTGTGAATGTGGGAAAGCCTTCAGCCAGCAGTCGCAGCTGGTTAGACACCAGAGAACTCACACTGGGGAGAGGCCCTACCCTTGCAAGGAGTGTGGGAAGGCCTTCAGCCAGAGCTCCACCCTAGCCCAGCATCAAAGGATGCACACTGGGGAGAAAGCTCAAATTCTAAGAGCCTCAGACAATCCAAGCCTTGTTGCACATCAGAGAATTCACGCTGTAGAGAAACCATTTAAGTGTGATGAGTGTGGGAAAGCTTTTAGGTGGATCTCTCGCCTGAGTCAGCATCAGCTGATTCACACTGGAGAGAAGCCTTATAAATGCAACAAGTGTACAAAAGCCTTTGGTTGTAGTTCACGACTTATTCGCCATCAGAGAACTCACACTGGAGAAAAACCATTTAAATGTGATGAATGTGGCAAAGGCTTTGTTCAGGGTTCACACCTTATTCAGCATCAGCGAATCCACACTGGAGAGAAACCCTATGTGTGTAATGACTGTGGAAAAGCCTTCAGTCAGAGTTCTAGCCTTATTTACCATCAGAGAATCCATAAAGGAGAGAAGCCCTATGAATGCCTCCAGTGTGGAAAAGCCTTCAGCATGAGCACACAGCTTACAATACATCAAAGGGTTCACACTGGAGAGAGGCCCTATAAATGTAACGAATGTGGGAAAGCCTTCAGTCAAAACTCAACCCTTTTCCAGCACCAGATAATTCATGCAGGAGTGAAGCCCTATGAGTGCAGTGAATGTGGAAAAGCCTTCAGCCGGAGCTCTTATCTTATTGAACACCAGAGAATACACACTAGGGCCCAGTGGTTTTACGAATATGGGAATGCCCTGGAAGGGTCCACCTTTGTGAGCCGTAAAAAGGTTAATACTATAAAGAAACTGCATCAGTGTGAAGACTGTGAGAAAATATTTAGGTGGCGTTCACACCTAATTATACACCAGAGAATTCACACTGGGGAGAAGCCTTATAAATGCAATGACTGTGGCAAAGCTTTTAATCGGAGCTCACGGCTTACCCAGCATCAAAAAATTCACACGGGATAGACCACTTATGTATAAATGTGTATATATGTGAATAAACCTACAGCCTTAACTTATAAAAAA


>mmulZNF250 [ENSEMBL:ENSMMUT00000016174]
AGCCCTTTGTCTGAGGGTGCTGCGGGATGCCGCTTCTTCGCGCGTGAGGCTGCGGCGTGGACGCCCCACAGGCTCCTTCAATTTCTGTGATCCTCAGAGGCCCCAGGAGACCAGGTGATGGCAGCAGCCAGGCTCCTGCCAGTGCCGGCAGGACCCCAGCCCCTGTCATTCTAGGCCAAATTGACTTTCGAGGATGTGGCTGTGCTCCTCTCCCAGGATGAATGGGACCGCCTGTGTCCTGCTCAGAGGGGCCTCTACAGAAATGTGATGATGGAAACCTATGGAAATGTAGTCTCATTGGGACTTCCAGGATCCAAGCCTGACATAATCTCCCAGCTGGAACGAGGGGAAGATCCCTGGGTCCTGGACAGGAAGGGGGCTAAGAAGAGCCAGGGCCTGTGGAGTGACTACTCAGACAACCTCAAATGTGACCACACTACAGCCTGTACACAAGACAGTTTATCTTGTCCATGGGAATGTGAAACCAAGGGAGAGAATCAAAATACAGACTTGAGTCCGAAGCCATTAATTTCAGAGGAAACAGTGATTCTGGGGAAAACACCCTTGGGGAGGATTGATCAAGAAAATAATGAAACAAAGCGAAGCTTCTTTCTGAGTCCAAACTCTGTTGACCACCGTGAAGTTCAGGGCTTAAGCCAAAGCGTGCCACTCACTCCACACCAGGCAGTGCCTAGTGGAGAGAGGCCCTACATGTGTGTTGAGTGTGGGAAGTGCTTTGGCCGGAGTTCTCACCTCCTTCAGCATCAACGTATCCACACTGGAGAGAAGCCCTATGTGTGCAATGTGTGTGGGAAGGCCTTCAGCCAGAGCTCAGTCCTTAGTAAACACAGGAGAATTCACACAGGTGAGAAGCCCTATGAGTGTAATGAGTGTGGAAAAGCCTTTAGAGTGAGCTCAGATCTTGCTCAGCATCACAAGATACATACGGGAGAGAAGCCTCACGAATGTCTTGAGTGTCGGAAAGCCTTCACTCAGCTCTCACATCTCATTCAGCACCAGCGGATCCATACGGGAGAAAGGCCGTACGTGTGTCCATTGTGTGGGAAAGCCTTCAACCATAGCACTGTCCTGCGGAGCCACCAGAGGGTACACACTGGGGAGAAGCCTCACAGGTGCAATGAGTGTGGGAAAACCTTCAGTGTGAAGAGGACACTGCTGCAGCACCAAAGGATCCACACCGGGGAGAAGCCCTACACGTGCAGCGAGTGTGGGAAGGCCTTCAGCGACCGCTCAGTCCTCATTCAGCACCACAACGTGCACACCGGGGAGAAGCCCTATGAGTGCAGTGAGTGTGGGAAGACCTTCAGCCACCGGTCCACCCTGATGAATCACGAGCGGATCCACACCGAGGAAAAGCCCTATGCATGCTACGAATGCGGGAAGGCCTTCGTTCAGCACTCACACCTGATCCAGCACCAGAGAGTCCACACTGGGGAGAAACCCTATGTGTGTGGTGAATGTGGGCACGCCTTCAGTGCACGCCGGTCTCTGATCCAGCATGAGAGAATCCACACGGGTGAAAAGCCCTTCCAGTGCACAGAATGTGGCAAAGCCTTCAGCCTAAAAGCAACTCTGATTGTGCACCTGAGGACCCACACGGGCGAGAAGCCCTATGAGTGCAATAGCTGTGGGAAGGCCTTCAGCCAGTACTCAGTGCTCATCCAGCACCAGCGAATCCACACAGGCGAGAAGCCCTACGAGTGCGGGGAGTGTGGGCGTGCCTTCAACCAGCACGGCCACCTAATCCAGCACCAGAAAGTGCACAGAAAGTTGTGACCCATGGCTGACACAAGAATCCATTCTCACAGAAACTGCACGTGGAACCACAAGCAGCCTTCAGCCCAAGAGAAGTTTCTGTTAACTCTATAGGAAGCCTTTCTTTGGTGATTCAGTGTCACAAAATAACTCAAAAAAGAAGCACTTAGCATGCTGTTCCTGTGGAAAAACTTCAGAGACTGCCTGTTTTATTTTCCTCAACATCTTGAAGTTATATTGGAGAGTAATCATAACATTGTGGTGAATTTTGGTAAAAACAGCCATAATTTTTTCTTTGACATTAGTTTATTTGAACTAAGGGAATTTAAGGCATAAGAACTATTATCCCAATAAAATCTTACATTCCAAATAAAGTTCTTTTTCTAAGAAC


>mmulZNF16 [ENSEMBL:ENSMMUT00000022613]
AAATAGGGGCGGGACTTCCGGGGGCCAGCCGGCGCTGACTGAGAGGCGTTCGTGCCGCGGTGGTGCCTGCCTTCCAAGCGCGACCTGTTGAGGTCCTAGTCATGCCCAGCCTCAGAACTCGCCGTGAGGAGGCGGAGATGGAGCTCTCAGCTCCAGGACCATCCCCTTGGACCCCTGCAGCCCAGGCCCCTGTGAATGATGCTCCTGCTGTGACCCACCCTGGATCTGCAGCCTGTGGTCCCCCCTGCTGTAGTGATACTGAGCTGGAAGCCATCTGCCCTCACTATCAGCAGCCAGATTGCAACACCAGGACTGAAGACAAGGAGTTTCTTCACAAGGAAGACGTTCATGAAGATATGGAATCACAGACAGAAATATCAGAACACTGTGCTGGTGATGTTTCCCAGGTGCCCGAGCTTGGAGATCTGTGTGATGATGCATCAGAAAGAGACTGGGGAGTCTCTGAAGGCAGGAGGCTGCCACAGTCCCTCTCCCAGGAGGGGGACTTCACACCAGCGGCCCTGGGGCTCCTTAGGGGCGCCTTAGAGGAGAAAGATCTGGCCTGTAATGGTTTTGACAGTTGCTTCAGTCTGAGCCCAAACCTGATGTCATGTCAGGAAATCCCTACAGAAGAGAGGCCACATCCGTATGACATGGATGGCCAAAGTTTCCAGCACTGCGTGGACCTAACTGGTCACGAGGAGGTTCCCACAGCTGAAAGTCCACTCATATGTAATGAATGTGGGAAAACCTTCCAAGGAAATCCTGACCTTATTCAGCATCAAATATTCCACATTGGAGAGGCTTCCTTTATGTGCAATGGTTGTGGGAAAACCTTCAGCCAGAACTCAGTTCTTAAAAGCTGTCATCGATCTCATATGAGTGAGAAAGCCTGCCAGTGCAGCGAATGTGGGAAAGCCCTCCGAGGGTGCTCAGACTTTTCTAGGCATCAGAGTCACCACAGCAGTGAGAGGCCGTATATGTGTAACGAATGTGGAAAAGCCTTCAGCCAGAACTCAAGCCTTAAAAAGCACCAAAAGTCTCACATGAGTGAGAAGCCCTATGAATGCAATGAATGTGGGAAGGCTTTCAGGCGGAGCTCAAACCTCATCCAACATCAAAGAATCCATTCTGGGGAGAAGCCATACGTGTGCAGTGAGTGTGGGAAGGCCTTCAGGCGAAGTTCAAACCTCATCAAACACCACAGAACTCACACAGGAGAGAAGCCTTTTGAGTGTGGCGAGTGCAGGAAAGCCTTCAGCCAGAGCGCACACCTGAGGAAGCACCAGAGGGTCCACACTGGAGAGAAGCCTTACGAGTGTAATCGAGGCAAGCCCTTCAGTCGGGTCTCCAACCTCATTAACCCCCACAGGGTTCACACTGGAGAGAAACCCTATAAGTGCAGCGACTGCGGGAAAGCATTTAGTCAGAGCTCCAGCCTTATTCAACACCGGAGAACTCACACTGGAGAAAAGCCTCATGTGTGTACCGTGTGCGGAAAAGCCTTTAGTTACAGCTCAGTGCTCCGGAAGCACCAGATAATCCACACAGGAGAGAAGCCGTACAGATGCAGTGTCTGTGGGAAGGCCTTCAGCCACAGCTCAGCCCTCATTCAACACCAGGGCGTGCACACAGGTGACAAGCCCTATGAGTGCCACGAGTGTGGGAAGACCTTTGGTCGCAGCTCCAACCTCATCCTTCACCAGCGAGTCCACACTGGAGAGAAGCCCTATGAATGTACTGAATGTGGAAAAACCTTCAGCCAGAGCTCAACCCTCATTCAGCATCAGAGGATTCATAATGGGCTGAAGCCCCATGAGTGTAGCCAGTGTGGCAAAGCCTTCAACCGAAGCTCCAATCTCATTCACCACCAGAAAGTTCATACTGGGGAAAAACCCTACACCTGCGTTGAATGCGGTAAGGGCTTCAGCCAGAGCTCACACCTCATTCAGCATCAGATAATCCACACTGGCGAGCGCCCCTACAAATGCAGTGAGTGTGGGAAAGCCTTCAGCCAGCGTTCCGTCCTCATCCAGCACCAGAGGATCCACACTGGGGTGAAGCCTTACGACTGCGCTGCTTGTGGGAAAGCCTTCAGCCAGCGATCAAAGTTGATCAAACACCAGTTGATTCACACCAGGGAGTAGCCTGTTGGGCTGGCAGGAGTGAAATTGAGCATAGTCTCCTCTCCAGCTCCACTTTGGCCATTGTCTCAGCCTCTGCCACTTCCCAGACCGAAAGCCTGCACTCAGCTCACTGCCCCACATCCAGTGGCCACATGGCCTGGGACCCTCCTTTGAAACATCCTTTACGGGCTCCCTTCTCACTGCCACTGCTCTTGCCTCTTGTGATGTCTTGAGTTTGGGTTTGTTTTTACAGCTTGTGAACAGACTGCACGTTTGTTACAAGGGAGAAGATTATGGAGGCTACATATTCATGAATAGAGTTTATTATTGTAATCAAAAGTTACCAAAAAAGTAAAGTGACATTTGGTTTGAGAT

>mmulZNF252 [GenBank:XM_001096362]
ATTTCCGGCCCAGGGGGTTTGGCTCGCCACGGCACGGGAGTCAGCGTTCGTGCTCCTTCCCTTTCCGTGGTCGAGCTGATTCCTGACCTGAGGGCTGCATCAAGATCTTGTCATTCCACATCATGGTTTCCTTTGAGGATGTGGCTGTACTCCTCTCCCAGGAGGAGTGGGACTGTCTGATCCCTGCTCAGAGGGGCCTCTACAGGGATGTGATGCTGGAGACCTATGGGAACCTTGTCTCACTAGGACTGCAAGCTTCCAAACCTGATGTCATCTCCAGGCTGGAGCAGGGGGACAAACCATGGACCCCACACATCCTGAGAACTCAGATTAGCTGGAGCTGGAGGCACAAGAGAGAATGCTATGATTCTATGATTGAAAAAGAGGAATTGATTCCAAAGCAGGAAATTTCTGAAGAATTGGAATCCCAGAGAGCAAAATCAGAAGATCATGTAAGGAATATTTTTAAGGAAACTGAAGAGATGAGTAAAACTGAGGGAAAGTTAGAGAATTGCTGGAGAAAATACGCAGTAGAAGGAGTTAAAAACTCCTTTTCCCGGAAGAACAATTTCAGAGCAATTACTATGAGATGTGTGAAAACCCTCTCTAGAGAGAATGGCCACAAGTTCAATACTGTTGGAGAAAACTGCATTACAGACTCAAATCTTGACAAACATGTTAGAATGTCTAGAGAGAAGAGTCTCCATCCAAGAGTGTCAAGTGTAGAAAACTTGAAACAACATGAGGACCTCACTAACCTCCAGAGTTTCCAGTTAGGAGAAAGAGCCTGTCAGACAGATGTGTTAGTGAAAGTACCCAGACAGAGTTCAGTTCTTAGTGAGAATCAGAGGATGAACAATCCAGACAGACCATTTGAGCGTACTGGGCATGGAAAAACTTTCAATCAGAACACAGCTTTTAACCAGCACCAGAGAATTCATAGTGGAGAGAAGCCCTATGAGTGCAATGAATGTGGAAAAGCTTTTAGTCGGCCCTCTATCCTCAGTAAACATCAGAGAATCCACAATGGTAAGAAACCCTACACATGTGAGGATTGTGGCAAATCTTTCAGTGCTCACTCATACTTTATTCAGCACTGTAAAATTCACACTGGACAAAAGCCCTATGAGTGTATTAAATGTGGGAAAGCTTTTAGTACACATTCATCTTATATTCGACATCTAAAAATTCATACAGGAGAGAAACCTCATGAGAGTAATCAGTGTGGAAAAGCCTTTAGTCATAGCTCTAATCTAATTCATCATCAGAGAATTCATAGTGGAGAGAAACCTTACAAGTGCAGAGAATGCGGGAAAGCCTTCAACAGAAAATCACACCTTATTCAACATCAGAGAATTCATTCTGGAGAGAAACCTTATGACTGTGAGGAGTGTGGCAAAGCCTTCAGTACACGATTATCTCTCATTCAGCATCAGAGAGTCCATACAGGAGAAAAGCCCTATGAATGTAATGAGTGTGGAAAATCCTTTAGCCTGAACCGAACTCTTACTGTCCATCAGAGAATTCACACTGGAGAGAAACCTTATAGGTGTAATGAATGTGGGAAATCCTTTAGTCAACGCTCACAAGTTATTCAACATAAGAGAATTCATACTGGAGAGAAACCTTATATCAGCAATGAGTGTGGAAAATCATTTGGTGCTCGTCTATCCCTTATCCAGCATCAGATAGTTCACACTGGAGAGAAACCTTATGGTTGTAGTGTGTGTGGGAAAACCTTTAGTCAAAAGGGACATCTTATTCAGCATCAGCGAATTCACACAGGAGAGAAACCGCATGAATGTAGTGAGTGTGGAAAAGCTTTCAGCCAGAGTTTTAATCTTATTCACCATCAAAGAACACACAATGGCGAGAAGCCCTATGAATGTAATGAATGTGATAAAGCCTTCAGTGTGCTTTCTTCCCTTGTTCAGCATCAGAGAATACATAATGGAAAGAAACCCTACGAGTGTCACAAATGTGGGAAGGCCTTTAGCCAGGGGTCACACCTTATTCAACATCAGAGGAGTCACACTGGTGAGAAACCCTATGAGTGTAATGAGTGTGGGAAAACCTTTGGACAGATATCCACCCTAATTAAGCATAAGAGAACACACAATGGAGAGAAGCCCTATGAGTGCAGTGACTGTGGGAAGGCCTTCAGCCAGAGTGCACACCTTATCCGCCATCGAAGAATTCACACTGGAGAGAATCCCTATGAGTGCAGTGACTGTGGGAAGGCCTTCAATATTCGCTCCTCTCTCATTCAGCATCAGAGAATTCATACTGGTGAGAAACCTTATGAATGTAGTGAGTGTGGCAAGGCATTCAGTCAGCATTCACAATTTATCCAACATCAGAGAATTCACACTGGAGAGAAACCCTATGTATGCACTGAATGTGGAAAATCCTTCCGACAAAGGTCTCACCTTACTCGACATCAGAGAATTCACAGTGGGGAGCGACCTTAA


cDNA sequences of dog (Canis familiaris) ZNF genes from the chromosomal region syntenic to human 8q24.3:


>cfZNF34 [ENSEMBL:ENSCAFT00000002631]
CAGGCTGAGGTGACCTTTGAGGACGTGGCCGTGCTATTCTCCCGGGAGGAGTGGGGCCGTCTGGGCCCTTCTCAAAGGGGCCTCTACAGGGATGTGATGCTGGAGACCTACAGGAATCTGGTCTCCCTGGGAGCTGGACCTGCAGGTCCCAAGCCTGGGGTGATCACGCAGTTGGAGCGAGGGGATGAGCCATGGGACCTGGATGCACAGGGCGCCAAGGGGACAGAGCGACTGAGAGTCAGTGTCTCAGGTCATGGGGCCAGGACTGAATTTAAGGAGTTGTCTTCGGAGGAGATGCTTGATAGGGAAGAACTGGATCAACTCCAGGGGGCTGTTCCCCAGGGACGTGATCCTGAAGAGATCCCTGCATGGAGCAGGGAGCCAGAGGAGAGCCTGGACAAGCATATGGAGCAGAGAGACCTGAGGCCAGTCCCACTGACCAGTGAGGACAGCATTCAGGAGTCTGGGGGAGGCCTCAGGTTTCGGTCAAGCCCTGTCTCTGATCAGAGACCTCACAAATGCGATATATGTGAACAGAGTTTCGAACAGAGATCATACCTCAATAACCACAAGCGTGTACACAGGTCAAAAAAGACAAATATTGTCCATGATTCTGGGGAATTCTTCAGTGCAAATTTAGTTGTTAAAGAAGATCAGAAAATTCCTCTTGGGAAAAAATTACATTATTGTGGTTACTGTGGGAAGGCCTTCAGGTACAGTGCTAATCTTGTCAAACACCAGCGGCTTCATAGTGAAGAGAAGCCCTACAAATGTGATGAGTGTGGGAAAGCCTTCAGCCAGAGCTGCGAGTTCATCAATCACCGAAGGATGCACTCAGGCGAGATCCCCTACCGTTGTGGTGAGTGTGGGAAGACATTCAATCAGAGGCCCAACCTCATGAAGCATCAGAGAATTCATACTGGGGAGAAGCCCTACAAGTGTGGTGATTGTGGGAAACACTTCAGCGCCTATTCTTCCCTTATATATCACCAGAGAATCCACACTGGAGAGAAACCCTATAAGTGTAACGACTGTGGGAAAGCCTTCAGTGATGGCTCAATCCTTATCCGACATCGCCGGACCCACACTGGGGAGAAGCCGTTTGAGTGTAAAGAATGTGGCAAAGGCTTTACCCAAAGTTCTAACCTTATCCAACATCAAAGAATTCACACTGGTGAGAAACCCTATAAATGTAATGAATGTGAGAAAGCCTTCATCCAAAAAACCAAACTTGTTGAACATCAGAGAAGCCACACTGGAGAGAAGCCCTATGAATGTAATGACTGTGGCAAAGTCTTCAGCCAGAGCACACACCTTATCCAGCATCAGAGGATTCACACAGGAGAGAAGCCATACAAGTGTAGTGAGTGTGGGAAGGCCTTCCACAACAGTTCCAGACTCATCCATCACCAGAGGTCTCACCACGGAGAGAAGCCGTACAAATGCAGTGATTGCAAGAAAGCCTTTAGCCAGGGCACTTATCTCATCCAGCACCGGAGGATCCACACTGGCGAGAAGCCCTACAAATGCAGCAAGTGTGGGAAGGCCTTCCGGCACAGTTCGAACATGTGCCAGCATCAGAGGATTCACCTCCGGGAAGACTTCTCG


>cfZNF517 [GenBank:XM_539227]
ATGTTGCTAGACCGGCAGCATCTCAAGGGTGCCTCCTCCCCAGACCCCTGTAGGCTGTGGAGAGACAGCCCCAGACAGCAGGGTTTGTGGTTGCAGGAGGCCGTGGTGTTTGAGGATGTGGCCGTGTACTTCACAAGGATAGAGTGGAGTTGCCTGGCCCCCGACCAGCGGGCACTGTACAGGGATGTGATGCTGGAGAACTACGGGCACGTGGCCTCGCTGGGCTTTCTTGTTGCCAAACCAGCACTGATCTCCCTCTTGGAGCAAGGGGAGGAGCCGGGGGCTTTGATTCTGCAGGTGACTGAGGAGCGAGGGTCCGCAACCAGCCGCTGCCCAGATTCTAGGATGGAGGCTGGGATCAAGCAATCTCCTCTGAGGAGAGTGTCCTCTAAGCAGTTAGGACTATTGGGCACGATTTGGGGCCGCCTCCCTGCGGGGAGGCCCAAGTTAACTGAATTGAATGGCAGTCCTGAGGATGGGTTAGATAAAGTACCCCTCCCCCTCCAGGCAGGCGGCCCTGGTGGGGTACTCAGCACGTCCCTGGGGGTCCTGGAGGACAAACAGCGAGCCTCGGGCCGTGCAGGCACTGTTGGGCAGAGAGTGTACAGGTGTGCTTGTGGCAAGGCGTTTAAGTACAACTCGCTGCTGCTCAGGCACCAGGTCATCCACACGGGCGCCAAGCCCTACCAGTGCACTGAGTGCGGCAAGGCCTTCAAGCAGAGCTCCATTCTCCTGAGACACCAGCTGATCCACACCGAGGAGAAGCCCTACCAGTGCAGTGAGTGTGGCAAGGCCTTCCGGCAGAGCACACAGTTGACTGCGCACCACCGAGTCCACACTCGGGAGAAGCCCTACAAGTGTGGGGAGTGCGGGAAGGCCTTTGGCCGCAGCTCCCGGCTCCGGCAGCACCAGAAGTTCCACACAGGGGAGAAGCCCTACGAGTGCGGGGAGTGCGGGAAGGCCTTCTGCCGCAGGTTCACACTCAATGAGCACTGCCGCATCCACAGCGGGGAGAGGCCTTACACCTGCCTGCAGTGTGGGCAGCGCTTCATCCGTGGGTCCTCGCTCCTCAAACACCACAGGCTGCATGCCAGGGAGAGCCCCCGAGACGACAGCGGCTGTCCGAATACCCTGCTCGGCGCAGCGCAGAAAGCTGCTGCAGGGGACAAGCTGTACCAGTGCTCCGTATGCCAGAGGCTCTTCAAGCACAACTCCTTGCTCCTCCTGCACCAGAGGCTGCATACGGGCGAGAAGCCCTTCGAGTGCAGGGAATGTGGCAAAGCCTTCAGCCGGAAGTCCAACCTCACTCTGCACCAAAAGACCCACACCAAGGAGAAGCCATTTGCCTGCACGGAATGTGGCAAGGCTTTCCGCAGGAGCTACACACTGAATGAGCACTACCGGCTGCACAGCGGTGAGAGGCCCTACAGGTGCCGGGCCTGTGGGAGGGCCTGCAGCCGGCTGTCTGCCCTCATTCAGCACCAGAAGGTCCATGGCCCAGAGTGCTCCCGGGAGGGTGGGGAACACAGACGAGTTGGTAAAAGTCAGAGACTTGGGTGTCTGACTCTTGATTTCGGCTCAGGTCATGATCTTGGAGTGTGAGATTGAGCCCCGATCTCACGTGGAGTCTCCTGGGCATGGAGTCTGCTTGAGATTCTCTCTCCCACTTTCCCTCTGCCCCCCCCTCCCATCCCCAAACAACAGAAAAGGCAGAGAACCCATTGGGGAGAGGCCCCAAGGCAGAGGGCCCGGGGCCAGGGCTGTGGTCAGGCAGCACGCAGGGTGAGGACGGTGAGCCAGAGGGGTCCTCCCGCAGCTGGGGAGCCTGGATGCACACACATGCTCCAATGAGTCACATGAGGATTGTGGGTGGGTTCTGTTTTCTTTTTCCACTCATTTGTTTTCCAAACACTGACTTGTGATCAGAACAAAACAAACAAAAAGGC


>cfZNF7 [GenBank:XM_532359]
ATGCGAGGTTCTCCCCGTGTCCTCAAGTTCCTGGAGTCCACGGGGAGCGTCCCTCCGCCAGGACACATGGATGCCAACCCACCGAGCCTCATGGAGGCTGTGACGTTTGGTGACGTGGCCGTACACTTCTCAAGGGAGGAGTGGCAGTGCCTGGACCCTGGCCAGAGGGCACTGTACAAGGAGGTGATGCTGGAGAACCACAGCAGCGTGGCTGGACTAGCAGGATTCCTGGTCTTCAAGCCTGAGCTGATCTCCCGGCTGGAGCAGGGGCAGGAGCCATGGGTCCTCGACCTGAAGGGAGTCGAAGGGAGAGAGGGGGCAAGGACCTTCCTTACAGATTCTGCAGGTGGGATTGCGAGCGAGCAGGCCGGTGAGGACGTGGATGTTCTAAAATCAGAACCCTGTGCGGCAATGGTCAGAAGCCCCCCACCGGCTTTTCCTCAGAGTTCTAGCTTTAGCGATCCCTCTGATGGTGCAGTCTGGTCAGAGAGTAAGCCAGGCTCTCTCCAGAGAAACCGCCTGAGCACAGGGACTGTGGCTCCCAGGAAGACCTTCACAAGGGAGGGTGCCCAGGGATGTGGTGAGCTGGAGAGCAGTGGTGGCCTGGGTTGTCAGCCTGGCGAAAGTCCAGGAGGTGCAGAAGGGACGTCCCGAAGGTGTGACGTGTGTGGCCGAAGCTTCCGGTCTGCTTCAGACATTGCACTGCGTCGAGAAATTGATACACGAAAGAAACCAAACACATGTCCGGAGTGTAAAACAAAAGTACCCGATTGTTTGCAGGGGAAGCCTCGAGGTAACTGCCACGGAGAGAAGCCGTATGAATGTGAGGAGTGTGGGAAGGTCTTCAGGTTGTGCTCACAGCTTAATCAGCATCAGAGGATCCACACTGGTGAGAAGCCATTCAAATGCATCGAGTGTGGAAAAGCCTTCCGTCTGAGTTCAAAACTTATTCAGCATCAAAGAATTCACACTGGAGAGAAGCCCTACCGGTGTGAGGAGTGTGGAAAGGCCTTTGGTCAGAGCTCCAGCCTCATCCACCACCAGAGGGTCCACACGGGGGAGAGGCCCTATGGCTGCCGGGAGTGTGGAAAGGCCTTCAGCCAGCAGTCTCAGCTGGTCAGACACCAGCGGACCCACACTGGAGAGAGGCCCTACCAGTGCCAGGAGTGCGGGAAGGCCTTCAGCCAGAGCTCAACCCTGGCCCAGCACCAGCGGATGCACGCTGGGGACAAACCTCAACTTCCAAGGAACCCAGATAGCCCCAGCCTTGTTGCACATCAGAGAATACACGCTACAGAGAAGCCATTTAAGTGTGACGAGTGTGGGAAGGCCTTCAGGTGGGTGTCTCGCCTGAGTCAGCACCAGCTGACCCACACCGGAGAGAAACCTTATAAATGCAACAAGTGTGCAAAAGCCTTTGGTTGTAGCTCACGGCTTATTCGCCACCAGAGAACTCACACTGGAGAAAAGCCATTTAAATGTGAGGAGTGCGGGAAAGGCTTTGTCCAGGGCTCCCACCTAATTCAGCACCAGAGAATTCATACTGGGGAGAAACCCTATGAGTGTAGTGACTGTGGAAAGGCCTTCAGCCAGAGCTCCAGTCTCATTTACCATCAGAGAATCCATAAGGGAGAGAAGCCGTACGAGTGCCTGGAATGCGGAAAAGCTTTCAGTATGAGCACACAGCTCACGATCCATCAAAGGGTCCACACTGGGGAGAGACCCTACAAGTGTACCGAGTGCGGGAAAGCATTCAGTCAAAACTCAACCCTTTTCCAGCACCAGATCATCCACGCAGGAGTGAAGCCCTATGGGTGCAGTGAGTGTGGGAAGGCCTTCAGTCGGAGCTCCTATCTCATCGAGCACCAGAGGATTCACACTCGTGCTCAGTGGTATCATGAATACGGGAATACCTTGGAAGCTTCTACCCATGTGAGCCGTAAAAAAGTCAGCACTGTAAAGAAACTGCATAAATGTAACGAATGTGAGAAAATATTCAGGTGGCGCTCGCATCTAATCATACATCAGAGAATTCACACCGGAGAGAAACCTTACAAATGTAATGAATGTGGCAAAGCTTTTAATCGGAGCTCCAGGCTTACTCAGCATCAAAAAATTCACATGGGATAG


>cfZNF250 [ENSEMBL:ENSCAFT00000002635]
ATGGCAGCGGCCAGGCTCCTGCCACCACCGGCAGGACCCCAGCCCCTGTCATTCCAGGCCAAGGTGACCTTTGAGGATGTGGCTGTGCTCCTCTCCCAGGAGGAATGGGACCGCCTGGGCCCCGCTCAGCGCGGCCTCTACCGACACGTGATGATGGAGACGTATGGGAACGTGGTGTCACTGGGACTTCCAGGATCAAAGCCCAACGTGATCTGCCAGCTGGAACGAGGAGAAGAGCCATGGGTCCTGGATGGGCAGGGGACTAAGGAGACTGGGGGCCTGGGGAGTGGCCATTCAGACAACTACAGACACGACCACATGCCAGCTTGTATGGGACAAGACAGCTCACCCTGTCCGTGGGAATGTGAAAACCAGGGAGAGAACCAAGAAAGGGACTTGGGCGTGAAGCCAGGTGTCTCCGAGGACGGCTCCGTGGTCCCGGGGGAAGGCCGCCCGGGATGGCGCGACCAGGGGCGCCCGCGCAACGCTCCGCCACCTCGGGCCCCGGCTGCTGCTGGCGCCGAGCGGCCCTACAAGTGCACCGAGTGCGGCAAATGCTTCGGCCGCAGCTCGCACCTCCTGCAGCACCAGAGGACCCACACCGGCGAGAAGCCGTACGTGTGCGGCGTGTGCGGGAAGGCCTTCAGCCAGAGCTCCGTGCTCAGCAAACACCGGCGCATCCACACGGGCGAGAAGCCGTACGAGTGCAACGAGTGCGGGAAGGCCTTCCGCGTGAGCTCGGACCTGGCGCAGCACCACAAGATCCACACGGGCGAGAAGCCGCACGAGTGCCTGGAGTGCCGCAAGGCCTTCACGCAGCTCTCGCACCTCATCCAGCACCAGCGCATCCACACGGGCGAGCGGCCGTACGTGTGCGCGCTGTGCGGGAAGGCCTTCAACCACAGCACCGTGCTGCGCAGCCACCAGCGCGTGCACACCGGCGAGAAGCCGCACGAGTGCGCGCAGTGCGGCCGTGCCTTCAGCGTCAAGAGGACGCTGCTGCAGCACCAGCGCGTGCACACGGGCGAGAAGCCGTACACCTGCAGCGAGTGCGGCCGCGCCTTCAGCGACCGCTCGGTGCTCATCCAGCACCACAACGTGCACACGGGCGAGAAGCCGTATGAGTGTGGCGAGTGCGGCAAGGCCTTCAGCCACCGCTCGACGCTCATGAACCACGAGCGCATCCACACGGAGGAGAAGCCGTACGGCTGCTACGCGTGCGGCAAGGCCTTCGTGCAGCACTCGCACCTGACGCAGCACCAGCGCGTGCACACCGGCGAGAAGCCGTATGTGTGCGGCGAGTGCGGCCACGCCTTCAGCGCCCGCAGGTCGCTGGTGCAGCACGAGCGCATCCACACGGGCGAGCGGCCCTTCCGCTGCGCGCAGTGCGGCAAGGCCTTCAGCCTCAAGGCCACGCTGATCGTGCACCTGCGGACGCACACCGGGGAGCGGCCGTACGAGTGCAGCCGCTGCGGCAAGGCCTTCAGCCAGTACTCGGTGCTCATCCAGCACCAGCGCATCCACACGGGCGAGCGGCCCTACGAGTGCGGTGAGTGCGGCCGCGCCTTCAACCAGCACGGGCACCTGATCCAGCACCAGAAGGTGCACCGGAAGCTGTGA


>cfZNF16 [GenBank:XM_532361alt]
ATGCACCTGCCGGCGCCTGCAGTCCTCGGGCTCAGTGTGCCCCGGGCAGCCGCACGGAGCAGCGCCGCCGCCGCCCGGAAGTCGGGGGAGGGATTTCCGGCCCCGCCTCTGGGCCGAGCCGGCAGTCCTACGGCGGCGCGCGGCGACCGGAGCGACCCACGGGCGGGCCGCGGGGCGCGCGCCTTCCGGCCGACCGCCCCAGTCCCCGAGGCCCCAGGGCTTGCGCGGGAACCTTGCGTCCGCGTCGGCAACGGTTCCGGTAATGACACGGGCCACGCCGGAACTCCCTCTGCGGGGTCCCCGCCGCCGACTGTGCCGTATGCCCGGGTTGGAGGGCTTCGCAGCTGCACACGGAGGTCCAAGGCGTTCAGGTTACCGCAGGTTCGTGACGGTGAGGATGATTTCAAGAATTTTAAAATAAAGCGAGTGAATATTGCAAATGTTCATTTGTGTGGTGAAGACCCCCGGCCCGCGGAAGCAACCCCTGCTCTGTCCTGCTGCTACCGGCCAGGCACTTTAGTCCGCGTTCTCCTTGCTTTGACGATTGTCCGCACCATTCCAGGGAGCACCGGTTTGTTTGTCCTGATACAGAAACGTGGCCCCTGTGCCACCGTCAAGCAGCTACGGCCTCGTCTGTACCCCACGACCCTGCTTTTGAGAATGCTCTTCAGTGACAACCCCCAGGCCTGTGTGAGTGATGCTCCTGCTGTGACCCATGCTGGATCTACACTCCGTGATCCCCACTGCTGTGGCTACACTGAGCCAGGAACCACCCCCCCTCACCATCAGCAGCCAGATTGGGACACCAGGACTAAGAGCAAGGAATTTCTTCAGAAGAAAGAAGTTTCTGAGGATTTGGAATCCCAGGAAGAAATATCAGAAAACTATACCAGTGATTTTTCCCAGGTGCCTGAGCTTGGAGAACTCTGTGATGATGTACTAGAGAGGGATTGGGGAGCCCCTGACAGTAAGAGACGAGTGCAGTCACTCTACCAGAAGGGGGGCTTCACTCCAATGGCAGTGCTCCTTAGGAGCCCCTTAGAGAAAGAGCTGGGCTGTGATGACTTTGTAAGAAGCTTCAGTCTGAGCCCAAACCCAACGGCATCTCAGGGAATTCCTACAGAAGAGAGGCCACATCTGTATGACATGTGTGGCCACAGCTTCCAGCACAGTATGGACTTAGGTAGCCATGAGGAGCATCACATAGCTGAAAGCCCACTCATATGTAATGATTGTGGGAAAACCTTCAGAGGAAACCCTGATCTTATTCAGCATCAGATAATCCATACTGGACAGAAGTCCTTTGTATGCAATGAATGTGGAAAATCCTTCAGCCAGAACTCATTCCTTAAAAATCATCAGAGGTCTCATGTGAGTGAAAAACCCTATCAGTGCAGTGAATGCAGGAAAACCTTCAGTGTGCACTCAAACCTCATTAGGCATCAGATTAATCACAGCGGAGAGAAGCCTTATGTGTGCAGTGAATGCGGAAAAGCCTTCAGCCAGAACTCAAGCCTTAAGAAGCACCAGAAGTCTCACATGAGTGAGAAGCCCTATGAGTGCAGTGAATGTGGGAAGGCCTTCAGGCGGAGCTCAAACCTCATCCAACATCAAAGAATTCATTCTGGAGAGAAGCCGTATGTGTGCAACGAATGTGGGAAGGCCTTTAGGCGGAGCTCAAATCTCATTAAACACCACAGGACTCACACTGGTGAGAAGCCTTTTCAGTGTAATGAGTGTGGGAAAGCATTCAGCCAGAGCTCACATCTGAGGAAGCATCAGAGAGTTCACACTGGGGAGAGACCTTATGAGTGTAATGAGTGTGGCAAACCATTCAGCCGGGTCTCCAACCTCATTAAGCATCATAGGGTTCACACTGGAGAGAAACCCTATAAGTGCAGTGACTGCGGGAAGGCCTTCAGTCAGAGTTCAAGCCTCATTCAGCATCGGAGAATTCACACTGGAGAAAAGCCTCATGTATGTAATGTGTGTGGAAAAGCCTTTAGTTACAGCTCAGTGCTCAGAAAGCACCAGATAATCCACACAGGAGAGAAGCCGTATGAATGTAGCATCTGTGGGAAGGCCTTCAGCCACAGCTCAGCCCTCATTCAGCATCAGGGTGTGCACACAGGTGACAAGCCATATGAGTGTCGCGAATGTGGAAAAACATTTGGTCGGAGCTCCAACCTTATCCTTCACCAGCGAGTTCACACCGGAGAGAAACCCTACGAATGTACTGAATGTGGAAAAACCTTCAGCCAGAGTTCAACTCTCATTCAGCATCAGAGAATCCATAATGGATTGAAACCCCATGAATGTAGCCAGTGTGGTAAAGCCTTCAACCGAAGCTCAAACCTCATTCACCACCAGAAAGTTCACACTGGAGAAAAGCCGTACACGTGTGTAGAATGTGGTAAGGGCTTCAGCCAGAGTTCACACCTTATTCAACATCAGATAATCCACACTGGTGAAAGGCCGTATAAGTGCAGTGAGTGCGGGAAGGCCTTCAGTCAGCGTTCAGTCCTCATCCAGCACCAGAGGATCCACACCGGTGTGAAGCCCTATGACTGCTCTGCATGTGGGAAAGCCTTCAGCCAGCGGTCAAAAGCCCTGCCCTCTGCATACCCCTCCTGTTCTCACAACCTC


>cfZNF252 [RefSeq:NM_001002954]
CAACTTCCGGCCCGGCCGCTTCGGGCTCCCGGGTCCGGCGTCCGCGAGGCGCACCTGCCCCGAGTCCGAGCCGAACCTCTTGAGACCCAGGCATGGAAGTCTCAGTGTTGCTGTGAAAAGCAGGCTGGTCAAATTCCTTTACAGTCTTCCCTCCACCTGCCATTTCACCCACCTCCTTGGGATGAGCTAAGAGATCCAATGATGGCCGTCAAGCAGCTCCTTCCTGCTGGGTCCCAGGTTTTGGTTTCCTTTGAAGACGTGGCTGTGCTACTCTCCCGGGAGGAGTGGGGCCGTCTGGGCCCTGCTCAAAGGGGCCTCTACAGTGATGTGATGCTGGAGACCTACAGGAACCTGATCTCACTGGGACTTCAAGGTTCGAAACCTGATGTCATCTCCAGGCTAGAGAAGGGGGAAGAGCCATGGGCCCCATACTCAGCGAAAATTGAGGAGAGCTGGATCCGGAGTCATGAGAGTGAAAGTTTTCAGTCTCTGATGGAAAAGAAAGGATTGACTCCAAAACAGGAAATTTCCAAAGCAATGGGGTTCCGGAGAGCAAAGTCAGAATATGTAAGGAATGTTTCCAAGGAATCTGAGTTTGAAGAGATGAATAAAACCAAGGGAAAGTTAAAGAATTACAGGAAAAAATCTGCAGAGGAAGAACTTAAGAAATCCTTCTCCCAGAAGAACAGTTCCAGGCCAGTGACGTTGACACATGTGAAATCCCCTGTTTCAGGAAAAGGCCAAAAATCCAGTTCTCTGGAGGTAGACTACACTGTAGATGCAAGCCCTGTCAGATTTCATAGAGCATCTACAGGGGGTAGTCTCCACCAGAATGTGCCATGTGTAAATGACTTTCAACAAAGTCAGGACCTCATTAATCTCCAGTGTTTGCATCTAGGAGAAAGAGCTTGTCAGACAGATCTGTTCATGAAAGCACCCAGGCAGAGTTCAGTTCTCAGTGAGAATCAGAGGGTTAACAATCCAGAAAAATCCTTTGAATGTACTGAGTGTAGAAGACTTTTCAGCCCAAGCAAAGCTCTGTCTCAGCATCAGAGAAGTCACACTGGAGAGATACCCTGTGAGAGTGGTGGATGTGGAAGAACTTCCCACCACTGCTCTGTCCTCAGCCAACATCAGGAAGTTCACCATGGAGGGGAGTCCCATACCTGTGCTGAGTGTGGCAAAGCTTTCAAGGCCCATTCCTACTTCATTCAGCAGCATAACACTCACACAGGAGAAAGGCCTTATGAGTGCAGTGAGTGTGCACATTTATCTTACAGTCAACATCTTCAAATTCATAGTGGGCAGAAACCTCATGAGTGTAGTCAGTGTGGAAAAGCCTTTAGTCATAGTTCTAACCTGTTTCATCATCAGAGAATTCATAGTGGAGAGAAACCATACGAGTGTAAGGAATGTGGGAAGGCGTTCGGTAGGCACTCGCACCTCCTTCAGCATAAGAGAATTCATTCTGGAGAGAAACCTTATGACTGTACTGAGTGCGGCAAAGCCTTCAGTGCACGGTTATCTCTCATTCAGCATCAGAGAACTCATACAGGAGAAAAACCCTATGAATGCAATGAATGTGGGAAATCCTTTAGCCTGAACCGAACCCTTATTGTTCATCAGAGGATTCACACTGGAGAGAAACCCTATAGGTGTAATGAATGTGGGAAATCCTTCAGTCAGCGTGCACAAGTCATTCAGCACAAGAGAATTCACACTGGAGAGAAGCCCTATGTCTGCAATGAGTGTGGAAAGTCATTCAGTGCTCGCTTGTCCCTCATCCAGCATCAGAGAATTCACACTGGAGAAAAACCTTATGGATGCAGTGAGTGTGGGAAAACGTTCAGTCAAAAGGGACATCTGATTCAGCATCAACGAATTCACACTGGAGAGAAACCCTATGAGTGTAATGAGTGTGGAAAAGCCTTCAGCCAGAGTTTTAATCTTATTCATCATCAAAGGACACACAATGGTGAGAAGCCCTATGAATGTAATGAATGTGATAAAGCCTTCAGTGTGCTCTCTTCCCTTGTTCAACATCAGAGGGTCCATAATGGTGAGAAGCCCTATGAGTGTCACAAATGTGGGAAGGCCTTTAGCCAGGGCTCGCACCTCATTCAGCATCAGAGGAGCCACACTGGAGAGAAACCCTATGAGTGTAATGAGTGTGGGAAAACCTTTGGGCAGATATCCACCCTAATTAAGCATGAGAGAACACACAATGGAGAGAAACCCTATGAGTGTGGTGACTGTGGGAAAGCCTTCAGCCAGAGTGCACACCTCGTCCGCCATCGAAGGATTCACACTGGAGAGAATCCCTATGAGTGCAGTGACTGTGGGAAAGCCTTCAATGTCCGTTCCTCTCTTGTTCAGCATCACAGAATTCATACAGGGGAGAAGCCTTATGAATGCGAGAAGTGTGGCAAGGCCTTCAGTCAGCATTCACAATTTATTCAGCATCAGAGGATTCACACTGGAGAGAAGCCCTATATTTGCAATGAATGTGAGAAAGCCTTCAGTGCACGTTTATCCCTTATCCAACACAAGAGAATTCACACAGGGGAGAAACCTTACAAATGCACTGAATGTGGAAAATCCTTCCGACAAAGCTCTCACCTTATTCGACATCAGAGAGTTCACAGTGGAGAGCGACCTTATATGTGTAATGAATGTGGGAAAACTTTTAGCCAGAGAATAACTCTTACTAGTCATGAGAAAACTCACACCAGAGAGCAAGCCTATAAGTGTGTTAAACGTGAGGACCTCTTAACTGCACAGTCAGCTTCCATTCAGCACCATAAAGTTCACAATGGAGAATAACATCTTTATTCAATAAAGTCTTCACTATTTTACATCAGGAGCAGTATACTGATGGGAAGCCATCACTTTTACAGCTTTCATTTTGGAGATTTATCTCTTAAGTGATTTAACAGTCTCCCAGTGCACTGTTAACTAAAAGCAAGATGCCAAAATATGTGGCAAAAATTCCAAGGTCACATGCCATCCCTTAGATTCAGAGGGTGGATCTATGCTTGAGTATTTTAAAGGATCACAATTTAATTACACTTACTCAAGATTGTGTTTTCATAGCACCAGACTTTAA


cDNA sequences of bovine (Bos taurus) ZNF genes from the chromosomal region syntenic to human 8q24.3:


>btZNF34 [RefSeq:NM_001083520]
CCCAGGGCCCTGGTCTGTGCTGGTGAGAGGAGGGTAGCGGCTCTCCCTTGGGAGGTGGGGAAGCAGGCAGAGCCCGCAGCTGCCGGAGGGCTGACGGCTGGGTCTGTCTGCTGAGCCCCTTGACCAGTGTGGTCAGGATTTGTTGACTTTCCTGTATAAATGTTTTACTCCAATAAAGAGCTTTAAAAATACAGAGAAGGTAAATATAGCTGTTGTAGCTTTCTTTTAATTACTGTTTCTTTGGTCTGCCTTATGTTTAGCCTTTCTGTGTATTTTATTTTAGGTTATATAGGTGCACTTTGTGTATCTACTTGGGGAGTTTTTTGCATGCAGTAGGTGAGTTTAATCCTTTTGCTTCAGTTACCCATCCATTGGACTTACCTCTGCTCATTTTGTATCTTTTGACTCCTAATCTTTGTCTTTGCTTTTCGCCTTCTGTTCAATTTTTTTAACTTCCCTTTCCTTCTCAGCCCTTAATGTGTTATCTTTATTCTTTCATGTAAAGTGAATAACTCTCCATTTTCCACCTGAATGAGATAACTTTGTGCCTGGGAGGAGGTAGGGGCAGATGATCTGAAATGATGCATGTGTCTGCAGGGAGCAGAGTCCGCTTGGGAGGCCGAGTGCTGAGGCCAACTGTGGGAGGGGCCAGGCAGTGATTGGCTCCTGCATCCATCCATCCCAGGAAGCCCAGGGTACCAGGGGATGGCAGCTTTGGATCTGTGTGCCCTGCCTCAGGCTGAAGTGACCTTCGAGGACGTGGCTGTGTTCCTTTCGCAGGAGGAGTGGGGCCTTCTGGGCCCTGCTCAGAAGGGCCTCTACAGGGAAGTGATGCTGGAGACTTACAGGAACCTGGTATCGCTGGGAGCTGGACTTGCAGGTCCCAAGCCGGAGGTGATTGCACAGTTGGAGCAAGGGGATGAGCTGTGGGTCCTGGACATGCATGGGGCAGAGCAACCAAGCGTTGATGGCTCAGCTCATGGGACCAGGACTGAGAACCAGGAGGTCACTTCAGGAGAAATGCTGTTTGGGAGAGAACTGGACCCACTCAGGGGGAGTGTTCTCCGGGGACCTGAGCCAGGAGAAGTCCATGAGCGTGTCAGGGAGCCAGAGGGGCGCCTGGACAGGCCTGGGGAGCAGAGAGGCCCCAGGCTGGTCACACTGGCCAATGAGGAGTGTGGCCTGGAGTCTGGAGGAAACCTCAGGTCGCGGTCAAGGCCTGTCCCTGATCAAAGACCTCACAAATGTGATATATGTGAACAGAGTTTTGAACAGAGATCATACCTTAATAACCATAAACGTGTACACAGGTGCAAAAAAACAAATATAGTTCACGATTCAGGGGAAATTTTCGCTGCGAATTTAGTTAAAGAAGATCAGAAAATTCCTGTTGGGAAAAGATTGTATTATTGTGGTTGTTGTGGGAAGGCCTTCAGGTACAGTGCTAACCTTGTCAAGCACCAGCGACTGCACAGTGAAGAGAAGCCCTACAAGTGTGAAGAGTGCGGGAAGGCCTTCCACCAGAGCTGTGAGCTCATCAGTCACCGGAGGATGCACTCTGGAGAGATCCCCTACCGTTGCGATGAGTGCGGGAAGACATTCAACCAGAGGCCCAACCTCATGAAGCATCAGAGGATCCACACGGGGGAGAAGCCCTACAAGTGCAGCGAGTGCGGGAAGCACTTCAGTGCCTACTCCTCCCTCATTTACCACCAGAGGATCCACACAGGGGAGAAGCCCTACAAGTGCAGTGACTGCGGGAAGGCCTTCAGTGACGGCTCAATCCTCATCCGGCACCGTCGGACCCATACCGGAGAGAAGCCGTACGAGTGCAAAGAGTGTGGCAAAGGCTTTACCCAGAGCTCCAATCTGATCCAGCATCAAAGAATTCACACTGGGGAAAAGCCCTATAAATGTAATGAATGTGAGAAAGCCTTCATCCAAAAAACCAAGCTTGTTGAACATCAAAGAAGCCACACCGGAGAGAAGCCCTATGAGTGTAATGACTGTGGCAAAGTCTTCAGCCAGAGCACCCACCTCATCCAGCACCAGAGGATCCACACAGGAGAGAAGCCCTACAAGTGCAGTGAGTGTGGGAAGGCTTTCCACAACAGTTCCAGACTCATCCACCATCAGAGGTCACACCACGGAGAGAAGCCATATAAGTGTGCTGATTGCAAGAAGGCCTTCAGCCAGGGCACGTACCTCCTGCAGCACCGGAGGATCCACACCGGGGAGAAGCCTTACACGTGTGGCGAGTGCGGCAAGGCCTTCCGGCACAGCTCCAACATGTCCCAGCACCAGAGGATTCACCTCCGGGAAGACTTCTCCCTGTGACGGTCGGGAGGCCCAAGGTCTGTGGTGCACTTCCCCCACGTGACCACCCTCTCTTTACTTACTGTCCACATGGTGTCACAGGTGAACAGGCACTTCTAATTTTTAAAAACCTCTTTCTTAAAAGCAGTGGATGTTCATTTTAGAGAAATTGAGTGGAAAAAGGTAAGCAAAAGGGAGAAGCCCTGTGGCCTACCCTTAGAAAGAGAACTCACTACTATTGCGATCAGCGTAGGTTGTGTCCAGACACTCAAATAGCATCCACAAGAGAAGTACTGTGGAACTTGTGAGAGCAGCTCCTGTTTCCTCAGTCCTGGAAAACTCATACTGGAGAGTTCTACGTGTTTATGTGGTTTTACACCTTGTAAATTTTAAAACAGCATGTTGTTCATACTTTTTCTATGTCACTTAATAGAATTGATATTGCCTTTTTTATTGTGGTAATAACATTGTGTGTTAGTCACTCAGTCGTGTCTGACTCTTTGTGACCCCATGGGTTGTAGCCCGTCAGGCTCCTCTGTCTATAGGATTCTCCAGGCAAGAATACTGGAGTGGGTTGCCATTTCCTTCTCCAGTAAAAACATTATGTTTACCATTTTAACCACTTTTAAGTGTACTACTCAGTGGTATTGGATACATTCACAGTGTTGTATGACCATCAGCAAATCTGTTTCCAAAACTTTTTTTATTACAAACAGAAACTCTGCACTCCTCCGCTTTTCCTTATCCTCTCTCTTAAGAATTTGCTTACTCTGAAGTACCTCGTGTAAGTGAGGTCACACATTTGCCTTTGTGTCCTGGCTCATTTCACTTAACGTAAGGTCTCTTCATGGCTGAAGCGTACTCCATTGTTTGCAGACACCATGTTTTTTGTTCACCCATCATCTGTTGATGGGTTATGGGTGTTTCTGCTTGTTGACTTTGTGCATAATGCTGCTGTGAACATTGGTGTACAAATCTGTGCTTGTACTTTCCATTCTTCCAATACTTTGCGGGTATAATCCCAGGAATAAAGTTGATGGATCATAAAAAAAAAAAAAAAAAAAAAAAAAAAAAA


>btZNF7 [RefSeq:ENSBTAT00000024564]
CAGGAGGCTGTGACATTTGGTGATGTGGCCGTGCACTTCTCGCGGGAGGAATGGCAGTGTCTGGACCCTGGCCAGAGGGCTCTCTACAAGGAGGTGATGCTTGAGAACCACAGCAGTGTGGCTGGACTAGCAGGATTCCTGGTCTTCAAGCCTGAGCTGATCTCCCGGCTAGAACAGGGGCAGGATCCATGGGTCCTTGACCTGCAAGGAGCAGAGGGGAGAGAGGAAGCCAGGACCACCCGGACAGATTCTACAGTTGGGACTGATGGTGAGCAGGACATGGACAGTTTTAAATCAGAATCCGGGGGGGTCATGGTCAAAACCTTGCCCCAGAACTTTCCTCAGAGTCCTGGCTTTGGAAACACCTCAGATCCTGAAGTCTGTTCACAGAGACAGCCAACCTCCCTCTTCCATAAAAACTTCTTGAACATGGGGACCATGGGTCCCAGGAAGGCCTTCACCGAGGACGAGTTCCAGGGTCATGGTGAGTGGGGAAGCAGTGGCCGCCTGGGTTGTCAGCCTGATCAAAGTCAGTGGTCCTTCGGAAGGTGCGATGTATGTGGCAGGAGTCTGCGATCTCCTTCAGATGCTGCTCTACACCAGGAAGTGAATACCCAGCAGAAACCCAACAGATGCCAAGAGTGCCAAAAACAGTTATCCGATTGCTTCCAGGGGAGACCTCTGAGTACCTTTCCTGGAGAGAAACCATATGAGTGTAGGGAGTGTGGGAAGGTCTTCAGGTTGTGCTCACAGCTTACTCAGCATCAGAGGATCCACACTGGAGAGAAGCCGTTTAAGTGCACCGACTGTGGGAAGGCCTTTCGCCTGAGCTCAAAACTTATTCAGCATCAAAGGATTCACACTGGGGAGAAGCCCTACAGGTGTGAAGAATGTGGAAAAGCCTTTGGGCAGAGCTCCAGCCTCATCCATCATCAGAGGGTCCACACGGGAGAGAGGCCCTATGGCTGCCGGGAGTGCGGGAAGGCCTTCAGCCAGCAGTCTCAGCTGGCCAGGCACCAGAGGACCCACACGGGAGAGCGGCCCTACCCGTGCCGGGAGTGCGGCAAGGCCTTCAGCCAGAGCTCAACCCTAGCTCAGCACCAGCGGATGCACGCTGCAGAGAAGCTTGAGCTCCCGAGAACCCCGGAGAGTCCCAGCCTGGGTGCACGTCAGAGGATGCATGTTCCTGAGAAGCCATTTAAGTGTGATGAGTGTGGGAAGGCTTTCCGGTGGGTCTCCCGCCTGAGTCAGCATCAACTGACACACACTGGAGAGAAACCTTATAAATGCAACAAGTGTTCAAAAGCCTTTGGTTGCAGTTCACGACTTATTCGCCACCAGAGAACTCACACTGGAGAAAAACCGTTTAAGTGCGATGAGTGTGGGAAGGGCTTTGTCCAGGGCTCACACCTCATTCAGCATCAGAGAATTCATACCGGGGAGAAGCCGTATGAGTGCAGTGACTGCGGGAAGGCCTTTAGCCAGAGCTCGAGCCTCATTTACCATCAGAGGATTCATAAGGGGGAGAAGCCCTACGAGTGCCTCGAATGTGGAAAAGCCTTCAGCATGAGCACACAGCTCACAATACACCAGAGGGTGCACACGGGCGAGAGGCCATATAAGTGCAGTGAGTGTGGGAAGGCCTTCAGCCAGAATTCCACCCTTTTCCAGCACCAGATTATCCATGCCGGGGTGAAGCCCTATGGATGCAGCGAGTGCGGGAAGGCCTTCAGCCGGAGCTCATACCTGATCGAGCACCAGAGGATCCACACTCGTGCCCAGTGGTACCGTGAGTACGGGAGCACCCTGGAGGCTTCCACCCACACGAGTCGTAGGAGAGTTAATACTGTAAAGAAACTTCACAAATGTAATGAATGTGAGAAAATATTCAGGTGGCGGTCGCATCTCATTATCCACCAGAGAATTCACACTGGAGAGAAACCTTACAAATGTAATGAATGTGACAAAGCTTTTAATAGGAGCTCAAGGCTTACTCAGCATCAGAAAATTCACATGGGCTAG


>btZNF16 [GenBank:XM_591561alt]
ATGGACAGTGAAGGTGCCGGAGAGCCAAGTCAGACCTGGGTTCCCAGCACCTCGCAGAGGCGTGGGGAGGGCTGGTCCCGGGTCAGGAGTGGAGCCGCCGAGTCACTTCTCCTCCCTCAGCCTAGAGCAGCCACATTGGGTAGGGCTGGCGGGTCCGCAAGCCGGAAGTTGAGGCGGGACTTCCGGAGCACCTCGAGGCCGAGCCGCCTGGGTATCTCTTCGTTGCGCTCTTCCCCGAGCCGCTCTAGGCGGCGGCTGTTCGCGCTGTGGCGTCTTCGAGTTGCTGATCGCTCTACCTATGACTCCGATGCACCGGCGGTGGGTGCTGGCCCCGGGAGGCCTGTCTCGGGTGCGCGTGGGGCCGTGGAATCGTGGGCGGCTTGCAGCCTCGGAGGGCCCCAGACGGGAGGGCGTGTGAGGGACGCCCTGTGGACGCTGCCCGACAGAGCCGAGCAATCCCTGCCCTGGAGCCGGGCGGCGGGACCGCTCGGCGAGGCAGCCGCAGAGCGACCGGCAGGCTGGGACACCAGGACTAAGGGCCAGGAGTTTGTTCAGAAGGAAGAAGTTTCTGAGGACTTGGAATCACAGGTAGAAATATCAGACAGCTCTGGCAGTGATGCTCCCCAGACACCTGAGCTTGGAGGACTCCATTGTGGTGTAGAAGAAAGAGACTGCGGGCTCCCTGAGGATGAGAGGCAGATGCACCGCCCTCACCAGGAGGGAGCCTTCACTCTAGCACAGATGCTTCTCAGGAGCCCCTCAGGAGAGAGAGAGGTGGACTGTGATGACATCAAGAGCAGCTGCAGCTGGAGCCCAAGCCCAGTGGAGTGTCAGGGGCCTCCTGCAGAGGGGAGGCCCCATCCACTTGGCATACGGAGCCTGCCCTGCAGCCTGGACCTGCCCAGCTGCACAGGACTTCACGTGGCCGAAAGCCCATTCATATGCAGCGAGTGTGGGAAAACCTTCGAAGGAGACCCCGGTCTTACTCAGCATCAGACAGGGCACACTGGACACAAGTCCTTCATCTGTAACGAGTGTGGAAGGCTCTTCAGCACACACACAGGCTTCCTCCAGCACCAGCTTACCCACCATGGGGAGAAGCTGCATATGTGTTCTGAGTGTGGGAAGGCCTTCTGCCAGAGCTCCAGCCTTAAAAAGCACCAGAAGTCCCATGTGAGCGAGAAGCCCTACGAATGCAGTGAGTGCGGGAAGACCTTCAGGCGCAGCTCCAACCTCATCCAGCACCAGAGAATCCACTCTGGCGAGAAGCCGTACGTGTGTCATGCCTGTGGGAAGGCCTTCAGGCGCAGTTCCAACCTCGTCAAGCACCAGAGGGTCCACACAGGGGAGAAGCCCTTCGAGTGTACTGAGTGTGGGAGGGCCTTCAGCCAGAGCTCACACATGAGGAAGCACCAGAGGGTCCATACTGGGGAGAGGCCTTACTCATGTAGCGAGTGTGGCAAGCCCTTCAGCCGGGTGTCCAACCTCATCAAACACCACAGGGTCCACACGGGGGAGAAGCCCTACAAGTGCAGTGAGTGTGGGAAGGCCTTCAGCCAGAGCTCGAGCCTCATCCAGCACCGGAGGATCCACACCGGGGAGAAGCCTCATGTGTGTGCTGTGTGTGGCAAGGCCTTCAGCTACAGCTCGGTGCTCCGAAAGCACCAGATCATCCACACGGGGGAGAAGCCGTATGAGTGCGGCGTCTGCGGGAAGGCCTTCAGCCACAGCTCCGCACTCGTGCAGCACCAGGGTGTGCACACGGGAGACAAGCCCTACGAGTGCCGCGAGTGTGGAAAGACCTTTGGCCGCAGCTCCAACCTCATCCTGCACCAGCGGGTCCACACGGGGGAGAAGCCCTATGAATGCACTGAGTGCGGGAAAACCTTCAGCCAGAGCTCAACTCTCATTCAGCATCAGAGGATCCACAATGGGTTGAAACCCCACGAGTGTAACCAGTGTGGCAAAGCCTTCAACCGGAGCTCCAACCTCATCCACCACCAGAAGGTCCACACTGGAGAGAAGCCCTACACCTGCGTCGAGTGTGGGAAGGGCTTCAGCCAGAGCTCGCACCTCATCCAGCACCAGATCATCCACACGGGCGAGAGGCCCTATCAGTGCAGTGAGTGTGGGAAGTCCTTCAGCCAGCGCTCCGTTCTCATCCAGCACCAGCGGATCCACACTGGGGTGAAGCCCTACGACTGCACGGCTTGTGGAAAAGCCTTCAGCCAGCGCTCCCACCTGCTGCAGCACCAGAGGACGCACACCGGGGAGAGGCCCTATGTGTGCGGTGTGTGTGGCAAGGCCTTCAGCCAGAGCTCGGTGCTCAGCAAGCACAAAAGGATCCACACGGGCGAGAAGCCC


cDNA sequences of mouse (Mus musculus) ZNF genes from the chromosomal region syntenic to human 8q24.3:

>mmZNF251/Zfp251 [RefSeq:NM_001007568]
GTTCCGCCCCCAGCCGACAGTCAACTTGCCCCTTCTGCGCGGGCCAGGCTGGGTGTAGGTGGGTGCCTGGTCCAGAGGCTGAGGCGAGGCTCCCGCGTTCCCATCGCTCTGCAGGTGGGCAAGGACAGCCAGCAGGCAGCTCGGGCTGGCCTGCCTCAGCTTCTGGCCAAGGGCGCCGATGATGCCGCGACACCTGGGAGGAGGGATAGTGGAGGAGGCAGCGGTCGCAGCCACAAGGGCCACCCAGCAGCGGGAGAACCCGTGGGAGGGACAAGCCGCTGGCGGCGACGCGGCCTTGGGAGGCATGGATGTGAAGGACGATGACTTTTAAGTTTTTGCCCAGAGCTAGCCCTCGCCGCCTGCGTGTCCACTCACACCCGCAACCCGGTGAGGCCTGTCGCGGCGAGGCCCGTAGCCTTTTCCAGGCTGGCCCTCTAAAGCTGAGGTGGTGCTATGCGTCCACTCTGGGTGTCCTGGCGCATGGCTGGAACTGCCAACTCGCGCCGTGCATGGGGCAGTTCTCAGTTTGGATAGCGGCACGGGACAGGTCACACGCCGGCACTCGCGGCTTTAGGACACTCCAGAAGGCCACTTCCGAAGCAAGGTCTGTCGTCGTCTTCTGACCATCCGGCCATTGCCCCAGCTGAATGGGGCTGAGTTTGTCTGTTTTCTCTCCCTGAGAGTAAGTTAGGGAGACCCCAGGCCGGGGCTGCCTCCTGCCTACCACGGATTCCAAGGTGGTTCACAGCCCAGATGGCTGACAAGTCCTGCTCCCCGACTCCGGGTGAGACGCCTCTGACCTTCCAAGATGTGGCTGTGTACTTCTCCAGGGCAGAAGGGCAGCAGCTTAGCCCCCAGGAGCGGGCGCTGTACCGGGATGTGATGTTGGAGAACTATGGGAACGTGGCTTCACTGGGATTCCCAGGCCCTAAGCCAGAACTGATCTCCCAGCTGGAGCAAGAGGAGGAGCTGTGGGTCCTGGATCTTCTCGGGGCTGAAGAACCAGAAGTCTTGAGACGTTGCCAGACAGATTCTGAGATCGAGGCCGAGAAGGAGCTATCCATTTTAAACCAGAAATGTTTCGAAGAAGTAAAAACCCCAGAATTCATATCACCGAAATTCCCAAGGGCTTATTCACAGGCGTCTGAGCCCCAGGAAGCTTGTGCCCATGAAGGTCAGGGAGATGGGAGCCACGGAAGCTCTGCTACTCAGGGTTTGAAGAGCATTGCTAAAAAGGACGCTGCGGTCTGTAGGGAGCAGTTTCCCAAGAACGCCCAGACTTCTGTGTTTGACAAACACTTGAATCCCAGCCAAAGTGTTGTTACAGTTCAAAGAAATAAAGCAGGACAGAGAATCTTTAAATGTGACATATGCAATAAAACGTTTAAATATAATTCAGACCTAAGTCGGCACCGCAGGAGTCACACTGGGGAAAAGCCTTATGAATGCGGTCCGTGTGGGCGGGCCTTTACACACAGCTCAAATCTTATTCTTCACCAGCGAATTCACACTGGGAATAAACCATTTAAGTGCGAGGAATGTGGGAAAACTTTTGGGCTCAATTCCTATCTCCGTCTGCATCAGAGAATTCACACCGGAGAAAAGCCGTTTGGGTGTAAGGAGTGTGGGAAGGCCTTCAGTAGAAGTTCAAGTCTCATTCAGCACCGTATAATACACACAGGGGAGAAACCCTACAAGTGTGATGAGTGTGGGAAAGCCTTCAGCCAAAGCCCCCAGTTAACTCAGCACCAGAGGATCCACACCGGGGAGAAGCCCCACGGCTGCACTTGGTGTGGAAAGGCTTTCAGTCGGAACGCGAGCCTTATCCAGCACCAGAGGATCCACACGGGGGAGAAGCCCCACAAGTGCGCTCAGTGCGGGAAAGCCTTCAGCCAGAGCTCCAGCCTGTTCCTTCACCACCGGGTCCACACTGGCGAGAAGCCCTACGTGTGTGGAGAATGTGGCCGGGCCTTTGGCTTCAATTCTCATCTTACCGAGCACGTTAGGATTCACACCGGGGAGAAGCCCTATGTGTGCGGCGAGTGTGGCAAAGCCTTCAGCCGTAGTTCCACGCTGATGCAGCACCGCCGAGTGCACACCGGGGAGAAGCCCTATCAGTGCGCGGAGTGTGGGAAGGCCTTCATCCAGAGCTCCCAGCTCACCCTCCACCAGCGTGTTCACACCGGGGAGAAGCCTTACGAGTGTGGGCTCTGTGGGAAGGCCTTCAGCAGGAGGTCAGCCCTCACTCAGCACCAGCGGGTTCACATGGGCGAGAACCCACAGGAATTTGAGTGTGGCCCCGATTTTGTTTATGACTCCAGCCATCTGTCCGCTGGAGAGAGACACGGCAGGGCCTTCAGCCACAGTGCGAAACTTGTCCTGCAGTGGACGATTCGAAGTGATGAGAAATCCCGGGGTTGTCATGAGTGTGGGAAAACTTACAGCACTTCCTCACAGTCCATGGACTATCAGAAAAGTCAGGCTGGGGAGAAACCCTACAAATGCCAGGAATGTGGTGGTAAAGCTGGTAGTGGTGTGTCGCCCCTTACTCCACACCACGTGACGCGTGTTGGAGAGAAGCCACAATTGAAGGACGGATCTGAAAGATACTTAATTCAAATCAAAAAGATTTTCCAAGAAAGAGATTTTTAATGTGACAAATGTGGAAAAAAGCTTAGAAACGTTGCTTTATATTAGGTAGGATTGCATAATGCAAGTTAAATGAACTGTTACTAACAGACTTGTTCGTTAGGAGGCTGGGGCTGTGTAGCCTATTGTGTCCACCCAACCCACACTCCATGCCCAAGGTCCTGACCCACTCAGAACAGCAGGTCACGTTAGCATCAGGTCAAGTTTAGGTTTTTATCACAGGGTTGAATTCCCACACTAAAGAGCAGTTGATGGGAGGTTTCAAGATGAGGACGTCTCTTCATTGGTGATTTTAAGAGAGAAACCCAGAGAACTGGGGGATTTGGCTTGTCTCAATGATGCTAAGGAAAGATATGGCCATGATTGGCAAGCATGTGGATGGTTTGGGGACCGAGGTGTCTCTGTCAGTGGCTGGTGGGACTAAATGTAGGTTCTCTAGGTGCAAAGGATATGGAAGCTTTGGGGGGAAGCCACTGTGAGGCGAAGGGTGGAGGCTGCAGAGACATCAGCACAAACTGACAGCTGTGGTGACCAGAGCTTAGGGCCAGCAGTGACAAGGACAGCTACAAAGAGTAAAACTGCTGGAGATTGTCCTGGTGCTGTAATTTAAAGATCTTTTGGTCCTCACTGAATTAGTCATGTGCAGTGTACAAATGTTGTAATTTCCATTCTCCTCTGCCAACACAGCATGACTCCCAGGTATCCTCATTACCAGACTTTCCTTTTTAACAAAGTGCTGTCTGTGTTTTGTTTTTTAAAAGTCTTGCCAGGCACTCGAGTGACAGAGACAGGTGGATCTCTCTGAGTTTGAGGTCCCAGATCAGCCAGGGCTACATAGACCCTGTCTCATAAAAAAAAAAAAAAAAAAAAAAAAAAA

>mmZNF7/Zfp7 [RefSeq:NM_145916]
GGCGAGCGGAAGTCGCCCGGGTGGGGCACAGTGGGATAAGCTTGCAAAGCCCTTTCCGGTGGCTCTCTTGTTGGGATCCACCACGCGCCGGGGCAGGTGTCTGACTCAGGACAAGCGGATCCCACCCTCCAGATCTCATGGAGGCGGTGACCTTTGGCGACGTGGCTGTGCACTTCTCTCGAGAGGAGTGGCAGTGTCTGGATTCTGGTCAGAGGGCCCTCTACAAGGAGGTGATGCTGGAGAACCACAGCAGCGTGGCAGGACTAGCAGGATTCCTGGTCTTTAAGCCGGAATTGATCTCCCGGCTGGAGCAAGGACAAGAGCCGTGGGTCCTTGATCTACAGGGAGCAGAGGGGACAGAGGCACCAAGGATTTGCCAGACAGACTCCGCTATTAGAACTGATCGCAAGCAGACCTGTGAGTACACGAGCCTCCTCCAAAGGCAGATTCCTGGCTTTGGAGACAATTTGGATTCTAAGGTCTGGTCAGAGAATTGCCCAAGAAGCCTTGGGCTAAGCGTGAGTGGCTCACTTTTCCAGAAGCACCGTTTGAATAGCGAGGCCGTGATGCCCAAGAACTCCACAAAGGATGCTGTCCAAGAACGTAAGGAGCTGCAGGCCACCGATGTGGGTTATCGGCCTGATGACCAGAGAGACCACCTGAGCTCAAAACTGATTCGGCGTCAAAGCGTTCCTACCGGAGAGAACCGTTACCCATGTGAAGAGTGTGGCAAAGCTTTCAGGTGGCGCTCTCGCCTGAATCAGCACAAACTGAGCCACACTGGAGAGAAATCTTATCAGTGCAATAAGTGTACAAAAGTCTTCGCTTCTAGCTCGCGACTGATTCGCCATCAGAGAGCTCACACCGGAGAAAAGCCGTTTAAATGTGACCAGTGTGGGAAGCGCTTCGTCCTGGCCTCGGTACTCACTCAGCATCAGAGGATCCACACCGGAGAGAGGCCATTTAAATGTGCCGAGTGCGGGAAAGGTTTCCACCTGAGCGCAAAGCTGGTTCAGCATCAAAGAATTCATACTGGAGAGAAGCCTTACCGATGTGAGGAGTGTGGGAAAACCTTTGGTCAGAGCTCAAGCCTCGTCCATCATCAGAGAATCCACACCGGAGAGAGGCCCTTCATTTGTCAAGAGTGCGGGAAAGCCTTCTGCCAGCGCTCACAGCTCAGCAGACACCGGAGGACGCACACTGGAGAGAGGCCCTACTCCTGCCAGGAGTGTGGCAAGGCCTTCTGCCAGAGAGCAACTCTAGCCCAGCATCAGAAGATGATGCATACAGCGGAGAAATCTCAAATGCCGAGAGCCTCAGAGAGCCCAAGCCTTATTGCCTGTCAGGGAAATACCACTGAAGAGAAGCCATTTAAGTGTGAGCAGTGTGGCAAAGCTTTCAGGTGGCTCTCTCGCCTGAATCAGCACCAGGTGGTCCATTCTGGAGAGAAACCTTACCAGTGCAACAAGTGCTCGAAAGCCTTTGGCTGTACCTCACGACTGATTCGCCACCAGAGAACTCACACCGGAGAAAAGCCCTTTAAATGTGACGAATGTGGCAAGCGCTTCGTCCAGAGCTCACACCTTATTCAGCATCAGCGAATCCACACGGGAGAGAAGCCTTACGTGTGTGATGACTGTGGGAGAGCTTTTACCCAGAGCTCCAGCCTCATTTACCACCAGAGGATCCATAAGGGAGAGAAGCCATATAAATGCAGCCAGTGTGGCAAAGCCTTCAGCATGAGCACACAGCTCACCAGCCATCAGAGGACTCACACCGGGGAGAGACCTTATACGTGTAACGAGTGTGGGAAGACCTTCCGTCAAAACTCAACCCTTTTCCAACACCAGATAATTCATGTTAGAGTAAAGCCCTATGAGTGTAATGAGTGTGGAAAGGCCTTCAGCCGGAGCTCATACCTTATTGAACACCAGAGAATACACACTAGAGCCCAGGGGGGCCATGAATTTGGGGACACCCCTGAAAGTCCCACCGTTTTGAACCATAAAAAAGTGCACACTGTAAAAAAGTTGTACCAGTGTGACGACTGCGATAAGGTCTTCAGGTGGCGTTCGTACCTGATTATCCACCAGCGGATTCACACAGGAGAGAGGCCTTATAAATGTAATGCCTGTGGCAAGGCTTTTCATCAGATCGCAAAGCTCACTCAGCACCAAAAACTTCACATGAGATAAATCATTTCCCTAAATGTCTTGGTTACTTCTCTCTTGGCTGTGATGGCACCATGAGTGACCAAGGCAGCTTAGACAAGAGTTCTCGTGGGGCTTACAGTGGAGCGTTAGTTTCAGAGATTGTTCACGGCAGTCTTGGCAGGGAGCAGGGCGGCAGGAAGGCCTGATGTTAGAGCAGTCGCTTAGAGCTCACATCGTGAGACAGCCAGTTACTAGGCAGAGAGATCTAACTGGAAATGGTGAGAGGTTTTAAAAAACCTCAAAGCTGGCCTCCCAGTTACCTCCTCTAAAGAGGGCATACCTCCTAATAATTGCCAAACAGTTCCACCAACTGAGGACCACATGTTGGAACATATGAGCCCCTGGAATTTGTTCTCATCCAAACCACCACACTAAAAGTAATGTAACTATGAATAAGCCAGAAGCCTTATTTAATTTGGGTAATTTATGCTATCAGAGACTTGAGGATGCTAGGAAGATTCAGATTTGTTCTTAAAGAATAGTCTACTTAGGAAATGTTTTATTCGTTTCTGTAATATTTTACCATTTACCCATTAAATAAAAGTGTGTGTCACACTAAAAAAAAAAAAAAAAA


>mmZNF250/Zfp647 [RefSeq:NM_172817]
GAGACGGGGCGGGACGGCCCCTTTGTCTTAGGGAGCCGAAGCTGCCGCCACAGGGCGAGAGGTTGCGGCAGATGCCCCACAGGATCGCCTGATTTCTGTTGTCCTCGCAGTCTTGAGATACCAAGATGGCAGCAGCCGGGCTTCTACCGCTGCCAGCAGCACCACAGGCCAAAGTGACCTTTGAGGATGTGGCTGTTCTGCTGTCTCAGGAGGAGTGGGCCCGCCTGGGCCCTGCTCAGCGGGGCCTCTACCGACACGTCATGATGGAGACCTACGGGAATGTAGTCTCACTGGGACTTCCAGGATCTAAGCCTGTTGTGATCTCCCAGCTGGAGCGAGGAGAAGACCCGTGGGTCCTGGATGGACAGGGAACCGAGCTGAGCCAGAGCCTGGGAAGTGACCACTCAGAATGCAAAGCCAAGGAAGAGAACCAAAACACAGACTTGAATGTGCCACCATTAATCTCAGACGAAGCATCAGCGACGCTGACGGAAACACCGCTGAGGAAGGTTGCTGAAGAACGTTACAAAACAGAACCGAAGGTCTGCCCCAGTCCAAAGCCCATTGGCCCCCAGAATGCCCACGGGTTGAACCCTAGTGTACCCGTTGCCCGACCCCAGACGGCTCCCAGTGTTGCGAGACCCTATATCTGCATTGAGTGTGGAAAGTGCTTTGGTCGGAGCTCCCATCTCCTTCAGCATCAGCGAATACACACTGGCGAGAAGCCCTACGTATGCCATGTATGCGGAAAGGCTTTCAGCCAGAGTTCCGTCCTGAGCAAGCACAGGCGGATCCACACGGGCGAGAAGCCCTACGAGTGTAACGAATGTGGGAAAGCCTTTCGCGTGAGTTCCGACCTTGCCCAGCACCACAAGATCCACACCGGAGAGAAGCCTCACGAGTGTCTGGAATGTGGGAAGGCGTTCACCCAGCTCTCCCACCTCATCCAACATCAGCGGATCCACACGGGGGAGCGGCCCTACGTGTGTCCCTTGTGTGGGAAAGCCTTCAACCACAGCACCGTCCTGCGGAGCCACCAGAGGGTGCACACTGGGGAGAAGCCTCACGGGTGCAGCGAGTGTGGGAAGACCTTCAGCGTGGAGAGGACGCTGCTGCAGCACCAGCGGGTGCACACCGGGGAGAAGCCCTACACGTGCAGCGAGTGCGGGAAGGCCTTCAGCGACCGCTCCGTGCTCATCCAACATCACAACGTACACACTGGGGAGAAGCCGTACGAGTGCAGCGAGTGCGGCAAGACCTTTAGCCACCGCTCCACCCTGATGAATCACGAGAGGATTCACACGCAGGAGAAGCCCTACGCGTGCTACGAGTGCGGGAAGGCCTTTGTCCAGCACTCGCATCTCATCCAGCATCAGAGAGTCCACACGGGAGAGAAACCCTACGTGTGTGGCGAGTGTGGCCATGCTTTCAGCGCACGCCGGTCCCTGATCCAGCATGAGCGAATCCACACAGGCGAGAAACCCTTTCAGTGCACAGAGTGTGGCAAAGCCTTCAGCCTGAAAGCAACTCTGATCGTGCACCTGAGGACCCACACGGGCGAGAAACCCTATGAGTGCAATAGCTGCGGCAAGGCGTTCAGCCAGTACTCGGTGCTCATCCAACACCAGAGGATCCACACCGGAGAGAAACCCTATGAGTGTGGCGAGTGCGGACGGGCCTTCAACCAGCACGGCCACCTGATCCAACATCAGAAAGTACACAAGAAGCTGTGACCCATGGCTGTCAGAAGCCCGCACTTAGAACATGCACAAGACCTAAACAGCCTTCCTCCAGGAGGGAGTTCTGTTCCATGTGCAGAATCGTCGTTCCCTTGGGGACTCTGTTACAGTCCCATGTCCCTCCTGGGGAGAAACATTCAGCATGTGTTAAATTTATTTTCCTCAACATCTTGGAAGTTAAACTGGAGAGTAATCCTACAACTGTAGATACTGGTAAAGACAGCCATAATTCTACCTTTGTCTATTTGACATGAATTAGTTTATTGAAGCAACTGAAAAGCTATAAAGCTTAGGGACTATTCACATTTCAAATGAAGTTCCTTTTACACAGCTTCATGACGTTCCTAAATGTATCTGATCATTCTAATATTGTGCTTAAATTTAGAATGTTGGACCATTTTCGGTATCCTTTTAAAACACTTAAGTGCTATGTTGATAAAGTAATATTAAAAAAAAAAAAAGATCTTGGGCCAGG

>mmZNF16_genomic [GenBank:AC157554]
CCCAAGGAtCACgTTTCTGAAGAgtTGGAATCACAGaCAGAAATACCAACAgtCGTtcGTTGGTGATGTTTCTCCAtCgtCTAAGTTTGGAGGGCTCTcaGATGAACTAccAGAAAgTGACTGgAATGTCTCTGAAGCTGAgAGCCTGCTCCAGCCCCTCTgCcGGTAGGGAaACCTCAagCCAGtGACcATGTTCCTcAgGAGcCCCTTAGGGGAGAAggGTCTGaTTTGtAATGtTTTTGACAaAAaCTTCAGCTTGAGCCCAaGgCCatcAGaGTGTCAGGGAATCTCTaCAGAAGAGAGGCCACGTCCAtgctACACctCAGAGAACTtTCAGcACAATgGaGccCTAACTGGCCAGGAGgGgCTTCCTACAATTGAAAGCCCAtTCATATaTACTGcGTGTGGGAAagcggtcaggggaagCCTTGAcattggTCAGCATCgGgaAGCCAACtCGGGAGAGgTCTTTAATGTGTTCTGAATGTGGAAAGGCCTTTAGCCAAAACTCTGttCTTAAAAaCCACTGgtaggggctggtgagatggctcagcaggtaagagcacccgactgctcttccgaaggtccggagttcaaatcccagcaaccacatggtggctcacaaccatcccttacgagatctgactccctcttctggagtgtctgaagacagctacagtgcacttacatataataaataaataaaaaaaatttaaaaaaaaaaaaaaaccactggcaaccttgcctacctgtgtCAGAcTTTGAcAGGCATCAGGTTCAcCACCACAATGAggAtTATGTGTGTAACaAcTGcGGAAAAGCCTTCAGCCAGAAttcgagccttaaaaaaaaaaaaaacaccaagaggctcacgtgatcgagaagccgtatgaatacagtgaatgtggcaagacttttaggtggaactcaaacctcctccagcatgaaagaatctattctgaagaaaagccatatgcatgcagccagtgtgggaaaagcttcaGGCGGAGCTCGAaCCTCACCAAacaccacagggttcacacaggtgagaagcccttcGAGTGCAGtAgGTGTGGgAAAGCCTTtAGCCAGcGctCACACCTCAGGAAGCATCAGAtgGTTCACACtGGAGAGAAAcCTTAtGcGTGTAAAGAGTGTGGCAAGTCCTTCAGcCGCAtCTCAAACCTCATTAcGCATCACaGGGTTCAcACcGGAGAGAAACCTTAtccgtgccgtgagtgcgGGGAAAGCcTTTAGCCAGAGTTCTAGCCTgacgcagcatcggagatttcacaccggtgagaCCACAGCTCcGCCCTCATCCAaCAgCAGGGTGTaagacacccttgtctccatCACACTGaAGACAAGCCCTAtGcGTGCCAgGAATGTGGGAAGACCTTCAGTCGtAGCTtTAACCTtgTCCTtCACCATCGAGTCCACACtGGGGAAAAACCTTATGAATGTACcGAATGcGGgAggACCTTCAGCAGAGCTCAACCCTCATTCAGCACAGGATgATAACGGgtTGAAACCTCATGAATGTAACCAGTGTGGCAAAaCCTTCAACcGAAGTTCAAATCTCATTCACCACCAGAAAGTgCACACcGGaGAGAGGCCCTACACGTGTGTcGAgTGTGGCAAgGGCTTCAGgCAGAGCTCACActggcGAGAGGCCCTATCAGTGCAGtGAcTGTGaCaGAaCCTTCGGTCAGCGTTCCGTCCTTACCCAGCACCAaAGGAtTCACACTGGAGTGAAGCCCtACGCCTGCgCtGCtTGTGGgAAGgCTTTCAGCtAGCgGTCCAAGTTGGCTAAACACcAGtTGAtTCATAtGGAGtAGCCTGGAGACTGGGAGGaGTGAtgctgagccatgggTCCTCTgGTTCTTCTGgccacccagcgggggaGCCAaTTCACTGATTACTGTGcCCAGCTGcCACAGAACCcaGGGCCTCCtTTGGA


cDNA sequences of rat (Rattus norvegicus) ZNF genes from the chromosomal region syntenic to human 8q24.3:

>rnZNF251 [GenBank:XM_00107545alt]
GTTCCGCCCCtAGtCagCAGTCAgCTTGCCCgcTtaGCGCGGGCCAGGCTGGGTGTAGGTGGaTGCCTGGTCCAGAGcgccctgGCTGAGGtGAGGCTgTCGCTtTGCAGGTGGGCAAGGACAGCCAGCAGaCAGcgtCTCGGGCTGGCTGCCTCtGCTTCTGGCCAAGGGCGCtGATGATGCCGCGACACCTGGGAGGAGGGATAGTGGAGGAGGCAGtGGTCGCAGCCAgAgGGGCCACCCAGCAGCGGGAGAACCCGcGGGAGGGACAAGCgGCcGGCGGCGACGCGGCCTTGGGgGGCATGGATGTGAAGaACGATGgcctttaggTTTTGCCCAGgGCTAaCCCTCGCCGCCTatttcaTGTCCACTCACACCCGCAACCCtGTGAGGCCTaTCGCcGtGAGGCCCGcAGCCTTTTCCAGGCTGGCCCTCTAAgGCTGAGGcGGTGCTATGaGTCCACTCTtGGTGTCCTGGTGCATGGCTGGAACTGCCAACTTGCGCCGTGCTTGGGGCAGTTCTCAGTTTGGATAGCAGCACGGGACAGGTCGGTCACACGCTGGCTCTCGAGGCTTCAGGACACTCCAGAAGGCCACTTCCGAAGCAAGGTCTGTCGTCCTCTTCTGACCATCTGTCCACTGCCCCAGCCGAATGGGGCTGAGTTTGTCTGTTTTCTCTCCCTGAGAGTAAGTTGGAGAGGCCTCAGGCTGGGGCTGCCTCCTGCTTACCACCGATCCCAACCCAGATGGCTGCCAAGTCCTGCTCCCGGACTCCGGGTGAGATGCCTCTGACCTTCCAGGATGTGGCTGTGTACTTCTCCAGGGCGGAAGGGCAGCAGCTTAGCCCCCAGGAGCGGGCGCTGTACCGGGATGTGATGTTGGAGAACTATGGGAACGTGGCCTCACTGGGATTCCCAGGCCCTAAGCCAGAGCTGATCTCCCAGCTGGAGCAAGAGGAGGAGCTGTGGGTCCTGGATCTTCTCGGGGCTGAGGAACCAGAAGTCTTGAGAAGCTGCGGGACAGATTCTGAGATCAAGTCTGAGAAGGAGCGATCCATTTTAAACCAGAAATGTTCCGAAGAAGTAAAAACCCCAGAATTATCACAAAAGTTCCCAAGGGCTAATTCACAGGCATCTGAATCCAAGGAAGCTTGTGCCCATGAAAGTCAGGGAGATGGGAGCCAAGGAAGCTCTGCGACTCAGGGTTTGAAGGGTGTTGCTAAAAAGGACTCCACAGTCTGTAGGGAGCACCTGCCAGAGAACACCCAGGCTTCTTCTGCATTTGACAAACACTTGAATCCCAGCCAAAGTGGTGTTACAATTCAAAGAAATAAAACGGGACAGAGAATCTTTAAATGTGACATATGCAATAAAATGTTTAAATATAACTCAGACCTAAGTCGGCATCGCAGGAGTCACACGGGGGAAAAGCCTTACGAATGCGGTCCGTGTGGGCGGGCTTTTACTCACAGCTCAAATCTTATTCTTCACCAGCGAATTCACACTGGGAATAAACCATTTAAGTGTGATGAATGTGGGAAAACTTTTGGGCTCAATTCCTATCTCCGTCTGCATCAGAGAATTCACACTGGAGAAAAGCCGTTTGGGTGTAACGAGTGTGGGAAGGCCTTCAGTAGAAGTTCAAGTCTCATTCAGCACCGTATAATACACACAGGGGAGAAACCCTACAAGTGCAATGAGTGTGGGAAAGCCTTCAGCCAGAGTCCCCAGCTAACTCAGCACCAGAGGATCCACACCGGGGAGAAGCCCCACGGGTGCAGCTGGTGTGGAAAGGCTTTCAGTCGGAACGCAAGCCTTATTCAGCACCAGAGGATCCACACGGGGGAGAAGCCCCACAAATGCACTCAGTGTGGGAAAGCCTTCAGCCAGAGCTCCAGCCTTTTCCTTCACCACAGGGTTCACACCGGAGAGAAGCCCTATGTGTGTGGAGAATGCGGACGGGCCTTCGGCTTCAATTCTCATCTTACGGAGCACGTTAGGATTCACACTGGGGAGAAGCCCTATGTTTGCGGCGAGTGCGGCAAAGCCTTCAGCCGTAGTTCCACTCTGATGCAGCACCGCAGAGTGCATACCGGGGAGAAGCCCTATCAGTGCGCGGAGTGTGGGAAGGCCTTCATCCAGAGCTCCCAGCTTACCCTCCACCAGCGCGTTCACACTGGAGAGAAGCCTTACGAGTGCGGTCTCTGTGGGAAGGCCTTCAGCAGGAGATCAGCCCTCACTCAGCATCGGCGGGTTCACATGGGCGAGAGCCCACAGGAATTCGAGTGTGGCCCAGATTTCGTCTATGACTCCTCCAGCCACCTGTCCGCTGGAGAGAGACATGGCGGGACCTTCAGCCACAGTGCGAAACTTGTCCTGCAGTGGACGATCCGAAGTGAGGAAAAACCCCGGGGTTGTCGTGAGTGTGGGAAAACTTACAGCTCTTCCTCACAGTCCGTGGACTATCAGAGAATTCAGGCTGGAGAGAAACCCTCTACATGCCAGGAATGTGGTGGGAAAGCTGGGAGCGGGGTGTCGCCCCTTACTCCACACCATGTGACTCGTGTTGGAGAGAAGCCACAATTGAAGGATGGATCGGAAAGATACTTAATTCAAATCAAAAAGATTTTCCAAGAAAGACATTTTTAATGTGACAGATGTGGAAAACGGTTTAGAAACTTCGCTTTATATTAGGTAGGATTCCATAATGAAAGGTAAACTGTTACCAACAGACTGGCGAGTTAGGAGGCTGGGGCTGTGTGGCCTACTGTGTCACAACACAGCAGGCCACCTTAGCATCGGGTCAAGTTTAGGGTTTTATCGCAGGGTTGAATTCCCACAATGAAGAGCAGTTGATGGGAGGCGTCAAGATGATGACGTatCTCTTCATgggcgattgtaattACCCAGAGAcCTGGGGGATTaGGCTTGTCatgtgtgagaatgttgATGATGCTAAGGAcAAGATATGGCCATGgaTGGCAAGCATGTGGATGGTcTGGGaACCGAGGTGgCTCTGcCcGTGGCTGGTGGaACcAAATGTAGGTTCTCTAGGcGCAgAGGAgAcGGAAGCTTTGGGGaGcAGCCACTGTGAGGCcAgGGGcaaAGGCTGCAGAGACATCAGCACAAACcaACAGCTGTGGTGACCAGAGCTgAGGGCCAaCAGTGACAAGGACAGCTACAAAGAGTAAAACTctgGCTGGAGATTGTCCTGcTGgctatATTTtAAGgTCTTTTGGTCCTCACTGAATTAacCATGTGCAGcGaACAAATcTTGTgATTTCCgTTCTCttccgcctacacagcctgactgctgcccgCTCCCAGGTAaCCTCATTACCAGACTTcCCTTTTTcACAAAGTGCTaTCctTGTTTTGTTTTTAAAcGTtTTGCCAGGCACTtGAGTGACAGAGACAGGTGGATggatCTCTgTGAGTTTGAGGTCCCAGAcCAaCCAGGGCTACgTAGACCCTGTCTCcaaAgAAAAAAA


>rnZNF7 [GenBank:XM_235457alt, NM_001142757]
CGGAAGTCGGCCGGGTGGGACACAGAGGGATAGGCGTGCAAAGCCGTTTCCGGTGGCTAGATTGTTGGcgggtccctgacgcgtcccggccgGTGTCTGAgTCAaGACcAGCGGgTCCCACCCTCCccATCTCATGGAGaCGGTGACaTTTGGCGAcGTGGCTGTGCACTTCTCTCGgGAaGAGTGGCAaTGTCTGGATTCcGGTCAGAGGGCCCTCTACAgGGAGGTGATGCTGGAGAACCACAGCAGtGTGGCtgggctagCAGGATTCCTGGTCTTTAAGCCtGAAcTGATCTCCCGGCTGGAGCAAGGACAAGAaCCGTGGGcCCTgGATCTACAGGGAGCAGAgGGGACAGAGGCACCAAGGATTTGCCAGACAGACTCCaaTAcTAGAACTGATtGCAgGCAGACCTGTGAGTACACGAatCTtCTCaAAAGGCAGATTCCTGaCTTTGGAGgCAATTTGGATTCTAAGGTCTGGTCAGAGAATTGCCCAAGAAGCCTTGGGCTAAGaGTGAGTGGCTCcCTTTTCCAGgAGCACCaTTTGAAaAaCGAGaCCaTGATGCCCAAGAACTtCACAAAGGATGCTGTCaAAGAAtGTAAGGAGCTGCAGGCCACtGATtTGGGTTATCaaCCaGATGACCttAGAGACCATGTGAAGgggacatcagaaaactgtgaagtGTGTGGCAgagtcgttaggccagttttgaacctggatccgtatgaaatgaatgggcaggaaagaccatacagatgtcaggagtgccaggaaacatcatctgactgcaaacaggagaaacacacaggtgactgccatgggaagaagccatatgggtgtgaggAGTGTGGGAAagtCTTtaggCTGtgtTCacaACTCAaTCAGCATCAGAGaATCCACACCGGAGAGAaaCCATTTAAATGTGttGAtTGtGGGAAAGGTTTCCgCCTGAGCtCAAAaCTtaTTCAGCATCAgAGAATTCAcACTGGAGAGAAGCCTTACaGATGTGAGGAGTGTGGGAAAACCTTTGGTCAGAGCTCAAGCCTCaTCCATCATCAGAGAATCCACACgGGAGAaAGGCCaTaCAgTTGTCAAGAGTGtGGGAAAtCCTTCaGCCAGCagTCACAGCTggtCAGACAtCaGAGGACtCACACTGGAGAGAGGCCCTACcCCTGCCAGGAGTGTGGCAAGGCtTTCaGCCAaAGctCAACgCTAGCCCAaCATCAGAgGATGCATACtGgGGAGAAATCaCAAATGCCcAGAGCtTCcGgGAGCCCAAGCCTTcTTGCCcGTCAGaGAAAcAaCACTGtAGAGAAGCCATTTAAGTGTGAGgAGTGTGGgAAAGCTTTCAGGTGGaTCTCTCGtCTGAATCAGCACCAGcTGaTCCATaCTGGAGAGAAACCTTAtaAGTGCAACAAGTGCaCaAAAGCCTTTGGtTGTAgCTCtCGACTGATTCGCCAtCAGAGAACTCACACtGGAGAAAAGCCgTTTAAgTGTGAgGAgTGTGGgAAGgGCTTCGTCCAGgGCTCACAtCTTATTCAGCATCAGCGAATCCACACGGGAGAGAAGCCTTAtGTGTGTGATGACTGTGGGAagGCTTTTAgCCAGAGCTCCAGCCTCATTTACCACCAGAGGATCCAcAAGGGAGAGAAGCCATATgAgTGCAtCCAGTGTGGaAAAGCCTTCAGtATGAGCACgCAaCTCACCAGCCATCAGAGGAtTCACACCGGGGAGAGgCCcTATAaGTGTAgCGAGTGTGGaAAGgCCTTCaGTCAAAACTCAACCCTTTTCCAACACCAGATAATcCATGcTgGAGTAAAGCCCTATGAaTGTAgcGAGTGTGGAAAGGCCTTCAGCCGGAGCTCcTACCTTATTGAACACCAGAGAATACAtACTAGAGCCCAGtGGtaCtATGAATTTGGGaACACCaCTGAgAGTtCCACCtTTTTGAACCATAAAAAAGTGaACcCTGTAAAAAAGcTGcAtCAGTGTGAtGACTGtGAcAAGaTaTTCAGGTGGCGcTCacACCTGgTTATtCACCAGaGGATcCACACAGGAGAGAaGCCTTAcAgATGcAATGCCTGTGGCAAaGCTTTTaAcCgGAgCtCgAgGCTCACTCAGCACCAAAAAaTTCACATGAGATAAATCAcTTaCCgAAATGTCTTaGcTACTTCTCTTTcGCTGTGATGGCACCATGACCAAGGCAaCTTAcACAAGcGTTtaCaTaGGGCTTACAGTttctgagaggGTTAGTTTCAGAGAgTGTTCACGGCcaTCTaGGCAGGGAGCgaGGCGGCAagcaggcaggcaggcaggcaggcaggcaggcaggcatTGATGcTAGAGCAGTaGCTTAGAGCTCACATCtTGAGACAGCCAGTTACTAGGCAGAGAGAaCTAACTGGAAATGGcGAGAGtcTTTAAAAgaccccagagctcgcccccagcgacacACCTCCTCcaaggccgTACCTCCgAATAATTaCCgAACAcTTCCACCAACTGAGGACCcggtgttgagacctgagctcgtggaggccaTTCTCATCCAAACCACCACACTAcgtgaatatgaaactgtgaatacacctGAAGCCTTATTTtATTTGGGTAATTTATGCTAaCAGAaACTTGAGGATGCTAGGAAGgATTCAGATTTGTTCTTAAAGAATAGTCTACTTAGGAcATGTTTTGTTTCTGTAATATTTTAgCATTcACCCATTAAATgAAAGTGcGTGTCACACTgAA


>rnZNF250 [GenBank:XM_343279alt]
AGACGGGGCGGGACGGCCCCTTTGTCTTAGGGAGCtGAAGCaGCCGCCACAGGGCGAGAGGTTGCGGCAGATGCCCCACgGGATCtCCTGATTTCTGTTGTCtTCaCAGTCcTaAGAaACCAAGATGGCAGCAGCtggactcctgccactgccagcagcaccccagGCCAAgGTGACCTTTGAGGATGTGGCTGTTCTaCTcTCTCAaGAaGAGTGGGCCCGCCTGGGCCCTGCTCAGCGGGGCCTCTACCGAaACGTCATGATGGAGACCTACGGGAATGTAGTCTCACTGGGACTTCCgGGATCTAAGCCTGTTGTGATaTCCCAGCTGGAGCGAGGAGAAGAtCCGTGGGTCCTGGATGGACAGGaAACtGAGCTGAGCCAGgGCCTaGGAAGTGAtCACTCAGAATGCAAAGCCAAGGAAGAGAACCAAAACACAGACTcGAATGcGCaACCcTTAATCTCAGACGAAGCgTCAGCcAtGtTGgCGGAAACACCGtTGAGGAAaGTTGaTGAACaTTACAAAACAGAACCGAActTCTGtCCgAGTCCAAAGtCtgTTGGCCCCCAGAATGCCCACGtGTTGAACCCcAGTGTACCaGTTGCtCGgCCCCAGAtGGCTCCCAGTGgaGaGAGACCCTATATaTGCATTGAGTGTGGAAAGTGCTTTGGTCGGAGCTCtCAcCTCCTTCAGCATCAGCGAATACACACTGGCGAGAAGCCCTAtGTgTGCCAcGTATGCGGcAAGGCcTTCAGCCAGAGTTCtGTCCTtAGtAAGCACAGGCGGATCCACACGGGCGAaAAGCCCTAtGAGTGTAAtGAATGTGGGAAAGCtTTTCGaGTGAGcTCtGACCTTGCCCAGCACCACAAGATaCACACgGGAGAaAAGCCcCACGAGTGTCTGGAATGTGGGAAGGCGTTCACCCAGCTCTCCCACCTCATCCAgCAcCAGCGGATCCACACcGGGGAGaGGCCCTAtGTGTGTCCCTTGTGTGGGAAAGCCTTCAACCAtAGCACCGTCCTGCGGAGCCACCAGcGGGTGCACACTGGGGAGAAGCCcCAtGGGTGCAGCGAGTGcGGGAAGACgTTCAGtGTGaAGAGGACGCTGCTGCAGCACCAGCGGGTcCACACtGGGGAGAAGCCCTACACGTGCAGCGAGTGCGGGAAGGCCTTCAGCGACCGCTCCGTGCTCATCCAgCATCACAAtGTACACACcGGGGAGAAGCCGTACGAGTGCAGCGAGTGCGGCAAGACCTTTAGCCACCGCTCCACCCTGATGAAcCACGAGcGGATTCACACcCAGGAGAAGCCCTACGCGTGCTACGAGTGCGGGAAGGCCTTTGTCCAGCACTCGCAcCTCATCCAGCAcCAGAGAGTCCACACtGGAGAGAAACCCTACGTGTGTGGCGAaTGcGGgCAcGCTTTCAGtGCACGCaGGTCCCTGATCCAGCATGAGaGAATCCACACAGGtGAGAAACCCTTTCAGTGCACAGAGTGTGGCAAAGCtTTCAGCCTGAAAGCAACTCTcATCGTGCACCTGAGGACCCACACcGGCGAGAAACCCTATGAGTGCAATAGCTGtGGCAAaGCcTTCAGCCAGTACTCGGTGCTCATCCAgCACCAGAGGATCCACACgGGAGAGAAACCCTAcGAGTGTGGtGAGTGCGGtCGtGCCTTCAAtCAaCACGGgCACCTGATCCAgCATCAGAAAGTgCACAAGAAGCTGTGAtCCATGGCTGTCAGAAGCCCaCACTTAGggggAcCATGCACAAGACCTAcACAaCCTTCCTCCAGGgaGGAaTTCTGTTacatccagagacccTTCCCTTGGtGACTgTGTTACAGTCtCATGTCCCTCCTGGGGATTATTTTCCTCAACATCTTGAtGTTAAAtcaGAGAGTAATCCTACAAtTGTAGATACTGcTgAAGACgGCCATAATTCTACCTTTGTCTGACATGAATTAGTTTATTGAAGCAACTGAAAAGCTATAtatacggtgTAGGtACTAcTCACATTTCAAgTGAAGTTCCTTTTcttACAtAGCTTCATGACGTTCTAAgTGTATCTGATCATTCTAATATTGTGCTTAAATTTAGAAcGTTGGACCATTTTtGGTATCCTTTTAAAACACTTAAGTGCTATGTTGATAAAGTgATATcAAAAAA

>rnZNF16_genomic [GenBank:NC_005106]
cacaaccgaggaggagccgttctaccccaaggagcacatttcTGAAGATatcTGGAATCACAGGCAGAAATAccaacacccgtctgtTGGTGATGTTTCtCCAGcccctaagtttggagggctctgtgatgaactattagaaactgactgcaatgtctctgaagctgacagcctgctccagcccctctactggtagggacacctcacaccagggactatgttcctgaagagaCCCTTAGGGGAGAAAcgTCTGcttTGcAATGGTTTTGACAcaaGCTTCAGctTGAGCCCAtgtCcGcTaGtgTGTCAGGgAATCtCTtCAGAAGAGAGGCCACgTCCAcATGACActgcagagaactctcagtacaatagggggctaactggccaggagagccttcctacaatTGAAAGcCCACTCATATGTAcTGAGTGTGGGAAggcattcagaggaacccttgaccttgatcagcatcagagagccaacccgggagagccatctttaatgtgttctgaatgtggaaaggcctttagccaaaactctgaccttaaaagccactgtcaaccttgcctccctgggaaagcctaccagtgtggcaagtgtacagaagtctttgtggggcacacagattttgataggcatcaggttcagcaccacaatgaataatatgtgtgtaacGAATGTGGAAAAGCCTTCAGCCAGAACTtaAGCCTTcAAAAcCACCAAAAGTCTCAtgTGAtgGAGAAaCCtTATGAgTGCAATGAgTGTGacAAGGCTTTTgGGCGGAGCTCgAgCCTCATCCAACATCgAAGAATCCATTCTGaaGAaAAACCaTAcGTaTGCAGctgGTGTGGGAgaGCCTTCAGaCGgAGCTCAAACCTCcTCAAACACCACAGGAtTCACACAagtgaatCCTTTTGAGTGcaGCaAGTGTGGtAAAGCCTTCAGCCAGAGTGCACACCTcAGGAAGCAtCAGAGaGTtCACACcGGAGAGAAatCTTAcGAGTGTAAaGAgTGTGGCAAGtCCTTCAGaCGcacCTCaAACCTCATTAAGCAtCACcGGGTTCAtACaGGAGAGAAaCCtTActcGTGCAGTGACTGTGGGAAAGCATTTAGcCAGAGtTCtAGCCTTATTCAGCAcCGGAGAATTCACACTGGtGAgAAGCCTCACGTGTGcAATGTgTGTGGgAAAGCCTTcAGTTAcAGtTCtGTGCTCaGAAAGCACCAaATaATtCACACGGGAGAGAAGCCGTACgGgTGtgGTGTCTGTGGGAAaGCCTTCAGCCACAGCTCAGCCCTCATcCAGCAtCAGGGtGTGCACACtGGaGACAAGCCCTACGagTGCCACGAaTGTGGGAAGACCTTcaGTCGaAGCTCtAACCTCATCCTgCACCAtCGAGTCCACACcGGgGAaAAaCCtTATGAATGTACgGAATGTGGAAAAACCTTCAGCCAGAGCTCAACCCTCATTCAGCAcCAGAGGATTCATAAcGGaCTGAAaCCtCATGAATGTAACCAGTGTGGcAAAGCCTTCAACaGAAGtTCAAATCTCATTCACCACCAGAAAGTaCAcACTGGGGAgAggCCCTACACgTGTGTTGAATGTGGcAAaGGCTTCAGCCAGAGCTCACAtCTCATTCAaCAcCAGATAATCCACACtagagagaggccctatcagtgcagcgagtgtggccGAGCCTTCGGTCAGCGTTCCGTCCTTACCCAGCACCAGAGGACTCACACTGGAGTGAAGCCCCACGCCTGCTCGGCGTGTGGAAAGACTTTCAGCCAGCCGTCCAAGTTGGCTAAACACGAGCTGACTCATAAGGAGCAGCCTGGAGACTGGGAGGGGTGAAgctccattcctctgttcttctgaccctggtttcagccgccccactcccagctgtgtggccagttcactgattactgtgtccagctgtcacagaacctggggcctcccttggatcacccttcagggctccc

>"rnZNF252_krab_B" (genomic rn4 chr7(+):115003928-115004038)
agccaaggttcgaaacccagtatcagttccaggctggagcagggatcagagccttgggccccacacttgtcaagagtctctgaggagtcaaagctggtgacgttgtttccc


cDNA sequences of opossum (Monodelphis domestica) ZNF genes orthologous to human 8q24.3 ZNF genes:

>mondomZNF252 [ENSEMBL:ENSMODT00000005415]
tgaagctacaataggactctattgtttcagacatctCTGATGTTCAAGGATGTGGCTGTGTACCTTACTCGGGAGGAGTGGGACTGTCTTGGCCCTGCCCAGAGGAGCCTCTATAGGGATGTGATGCTGGAGAATTACAGGAACCTGGTCTCATTGGGATTCTCAGTATCCAAACCTGATGTGATTTCCCATTTGGAAAAAGGGGAAGAGCCTTGGGTCTTGGACAAACAGGGAGCTAGAGAGAGAGAGATCCTAAAAAGGGAGATAGCAGATATAATGAGTTGGCTCAGAGAGAGGAGAGGGGATTTGAAGATTTCCCCTCTTCTGCCTTCCCTCCTTAGCCAAGTTGCACTCCCCAATTTGCTATTGTCCCTCTTGTCTCCCCTCCACTACTCGAGACCAAAGGGAAGATTCAAGGGACCTGAGTTTGGAGAACCCTGTAAAATTGAGAATCAAACAGAAAAACACTGGGAAAAACCTGTGGTGGGAGTCCTGAGGAAATCCCTTTCCTTGGAAAGAAATTATAAGCCAGTGACAGTGATCTCTATGAAAACCTCTACAAGGGTGAAAGGTCAGGAATGTAATGAATTTGGAAGGCGCTTAAATCTGACTTCAAAATGTGTTAAACATCAGCGGGTTCCAAGAAGTGAAAAGATCCATCCATGTGCAACTTGTGGCCAAACTTTCAAAAAGAATTCAGACCTTATTAATCATCAGAGTATCCATACAAGAGAGAAAGCTCATAAACTTAATATATATGGGGAAATGCCTAGACAGAGTTCAATTAGTATTGAGCATCAGCGACTTCACAACACAGAGAAACTCTATGAGTGTAATGAATGTGGAAAAGCTTTTAGCCAGAACAGAACACTTATCCAGCATGAGAGAATTCATACTAGAGAGAAACCCTATGAGTGTGGTGAATGTGGAAAAACTTTTAACCGCAGTTCTATCCTAACTAAACATCAGAGGATTCATACTGGTGAGAGACCCTACAAATGTAATGAATGTGGAAAAGCCTTCAGTGCTCGTTCATACTTTTTTCAACATCGTAAAATTCATACTGGAGAGAAACCTTATGAGTGTAATGATTGTGGGAAGTCCTTTAGTACTCGATCATCATTTACTCAGCATGGTAAAATTCATACTGGAGAGAAACCTCATGAATGCCATCAGTGTGGGAAAGCCTTTAGTCATAGTTCTAACCTTATTCATCATCAGAGAATTCATACTGGAGAAAAACCCTATAAGTGTAAAGAATGTCAGAAAGCCTTCAGTAGACACTCACATCTTATTCAACATCAGAGAATTCACACTGGAGAAAAACCATATGAGTGTAATGACTGTGGAAAAGCCTTCAGTGCACGTTTATCCCTTATTCAGCATCAGAGAATTCATACTGGAGAGAAACCATATGAGTGTAATGAATGTGGCAAAACATTCAGCCTAAATCGAACCCTTATTGTACACCAAAGGATTCACACTGGGGAGAAACCCTATGAGTGTAATGAATGTGGGAAATCCTTCAGTCAACGCTCACAAGTTATTCAACACAAGAGAATTCATACTGGAGAGAAACCATACATATGTAATGAATGTGGAAAATCCTTTAGTGCACGCCTATCTCTTATTCAACATCAAAGAATTCATACTGGAGAGAAACCCTATGAGTGCAATGAATGTGGCAAAACCTTTAGCCAGAAAGGTCACCTTATTCAGCATCAGCGAATTCACACAGGAGAGAAACCCTATGAGTGTAATGAATGTGGGAAAGCCTTTAGTCAGAGTTTTAATCTTATTCACCATCAGAGAACACATAATGGAGAGAAACCCTATGAATGTAATGAGTGTGATAAAGCATTCAGTGTGCTTTCTTCTCTTGTTCAACATCAGAGAGTGCATAATGGAGAGAAACCCTATGAATGTAATAAATGTGGGAAAGCCTTCAGTCAGGGTTCACACCTTATTCAACATCAGAGAAGTCACACTGGGGAGAAACCTTATGAGTGTAATGAGTGTGGGAAAACCTTTGGCCAAATTTCAACTCTTATTAAACATGAAAGAACACATAATGGTGAGAAACCATATGAGTGTAATGAATGTGGAAAAGCTTTTAGTCAGAGTGCGCACCTTATTCGACATAGGAGAATTCACACTGGAGAAAATCCCTATGAGTGCAATGTCTGCGGGAAAGCCTTCAATGTACGTTCATCACTTATTCAGCACCAGAGAATACACACTGGAGAGAAACCTTATGAGTGTAATGAATGTGGAAAAGCCTTCAGTCAGCACTCACAGTTTATTCAGCACCAGAGAATTCACACTGGGGAGAAACCCTATGTTTGTGATGAATGTGATAAATCCTTCAGTGCACGCTTATCACTTATCCAACATAAGAGAATTCACACTGGAGAAAAACCCTATGAATGCAGTGAATGTGGAAAATCATTTCGCCAAAGTTCTCATCTTATTCGACATCAGAGGATACACAGTGGAGAGCGACCTTTTATATGTGATGAATGTGGAAAAACCTTCAGCCAGAGAATAACTCTTATTAGTCATGAGAAGATTCACACCAGAGAATAAtcttgtgaatgtaatgcacacgaggaagcatttagatatcctcatctttaattcagcatcacaaaattcactgtagggagagttacattgtgaatgtaatttgaaaaagttctctttgcactcacacaaaattttgcattatagagtttataatggagagaaatactgtaagaaaacttgcttcataaatttttacaattttagaa


Other ZNF cDNAs used as outliers and outgroups in alignments:

>ZNF10 [RefSeq:NM_015394]
GACTCACCTCTGACGCCGCTCTTCGCGCTCCGCTGGTGAATGGAGTCGCGTTCTCTGTTTTGCTGTTGCTGCTGCCTTTGTGACGGGATCGCTTTCTCCCATCGAACCTTCTAGTTGCTTATTGCAGCTTTGTCTCCTCAGCACTCTGCTGTCACTCAAGGAAGTATCATCAAGAACAAGGAGGGCATGGATGCTAAGTCACTAACTGCCTGGTCCCGGACACTGGTGACCTTCAAGGATGTATTTGTGGACTTCACCAGGGAGGAGTGGAAGCTGCTGGACACTGCTCAGCAGATCGTGTACAGAAATGTGATGCTGGAGAACTATAAGAACCTGGTTTCCTTGGGTTATCAGCTTACTAAGCCAGATGTGATCCTCCGGTTGGAGAAGGGAGAAGAGCCCTGGCTGGTGGAGAGAGAAATTCACCAAGAGACCCATCCTGATTCAGAGACTGCATTTGAAATCAAATCATCAGTTTCCAGCAGGAGCATTTTTAAAGATAAGCAATCCTGTGACATTAAAATGGAAGGAATGGCAAGGAATGATCTCTGGTATTTGTCATTAGAAGAAGTCTGGAAATGTAGAGACCAGTTAGACAAGTATCAGGAAAACCCAGAGAGACATTTGAGGCAAGTGGCATTCACCCAAAAGAAAGTACTTACTCAGGAGAGAGTCTCTGAAAGTGGTAAATATGGGGGAAACTGTCTTCTTCCTGCTCAGCTAGTACTGAGAGAGTATTTCCATAAACGTGACTCACATACTAAAAGTTTAAAACATGATTTAGTTCTTAATGGTCATCAGGACAGTTGTGCAAGTAACAGTAATGAATGTGGTCAAACTTTCTGTCAAAACATTCACCTTATTCAGTTTGCAAGAACTCACACAGGTGATAAATCCTACAAATGCCCTGATAATGACAACTCTCTTACTCATGGTTCATCTCTTGGTATATCAAAGGGCATACATAGAGAGAAACCCTATGAATGTAAGGAATGTGGAAAATTCTTCAGCTGGCGCTCTAATCTTACTAGGCATCAGCTTATTCATACTGGAGAAAAACCCTATGAGTGTAAAGAATGTGGAAAGTCTTTCAGCCGGAGTTCTCACCTCATTGGACATCAAAAGACCCATACTGGTGAGGAACCCTATGAATGTAAAGAATGTGGAAAATCCTTCAGCTGGTTCTCTCACCTTGTTACTCATCAGAGAACTCATACAGGAGACAAACTGTACACATGTAATCAGTGTGGGAAATCTTTTGTTCATAGCTCTAGGCTTATTAGACACCAGAGGACACATACTGGAGAGAAACCCTATGAATGTCCTGAATGTGGGAAATCTTTCAGACAGAGCACACATCTCATTCTGCATCAGAGAACCCATGTGAGAGTGAGGCCCTATGAATGCAATGAATGTGGAAAGTCTTACAGCCAGAGATCTCACCTTGTTGTGCATCATAGAATTCACACTGGACTAAAACCTTTTGAGTGTAAGGATTGTGGAAAATGTTTTAGTCGAAGCTCTCACCTTTATTCACATCAAAGAACCCACACTGGAGAGAAACCATATGAGTGTCATGATTGTGGAAAATCTTTCAGCCAGAGTTCTGCCCTTATTGTGCATCAGAGGATACACACTGGAGAGAAACCATATGAATGCTGTCAGTGTGGGAAAGCCTTCATCCGGAAGAATGACCTCATTAAGCACCAGAGAATTCATGTTGGAGAAGAGACCTATAAATGTAATCAATGTGGCATTATCTTCAGCCAGAACTCTCCATTTATAGTTCATCAAATAGCTCACACTGGAGAGCAGTTCTTAACATGCAATCAATGTGGGACAGCGCTTGTTAATACCTCTAACCTTATTGGATACCAGACAAATCATATTAGAGAAAATGCTTACTAATAAATATGGGAATTTTTCACAAAGAGCAATGACTTTATTTTGCATTGGAGAACTCCTGGAGATAAGCTGTACAAATTGAATCTATGTGGAAATGCTTTCAGTCTTGTTACTATCCTATTGCACATTAGAGAATTGGTCCTGGAAGGGAAAGAAACCACAGATTTTATTTCAGTACACAAATCCATCAGATTTTCTTCTTTTCATGAATTCCTACAGAAGTAATTGGCCTGAGAGCATTCTTGACCAAGTCTTAAATGCTAGAATCTGAGAAGGAATTATTAAATAGGTGAGTTGTTGAGCGAGAACCCCTTCATTTGAAAAGAAATGAGTATGCTACTATAGGGAGAGTTGTTGCTGAGAATTAAGAAATGATACAGTTAATGCAACAAAAGATGGAAAATAATATTTCAGTCAATATGTCATTGTTTTCTTGACTATGTCTCTCTTCTGGGACATTTAGTAGTGTTTGGTATGTTTTATGTGTCTGGTAGAAACCATATTTTGGTTAACAGCAAGAAAAATGCTTATAATGTAGTACAATTAAAAACAACACATCTCCACTACCAGTGCTAACCCATTTTTAAGTACATTTGCATGTGGGCAAGAATTGAAAGTATACAGATAATTGAACAGAATTGATTTGTTAGATAAGGAGATTTTGACTGAGTTTTATAGTCTGTTTAATGTTGCTGTAATAATTATTTTAAGAAACTTTTAAATATTGTAAGAGGATATCTAGTTTCTCTATTCTACCATCAAAGAAGCTTTTGAGTACCACCTGTTAATGAGCTTTCCTATTCTAAATTGTTTTGGGTCACAGAGTTCCACTTTTTCCACTCTTATTAGCACTGCAAAAGCTCCTGAGAATTTAAAAACACAGTAATTCTCTGGATGTTAGGACCTAGGGGAACATTGGGCATTTGAACATATCAGGGAGGGTCCCCATTTTAGTGGGAACAAGTATTTAAACAATATTTAGAGCAAGTGTCCTCATGTGATAAACAGAGCACAGTCCAAAGATACCCTCTTTCTCAAGGTAGTCTTTTATCTTTATAAAGAAAGATTAGTGTTTAAGAGCGTAGACTTGAGCTAGACTACCCAGGTTGAAACCCCACTAGCTGGGTGATCTTGAACATGCTGCTTAGCTTCTCTGTGCCAAACTTACTGATATCCTCATCTGTAAATTAGCGATAATAATAGTACTTACCTGGTAGGATTATGGTGAGTATTAAATGAGTAAATGGAAACAGCTTAGAATAGTGCCTGACATATATTATGTTCTCTGTAGTTACTGGCTGTGATTATTAATAATATTGCCGCACCCTAATATCTGTTATTTAATTGAAATCTGCTTGTGTGCTTATAAGAATTTACTGAGTTCTCACTTCCCTTACAGTCATACATGGTTTTTCCCTTTTGCCTAAATCAGAACTCAACAGCCTGGGAAAATCACTGACAAATAGTGGATATAGGTTTCATTTCTGAGGATCAATAAACAGCTTGTAATGTTGTATACACATTTATTCTGTTTGTACAAAGGTTTTCCATTCTATGGAGAGGATCCCAGTCTTCAGATTCTCTGGTAAGTTAATAACCCACAAAAGATTCAGGAAGAGGATCTATTTTAATTTTCTCTTTCATTCTATAAATCAGTCTGTTGTATATAGGGACTGACCATGATTCTCCATTTTCTGGCATAAATATTAGCTGCTCAATGATTGATTGTTGAATGATAGATGAAGTATTGCTGAAGCCAACCAGAGATCTTGATCTCTCTCTGAGGAAAAAACCTAGAAATGATGGTTAGAATCAGGAGACTTGAGAACTACGAGGAACATGGCAGCCTCTAGCTCATACTTGTCGTTGTACAATTGAGATCAAGTGAATTGGCCCCGGTCTCACATTTTCAGTGAAGTCAGACTCAGAACTAGGTCCTGGGTTTCATGTTTCCTGCTGCTCTTCTCACTGTGTACACACCATGCCCACATACAACATACCTATCAGAAATGGTTTTCATTAAGGGAGTAGAATAGTCACTTAACTGGGGCTCTTAACATTGCTAATAACCTGTGGCTCAATTTCCTCAACTGTATAATGAGGTTACTACTAGTATCTACCTCAAAGCGTTGTCATGTGGATTGAGATGATATGTATGAATCACATAAAAGAGTGCCTGGCACATAGTAGGTGCCATTAATAATATTACTATTGTTAACACCTTAAGGGTCTAACTTGTAATGAAGACAGAAATATGTATGAGGATGATCACTGAAATATTTATGACAGTGCATAATTTGCAAAATAAAAAATGTACACTAGCATAATATTTGTGGCATATCCGTGTAATGGAAGATTATGCAGGCATTAAAATATGTACACAAAAAGTTTGGTAAAGGAAAAATGCTGTCATGTGTTACAAAAGCATGATAAATAATTGTTAATATGGAATAAGCTCAAATATGTCAAAATAAATGTAAAAAATGAGTGTTGCCCAAAAAAAAAAAAAAAA


>ZNF136 [RefSeq: NM_003437]
CAGAGGCCCAGAGTGGCTCGCCTGGAGTCTCTGTGGCGCGGTTTCCTGTACCTGCCTTGGGATCCGGAGGGAGGAAGCTGGGACACCCGGGAGTCAGGAAATGGACTCGGTGGCTTTTGAGGATGTAGATGTGAACTTCACCCAGGAGGAGTGGGCTTTGCTAGATCCTTCCCAGAAGAATCTCTACAGAGATGTGATGTGGGAAACCATGAGGAATCTGGCCTCTATAGGGAAAAAATGGAAGGACCAGAACATTAAAGATCACTACAAACACCGAGGGAGAAATCTAAGAAGTCATATGTTAGAAAGACTCTATCAAACTAAGGATGGTAGTCAGCGTGGAGGAATTTTTAGCCAGTTTGCAAATCAGAATCTGAGCAAGAAAATCCCTGGAGTGAAACTCTGTGAAAGCATTGTATATGGAGAAGTCAGCATGGGTCAGTCATCCCTTAATAGACACATCAAAGATCACAGTGGACATGAACCAAAGGAATATCAGGAATATGGAGAGAAGCCAGATACACGTAACCAGTGTTGGAAACCCTTCAGTTCTCACCACTCCTTTCGAACACATGAGATAATTCACACTGGAGAGAAACTCTATGATTGTAAGGAATGTGGAAAAACCTTCTTTTCTCTCAAAAGAATTAGAAGACACATCATCACACACAGTGGATATACACCATATAAATGTAAGGTGTGTGGGAAAGCTTTTGATTATCCCAGTAGATTTCGAACACATGAAAGAAGTCACACTGGAGAGAAACCCTATGAATGTCAGGAATGTGGAAAAGCCTTCACTTGTATCACAAGTGTTCGAAGACACATGATAAAGCACACTGGAGATGGACCTTATAAATGTAAGGTATGTGGGAAACCCTTTCATTCTCTGAGTTCATTTCAAGTGCATGAAAGAATTCACACTGGAGAAAAACCCTTTAAATGTAAGCAATGTGGTAAAGCCTTCAGTTGTTCCCCAACCTTACGAATACATGAAAGAACCCATACTGGAGAGAAACCTTATGAATGCAAGCAGTGTGGGAAGGCCTTCAGTTATCTCCCCTCCCTTCGACTACATGAAAGAATTCACACTGGTGAGAAACCCTTCGTATGTAAACAATGTGGTAAAGCCTTTAGATCTGCCAGTACCTTTCAAATACATGAAAGGACTCACACTGGAGAAAAACCTTATGAATGTAAGGAATGTGGGGAAGCATTCAGTTGTATCCCAAGTATGCGAAGACACATGATAAAACATACTGGAGAAGGACCTTATAAATGTAAGGTATGTGGGAAACCCTTTCATTCTCTGAGTCCATTTCGAATACATGAAAGAACTCACACTGGAGAGAAACCTTATGTATGTAAACATTGTGGTAAAGCTTTCGTTTCTTCAACATCAATTCGAATACATGAAAGAACTCATACTGGAGAGAAACCCTATGAGTGTAAGCAATGTGGGAAAGCCTTCAGTTATCTCAACTCCTTTCGAACACATGAAATGATTCACACTGGTGAGAAACCCTTTGAATGTAAGCGATGTGGTAAAGCCTTTAGATCTTCTAGTTCCTTTCGACTACATGAAAGGACTCACACTGGACAGAAACCCTATCATTGCAAGGAATGTGGGAAAGCCTATTCTTGCCGTGCCAGCTTTCAGAGACACATGTTAACACATGCTGAAGATGGACCACCTTATAAATGCATGTGGGAAAGCCTTTAATGCTCTGGGTTCATGTCAGATACATTAAAATACTCACTGAAGAGAAGCCCTATGAATGTAAGTAACGTGGGAAAGCATGAAATTCTGTCAGTGCCTTTTTTAATACATGAAAGAATTCTAGAGAGAAGCCATATACATGTAAGTAACATGGGAAAGCTTTCAATCATTTTAGTTCCTTTCAAATACATGAAAGAACTCATCATGGAGAAAACCAAACAAATGCTTTTTGGAAAGGAACCCATATTTGAGAGAAACCCTGGGTAAAGGGTGTAGGAAAGGTTTTGTCATCACACAACCCTGTCACACATGAATGTGTAGTGGAGAGAACCTTGGTAAATGTAAAGAGTGTGGGAGAGCCTTTAGTCATTTTAGTTCCACTTGAAGCCATGAAAGAACACACAAAAGAGAAAGCTCTTGAATATAAGCAATGTCCACACGTTTTTAGCCAGCCCACATGCTTTCAACAACATGTAAGAATGTACCCTGGATCAAAACACTATAAATGTGAAAACTACAAGGAAGGTTTTCCATTATACTTTTAAAGTCATTTGAGGGCTCTGGCTCTGTGGCTTTGTGGGTTAAAGTACTTGTCTGGCAAACAGGAGATGCTGGATTTCAATCCCAGCAGGGCCTCACTAGTTGAGGTATTGAATTTGAACAGATGTTTTGGCTCTGGCTGGGTGTGGTGGCTCACACCTGTAATCCCAGCACTTTGGGAGGCTGAGGCAGGAGGATCACTTGAGGTCAGGAGTTTGAGACCAGTGTGCAAAACATAGCAAGACCTCATCTCAGAAAAAAAAAAAAAGTCATTTGAAAACTTCTAATACAGCAAAATCCTATAAATGTAAGTACTTCAAAAAGCCTCATGCATAGCGGGTCATGTAGTCTCTAGAAAATACATAACCTGATGGAAATGTAGAAGCTATATGTATATTTTGTTAATTAGTGGCTCATATTTAAAATAGTTCTCTGACTATGGATTTCAAATGCTTAATCTCACAAAGAAATGTTAAATTGATATAATCCTGTAAGTAATCTGAAAGCAATAGTGCATGAATTTCATAGGTAGTGTTGTTTTAGGTCAGTAAATAAAATATTTGTCTTTCCATTTTGAAATGTGTTTAATCCAACAATGAATTTAAATGTGTTTCTCATATTCAGGTATGTTAAATTGTATATGGTTGCTTAATTGTTCTGTGATTGAGGACCACTGAAAAATAAGTCTTAATAAATGTTGGTATGAACCTAAAAAAAAAAAAAAAAAA

>ZNF248 [RefSeq: NM_021045]
CAATTATCCACGGGAGACTCGGTTTTCCTCTCTGTAAATTACGGATATTAGGAATACTTGCCGCCTGGGGTTGTTTTGAGCACTTTCATTGTTTAGGTGCTGTATTTATTGGGTGTGCGTTCCCTGGGAGAGCCCGAGGGAGACGGCTCCTGCACCGGCCCCAAGGGCCTCTCTGCCCGTGGAGTGCAGGAGCGAAGGGGCTGGCCTAGCTGACCACCAGGGCCCCTGCTGGCTCTGACCGCCCTCCTATCCCAAGTAATTATTTACACGTTTCGCGTTCGCTTATGTATTATGTGTAATCATGGCACAGTATCCTGAAGCCGTCGGAGTCTGTGTGCTGGTGCAAGGTCTGGGACAAATTTAAGAAGAGAGAGAACCTAAATTGGTAATAAATCAATAAGAAATATTTACATTCACTCAGTGAAAAGTGTCATGTCTGAGCTCAGCATATATCGGAGCCACACATGGACACCTCTCCTTCCTCCACTAAGAGCGGAAAATGAACAAATCCCAGGAACAAGTGTCATTCAAGGATGTATGTGTGGACTTCACTCAGGAAGAGTGGTATCTGCTGGACCCTGCTCAGAAGATTCTATACAGAGATGTGATCCTGGAAAATTATAGCAATCTTGTCTCAGTAGGGTATTGCATTACTAAACCAGAAGTGATCTTTAAGATCGAGCAAGGAGAAGAGCCCTGGATATTAGAAAAAGGATTCCCAAGCCAGTGCCACCCAGAAAGGAAATGGAAAGTTGATGACGTGTTAGAGAGCAGCCAGGAAAATGAAGATGACCATTTTTGGGAGCTTCTATTCCACAACAACAAAACAGTAAGTGTAGAAAATGGAGATAGAGGAAGCAAAACTTTCAATTTGGGCACAGACCCTGTTTCTTTAAGAAATTATCCCTATAAAATATGTGACTCATGTGAAATGAATTTGAAAAATATTTCGGGCTTAATTATTAGTAAAAAGAACTGTTCCAGAAAGAAGCCTGATGAGTTTAATGTATGTGAGAAATTGCTCCTTGATATTAGGCATGAGAAAATCCCTATTGGAGAGAAGTCTTATAAATATGATCAAAAAAGGAATGCCATTAATTATCACCAGGATCTCAGTCAGCCAAGTTTTGGCCAATCTTTTGAGTATAGTAAAAATGGACAAGGCTTCCATGATGAGGCAGCATTTTTTACAAATAAGAGATCTCAGATAGGAGAGACAGTCTGTAAATATAACGAATGTGGAAGAACCTTCATTGAAAGTTTAAAGCTGAATATATCTCAAAGACCTCATTTGGAAATGGAGCCGTATGGATGCAGTATTTGCGGGAAGTCCTTCTGCATGAATTTAAGGTTTGGACATCAGAGAGCTCTTACAAAGGACAATCCTTATGAATATAATGAATATGGGGAAATCTTCTGTGACAATTCAGCTTTCATTATCCATCAGGGAGCTTACACAAGAAAGATTCTCCGTGAATATAAAGTGAGTGACAAAACCTGGGAAAAGTCAGCTCTCTTAAAACATCAAATAGTACACATGGGGGGAAAGTCTTATGATTACAATGAAAATGGGAGTAATTTCAGCAAGAAGTCACATCTTACCCAGCTTCGGAGAGCTCACACAGGAGAAAAAACCTTTGAATGTGGTGAATGTGGGAAAACCTTCTGGGAGAAGTCAAACCTCACTCAACATCAGAGAACACACACAGGAGAGAAGCCCTATGAATGTACTGAATGTGGGAAAGCCTTTTGCCAGAAACCACACCTGACCAACCATCAGCGAACACATACAGGAGAAAAACCCTATGAATGTAAGCAATGTGGAAAAACATTCTGTGTGAAGTCAAACCTCACTGAACATCAGAGAACACACACAGGGGAGAAGCCCTATGAATGTAATGCATGTGGGAAATCCTTCTGCCACAGATCAGCCCTCACTGTGCATCAGAGAACACACACAGGGGAGAAACCGTTTATATGTAATGAATGTGGAAAATCCTTCTGTGTGAAGTCAAACCTCATTGTACATCAAAGAACTCACACTGGGGAGAAACCATATAAGTGTAATGAATGTGGGAAAACCTTCTGTGAAAAATCAGCTCTCACTAAACATCAGAGGACTCACACAGGGGAGAAGCCGTATGAGTGTAATGCATGTGGGAAGACCTTTAGTCAGAGGTCAGTGCTCACCAAACATCAGAGAATTCACACAAGGGTGAAAGCTCTTTCAACATCCTGAATGTTAGAAGCCTTCATACACTTGTGAAATTGGTTATACAGTTTCAAAAAAGGAGATCAGAGAAAGCCAAAGAATGTCAGAAATTTGTAGAAAATGACTTCTTGTTTGAATATGTAAAAGCTTTCAAGAAAAATTAAAACTTTTCATTAGAAAATTTGTACTGAGGGGAATTCTATTCATCTAAGTAATATGGTGAAAATATTTATCTGGAATTTATGTTGTTTAGTGTTATATTCCAGACGTGATACCAAAATTTTGTTGCAAATATAATGGACAATATTTATTTATACCCATATTCACAGTGGAATCTGAAGCTTATAAAAGTTGAATGACAGCAGCATTAAACATATATGTGAAGAGTCCCCGATGTTTGTAGACCTTATGTGAGTATGCAAATATATAAATTTGAGTATGCTTGATTTGTATATTGGAACTCAACATGATCATAAGGAGAAGATACGTACCCTTAATTGAGTAACTACTATGGTATTTGTTAATATTTTCTACACTAAATACCATTGGTGTCTTTATAGGTTGACATAATTATATATGTGTATGTACATATGTTTGTGTGTATATGTAAATATATTTCTACACACATACTTAAATATAGTGATGTGCTAGTATAACCTCATACTGACTTAAAAGTTCTGATTGTTAAATTTTAAGGAATTTTGTGAGCCAGTTATTAAAAGCAGTCATTATTTAAAATATGTAAACTTACAGTTAAAAACAAAGGTAATAAATACTCAGCACTCATCACTTCCTAATTATTTTGCTACATTTCACTATTACCTTTGCTGTTTTACTTATTTAATCTGTATGATGAAAATACTGTATAATAGTGTGCACTGCACATCTGTCTCTTCCCAGCTCCACATTCAGTGCTGTCTTGGTAGCTTGGACTTAGTGGGAGTGTTTACACCATGGAAATTGACAAACTATAAATCAGGGTTTTAATTTTCTCAGAGAACCTGCTGTCAAATATTTACAGCACATCACTGTGTATATATGAAAACGTATTTGGCAATACAAACCACATACCCCTTCTATTTCCTGACATAAATAAATGGCTATGGCCATTTACAGCTGAACCACGTCTTCAAGAAAGAAGCCAAAAATATTTCCGTGAGGTTTTTAACTACCTCTGAATCTGTCCTACTCTAAATACTACCGGAGTCTCTTTGTAGGTTGGCCAGTATATGTTTTTAGTGAAATATTATTTCACAAAGAACTATATCACGTACCTTTCCTCTGACTGTTTCCTGGCATATATGCATGAATATGGCCATTATTGAACTATCACTTCAGTAAAGAAGTTAAACAGTACTTTTCTGAGGTTTTTCAGCTACCTCTGGGTCATTCTGTAATGTAAATGTTGTTAATAAGAATGGTTTTTACATAAATTATGCAAAGGTTAACAAGCAGTAACACTGCACTCCTCAAAAAGTGGCGGTATGTAATGAAAGGCCCTTTTGATATCCTTGATTTTTCATTGTGTATCTGTTTGGGCACGGTCTATGTAACACTAGTTCTGCGTATTAGTATTTTAGAGTATCTCTGCCTCCCTTGTCCTGTTGTTTCTTTTGCCCCCTTGGAACACATTGGTCAGCAGTTCTAAGAGACACTGCCCACATGATGGCCATTCCCTACTTCATCCTTGCTGAGCTAAATTTTATATTTTTGTGCATCCTTCTCCCAGATGACTTAGGTGGTAAGTCCAGATTAGTCAAAGCTAATCATGGAAGTTCCATTTTAATGATTCTGTTGGGGTGAACTTGGGAGCAATGAGATGTTTGGGAAGTATTGTGTAGTACTTCTGGGAAAGATCTCCTTGATACAACATTGTCATGACATGAGAAGAGACTCTGCTGGGCTTTTTCATGTCTGTAACATGGTATTGGCTTATCGTTTTTATCTCTGAAGGGCAGTAGCCTGAAGATAACAGTGCACAAGGTGGGAAAAGCCAGCTCAGAGGTGACGTTGCCGAGCTACTCTGCTCTCTATACCTGTTCTCTACTGGGACTTTTTATAACCCTCAATAACTGTTTTTTATTTGGTCTTAGGGCTGTCTGATACTTAGAGCTGAAGGCATTCCAGCTGACACAGAGGAATATTTTTCTAAGTGTTAATGTTCTATATGGTAATTAGGGGGAAGAATTATTTCTTTTCACAAGTTAATATAGGGATGGCTGTTTGTATCAGCCATGGTTCTTTCTGGTGGAAAACAGAATTCTCCAACTAAAAATATTTTAATGGCAGACTGATTACAGTGGTGTGGGCCAGAAACAAGGGACAGTGAAACACCCAGAGACTTGTATCAGCAGGAAGCCATTGCCATTCTGAGCCTTGAAGGGCAAGGAGGGAAACAGTGTTACCAGAGCCCAGTAAGAACTGCTGTCATGAAGGAGGGGCCACCTTGTAAGAGACATCATTACTACCAGAACTGTGGTGCCAAATTGCTGGTGTCTCTCTTTGGAGAAACCAACCAGATACATCTGCTGGAGAGCCCAGGTGGGCACAGAGAAGGGTGGAGAGAGAATCTGGGAAGAGAAATGGAGAATAAGCAGCACAGTGTTATTCATTTCTGTAAATTCCTATGTAGAAGGCTCAGTGTTAGAAATAAAGTTATTCTACTAGTTGC

>ZNF25 [RefSeq: NM_145011]
CAGAGGCCGAGACCCTGCAGGTGATTCCTGGGGCTGCCAGCTAAGGTCCCTTATGATTTCTGCTCTGGCTTTGCAGTTTTCAGCCTTTCCCAAGAGCAGCAGAAAATGAACAAGTTCCAGGGACCCGTGACATTAAAGGATGTTATTGTGGAATTCACCAAGGAAGAATGGAAGTTACTGACCCCTGCTCAGAGGACTCTGTATAAGGATGTGATGCTGGAAAACTATAGTCACCTTGTCTCAGTGGGTTACCATGTGAATAAGCCAAATGCAGTCTTCAAGTTGAAGCAAGGAAAAGAGCCATGGATATTAGAAGTAGAATTTCCACATCGGGGCTTCCCTGAAGACCTATGGAGCATTCATGATCTAGAAGCAAGATACCAGGAAAGCCAAGCTGGAAATTCAAGGAATGGAGAACTCACAAAACATCAGAAAACTCATACCACAGAGAAAGCCTGTGAATGTAAGGAATGTGGGAAGTTCTTCTGCCAGAAGTCTGCCCTCATAGTACATCAGCATACTCACTCAAAGGGCAAATCCTATGACTGTGATAAATGTGGGAAATCTTTCTCTAAAAATGAAGACCTCATAAGACATCAGAAAATTCACACGAGAGATAAAACCTATGAGTGTAAAGAATGTAAGAAAATATTTTACCACCTATCATCTCTCAGTAGACATCTGAGAACCCATGCAGGAGAGAAACCCTATGAATGTAATCAGTGTGAAAAATCCTTCTACCAGAAACCACATCTCACAGAACATCAGAAAACACACACAGGGGAGAAACCTTTTGAATGTACTGAATGTGGGAAGTTCTTCTATGTGAAGGCATACCTCATGGTACATCAGAAAACACACACAGGGGAGAAACCCTATGAGTGTAAGGAGTGTGGGAAAGCCTTTTCCCAGAAGTCACACCTCACAGTACATCAGAGAATGCACACAGGGGAGAAACCCTATAAATGTAAGGAATGTGGGAAATTCTTCTCTAGGAATTCACACCTCAAAACTCATCAGAGAAGTCACACAGGAGAGAAACCCTATGAATGTAAGGAATGTAGGAAATGCTTCTACCAGAAGTCAGCCCTCACAGTACATCAGCGAACTCACACAGGGGAGAAACCCTTTGAATGTAATAAATGTGGGAAAACATTTTACTATAAATCAGACCTCACTAAACATCAGAGAAAACACACAGGGGAGAAGCCCTATGAATGCACAGAATGTGGCAAATCTTTTGCTGTGAATTCAGTCCTCAGATTACATCAAAGGACTCACACAGGAGAGAAGCCCTATGCATGCAAGGAATGTGGGAAGTCCTTTTCTCAGAAGTCACATTTTATTATACATCAGAGAAAACACACAGGGGAGAAGCCCTATGAGTGTCAGGAGTGTGGGGAAACCTTTATCCAGAAGTCACAACTCACTGCACATCAGAAGACACACACAAAGAAGAGGAATGCTGAGAAGTAAGATGAGTTGGGAAATTCTCTTGACTGAATTAATCCTTCAAAACAATCAGATAATTCACACAAGAGGTACACCTTATGAATCGCATCAATAGAGGGAAATTTTCAGCTACAATCTTTGCTCTCTCTGTGTATCAGCAAATACATAGAGAGATTCTGTACATTTATTTGGGAGAAAATTTTATCATATTTTAGGCTTTTCTAAATATTAGATAATATAGATGGCAGAAGTCATGCAAATTGTAAAACAGGAAGGAAACCTTTCAGAAGTCATACTTTTGTTTTGCAACATAGAAGTCACACAGGAGAAAAATCCTGTAAGTAAAACGAATGTCAAGACATTTTCTGTCAGGGCAGCCAGCTGTAGACATCCAAAAGCTCACAAATGAGAAGCTCCTTGAGTATCTAGAAATCTCTTTACACAATTGGAGCTCATGTACTAGAGAATTCAAAGGGATAACTGCAAAGACACTGGTAGATATAGAAGCCTTTATTAAGAATTGATGTTTCATTCAATTTCAGATCACTGGTACTTGGATAGATATTTTAAATATTTGGAAGGTGTTCAATAAAAATTCAAATAATTTGTACTGAGGGAAACAGTTATACTACAAATCATGTTGCAGATCTGATGCCGAGATTGCTCTATTTTAAAAGGAAACCTGCAAATGTCTACATATATGAAGCTTTTAAAAAGCACAAGTTATCAGGCAACATCATTAAACATACATTAAATGTCTGTTAGCATATAGGATTTTGTGCGTATGTGAGTGTGCTATAACATGTAACTTGCGGATGTTCAGCGTGCATGTGGAATCCATCCATAATGGTACATAGCGAAATAATATAATCTTAGTTCAGTAACTATTATGTGTATGAAGATGTTGCACATTAAGTCTCAGTAGTGACTCTGGTTGTTAATATTTGTATTGTAAGTGTAATATTCCTTTTAAATTATAAGGCATCTTTTTATCCTTTTTTGTGTTTGTAAATGGATATAGACAGTGGTAATAACTAATCCTTCAAAAATAAGTTAAAATGTTTTTGTAAAATTTTCAAATGTCAGTCAAGTTTTGCATTAGGGACACATTTATATATAGCAAAAGCCTGAGCTGTTTTTAAACAGCTAAAAACAGTAGTATTTATTACAATCTCCACAATGAAAACAAATAGTGCAATGTAAGAAATATTATGTATCTTACTGTTGATGTGTGAGCAGGGAGCAGAGCTATCTGCATTTGTATTTAGTGTGAGAGTCCTAAATGTTCTTCACCAACTTTTTCTTCCTCATAGAAGCAACATGGCATAGTTAAGGGTGTGGACTCTGGGCTCCTGCTGCCTGGGCCCCGATTCCAGCATGGGGCATGCTACTTAATATCTCTGTGCCTCAGTTCCCATCCCATAAAAGAGTAGTGATAGTGACAACATCTTAGGATTGCTTTGAGGTCTAAATGAGTTAACATTTTCAAAGCACTTAGAATAGTGTCTGAGACACAAGAGCTATATGTTAGAGGTTATCATCATTATTTGTATTTTGTGAATTACGCTGCAAAAAAAAAAAAAATGTTGGCTACCCAACATTCATTCCCCACCTTCTTGCTGCCAGTGCCTTGATTTGGTTGTTTGTTGTTTGTTTGTTTTCCTACAAGTGATTTAGGGGCAGATCCTGACTACACTAAGTCAGGGTTTGACAAATGACAGTCCATCAGACAGATCTGGCCCACTACCCATTTTTGTATCCTGCAAGCTAAGAATGGTTTTATATTTCTAAATGGTTGAAAGAATCAAAGAATATTGTGACACATGAAAATTATATGATATTCAAATTTCAGTGTCCATAAATAAAGTTTTGAAAACATGAATACAAACAAAAAACACCTATTTATGTTTGACCTGTGGCTGTCTTTGCACTACCCCAGCAGAGTTGAGTAGTTGTAGCAGACACCACATGCCCTTGAGCCTAAGATCTAGCACTTTACAAAGTAAGTTTTTCTGTTACTTAGAGCTGAAAATAAAAGCAATGTAGAGTTACGCTTTTATAAGTATTTCATGTTTCAGAAATGATGCATACAGCAATTTGTAGGTGGGTGGTTTTTTGCAGTATATTTATTTTGTGCATTATATTTATATGTTAAGAGCCTGAGCTTAGAAGTATTTTTTTCAAAGAAATGTGTTTTTTACTTGCTAAATTGTCATAACATCAAAACAAGAAATTTCTTGATGGAATGTAGCAGTAAACATATGAAATAAACATTGTCTAAATT


>ZNF439 [RefSeq: NM_152262]
GAAAAAATTGCTTTATGGAAGAAAGTAAGTATAGACAGAGAGAAAGGGATCTGATGACCAAAGCAGGGAATAAATGTTTGGAGTCCACGGCATCCTGAGAACTTCTTGGGAATAGAGTCTAGGCCCCCAATGCTGTCACTCTCACCCATCCTCCTCTACACATGTGAGATGTTTCAGGACCCAGTGGCTTTTAAGGATGTGGCTGTGAACTTCACCCAGGAGGAGTGGGCTTTGCTGGATATTTCCCAGAAGAATCTCTACAGGGAAGTGATGCTGGAAACTTTCTGGAACCTGACCTCTATAGGAAAAAAGTGGAAAGACCAGAACATTGAATATGAGTACCAAAACCCCAGGAGAAACTTCAGGAGTGTCACAGAAGAGAAAGTCAATGAAATTAAAGAAGACAGTCATTGTGGAGAAACTTTTACCCCAGTTCCAGATGACAGGCTGAACTTCCAGAAGAAGAAAGCTTCTCCTGAAGTAAAATCATGTGACAGCTTTGTGTGTGAAGTTGGCCTAGGTAACTCATCTTCTAATATGAACATCAGAGGTGACACTGGACACAAGGCATGTGAATGTCAGGAATATGGACCAAAGCCATGGAAGAGTCAACAACCTAAAAAAGCCTTCAGATATCACCCCTCCTTGAGAACACAAGAAAGGGATCACACTGGAAAGAAACCCTATGCTTGTAAAGAATGTGGAAAAAACATTATTTACCATTCAAGCATTCAAAGACACATGGTAGTGCACAGTGGGGATGGACCTTATAAATGTAAGTTTTGTGGGAAAGCATTCCATTGTCTCAGTTTATATCTTATCCATGAAAGAACTCACACTGGAGAGAAACCGTATGAATGTAAACAATGTGGTAAATCTTTTAGTTATTCTGCTACCCATCGAATACATGAAAGAACTCACATTGGAGAAAAGCCTTATGAATGTCAGGAATGTGGGAAAGCATTCCATAGTCCCAGATCCTGTCACAGACATGAAAGGAGTCACATGGGAGAGAAGGCTTATCAATGTAAGGAATGTGGAAAAGCATTCATGTGTCCCCGTTATGTTCGTAGACATGAAAGGACCCACTCTAGGAAAAAACTTTATGAATGTAAGCAGTGTGGGAAAGCATTATCCTCTCTTACAAGTTTTCAAACACACATAAGAATGCACTCTGGAGAAAGACCTTATGAATGTAAGACATGTGGGAAAGGCTTTTATTCTGCCAAGTCATTTCAAAGACATGAAAAAACTCACAGTGGAGAGAAACCGTATAAATGCAAGCAATGTGGTAAAGCCTTCACTCGTTCCGGTTCCTTTCGATATCATGAAAGGACTCACACTGGAGAGAAACCCTATGAGTGTAAGCAATGTGGGAAAGCCTTCAGATCTGCCCCAAATCTTCAATTGCATGGTAGGACTCACACTGGAGAGAAACCGTATCAATGTAAGGAATGTGGGAAAGCTTTCAGATCTGCCTCACAACTTCGAATCCATCGTAGGATTCACACTGGAGAGAAACCCTATGAATGTAAGAAATGTGGGAAAGCCTTCAGATATGTCCAGAACTTTCGATTTCATGAAAGGACACAAACACATAAGAATGCACTCTGGAGAAAGACCTTATAAATATAAGATATATGGGAAACACTTTTATTCTGCCAAGTTATTTCAAACACATGAAAAAATTCACACTGGAGAGAAACCCTATAAATGCAAGCAATGTGGTAAAGCCTTAATTGTTCCAGTTCCTTTCGATATCTAAAAGGACTCACAGTGGAGAAAAACTCTATGAGTGTAAGCAATGTGGGAAAGTCTTCAGATCTGTCAAGAACCTTTCAATTTATGAAAGGACACACACTGGAGAGAAACCCTATGAATGTAAGAAATGTGGAAAAGCGTTCCATAATTTCTCTTCTTTTCAAATACATGAAAGTTGCACAGAGGAGAGGCGCCCTAAGAATGTAAGCATTGTGGGAAAGCATTCATATCTGCCAAGATCGTTTGAATACATGCAAAACACACACTGGAGAGAAACCTATGAATGTAAGGAATGCAAACAAGCATTCAATTATTTTTCTTCCTTGCATATACATGAAAGGACTCATACGAGAGAGAATCCGTATGAATGTAAGGATTGTGGGAAAGCATTCAGCTTGCTTAATTGCTTTCATAGACATGTAAAGACACACCAGAAGGAAACCCTATGAATGTAAGCAATGTGGCAAAAGCTTTCACTTCTTCCAGTTCTTTTCAATATCATGAAAGGACTCACACTGGGGAGAAACCGTATCAATGTAAGCAATGTGGGAAAGCCGTCAGATCAGCCTCAAGACTTCAAATGCATGGAAGCACTCACACTTGGCAGAAACTCTATGAATGTAAGCAGTATGGGAAAGCCTTCAGATCGGCTAGGATTCTTTGAATACAAATAATGAATGTAAACAATTAACTGTTTATAATAACTGTATACTAACAAATGTTATTCTTTTTAAATAATTAAGAAGCTATAATAAAATATCCATTGGTGTCATGTATTAGATCAAGCTTAAAAAAAAAAAAAAAAA


>ZNF101 [RefSeq: NM_033204]
TTAGTTCCTCGGGGAGCCCCTGGTGCCCCGGATACGGCTGATTTTGTCGTGTGGGACCTGTTCTGGCTGCTCCAGCCCCAGGAAGGACCCAGGACACCCGGAAGCCGGAAATGGACTCAGTGGCCTTTGAGGATGTGGCTGTGAACTTCACCCAGGAGGAGTGGGCTTTGCTGAGTCCTTCCCAGAAGAATCTCTACAGAGATGTGACGCTGGAAACCTTCAGGAACCTGGCCTCGGTCGGAATCCAATGGAAAGACCAGGACATTGAGAATCTGTACCAAAACCTGGGGATTAAGCTAAGAAGTCTGGTGGAGAGACTCTGTGGACGTAAAGAAGGGAATGAACACAGAGAAACTTTCAGCCAGATTCCTGATTGTCACCTGAACAAGAAAAGTCAAACTGGAGTGAAACCATGCAAATGCAGCGTGTGTGGGAAAGTCTTCCTCCGTCATTCATTCCTGGACAGGCACATGAGAGCTCATGCTGGACACAAACGATCTGAGTGTGGTGGGGAATGGAGAGAGACGCCCCGTAAACAGAAACAACATGGGAAAGCCTCCATTTCCCCCAGTAGTGGTGCACGGCGCACAGTAACACCAACTCGAAAGAGACCTTATGAATGCAAGGTGTGCGGGAAAGCCTTTAATTCTCCCAATTTATTTCAAATCCATCAAAGAACTCACACTGGAAAGAGGTCCTATAAATGTAGGGAAATAGTGAGAGCCTTCACAGTTTCCAGTTTCTTTCGAAAACATGGAAAAATGCATACTGGAGAAAAACGCTATGAATGTAAATACTGTGGAAAACCTATCGATTATCCCAGTTTATTTCAAATTCATGTTAGAACTCACACTGGAGAAAAACCTTACAAATGTAAACAATGTGGTAAAGCCTTCATTTCCGCAGGTTACCTTCGGACACATGAAATCAGATCTCACGCGCTGGAGAAATCCCACCAATGTCAGGAATGTGGGAAAAAACTCAGTTGTTCCAGTTCCCTTCACAGACATGAAAGAACTCATAGTGGAGGAAAACTCTACGAATGTCAAAAATGTGCCAAAGTCTTTAGATGTCCCACGTCCCTTCAAGCACATGAAAGAGCTCACACTGGAGAAAGACCTTATGAATGTAATAAATGTGGTAAAACCTTCAATTATCCCAGTTGTTTTCGAAGACATAAAAAAACTCATAGTGGAGAAAAGCCATATGAATGTACAAGGTGTGGTAAAGCCTTTGGGTGGTGCAGTTCCCTCCGAAGACATGAAATGACTCACACTGGAGAAAAACCCTTTGATTGTAAACAGTGTGGTAAAGTCTTTACTTTTTCAAATTACCTTAGACTTCATGAAAGAACTCATTTGGCCGGGCGTAGCCAGTGCTTTGGCAGGAGGCAGGGGGATCACCTGAGCCCAGGAGTTTGAGACCAGCCTGGGCAACATAAGAAGGCCCCCGGAATTC

>ZNF20 [RefSeq: NM_021143]
CTACTCCGAGAGGCCCCGGGTCCCTCTGCCACAACTTCTGTCGCTCTGCCGCCTGCACCGTGACCCGCACTATTCACGGGAGCCCTAGAGAGGACACCGGGACACCCAGAAGCCGGGGAATGATGTTTCAGGATTCAGTGGCCTTTGAGGATGTGGCTGTCAGCTTCACCCAGGAGGAGTGGGCTTTGCTGGATCCTTCCCAGAAGAATCTCTACAGGGATGTGATGCAGGAAACCTTCAAGAACCTGACCTCTGTAGGAAAAACATGGAAAGTTCAGAACATTGAAGATGAGTACAAAAATCCCAGGAGAAATCTAAGTCTTATGAGAGAGAAACTCTGTGAAAGTAAAGAAAGTCATCACTGTGGAGAAAGCTTCAACCAGATTGCAGATGACATGCTGAACAGGAAAACTCTTCCTGGAATAACACCATGTGAAAGCAGTGTGTGTGGAGAAGTTGGCACGGGTCATTCATCTCTTAATACGCATATCAGAGCTGACACTGGACACAAGTCATCTGAGTATCAGGAATATGGAGAGAATCCATATAGAAATAAGGAATGTAAGAAAGCCTTCAGTTATCTTGACTCCTTTCAATCACATGATAAAGCTTGCACTAAAGAGAAACTCTATGATGGTAAAGAATGTACAGAAACCTTCATTTCCCATTCATGCATTCAAAGACACAGGGTAATGCACAGTGGAGATGGACCTTATAAATGTAAGTTTTGTGGGAAAGCCTTCTATTTTCTCAATTTATGTCTTATCCATGAACGAATTCACACTGGTGTGAAACCATATAAGTGTAAACAATGTGGTAAGGCCTTTACTCGTTCCACTACCCTTCCAGTACATGAAAGAACTCACACAGGAGTGAATGCCGATGAATGTAAAGAATGTGGGAATGCATTCAGTTTTCCTAGTGAAATCCGTAGACATAAAAGGTCTCACACTGGAGAAAAACCCTATGAGTGTAAGCAATGTGGGAAAGTCTTCATTTCTTTCAGTTCCATTCAGTATCATAAGATGACTCACACTGGAGAGAAACCCTATGAATGTAAGCAGTGTGGGAAAGCCTTTAGATGTGGCTCACACCTTCAAAAGCATGGAAGGACTCACACTGGAGAGAAACCCTATGAATGTAGGCAATGTGGTAAAGCCTTCAGATGTACCTCGGACCTTCAAAGGCATGAAAAGACACACACTGAGGATAAACCCTATGGATGTAAGCAGTGTGGGAAAGGCTTTAGATGTGCTTCACAACTTCAAATTCATGAAAGGACGCACAGTGGAGAGAAACCCCATGAATGTAAGGAATGTGGAAAAGTATTCAAGTATTTTTCTTCCTTGCGTATACATGAAAGGACGCACACTGGAGAGAAGCCCCGTGAATGTAAGCAATGTGGAAAAGCATTCAGGTATTTCTCTTCCTTGCATATACATGAAAGGACACACACTGGAGATAAGCCATATGAGTGTAAGGTATGTGGCAAAGCCTTCACTTGTTCCAGTTCCATTCGATATCATGAAAGGACTCACACTGGAGAGAAACCCTATGAATGTAAGCACTGTGGTAAGGCCTTTATTTCCAATTACATTCGATATCATGAAAGGACTCACACTGGAGAGAAACCCTATCAATGCAAGCAATGTGGCAAAGCCTTTATTCTGCCAGTTCATGTCGAGAACATGAAAGAACTCATACCATTAATAGATGAGAAATCCTTTTAGTGGAAGCAATGGGAGAAGCTTTCTGTTGTCCCACTTCCTTTGAAACACAAGAAAAGAGAGTGGTGAAAGAACCTCTGGATAACTGCTTTTTGAATTGAGAAGAGAGACTTGCGATAGGACAATAAAATCTAGAAGAACTTGGATGGTTTCGTAATACAATTCACCTATAGCCAATCTTGCATGAGATTTCAAAGGCACAGAAAAGGAAGGCATCAGTCACATATTAGAGGCCCCACACAGGGAGAGAGGTGTGTGGCTACCCGCTTCCAGCCCATTTTCCTGATTCTTTGGCTTGTTAATCGAGAATATCCCCCAAATCACTTGTCTCTGTTCCTCAGAGTTGTAGGTCTCCAGATATGTCCCCAGTGCTCCACATTAAAGATACATGCCCACTTTGCTGTTTCTTCAGAAATGTTAAAATGTTTACCAGAGAACTAATAAATGTTGTTATGGCTGGAAATACTCACTGATGCCCAACTCTTCTATAGATTGAGTTGGTTTAAGAGGTCTACTTTGTATTGCAAGCATTGTTGTTTCTACGGATTAGCGAGCCTGTTCTTGATTAAACCAACTAGATGGGAGCTTG


>ZNF23 [RefSeq: NM_145911]
CGGAGGTGAGTGCAGGGCTCCGGGCGCTGTGTGCCGTGCCCTGTCCCCCAGGCTCTGGGTCCGGTTGAGACGCACTCCCACCCTGGGTGCTGCAGGGTCTCCCGTGGCCTACGCTGCCAGGAGGCCCAGGCTGGGAGAGGGAGCAGGGCGGCGGCCCGGGGTGCTCTTCCGCGCTCCTCGACTCCAGCTTTGGTCTTTGTGGCTCAATAGCTTGCGCCCATTGGAAGGGGAAACGCACGTTGCTGTCCCAGCCACGAGGCCCTGAGCATTTAGCACTTGGTCGACGGGGGAAGATGTGTCGGTGCAGCTAAAAGATGGAATATTTGCGGGCGTGAAGCGGCCCGAGGTGATACCACAAGTCTTTTGACATGCTGAGGTCTGGCAGGAACATTAACCTATGTGCATCCCGAGTATGGACCGTGTGCTCCCAGGCTCCTGACATAGGGTCATGAATTAGGGCCGAGTGGGAGCGCAGAGCCCCTCCCAGTCACCCGGCAGCAGAAGCAGCCCGGCTTTTGGAGGACATTGTCTCCTGGAGCAGTGTCAGTCCCAAAAGCTCCAGCCCTTCTCTGAGACGGGGACCAGGGGATGGCAGCCATGCACCTGACAGCCTGGCCCCAGGGACTTCACGGATCCCCTGAAGCTCATCCATGGACTTCCATGGACTACAGGGCCTGAGCTATGGAATTGGCCCTAGGGCAGCAGGAACCTATTGTTTCAGAAGTCGGTGACCTTTGAGGACGTGGCTGTGTACTTCACCCAGGCGGAATGGGATGGCCTGTCCCCTGCACAGAGGACCCTGTACAGGGATGTGATGCTGGAGAATTATGGGAATGTGGCCTCCCTGGGATTTCCACTTCTCAAACCTGCTGTGATCTCACAACTGGAGGGAGGAAGTGAGCTGGGGGGCTCATCTCCACTGGCTGCAGGAACAGGCCTCCAGGGCCTCCAGACTGATATTCAGACTGACAATGATTTGACAAAGGAAATGTATGAAGGAAAAGAGAATGTATCATTTGAACTTCAAAGAGACTTTTCCCAGGAAACAGACTTTTCAGAAGCCTCTCTTCTAGAGAAACAACAGGAAGTCCACTCAGCAGGAAATATAAAGAAGGAGAAGAGCAACACCATTGATGGAACAGTGAAAGATGAGACAAGCCCCGTGGAGGAGTGTTTTTTTAGTCAAAGTTCAAACTCATATCAGTGTCATACCATCACTGGAGAGCAGCCCTCTGGGTGTACAGGATTGGGGAAATCCATCAGCTTTGATACAAAACTCGTGAAGCATGAAATAATTAATTCTGAGGAAAGACCTTTCAAATGTGAAGAATTAGTAGAGCCCTTTAGGTGTGACTCTCAACTTATTCAACATCAAGAGAACAACACTGAGGAAAAGCCTTATCAGTGTTCGGAGTGTGGCAAAGCTTTCAGCATTAATGAGAAATTAATTTGGCATCAGAGACTTCACAGTGGGGAGAAACCCTTCAAATGTGTGGAGTGTGGGAAAAGCTTCAGCTACAGTTCCCATTATATCACACATCAGACAATCCACAGTGGGGAGAAGCCCTATCAGTGTAAGATGTGTGGGAAGGCCTTCAGTGTTAATGGAAGCCTAAGTAGGCATCAGAGAATCCATACGGGAGAGAAGCCCTATCAGTGCAAGGAATGTGGAAATGGCTTCAGCTGTAGTTCTGCATATATTACACATCAGAGAGTCCACACTGGAGAGAAACCTTACGAGTGTAATGACTGTGGGAAAGCGTTCAATGTTAATGCAAAATTAATTCAACATCAGAGAATCCATACTGGAGAGAAACCTTATGAATGTAATGAATGTGGAAAAGGCTTCAGGTGCAGCTCCCAGCTTAGGCAGCATCAGAGCATCCACACAGGAGAAAAGCCCTATCAGTGTAAAGAGTGTGGAAAAGGCTTCAATAATAATACAAAACTCATTCAGCATCAGAGAATCCACACAGGTGAGAAACCCTATGAATGCACTGAATGTGGAAAAGCCTTCAGTGTCAAAGGGAAGTTAATCCAACACCAGAGAATTCACACAGGCGAGAAACCCTATGAGTGTAATGAATGCGGGAAAGCCTTCAGATGTAACTCCCAATTTCGGCAGCATCTGAGAATTCACACTGGGGAGAAGCCCTATGAGTGTAATGAGTGTGGAAAGGCCTTCAGCGTTAATGGGAAACTAATGCGGCATCAGAGAATTCACACTGGGGAGAAACCTTTTGAATGTAATGAGTGTGGGAGATGCTTTACTTCTAAAAGAAACCTACTTGATCATCACCGAATCCATACTGGAGAAAAGCCCTATCAATGTAAGGAATGTGGGAAAGCCTTCAGTATCAATGCCAAACTAACTAGGCATCAGAGGATACATACTGGGGAGAAACCTTTCAAATGTATGGAATGTGAGAAAGCATTCAGCTGTAGTTCTAACTATATTGTGCACCAGAGAATCCATACAGGAGAGAAACCCTTTCAGTGTAAGGAGTGTGGAAAAGCCTTCCATGTTAATGCCCATTTAATTCGGCATCAGAGAAGCCACACTGGGGAGAAACCCTTCAGATGTGTGGAATGTGGCAAAGGCTTCAGCTTTAGTTCTGACTACATTATACATCAGACAGTCCACACTTGGAAGAAACCCTATATGTGTAGTGTGTGTGGGAAAGCATTCAGGTTTAGCTTCCAGCTCAGTCAGCATCAGAGTGTCCATAGTGAAGGAAAATCCTAATAATGAGAAAGATACAGAAAACTCTTAAGGTTAATGCCAAAATGGATCAAGTATCATCAGATTCATCCATTGAAAAACCTCCAAGAGGGCATGAATATGGCAGAGTCTTCATATGGAAACAGTTTTTATTCTATTCAGTTTAAATCAGGAAAGGATGACCAGTTAAAGAGAAACATCCAAAAATAGCTTTGTTTTGTACCAACAGGAATTAGAAAATATAATGAAAAGATTTCGTTCCCAGCAGCATCAAGAAAAGTAGATTTTCTAGAAATAAACAGTTATGGAGGACTTGTATGGAGAAATTTAAGTCTTCACTGAGGGCCACTTTACAAAGGAAATTTGAATAAATGGAGAGAGAGAGAAGCCTTGTTGTTGGATAGGAAAACCCGTACTAAAGATACTCTACCTACATTAATTTATTTGTTTAATTTTTGACAACAAGCATGTATTACTTTTGAAAAGATGAAAAATAAAGATTTATTTAAAAAAGGAAAAAAAAAAAAAAAAAAAAAAAAAAAAAAAAAC


>ZNF155 [GenBank: AF187986]
CTGCTACTTAACAAGGTGGTTTGAGCCAAGTATGTGTCTTGCGGGTCCTTGCGAAAGTGAACCAGTTATTAGGGGCGCCTAACGACCTGGGGGGTCAACTAAGGGGACTGTGGCACGTTTCAGGCAGGATTCCTCCTTCATTCAAACTGCATCACCCAGGAGTCTGCAAATTCCCCAAAGTAGGAGGAAAAATGACCACATTCAAGGAGGCAGTGACCTTCAAGGATGTGGCTGTGGTCTTCACTGAGGAGGAGCTGGGGCTGCTGGACCCTGCCCAGAGGAAGCTGTACCGAGATGTGATGCTGGAGAACTTCAGGAACCTGCTCTCAGTGGGGCATCAACCGTTCCACCAAGATACTTGCCACTTCCTAAGGGAAGAAAAGTTTTGGATGATGGGGACAGCAACCCAAAGAGAAGGGAATTCAGGAGGCAAGATCCAAACTGAGTTGGAGTCTGTTCCAGAAGCAGGAGCACATGAAGAGTGGTCCTGCCAGCAAATCTGGGAACAAATTGCAAAAGACTTAACCAGGTCTCAGGACTCTATCATAAATAACTCTCAGTTCTTTGAAAATGGTGATGTCCCCTCCCAGGTTGAAGCAGGACTACCCACAATTCATACAGGACAGAAACCTTCCCAGGGTGGGAAGTGTAAACAGTCCTTCAGTGATGTTCCCATCTTTGATCTTCCTCAGCAGTCATACTCAGAAGAGAAGTCTTATACATGTGATGAGTGTGGAAAAAGCATCTGTTACATCTCAGCTCTTCATGTTCATCAGAGAGTCCACGTGGGAGAGAAACTCTTTATGTGTGATGTGTGTGGCAAGGAATTTAGTCAAAGCTCACATCTGCAAACTCATCAGAGAGTCCACACTGGAGAGAAACCATTCAAATGTGAGCAATGTGGGAAAGGTTTCAGTCGTAGATCAGCACTTAATGTTCATCATAAATTACACACAGGAGAGAAACCTTACATTTGTGAGGCATGTGGGAAGGCTTTCATTCATGATTCCCAGCTTAAGGAACATAAGAGAATCCATACTGGGGAGAAACCATTCAAATGTGATATATGTGGTAAGACCTTCTATTTTAGGTCAAGACTTAAGAGCCATTCCATGGTTCACACAGGAGAAAAACCATTTAGGTGTGATACATGTGATAAGAGCTTTCATCAGAGATCAGCACTTAATAGGCATTGCATGGTCCACACAGGAGAGAAACCGTACAGATGTGAGCAGTGTGGAAAAGGCTTTATTGGTAGGCTAGATTTTTATAAGCATCAGGTGGTCCACACAGGAGAGAAACCATATAATTGTAAAGAATGTGGGAAGAGCTTCAGATGGTCCTCATGCCTTTTGAACCATCAGCGAGTCCACAGTGGAGAAAAAAGCTTCAAATGTGAAGAATGTGGGAAGGGATTTTATACAAATTCACAACTGTCTTCCCATCAGAGATCCCACAGTGGTGAAAAGCCATATAAATGTGAGGAGTGTGGGAAGGGCTATGTTACTAAGTTTAATCTTGACTTGCACCAGAGGGTCCACACGGGAGAGAGACCTTATAATTGTAAGGAATGTGGGAAGAACTTTAGCCGGGCCTCAAGTATTTTGAATCATAAGAGACTCCACTGCCAGAAAAAACCATTCAAATGTGAGGACTGTGGAAAGAGGCTTGTACACAGGACATACCGTAAAGACCAGCCGAGAGACTATAGTGGGGAAAACCCATCCAAATGTGAGGATTGTGGGAGACGCTACAAGAGGCGCTTGAATCTGGATATACTTTTATCATTATTTCTAAATGACACATAACTGTTCATATTTATGGGGTACAACGTGCTATTTTAATGTGTGCATACAATTTATAGTGATCCAATCAGTGTAATTGGTGT

>ZNF221 [GenBank:AF187987]
GCGGGAGCTACGGCTGCGGGAGTTACCCTTTATTCTGTGATATTTTGAGCTGTTCTGCGTCTCGCAGGCCCTTCTGAATTTCCTGGACCTACTCTCGGAACCCTCAACGAGCTAGCGATAGATTTAGAGGAAAGGAAGCACCGTCGGAAAGCAAAGGGATTTCTGGAAGCTTGAACTTAAGAGTGATAACTTAGGGTACCTGGCAAAAGAAATTTCTGAGCAGCAAAGCATTCAAGAAGTGGCCTGGCTGCCTCACTGCTTCAAACAGCCTACAATTATATATGGGAGCAAAAAAATGACTTAAAGTTGGAATTTATATTTAAAAGGCATGATTTCACCTTCACTTGAACTGCTTCATTCAGGACTCTACAAATTCCCTGAAGTAGAAGGAAAAATGACCACATTCAAAGAGGCAGTGACATTCAAGGATGTGGCTGTGGTCTTCACTGAGGAGGAGCTGGGGCTGCTGGACCCTGCCCAGAGGAAGCTGTACCGAGATGTGATGCTAGAGAACTTCAGGAACCTGCTCTCAGTAGGGAATCAACCATTCCACCAAGATACTTTCCACTTCTTAGGGAAGGAAAAGTTTTGGAAGATGAAGACAACAAGCCAAAGAGAAGGGAATTCAGGAGGCAAGATCCAAATTGAGATGGAGACTGTTCCAGAAGCAGGACCACATGAAGAGTGGTCCTGTCAGCAAATATGGGAACAAATTGCAAGTGACCTAACCAGGTCTCAAAACTCCATAAGGAACAGCTCTCAGTTCTTCAAAGAAGGTGATGTCCCCTGCCAGATTGAGGCAAGACTATCTATAAGTCACGTGCAACAGAAACCTTACCGTTGTAATGAATGTAAACAGTCCATCAGTGATGTTTCTGTCTTTGATCTTCATCAACAATCACACTCAGGAGAGAAATCTCATACATGTGGTGAGTGTGGAAAAAGCTTCTGTTACAGCCCAGCCCTTCATATTCATCAGAGAGTCCATATGGGAGAAAAATGCTATAAGTGTGATGTGTGTGGTAAGGAATTTAATCAGAGCTCACATCTGCAAACTCATCAGAGAGTCCATACTGGAGAGAAACCATTCAAACGTGGGCAATGTGGGAAAGGCTTCCATAGTAGATCAGCACTTAATGTTCATTGCAAATTGCACACAGGAGAGAAACCTTATAATTGTGAGGAATGTGGGAAAGCCTTCATTCATGATTCACAGCTCCAGGAACATCAGAGAATCCATACTGGGGAGAAGCCATTCAAATGTGATATATGTGGTAAGAGCTTCCGTGTTAGATCAAGACTTAATAGGCATTCCATGGTTCACACAGGAGAAAAACCATTCAGATGTGATACATGTGGCAAGAACTTTCGTCAGAGATCAGCACTTAATAGTCATTCCATGGTCCACATAGAAGAGAAGCCATACAAATGTGAGCAATGTGGAAAAGGCTTCATTTGTAGGCGAGATTTTTGTAAGCATCAGATGGTCCACACAGGAGAGAAACCATATAATTGTAAAGAATGTGGGAAGACCTTCAGATGGTCCTCATGTCTTTTGAACCATCAGCAAGTCCACAGTGGACAAAAATCCTTCAAATGTGAAGAATGTGGGAAGGGATTTTATACAAATTCACGACGATCTTCCCATCAGAGATCCCACAATGGAGAAAAGCCATATAACTGTGAGGAGTGTGGTAAGGACTATAAAAGGAGGTTGGATCTTGAGTTTCACCAGAGGGTCCACACGGGTGAGAGACCCTATAATTGTAAGGAATGTGGCAAGAGCTTTGGCTGGGCCTCCTGTCTTTTGAAACATCAGAGACTCCACAGTGGGGAAAAACCTTTCAAATGTGAAGAGTGTGGAAAGAGATTTACTCAGAGTACACAACTTCATTCCCATCAGACATGCCATACTGGAGAAAAGCTATACAAATGTGAGCAGTGTGAGAAGGGGTACAACAGTAAATTTAATCTTGACATGCACCAGAGGGTCCACAGGGGAGAGCGACCCTATAATTGTAAGGAATGTGGAAAGAGCTTTGGCTGGGCTTCATGTCTTTTGAAACATCAGAGACTCCACAGTGGAGAAAAGCCATTGAAATCTGGAGTGTGGGAAGAGATCTACTCAGAATTCACAGCTTCATTTACATCAGTAAGTCTATGTGGGAGAAAAGCCATATAAATGTGAGAAGTGTGGGAAGGGCTTTGGCTGGGCCTCAACTCATCTGACCCATCAATTCTCCACAGCAGAGAAAAACCATTCAAATATGAGAACTGTGGGAAGAGCTTTGTACATAGATCATATCTTTTTTTTTTTTTTTGAGACAGAGTCTCACTCTTTCACCCAAGCCTGACTGCAGTGGCGCTATCTCAGCTCGCTGCAAGCTCCGCCTCCCAAGTTCACGCCATTCTCCTGCCTCAGCCTCCCGAGTAACTGGGACTACAGGTGCCCGCCACCACACCCAGCTAATTTTTTGTATTTTTAGTAAAAACGGGGTTTCACCGTGTTAGCCAGGATGGTCTCGATCTCCTGA

>ZNF223 [GenBank: AF187989]
AAAAAAACAAAACAAAACAAAAAACTCTGACAGAGATGTCTGGTTTGAGCCATGTCTGCGTCTTGAAGGACATTTTGAACGAACCCCCTTTGCTTGAGGCTCGCAACCACCCGATGATCGATGATCGTCTCAGGGGAAAAGAAGCCTTGGCGAAGAGCAGAGGTTTAGAGGCACAATTCTGCTTTCCCTGGAACTGTGTCATTCAGGACTCTGCAAATTCCCTAAAGTAGGAGGAAAAATGACCATGTCCAAGGAGGCAGTGACCTTCAAGGATGTGGCAGTGGTCTTCACTGAGGAGGAGCTGGGGCTGATGGACCTTGCCCAGAGGAAGCTGTATCGAGATGTGATGCTGGAGAACTTCAGGAACCTGCTGTCAGTGGGGCATCAACCATTCCACCGAGATACTTTCCACTTTCTAAGGGAGGAAAAGTTTTGGATGATGGATATAGCAACCCAAAGAGAAGGGAATTCAGGAGGCAAGATCCAACCTGAGATGAAGACTTTTCCAGAAGCAGGACCACATGAAGGGTGGTCCTGCCAGCAGATCTGGGAAGAAATTGCAAGTGATTTAACCAGGCCTCAAGACTCTACCATAAAGAGCTCTCAGTTCTTTGAACAGGGTGATGCCCACTCCCAGGTTGAGGAAGGAATATCTATAATGCACACAGGACAGAAACCTTCCAATTGTGGGAAGAGTAAACAATCCTTCAGTGATATGTCCATCTTTGATCTTCCTCAGCAAATACGCTCAGCAGAGAAGTCTCATTCCTGTGATGAGTGTGGAAAAAGCTTCTGTTACATCTCAGCACTTCATATTCATCAGAGAGTCCACCTGGGAGAGAAACTCTTTAAGTGTGACGTGTGTGGTAAGGAATTCAGTCAGAGTTTACATCTGCAAACTCATCAGAGAGTCCATACTGGAGAGAAACCTTTCAAATGTGAACAATGTGGGAGAGGCTTCAGATGTAGATCAGCACTTACAGTTCATTGCAAATTACACATGGGAGAGAAACATTATAATTGTGAGGCATGTGGGAGGGCCTTCATTCATGATTTCCAGCTTCAGAAACATCAGAGAATTCATACTGGGGAGAAGCCATTCAAATGTGAAATATGTGGTAAGAGCTTCTGTCTTAGGTCAAGTCTTAATAGGCATTGCATGGTCCACACAGCAGAGAAACTGTACAAATCTGAAAAGTATGGAAGAGGTTTCATTGATAGGCTAGATTTGCATAAGCATCAGATGATTCATATGGGACAGAAACCATATAATTGTAAAGAATGTGGGAAGAGCTTCAAATGGTCCTCATATCTTTTGGTCCATCAACGAGTCCACACTGGAGAAAAGCCATACAAATGTGAGGAGTGTGGGAAGGGCTACATTAGTAAGTCAGGTCTTGACTTGCACCATAGAGCCCACACAGGAGAGAGACCTTATAACTGTGATGACTGTGGGAAGAGCTTTAGACAGGCCTCAAGTATTTTGAATCATAAGAGACTCCATTGCCGAAAAAAACCATTCAAATGTGAGGATTGTGGAAAGAAGCTTGTATACCGGTCATACCGTAAAGACCAACAAAAAAACCACAGTGGAGAAAATCCATCCAAATGTGAAGACTGTGGGAAGCGCTACAAGAGGCGCTTGAATCTTGATATAATTTTATCATTATTTTTAAATGACACGTAAGTGTTGTACATATTTATGGGGTACAGTGTGATATTTAAATATATGTATATGATGTATAATGATCAAATCAGTGTAATTAGCACATTTATCACCTCAATTATCTCTTTTTTGTGTTGAGAAAATTAAAAATTCATTGTTCCAGCAGTTTGAAAATAAATTGTTGTCGATGATAGTCACCTTTAGTGCTGCAGAACAGCAGAACTTCTTCCTCTTATCTACCTGTACTTTTGCATCCATTAGCCAACCTTTGGCCATCCCACCTATCCCTTACCCTTCCCTGCCTCTAGAGCACTGTCTCACTACTTCTAGAAATCAGCTTTTAAGCTCCCATGTGATGAGAAGTTGA

>ZNF224 [GenBank: AF187990]
GAGTCCAAACATTCGGTGGAGTCGCGGACACTTCCGCTCGGGGACTGAGGTTGCTGCAGTTTTTCCGCGATAGTTTGGGGCAGTTCCGCGCGTTGCAGGCCCTTCTGAATTTCCTGGACCTACGCATTGGATCCTCAAAGAACTGCTGAATACCACTAGAAACATACCTGTAACCAGAGACAGCTGATTATAGCTTTCTGCAGCAAGGAAGCCCACGTACCAGGGGCTGTCTTGGCACAATTCTGCTTTCCCAGGAACTGCATCACTCAGGACTCTGCAAGTTTCCAGAAGTAAGAGGGAAAATGACCACGTTCAAGGAGGCAATGACCTTCAAGGACGTGGCTGTGGTCTTCACTGAGGAAGAGCTGGGGCTGCTGGACCTTGCTCAGAGGAAGCTGTATCGAGATGTGATGCTGGAGAACTTCAGGAACCTGCTCTCAGTGGGACATCAAGCATTCCACAGGGATACTTTCCACTTCCTAAGGGAAGAAAAGATTTGGATGATGAAGACAGCAATCCAAAGGGAAGGGAATTCAGGAGACAAGATCCAAACTGAGATGGAGACTGTTTCAGAAGCAGGAACACATCAAGAGTGGTCCTTCCAGCAAATCTGGGAAAAAATTGCAAGTGATTTAACCAGGTCTCAAGACTTGGTGATAAATAGCTCTCAGTTCTCCAAAGAAGGTGATTTCCCCTGCCAGACTGAGGCAGGACTATCTGTAATTCACACAAGACAGAAATCTTCCCAGGGCAATGGATATAAACCATCCTTCAGTGATGTCTCCCACTTTGATTTTCATCAACAATTACACTCAGGAGAGAAATCTCATACGTGTGATGAGTGTGGAAAGAACTTTTGTTACATCTCAGCCCTTCGTATTCATCAGAGAGTCCACATGGGAGAGAAATGCTATAAGTGTGACGTGTGTGGTAAGGAATTCAGTCAGAGTTCACATCTGCAAACTCATCAGAGAGTCCACACTGGAGAGAAACCGTTCAAATGTGTGGAATGTGGGAAAGGCTTCAGTCGTAGATCAGCACTTAATGTTCATCACAAATTACACACAGGAGAGAAACCTTATAATTGTGAGGAATGCGGGAAGGCCTTCATTCACGATTCCCAGCTTCAAGAACATCAGAGAATCCATACGGGGGAGAAGCCATTCAAATGTGATATATGTGGTAAGAGCTTCTGTGGTAGATCAAGACTTAATAGGCATTCCATGGTTCACACGGCAGAGAAACCATTCCGATGTGATACGTGTGATAAGAGCTTTCGTCAGAGATCAGCACTTAATAGTCATCGCATGATCCACACAGGAGAGAAACCATACAAATGTGAGGAGTGTGGAAAAGGCTTTATTTGTAGGCGAGATCTTTATACGCATCATATGGTCCACACGGGAGAAAAGCCATATAATTGTAAAGAGTGTGGGAAGAGCTTCAGATGGGCCTCGTGTCTTTTGAAACATCAGCGAGTCCACAGTGGAGAAAAACCATTCAAATGTGAAGAATGTGGGAAAGGATTTTACACAAATTCACAATGCTATTCCCACCAGAGATCCCATAGTGGAGAAAAACCATACAAATGTGTGGAGTGTGGGAAGGGCTACAAAAGGAGGTTGGATCTTGACTTTCACCAGCGCGTCCATACAGGAGAGAAACTGTATAATTGTAAGGAATGTGGGAAGAGCTTTAGTCGGGCCCCATGTCTTTTGAAACATGAGAGACTCCACAGTGGAGAAAAACCATTCCAATGTGAAGAGTGTGGGAAGAGATTTACTCAAAATTCACATCTTCATTCCCATCAGAGAGTTCACACTGGAGAAAAGCCATACAAATGTGAGAAGTGTGGAAAGGGCTACAATAGTAAGTTTAATCTTGATATGCACCAGAAGGTCCACACAGGAGAGAGACCATACAATTGTAAGGAATGTGGGAAGAGTTTTGGCTGGGCCTCGTGTCTTTTGAAACATCAGAGACTGCGCAGTGGGGAAAAACCTTTCAAATGTGAAGAGTGTGGGAAAAGATTTACTCAGAATTCACAGCTTCATTCTCATCAAAGAGTGCACACTGGAGAAAAGCCATACAAATGTGATGAGTGTGGGAAGGGCTTCAGCTGGTCCTCAACTCGTCTGACCCATCAGAGACGCCACAGCAGAGAAACACCTCTCAAATGTGAGCAGCATGGGAAGAACATTGTACAGAATTCATTCTCTAAAGTGCAAGAAAAAGTTCACAGTGTAGAAAAGCCATACAAATGTGAGGACTGTGGGAAGGGCTACAACAGGCGCTTGAATCTTGATATGCATCAGAGGGTCCACATGGGAGAGAAAACATGGAAGTGTAGGGAGTGTGATATGTGCTTTAGTCAGGCCTCAAGCCTTCGACTTCATCAGAATGTTCATGTTGGAGAAAAACCTTAGTGATGTGATGGTGCAATAAAGTCTTCACTCAGTCTTCATG

>ZNF225 [GenBank: AF187991]
TCCAGACACTCAGTGGAGTAGCGGGCCACTTCCGCTCCGTTACTGTGAGGTTGCTGCAGTTTTGTCCCTGGTAGTTTGGGTCAGTTCCGCGAGACCCTTCTGAATTTCCTGGACCTACGCACTGGATCCTTAACGAACTGGTGATCGAATTAGGGGAAAAGAAACCTCGTCGGAGAGCAGAGGCAGGATTCTGCTTTCCCTTGGACTGTATCACTCAGGACTCTGAATATTCCCTGAAATAGGAGGAAAAATGACCACGTTGAAGGAGGCAGTGACCTTCAAGGACGTGGCTGTGGTCTTCACTGAGGAAGAGCTGAGGCTGCTGGACCTTGCCCAGAGGAAACTGTACCGAGAAGTGATGCTGGAGAACTTCAGGAACCTGCTCTCAGTGGGGCATCAATCACTCCACAGAGATACTTTCCACTTCCTAAAGGAAGAAAAGTTTTGGATGATGGAGACAGCAACCCAAAGAGAAGGGAATTTAGGAGGCAAGATCCAAATGGAGATGGAGACTGTTTCAGAATCAGGAACACATGAAGGCTTGTTCAGTCATCAAACCTGGGAACAAATTTCAAGTGACTTAACCAGGTTTCAAGACTCCATGGTAAACAGCTTTCAGTTCTCCAAACAAGATGATATGCCCTGCCAGGTTGATGCAGGACTATCTATAATTCACGTTAAGACAGAAACTTCTGAGGGTAGGACGTGTAAAAAGTCCTTTAGTGATGTCTCCGTCCTTGATCTTCATCAACAACTACAGTCAAGAGAGAAGTCTCATACATGTGATGAATGTGGAAAGAGTTTCTGTTATAGCTCAGCTCTTCGTATTCATCAGAGAGTTCACATGGGGGAGAAACTCTATAATTGTGATGTGTGTGGTAAGGAATTCAATCAGAGCTCACATCAGCAAATTCATCAGAGAATCCACACTGGAGAGAAACCATTCAAATGTGAGCAGTGTGGGAAAGGCTTTAGTCGTAGATCAGGACTTTATGTTCATCGTAAATTACACACAGGAGTGAAACCTCATATTTGTGAGAAATGTGGGAAGGCCTTCATTCATGATTCCCAGCTTCAGGAACATCAAAGAATCCATACTGGGGAGAAGCCATTCAAATGTGATATATGTTGTAAGAGCTTCCGTAGTAGAGCAAATCTTAATAGGCATTCCATGGTTCACATGCGAGAGAAACCATTCAGATGTGATACATGTGGTAAGAGCTTTGGTCTGAAATCAGCACTTAATAGTCATCGCATGGTCCACACAGGAGAGAAACGGTACAAATGTGAGGAATGTGGAAAACGCTTCATTTATAGGCAAGATCTTTATAAGCATCAGATAGACCACACAGGGGAGAAGCCATATAATTGTAAAGAATGTGGAAAGAGCTTCAGATGGGCCTCAGGTCTTTCAAGACATGTGCGAGTCCACAGTGGAGAGACAACATTCAAATGTGAAGAATGTGGGAAGGGATTTTATACAAATTCACAACGTTATTCTCACCAGAGAGCGCACAGTGGAGAAAAGCCATATAGATGTGAGGAGTGTGGGAAGGGCTACAAAAGGAGGTTGGACCTTGACTTTCATCAGAGGGTCCACAGAGGAGAGAAACCCTATAATTGTAAGGAATGTGGGAAGAGCTTTGGCTGGGCCTCGTGTCTTTTGAATCATCAGAGAATCCACAGTGGAGAAAAACCATTTAAATGTGAAGAATGTGGGAAAAGATTTACTCAGAATTCACAACTTTATACCCATCGTAGAGTCCACAGTGGAGAAAAACCATTCAAATGTGAAGAGTGTGGGAAAAGATTTACTCAGAATTCACAACTTTATTCTCATCGCAGAGTCCACACTGGAGTAAAGCCATACAAATGTGAAGAGTGTGGGAAGGGCTTCAACAGTAAGTTTAATCTTGACATGCACCAGAGGGTCCACACCGGAGAGAGACCTTATAATTGTAAAGAATGTGGGAAGAGCTTTAGCCGGGCCTCAAGTATTTTGAATCATAAGAGACTCCATGGTGATGAAAAGCCATTCAAATGTGAAGAGTGTGGGAAGAGATTTACTGAGAATTCACAGCTTCATTCCCATCAGAGGGTTCACACTGGGGAAAAGCCATACAAATGTGAGAAGTGTGGAAAGAGCTTCAGATGGGCCTCAACTCATCTAACCCATCAGAGACTCCACAGTAGAGAAAAACTACTTCAATGTGAGGACTGTGGGAAGAGCATTGTGCACAGTTCATGCCTTAAAGACCAACAAAGAGACCAAAGTGGAGAGAAAACATCTAAATGTGAGGACTGTGGGAAGCGCTACAAGAGGCGCTTGAATCTTGATACGCTTTTGTCATTATTTTTAAATGACACATAACTGTTGTACTCATTTATGGGGTACAGTGTGATAGTTAATGCAAGTATACATGTGTAATGATCAAATCAGTGTAATTAACATACCTATCACTCAAC


>ZNF226 [GenBank: AF228418]
GGAACCTTTCAAACTTCCCCGAAATGCACTTGTGGTCAGCAGCTAGCGGTCTTTTGTTTGGGGAAAAAAGGAGTAGCGGCTAAGAGCGGAACCCTGACTTCTCAAAAAGCACTGCACAGAGGAGGAGGCAGCAGAACCCCATGGGCTGTTTTGGGAGAGGGACTGAGTACAGTGCAACACTACTCAAACTGTGGTTCACAAAGCAGCAACATTGGCATCACCCAGGTAGAAAGGCTCATTCTTGAGCCTACCGAAGGCCTTCTGATTTAGCATTTTTGCAGATAGGACCCAGAAATCTGCATTTCAACAAAATCCCAAGTTCAGCTTCTTAGGACTCTGCACTTCCCCAGAAGGAAGAATTAAAAATGAATATGTTCAAGGAAGCAGTGACCTTCAAGGACGTGGCTGTGGCCTTCACGGAGGAGGAATTGGGGCTGCTGGGCCCTGCCCAGAGGAAGCTGTACCGAGATGTGATGGTGGAGAACTTTAGGAACCTGCTGTCAGTGGGGCATCCACCCTTCAAACAAGATGTATCACCTATAGAAAGAAATGAGCAGCTTTGGATAATGACGACAGCAACCCGAAGACAGGGAAATTTAGGAGAGAAAAATCAAAGTAAGTTAATTACTGTTCAAGACAGAGAATCAGAAGAAGAGCTTTCTTGTTGGCAAATCTGGCAACAAATTGCAAATGACTTAACCAGGTGTCAAGACTCCATGATCAATAATTCTCAGTGTCACAAACAAGGTGATTTCCCTTACCAGGTAGGGACAGAACTGTCTATTCAAATTTCTGAAGATGAGAACTATATAGTAAATAAAGCAGATGGTCCCAATAATACTGGGAATCCAGAGTTTCCTATCTTGAGAACCCAGGATTCTTGGAGGAAAACATTCCTGACTGAGTCACAGAGATTGAACAGAGATCAGCAAATTTCCATAAAAAATGAATTATGTCAATGTAAGAAGGGTGTTGATCCCATCGGTTGGATTTCACATCATGATGGTCATAGAGTACACAAAAGTGAAAAATCTTATAGACCCAATGATTATGAAAAAGACAACATGAAGATTTTGACATTTGATCACAATAGCATGATTCACACAGGACAGAAATCGTACCAGTGTAATGAGTGTAAAAAACCCTTCAGTGATCTCTCCAGCTTTGATCTTCATCAGCAGTTACAATCAGGAGAGAAGTCTCTTACATGTGTTGAGCGTGGAAAAGGCTTCTGTTACAGCCCAGTTCTTCCTGTTCATCAGAAAGTACATGTGGGAGAAAAACTTAAGTGTGATGAGTGTGGTAAGGAATTCAGTCAGGGCGCTCATCTACAGACCCATCAGAAAGTCCACGTGATAGAGAAACCATACAAATGTAAGCAATGTGGGAAAGGTTTCAGTCGTAGATCAGCACTTAATGTTCATTGCAAGGTCCACACGGCAGAGAAACCTTATAATTGTGAGGAGTGTGGGAGGGCCTTCAGTCAGGCCTCTCATCTTCAGGACCATCAGAGACTCCACACTGGGGAGAAGCCATTCAAATGTGATGCATGTGGTAAGAGCTTCAGTCGGAATTCACATCTTCAATCCCATCAAAGAGTTCATACAGGAGAGAAACCATACAAATGTGAGGAGTGTGGTAAGGGCTTCATTTGTAGCTCAAATCTTTACATTCATCAGAGAGTCCACACAGGAGAAAAACCCTATAAATGTGAGGAATGTGGTAAAGGCTTTAGTCGGCCTTCAAGTCTTCAGGCCCATCAGGGAGTTCACACTGGAGAGAAGTCATACATATGTACTGTATGTGGGAAAGGCTTTACTCTGAGTTCAAATCTTCAAGCCCATCAGAGAGTCCACACTGGAGAGAAGCCATACAAATGCAATGAGTGTGGGAAGAGCTTCAGGAGGAATTCCCATTATCAAGTTCATCTAGTGGTCCACACAGGAGAGAAACCCTATAAATGTGAGATATGTGGGAAGGGCTTCAGTCAAAGTTCGTATCTTCAAATCCATCAGAAGGCCCACAGTATAGAGAAACCTTTTAAGTGTGAGGAGTGTGGGCAGGGTTTCAATCAGAGCTCACGACTTCAGATTCACCAGCTGATCCATACGGGTGAGAAACCATACAAATGTGAAGAGTGTGGCAAGGGATTTAGTCGTAGAGCAGATCTTAAAATTCACTGTAGGATCCACACAGGAGAGAAACCATATAATTGTGAGGAGTGTGGGAAGGTCTTCAGGCAGGCCTCAAATCTTTTGGCCCATCAGAGAGTCCACAGTGGAGAAAAACCATTCAAATGTGAAGAATGTGGGAAGAGTTTCGGTCGGAGTGCACATCTTCAAGCCCATCAAAAAGTCCACACTGGAGATAAGCCATACAAATGTGATGAGTGTGGGAAGGGCTTCAAGTGGAGCTTGAACCTTGACATGCATCAGAGGGTGCACACAGGAGAAAAACCATATAAATGTGGGGAGTGTGGTAAGTACTTCAGTCAGGCCTCAAGTCTTCAACTTCATCAGAGTGTCCACACAGGAGAGAAACCATACAAATGTGATGTGTGTGGTAAAGTCTTCAGTCGGTCTTCACAACTACAGTCTCATCAGCGAGTTCACACTGGGGAGAAACCTTATAAATGTGAGATATGTGGTAAGAGCTTCAGTTGGCGATCAAATCTTACAGTTCATCACAGAATCCATGTTGGTGATAAATCCTATAAAAGTAATAGGGGTGGTAAGAACATCAGAGAATCCACACAGGAAAAAAAATCTATAAAATGATTCTTTGTG


>ZNF228 [GenBank: AF198358]
GGCCTTCGTTCCTCCCAGAAAGGTCTCTGCCTTTCTCTAGAAAAAGAAAGAAAAGAATGACCGTTTCCAAGGAGATGGTGACATTCAAGGATGTTGCTGTGGTCTTCACTGAGGAGGAGCTGGGGCTGCTGGACTCTGTCCAGAGGAAGCTGTACCGAGATGTGATGCTGGAGAACTTCAGGAACCTGCTCTTAGTAGCACATCAGCCCTTCAAGCCAGACCTAATATCCCAGCTGGAGAGAGAAGAAAAGCTTTTGATGGTGGAGACAGAAACCCCAAGAGATGGATGTTCAGGAAGGAAGAATCAACAAAAGATGGAGAGTATTCAGGAAGTAACAGTAAGCTACTTTTCCCCCAAAGAGCTTTCCTCCCGTCAGACCTGGCAACAAAGTACAGGTGGGTTAATCAGGTGTCAAGATTTCCTGAAAGTTTTTCAAGGGAAGAATTCTCAGTTGCAAGAACAAGGTAATTCCCTCGGCCAGGCTTGGGCAGGAATACCAGTTCAGATTTCTGAAGATAAGAACTATATATTCACTCATATAGGGAATGGCTCCAATTATATAAAAAGTCAAGGGTATCCATCTTGGAGGGCACATCATTCTTGGAGGAAAATGTATCTGAAAGAGTCACATAATTATCAGTGTAGATGTCAGCAAATTTCCATGAAAAATCATTTCTGTAAGTGTGACAGTGTCAGTTGGCTCTCACATCACAATGATGAACTGGAAGTACACAGAAAAGAAAACTACAGCTGCCATGACTGTGGAGAAGATATCATGAAGGTATCATTACTTAATCAGGAGTCAATTCAAACAGAGGAGAAGCCCTATCCATGTTCTGGGTATAGAAAAGCCTTCAGTAATGACTCCAGCTCTGAAGTTCATCAGCAGTTCCACTTGGAAGGGAAGCCCTATACATACAGTTCATGTGGAAAGGGCTGTAATTATAGTTCACTTCTTCATATTCATCAAAATATTGAGAGAGAAGATGATATTGAGAATTCACATCTGAAATCCTATCAGAGAGTGCATACAGAGGAGAAACCATGCAAATGTGGTGAATATGGTGAGAACTTCAATCACTGTTCCCCTCTTAACACTTATGAACTTATCCACACAGGTGAGATGTCCTATAGGCACAACATTTATGAGAAAGCCTTCAGTCATAGCTTAGACCTTAATAGTATTTTTAGGGTCCATACTAGGGATGAACCCCATGAATATGAGGAAAGTGAGAATGTCTTTAATCAGAGTTCATGTCTTCAAGTCCATCAAAAAATCCACACTGAAGAGAAACTATACACAGATATAGAGTATGGAAAGAGTTTCATTTGTAGTTCAAATCTTGACATTCAGCATAGGGTTCATATGGAAGAGAATTCATATAATTCCCAGGAGTGTGGTAATGGCTTCAGTCTGGCCTCACATTTTCAGGACCTTCAGATAGTCCACACTAAGGAACAACCATATAAACGCTATGTGTGTAGTAACAGCTTCAGCCATAATTTACATCTTCAAGGTCACCCAAAAATTCACATTGGAGAGAAACCACGTAAGGAGCATGGGAATGGCTTCAACTGGAGCTCAAAACTTAAAGATCATCAGAGAGTCCACACTGGACAGAAGCCATACAAATGCAATATATGCGGCAAAGGTTTCAATCATAGATCAGTTCTGAATGTTCATCAGAGAGTCCACACCGGAGAGAAACCTTATAAATGCGAGGAATGTGATAAGGGATTCAGTCGGAGTTCATATCTTCAAGCCCATCAGAGAGTCCACACTGGAGAAAAACCTTATAAATGTGAGGAATGTGGGAAGGGGTTCAGTCGAAATTCATACCTTCAAGGCCATCAGAGAGTTCACACTGGAGAAAAACCATACAAGTGTGAGGAGTGTGGGAAGGGCTTCAGTCGGAGTTCACACCTTCAAGGCCATCAGAGAGTCCACACTGGAGAAAAACCATTCAAATGTGAGGAATGTGGGAAGGGGTTCAGTTGGAGCTTTAATCTTCAAATTCATCAGAGGGTTCACACAGGAGAAAAACCCTATAAATGTGAAGAATGTGGTAAAGGCTTCAGTAAGGCCTCAACACTTTTGGCCCATCAGAGGGTCCACACGGGAGAGAAGCCATACCAATGTGATGAGTGTGGTAAGAGTTTCAGTCAGAGATCATACCTTCAGAGTCATCAGAGTGTCCATTCTGGAGAAAGACCATATATATGTGAGGTATGTGGAAAGGGCTTCAGTCAGAGAGCATATCTTCAAGGTCATCAGAGAGTCCACACTAGAGTGAAACCGTATAAATGTGAGATGTGTGGGAAGGGCTTTAGTCAGAGTTCGCGCCTTGAAGCACATCGGAGGGTTCACACAGGAGGGAAACCATACAAATGTGAGGTGTGTACAAAGGGTTTCAGTGAGAGTTCACGCCTTCAAGCACACCAAAGGGTTCATGTGGAAGGGAGACCCTATAAATGTGAACAGTGTGGTAAGGCTTTCAGTGGGTATTCAAGTCTTCAAGCCCATCACAGAGTCCACACAGGAGAGAAACCATACAAATGTGAGGTATGTGGAAAGGGCTTCAGTCAGAGATCAAATCTTCAGGCTCACCAGAGAGTCCACACAGGAGAGAAACCATACAAATGTGATGCATGTGGTAAGGGTTTCCGTTGGAGCTCAGGTCTTCTCATTCATCAAAGAGTCCATAGTAGTGATAAATTCTATAAAAGCGAAGACTATGGTAAGGACTACCCTTCATCAGAGAATCTACACAGAAATGAAGATTCTGTTTTGTTTTGAAGTCCTCAAATGGGAGCTGAAATTTTCCAGTCACTAGAGTTCTTTCGGTAGAAAAAGAATTTTTAAAAATTAAAATGTAATGTTGCTGCCCAACTTCAACATTCATAATGGCCAGGAGACCACACAACAGAGACATTTAATGAGAGGGGTTTCACGAGGGATTTTGTTAGAACTTTAACATCAGTCATTGCACAGGAGCTAAGGCTTATAAAATATGAGTATGGTCAGGAATTTAATAAAAGTACAGTGATGAGCATGCCTAAATCCATGTTCTCTAGAATGTAAGCTTGGTGAAAGAAGGGACTTTGTTCATTGCTAGTCTACAAAACTAGGACATTTCATAACGCAATGTAGGTGCTCAGTACTTTTTGCTAAATAACTAAAAGTATGTAAAGGTAAAAGGTTTGAAATTTTTAAGCGCATTTTTTAAAACTTAAAACGTATTTGGAGGAGAAATCCTGCAAGTAATCTTAATAAAGTCATTTCAAGTAAATATTTGAAATGTAGAACAATAAGCATTATGATGATCTGAAATAACATTAATTTGATTGTAACCCTATTGGGTTAGTTCCCACCCTTTGTTCCATCTTGAGCAGAGATGTGCTGCATTTCTTAACGTTTGGTCTCTATACATTATACCATCTGCTGAAGTTTAGCTTTGTGTGTGTGTATATGTACCCATGTGTGCACTTTAAATATCAATACAAACTGATAAACAATGTTTCTGGTTAATGTTGCAAAAATGGAACACTGTATTTTACATAATTGATTTTCTACTCCTGCCCTGTGAATTAAGGCAGAGTTTTACTGTAATATTTGCAAGTATCCAAAATGGTTACTGATGACAAATACTTTTTAATAAATGTCAAAATCACT


>Xfin [Genbank:X06021alt; GenBank:EU277665M; Xenopus laevis]
TGCCTGGCACATACACGTTACTCTCATCCTCCTCAAGCGCCGGACTAAGATGCTGCCGCGCTGAATTGTGGGAAATAGAGTTCCAGGACGTCTCCAAGCAACGCCGAGCGCCACTACCGGAACTACATTTCCCGGGAACGCGCGTTTCCCTGGCAGCGGCAGAATAACGAGTGAGGTACCGACTTCCTTTGTCTTTTTCCCGGAGCCCCTCCCTTCCTGAGGCGAATCGCTCATATTGTCAATGAAAGAGAGAGAGGGGCAGCCCCGCACCAACCTCCCGTGTCCAACACTCGCCATGGCTGGGCCATTGCTCCCTCCTCAGATGGTGCCGACGTTCGACGATGTGGCGGTGTATTTCTCCCGGAGCGAATGGAAGAGCCTAAGTGCCTCCcAGAGGGAGATGTACAAGAGCGTCATGACTGAGAACTACCAGTGTGTGCTTTCCCTGGGTTATCCAATCAGAAAGCCGGAAATTGTATCTATGATGGAGGTGGGAGAAGAGCTGTGGTCCAAGAATGATTCTGCTCGTCCTGGACAGAAGGAGGTGGAGGGTGAAACACCCAAGGAGTCTGATTGGGCAGCAGAGAATTGCAAGCGAGCACAAATGCATAAAGAGGTGCTGGATCTTGATACTTTGGCTGCAGTAAAGTCGGAGCCAGTGGAGGAGGGAAGTAACTCGGCAAAGAAGAGCCATATCTGTAGTCACTATGGCAAGCTCTTTAGCTGTTATGCAGCTGTGGTCAGACACCAAAGGATGCACCAACTGCAGAAGTCGCATCACTGTCCCCACTGCAAGAAAAGCTTCGTACAGAGGTCTGACTTCATTAAGCACCAAAGGACCCACACTGGAGAGAGGCCGTATCAGTGCGTGGAGTGCCAGAAGAAATTCACAGAGCGATCGGCCCTCGTCAACCACCAGAGGACGCACACGGGTGAGAGACCTTACACCTGCCTGGATTGCCAAAAAACCTTTAATCAGAGATCAGCCCTAACCAAGCACCGCAGGACGCACACTGGGGAGAGACCTTACCGCTGCTCAGTGTGTAGCAAGAGCTTCATCCAGAACTCCGACCTGGTGAAACACCTGAGGACGCACACCGGGGAGAAACCATACGAATGTCCTTTGTGCGTTAAAAGGTTTGCCGAAAGCTCGGCACTGATGAAGCACAAGAGGACGCACAGCACACATCGGCCCTTTAGGTGCTCAGAGTGCAGCAGAAGTTTCACACACAATTCTGATCTCACCGCACACATGAGGAAACATACAGAGTTTAGAAACGTTCTCAATCTAGACTCTGTAGTCGGTACCGACCCATTAAGCTCACAGAATGTTGCCTCCAGTCCGTATTCCTGTTCCAAATGCAGGAAGACATTCAAACGGTGGAAGTCTTTCCTGAACCACCAGCAGACACATTCCAGAGAGAAACCCTATCTGTGCTCCCATTGCAACAAAGGCTTCATTCAGAACTCTGACCTGGTGAAGCACTTTCGCACCCACACCGGGGAGAGGCCGTATCAGTGCGCTGAGTGTCACAAAGGCTTTATCCAGAAATCTGACCTTGTGAAACACTTAAGAACCCACACAGGGGAAAAACCCTTCAAGTGCTCCCACTGCGACAAAAAGTTCACAGAGAGATCGGCACTGGCAAAGCATCAAAGGACTCACACTGGGGAGAAACCATACAAGTGCAGCGACTGTGGCAAAGAGTTCACTCAACGCTCCAATCTAATTTTGCACCAGAGGATCCACACTGGTGAGCGGCCATATAAATGCACCCTCTGTGACAGAACTTTCATCCAGAACTCAGACTTGGTGAAACATCAGAAGGTGCACGCAAATTTGCCGCTCTCCGACCCTCATACCGCAAATTCTCCACACAAATGTTCCAAATGTGACCTGACTTTTAGCCACTGGTCGACCTTTATGAAGCATTCAAAGTTGCACAGTGGGGAGAAGAAGTTTCAGTGTGCAGAGTGCAAAAAAGGCTTCACCCAGAAGTCTGATCTGGTGAAACACATCCGTGTTCACACCGGGGAGAAACCCTTCAAATGCCTTCTGTGTAAGAAAAGCTTCTCCCAGAATTCTGACCTGCACAAACACTGGAGAATTCACACTGGAGAGAAGCCCTTCCCGTGTTACACATGTGATAAAAGCTTCACAGAGAGATCAGCTCTTATTAAACACCACAGAACTCACACAGGGGAGCGGCCGCACAAGTGCAGCGTGTGCCAGAAAGGCTTCATCCAGAAATCTGCACTCACCAAGCACAGTAGGACCCACACAGGAGAGAAACCCTACCCCTGCACCCAGTGTGGCAAGAGTTTCATTCAGAACTCCGACTTGGTGAAACACCAGAGGATCCACACAGGAGAGAAACCATATCACTGTACCGAATGTAATAAGCGATTTACCGAAGGCTCGTCACTGGTGAAGCACCGGCGAACCCACTCGGGGGAGAAACCATATCGGTGCCCACAATGTGAGAAAACCTTCATCCAAAGCTCAGACCTTGTTAAACACCTTGTGGTTCACAATGGAGAAAACCCACCTGCTGCCACGGCGTTCCATGAAATCCTCATAAGACGGGAGAATCTGACAAGGAGCGAGCCTGATCCCTATCCGTGCACAGAGTGCGGCAAGGTCTTCCACCAGAGACCCGCCCTCCTCAAACACCTGAGAACTCACAAGACCGAAAAACGGTATCCATGCAACGAGTGTGACAAGAGTTTCTTCCAGACCTCTGACTTGGTTAAACACCTGCGAACGCACACAGGGGAGCGGCCGTACCATTGTCCTGAGTGCAACAAAGGTTTCATCCAGAACTCGGACCTGGTGAAGCACCAGAGGACACACACTGGGGAGAGACCCTACACCTGCAGCCAATGCGACAAAGGCTTCATACAGAGATCTGCCCTGACAAAGCACATGCGAACTCACACCGGGGAGAAACCATATAAGTGTGAGCAGTGCCAGAAGTGCTTCATTCAGAACTCTGATCTGGTCAAGCACCAAAGGATCCACACTGGGGAGAAACCCTATCACTGTCCGGACTGTGACAAGCGCTTCACTGAAGGCTCTTCACTCATTAAGCACCAAAGGATTCACAGCAGGATCAAACCCTACCCATGCGGCGTGTGCGGCAAAAGCTTCAGCCAAAGCTCCAACCTCCTCAAACACCTGAAATGCCACAGTGAACAGAACCCTCCTGTTGCTCTTAGCTCAGAACTGGGATTTGTAGCGGAGACACAGACACATCCTGATCCGGTGGATCATATTGTTTATGGTGATACCGCTAGTTACATTTCACCTGAGGCCGCGGGGGAGAGGTCGTTTAAATGTAACGACTGTGGGAAATGTTTCGCTCACAGATCGGTACTTATCAAGCACGTGCGAATCCACACGGGGGAACGGCCATACAAGTGCTCCCAGTGCACCAGAAGCTTCATCCAGAAGTCGGACCTTGTCAAGCATTATCGAACCCACACCGGGGAGAGGCCGTACAAATGCGGGTTGTGCGAGAGGAGCTTTGTGGAAAAGTCTGCCCTTTCCAGACACCAGAGGGTTCACAAAAATGAGAGCCCGGTTCTGAATTCTGCCATGGAACAGCAACAGGTTACTTACTGGGGCGAGTCTAAAGATGATCCAAACAGTCTGGTTCCACAGCTCCATGTCATTAAAGAAGAAGAGAGTCCACATATAGTAAACGCATACAGCCCACTTAGTATTCTGCAGAGCTATTTCCCCCCTATACTAGAACCCAAGGGAACCCCACGATATTCCTGCTCAGAGTGCGGCAAATGCTTCACACACAGGTCCGTGTTTCTCAAGCACTGGCGCATGCACACGGGGGAACAGCCGTACACTTGCAAGGAGTGTGGGAAGAGCTTCAGTCAGAGTTCTGCCCTAGTGAAACATGTGCGCATACACACTGGGGAGAAACCCTATCCCTGTTCCACCTGTGGGAAGAGCTTCATTCAGAAATCGGACCTGGCCAAACACCAGCGGATTCACACGGGGGAGAAGCCTTACACATGTACTGTGTGCGGGAAGAAATTCATTGACCGCTCATCAGTTGTCAAACACTCCAGAACCCACACGGGAGAGAGGCCATATAAGTGCAACGAGTGCACCAAAGGCTTTGTCCAGAAGTCCGATCTGGTGAAACACATGAGAACTCACACGGGGGAGAAACCCTATGGGTGCAACTGCTGTGACCGCAGCTTTAGCACCCACTCTGCCTCAGTCCGGCACCAGCGCATGTGCAACACCGGGAGACCCTATCAGGATGAGGAGTATGAGAACAGCTTGTTTTATAGCGCTGATATCACTTGGAAGGGAGATTATGCACAGTTGCTCCAAATACCATGTGGCTTAGAAGAACCAATGAAGGCAATTGGTTGGATTTCTGAAGTCGCTCTGTAAATAAATTTGTTTCATTTCATTGG
