# Supplementary material for: The ancient mammalian KRAB zinc finger gene cluster on human chromosome 8q24.3 illustrates principles of C2H2 zinc finger evolution associated with unique expression profiles in human tissues
Source: BMC Genomics. 2010 Mar 26;11:206. doi: 10.1186/1471-2164-11-206 (PMC2865497; doi:10.1186/1471-2164-11-206)
Supplement: Additional file 2 — Amino acid sequences. Listing of all amino acid sequences used for analysis. [file 1471-2164-11-206-S2.RTF]

Additional file 2									

Note: The list contains the amino acid sequences that were used for the analyses and relate to the gene models defined in this publication (see also cDNA sequences in Additional file 1). 

Species assignment: The human sequences are given with the gene names only whereas the names of the other species are preceded by abbreviations: Chimpanzee (pt, Pan troglodytes), rhesus monkey (mmul, Macaca mulatta and mfas, Macaca fascicularis), dog (cf, Canis familiaris), cow (bt, Bos taurus), mouse (mm, Mus musculus), rat (rn, Rattus norvegicus) and opossum (mondom, Monodelphis domestica).

Amino acid sequences of human 8q24.3 ZNF genes:

>ZNF251
MAATFQLPGHQEMPLTFQDVAVYFSQAEGRQLGPQQRALYRDVMLENYGNVASLGFPVPKPELISQLEQGKELWVLNLLGAEEPDILKSCQKDSEVGTKKELSILNQKFSEEVKTPEFVSRRLLRDNAQAAEFREAWGREGKLKERVGNSAGQSLNKPNIHKRVLTEATVGRERSLGERTQECSAFDRNLNLDQNVVRLQRNKTGERVFKCDICSKTFKYNSDLSRHQRSHTGEKPYECGRCGRAFTHSSNLVLHHHIHTGNKPFKCDECGKTFGLNSHLRLHRRIHTGEKPFGCGECGKAFSRSSTLIQHRIIHTGEKPYKCNECGRGFSQSPQLTQHQRIHTGEKPHECSHCGKAFSRSSSLIQHERIHTGEKPHKCNQCGKAFSQSSSLFLHHRVHTGEKPYVCNECGRAFGFNSHLTEHVRIHTGEKPYVCNECGKAFRRSSTLVQHRRVHTGEKPYQCVECGKAFSQSSQLTLHQRVHTGEKPYDCGDCGKAFSRRSTLIQHQKVHSGETRKCRKHGPAFVHGSSLTADGQIPTGEKHGRAFNHGANLILRWTVHTGEKSFGCNEYGKAFSPTSRPTEDQIMHAGEKPYKCQECGNAFSGKSTLIQHQVTHTGQKPCHCSVYGKAFSQSSQLTPPQQTRVGEKPALNDGSKRYFIHIKKIFQERHF

>ZNF34
MAALFLSAPPQAEVTFEDVAVYLSREEWGRLGPAQRGLYRDVMLETYGNLVSLGVGPAGPKPGVISQLERGDEPWVLDVQGTSGKEHLRVNSPALGTRTEYKELTSQETFGEEDPQGSEPVEACDHISKSEGSLEKLVEQRGPRAVTLTNGESSRESGGNLRLLSRPVPDQRPHKCDICEQSFEQRSYLNNHKRVHRSKKTNTVRNSGEIFSANLVVKEDQKIPTGKKLHYCSYCGKTFRYSANLVKHQRLHTEEKPYKCDECGKAFSQSCEFINHRRMHSGEIPYRCDECGKTFTRRPNLMKHQRIHTGEKPYKCGECGKHFSAYSSLIYHQRIHTGEKPYKCNDCGKAFSDGSILIRHRRTHTGEKPFECKECGKGFTQSSNLIQHQRIHTGEKPYKCNECEKAFIQKTKLVEHQRSHTGEKPYECNDCGKVFSQSTHLIQHQRIHTGEKPYKCSECGKAFHNSSRLIHHQRLHHGEKPYRCSDCKKAFSQSTYLIQHRRIHTGEKPYKCSECGKAFRHSSNMCQHQRIHLREDFSM


>ZNF517
NRSLRAGRRALLPALSRRPRNSLSVASAPPQRDPGMAMALPMPGPQEAVVFEDVAVYFTRIEWSCLAPDQQALYRDVMLENYGNLASLGFLVAKPALISLLEQGEEPGALILQVAEQSVAKASLCTDSRMEAGIMESPLQRKLSRQAGLPGTVWGCLPWGHPVGGHPAPPHPHGGPEDGSDKPTHPRAREHSASPRVLQEDLGRPVGSSAPRYRCVCGKAFRYNSLLLRHQIIHTGAKPFQCTECGKAFKQSSILLRHQLIHTEEKPFQCGECGKAFRQSTQLAAHHRVHTRERPYACGECGKAFSRSSRLLQHQKFHTGEKPFACTECGKAFCRRFTLNEHGRIHSGERPYRCLRCGQRFIRGSSLLKHHRLHAQEGAQDGGAGQGALLGAAQRPQAGDPPHECPVCGRPFRHNSLLLLHLRLHTGEKPFECAECGKAFGRKSNLTLHQKIHTKEKPFACTECGKAFRRSYTLNEHYRLHSGERPYRCRACGRACSRLSTLIQHQKVHGREPGEDTEGRRAPCWAS


>ZNF7
MEVVTFGDVAVHFSREEWQCLDPGQRALYREVMLENHSSVAGLAGFLVFKPELISRLEQGEEPWVLDLQGAEGTEAPRTSKTDSTIRTENEQACEDMDILKSESYGTVVRISPQDFPQNPGFGDVSDSEVWLDSHLGSPGLKVTGFTFQNNCLNEETVVPKTFTKDAPQGCKELGSSGLDCQPLESQGESAEGMSQRCEECGKGIRATSDIALHWEINTQKISRCQECQKKLSDCLQGKHTNNCHGEKPYECAECGKVFRLCSQLNQHQRIHTGEKPFKCTECGKAFRLSSKLIQHQRIHTGEKPYRCEECGKAFGQSSSLIHHQRIHTGERPYGCRECGKAFSQQSQLVRHQRTHTGERPYPCKECGKAFSQSSTLAQHQRMHTGEKAQILKASDSPSLVAHQRIHAVEKPFKCDECGKAFRWISRLSQHQLIHTGEKPYKCNKCTKAFGCSSRLIRHQRTHTGEKPFKCDECGKGFVQGSHLIQHQRIHTGEKPYVCNDCGKAFSQSSSLIYHQRIHKGEKPYECLQCGKAFSMSTQLTIHQRVHTGERPYKCNECGKAFSQNSTLFQHQIIHAGVKPYECSECGKAFSRSSYLIEHQRIHTRAQWFYEYGNALEGSTFVSRKKVNTIKKLHQCEDCEKIFRWRSHLIIHQRIHTGEKPYKCNDCGKAFNRSSRLTQHQKIHMG


>ZNF250
MAAARLLPVPAGPQPLSFQAKLTFEDVAVLLSQDEWDRLCPAQRGLYRNVMMETYGNVVSLGLPGSKPDIISQLERGEDPWVLDRKGAKKSQGLWSDYSDNLKYDHTTACTQQDSLSCPWECETKGESQNTDLSPKPLISEQTVILGKTPLGRIDQENNETKQSFCLSPNSVDHREVQVLSQSMPLTPHQAVPSGERPYMCVECGKCFGRSSHLLQHQRIHTGEKPYVCSVCGKAFSQSSVLSKHRRIHTGEKPYECNECGKAFRVSSDLAQHHKIHTGEKPHECLECRKAFTQLSHLIQHQRIHTGERPYVCPLCGKAFNHSTVLRSHQRVHTGEKPHRCNECGKTFSVKRTLLQHQRIHTGEKPYTCSECGKAFSDRSVLIQHHNVHTGEKPYECSECGKTFSHRSTLMNHERIHTEEKPYACYECGKAFVQHSHLIQHQRVHTGEKPYVCGECGHAFSARRSLIQHERIHTGEKPFQCTECGKAFSLKATLIVHLRTHTGEKPYECNSCGKAFSQYSVLIQHQRIHTGEKPYECGECGRAFNQHGHLIQHQKVHRKL


>ZNF16
FGAGLPGVSRRWLRRLRATVLPPFQARPVEVLVMPSLRTRREEAEMELSVPGPSPWTPAAQARVRDAPAVTHPGSAACGTPCCSDTELEAICPHYQQPDCDTRTEDKEFLHKEDIHEDLESQAEISENYAGDVSQVPELGDLCDDVSERDWGVPEGRRLPQSLSQEGDFTPAAMGLLRGPLGEKDLDCNGFDSRFSLSPNLMACQEIPTEERPHPYDMGGQSFQHSVDLTGHEGVPTAESPLICNECGKTFQGNPDLIQRQIVHTGEASFMCDDCGKTFSQNSVLKNRHRSHMSEKAYQCSECGKAFRGHSDFSRHQSHHSSERPYMCNECGKAFSQNSSLKKHQKSHMSEKPYECNECGKAFRRSSNLIQHQRIHSGEKPYVCSECGKAFRRSSNLIKHHRTHTGEKPFECGECGKAFSQSAHLRKHQRVHTGEKPYECNDCGKPFSRVSNLIKHHRVHTGEKPYKCSDCGKAFSQSSSLIQHRRIHTGEKPHVCNVCGKAFSYSSVLRKHQIIHTGEKPYRCSVCGKAFSHSSALIQHQGVHTGDKPYACHECGKTFGRSSNLILHQRVHTGEKPYECTECGKTFSQSSTLIQHQRIHNGLKPHECNQCGKAFNRSSNLIHHQKVHTGEKPYTCVECGKGFSQSSHLIQHQIIHTGERPYKCSECGKAFSQRSVLIQHQRIHTGVKPYDCAACGKAFSQRSKLIKHQLIHTRE


>ZNF252
MIPKQDISEELESQRAKSEDHVRNIFKETEEMSKTEGKLENCWRKYAVEGVKNSFSQKSNFREITMRYVKTLSRENGQKFNAVGENCITDSNPAKHLRGSREESLHPSVSSVENLQQHEDLINLQSFQLGERAYQTDVLVKVPRQSSVLSENQRMNNPERWFESTGCGKTYNQNRAFNQHQRFHSGEKTYEHNECGKAFSWPSILSKHQRIHTGKKLYTCEDCGKSFSVHSYFIQHCKIHTREKPYECIKCGKAFSTHSSYVQHLKIHTGEKHHECNQCGKAFSHSSNLIHHQRIHSGEKPYKCKECGKAFNRQSNLIQHQRIHSGEKPYDCKECGKAFSTQLFLIQHQRIHTGEKPYECNECAKSFSLNRTLTVHQRIHTGEKPYRCNECGKSFSQCSQVIQHKRIHTGEKPYICNECGKSFGARLSLIQHQRIHTGEKPYGCSVCGKTFSQKGHLIQHQ

>ZNF252_3'UTR
IHTGEKPYECSECGKAFSQSFNLIHHQRTHNGEKSYECNECDKAFSLLSSLVQHQRIHNGDKPYECHKCGKAFSQGSHLIQHQRSHIGEKPYECNECGKTFGQISTLIKHERTHNGEKPYECSDCGKAFSQSAHLIHHQRIHTGENPYECSECGKAFNVCSSLIQHHRIHTGEKPYECSDCGKAFSQHSQFIQHQRIHTGEKPYMCNECEKSFSACLSLIQHKRIHTGEKPYVCAKCGKSF*QSSHLIQHQRIHSGEQPHTCNRCEKTFS*RITLSSHEKIHTIHIREQVYECSKCGEL*RTVIFHSTLYSSQWR

>ZNF252art
IVVSFEDVAVPLSQEEWDCLIPAQRGLYKDVMMGTYGNLLSLVGLQASKPDVISRLERGDEP*TPHILRTQGSWSWRHKREGCDSRIEKEEMIPKQDISEELESQRAKSEDHVRNIFKETEEMSKTEGKLENCWRKYAVEGVKNSFSQKSNFREITMRYVKTLSRENGQKFNAVGENCITDSNPAKHLRGSREESLHPSVSSVENLQQHEDLINLQSFQLGERAYQTDVLVKVPRQSSVLSENQRMNNPERWFESTGCGKTYNQNRAFNQHQRFHSGEKTYEHNECGKAFSWPSILSKHQRIHTGKKLYTCEDCGKSFSVHSYFIQHCKIHTREKPYECIKCGKAFSTHSSYVQHLKIHTGEKHHECNQCGKAFSHSSNLIHHQRIHSGEKPYKCKECGKAFNRQSNLIQHQRIHSGEKPYDCKECGKAFSTQLFLIQHQRIHTGEKPYECNECAKSFSLNRTLTVHQRIHTGEKPYRCNECGKSFSQCSQVIQHKRIHTGEKPYICNECGKSFGARLSLIQHQRIHTGEKPYGCSVCGKTFSQKGHLIQHQ*IHTGEKPYECSECGKAFSQSFNLIHHQRTHNGEKSYECNECDKAFSLLSSLVQHQRIHNGDKPYECHKCGKAFSQGSHLIQHQRSHIGEKPYECNECGKTFGQISTLIKHERTHNGEKPYECSDCGKAFSQSAHLIHHQRIHTGENPYECSECGKAFNVCSSLIQHHRIHTGEKPYECSDCGKAFSQHSQFIQHQRIHTGEKPYMCNECEKSFSACLSLIQHKRIHTGEKPYVCAKCGKSF*QSSHLIQHQRIHSGEQPHTCNRCEKTFS*RITLSSHEKIHTIHIREQVYECSKCGEL*RTVIFHSTLYSSQWR


>ZNF252krab-A
VSFEDVAVPLSQEEWDCLIPAQRGLYKDVMMGTYGNLLSLVG

>ZNF252krab-B
GLQASKPDVISRLERGDEP*TPHILRTQGSWS

>ZNF252krab(artificially combined)
VSFEDVAVPLSQEEWDCLIPAQRGLYKDVMMGTYGNLLSLVGLQASKPDVISRLERGDEP*TPHILRTQGSWS


>krabA1B1(artificially combined)
GTVMFEEVAMYLTQEEGQHLGPPQRALYQDVMLENHCTLPALGFSVSTLRVISQQEQGKMPWVIALPGPPCT

>krabA2B2(artificially combined)
GSVTFDDVAAYFTRRKWMHLALHQKAAGELWELDEFSGLKPDLIF*LEREEEPWLPDVPGTENTDT


Amino acid sequences of chimpanzee (Pan troglodytes) ZNF genes from the chromosomal region syntenic to human 8q24.3:

>ptZNF251
MAATFQLPGHQEMPLTFQDVAVYFSQAEGQQLGPQQRALYRDVMLENYGNVASLGFPVPKPELISQLEQGKELWVLNLLGAEEPDILKSCQKDSEVGTKKELSILNQKFSEEVKTPEFVSRRLLRDNAQAAEFREAWGREGKLKECVGNSAGQSLNKPNIHKRVLTEATVGRERSLGERTQECSAFDRNLNLDQNVVRLQRNKTGERVFKCDICSKTFKYNSDLSRHQRSHSGEKPYECGRCGRAFTHSSNLVLHHHIHTGNKPFKCDECGKTFGLNSHLRLHRRIHTGEKPFGCGECGKAFSRSSTLIQHRIIHTGEKPYKCNECGRGFSQSPQLTQHQRIHTGEKPHECSHCGKAFSRSSSLIQHERIHTGEKPHKCNQCGKAFSQSSSLFLHHRVHTGEKPYVCNECGRAFGFNSHLTEHVRIHTGEKPYVCNECGKAFRRSSTLVQHRRVHTGEKPYQCVECGKAFSQSSQLTLHQRVHTGEKPYDCGDCGKAFSRRSTLIQHQKVHSGETRKCRKRGPAFVHGSSLTADGQIPTGEKHSRAFNHGANLILRWTVHTGEKSFGCNEYGKAFSPTSRPTEDQIMHAGEKPYKCQECGNAFTGKSTLIQHQVTHTGRKPCHCSVCGKAFSQSSQLTPPQQTRVGEKPALNDGSKRYFIHIKKIFQERHF


>ptZNF34
MAALFLSAPPQAEVTFEDVAVYLSREEWGRLGPAQRGLYRDVMLETYGNLVSLGVGPAGPKPGVISQLERGDEPWVLDVQGTSGKEHLRVNSPALGTRTEYKELTSQETFGEEDPQGSEPVEACDHIRKSEGSLEKLVEQRGPRAVTLTNGESSRESGGNLRLLSRPVPDQRPHKCDICEQSFEQRSYLNNHKRVHRSKKTNTVRDSGEIFSANVVVKDQKIPTGKKLHYCSYCGKTFRYSANLVKHQRLHTEEKPYKCDECGKAFSQSCEFINHRRMHSGEIPYRCDECGKTFTRRPNLMKHQRIHTGEKPYKCGECGKHFSAYSSLIYHQRIHTGEKPYKCNDCGKAFSDGSILIRHRRTHTGEKPFECKECGKGFTQSSNLIQHQRIHTGEKPYKCNECEKAFIQKTKLVEHQRSHTGEKPYECNDCGKVFSQSTHLIQHQRIHTGEKPYKCSECGKAFHNSSRLIHHQRLHHGEKPYRCSDCKKAFSQSTYLIQHRRIHTGEKPYKCSECGKAFRHSSNMCQHQRIHLREDFSM


>ptZNF517
NRSLRAGRRALLPALSRRPRNSLSVASAPPQRYPGMAMALPMPGPQEAVVLEDVAVYFTRIEWSCLAPDQQALYRDVMLENYGNLASLGFLVAKPALISLLEQGEEPGALILQVAEQSVAKASLCTDSRMEAGIMESPLQRKLSRQAGLPGTVWGCLPWGHPAPPHPHGGPEGGSDKPTHPRAREHSASPRVLQEDPGRPVGSSAPRYRCVCGKAFRYNSLLLRHQIIHTGAKPFQCTECGKAFKQSSILLRHQLIHTEEKPFQCGECGKAFRQSTQLAAHHRVHTRERPYACGECGKAFSRSSRLLQHQKFHTGEKPFACTECGKAFCRRFTLNEHGRIHSGERPYRCLRCGQRFIRGSSLLKHHRLHAQEGAQDGGAGQGALLGAAQRPQAGDPPHECPVCGRPFRHNSLLLLHLRLHTGEKPFECAECGKAFGRKSNLTLHQKIHTKEKPFACTECGKAFRRSYTLNEHYRLHSGERPYRCRACGRACSRLSTLIQHQKVHGRERRENTEGRRAPCWAS

>ptZNF7
ARTRVVLGPSDPWSLPAPSRLRHWGPRVPRASSRESAGALRGLERLAWEVVEPSHPPPRRREVSRPEHVDAHPPLSLMEVVTFGDVAVHFSREEWQCLDPGQRALYREVMLENHSSVAGLAGFLVFKPELISRLEQGEEPWVLDLQGAEGTEAPRTSKTDSTIRTENEQACEDMDILESESYGTVVRISPQDFPQNPGFGDVSDSEVWLDSHLGSPGLKVTGFTFQNNCLNEETVVPKTFTKDAAQGCKELGSSGLDCQPLESQRESAEGMSQRCEECGKGIRATSDIALHWEINTQKISRCQECQKKLSDCLQGKHPNNCHGEKPYECAECGKVFRLCSQLNQHQRIHTGEKPFKCTECGKAFRLSSKLIQHQRIHTGEKPYRCEECGKAFGQSSSLIHHQRIHTGERPYGCHECGKAFSQQSQLVRHQRTHTGERPYPCKECGKAFSQSSTLAQHQRMHTGEKAQILKASDSPSLVAHQRIHAVEKPFKCDECGKAFRWISRLSQHQLIHTGEKPYKCNKCTKAFGCSSRLIRHQRTHTGEKPFKCDECGKGFVQGSHLIQHQRIHTGEKPYVCNDCGKAFSQSSSLIYHQRIHKGEKPYECLQCGKAFSMSTQLTIHQRVHTGERPYKCNECGKAFSQNSTLFQHQIIHAGVKPYECSECGKAFSRSSYLIEHQRIHTRAQWFYEYGNALEGSTFVSRKKVNTIKKLHQCEDCEKIFRWRSHLIIHQRIHTGEKPYKCNDCGKAFNRSSRLTQHQKIHTG


>ptZNF250
MAAARLLPVPAGPQPLSFQAKLTFEDVAVLLSQDEWDRLCPAQRGLYRNVMMETYGNVVSLGLPGSKPDIISQLERGEDPWVLDRKGAKKSQGLWSDYSDNLKYDHTTACTQQDSLSCPWECETKGESQNTDLSPKPLISEQTVILGKTPLGRIDQENNETKQSFCLSPNSVDHREVQVLSQSMPLTPHQAVPSGERPYMCVECGKCFGRSSHLLQHQRIHTGEKPYVCSVCGKAFSQSSVLSKHRRIHTGEKPYECNECGKAFRVSSDLAQHHKIHTGEKPHECLECRKAFTQLSHLIQHQRIHTGERPYVCPLCGKAFNHSTVLRSHQRVHTGEKPHRCSECGKTFSVKRTLLQHQRIHTGEKPYTCSECGKAFSDRSVLIQHHNVHTGEKPYECSECGKTFSHRSTLMNHERIHTEEKPYACYECGKAFVQHSHLIQHQRVHTGEKPYVCGECGHAFSARRSLIQHERIHTGEKPFQCTECGKAFSLKATLIVHLRTHTGEKPYECNSCGKAFSQYSVLIQHQRIHTGEKPYECGECGRAFNQHGHLIQHQKVHRKL


>ptZNF16
FGAGLPGVSRRWLRRLRATVLPPFQARPVEVLVMPSLRTRREEAEMELSAPGPSPWTPAAQARVSDAPAVTHPGSAACGTPCCSDTELEAICPHYQQPDCDTRTEDKEFLHKEDIHEDLESQAEISENYAGDVFQVPKLGDLCDDVSERDWGVPEGRRLPQSLSQEGDFTPAAMGLLRGPLGEKDLDCNGFDSRFSLSPNLMACQEIPTEERPHPYDMGGQSFQHSVDLTGHEGVPTAESPLICNECGKTFRGNPDLIQRQIVHTGEASFMCDDCGKTFSQNSVLKNRHRSHMSEKAYQCSECGKAFRGHSDFSRHQSHHSSERPYTCTECGKAFSQNSSLKKHQKSHMSEKPYECNECGKAFRRSSNLIQHQRIHSGEKPYVCSECGKAFRRSSNLIKHHRTHTGEKPFECGECGKAFSQSAHLRKHQRVHTGEKPYECNDCGKPFSRVSNLIKHHRVHTGEKPYKCSDCGKAFSQSSSLIQHRRIHTGEKPHVCNVCGKAFSYSSVLRKHQIIHTGEKPYRCSVCGKAFSHSSALIQHQGVHTGDKPYACHECGKTFGRSSNLILHQRVHTGEKPYECTECGKTFSQSSTLIQHQRIHNGLKPHECNQCGKAFNRSSNLIHHQKVHTGEKPYTCVECGKGFSQSSHLIQHQIIHTGERPYKCSECGKAFSQRSVLIQHQRIHTGVKPYDCAACGKAFSQRSKLIKHQLIHTRE


>ptZNF252
MIPKQDISEELESQRAKSEDHVRNIFKETEEISKTEGKLENCWRKYAVEGVKNSFSQRSNFREITMRYVKTLSRENGQKFNAVGENCITDSNPAKHLRGSREESLHPSVSSVENLQQHEDLINLQSFQLGERAYQTDVLVKVPRQSSVLSENQRMNNPERWFESTGHGKT*NQNRAFNQHQRFHSGEKPYEHNECGKAFSQPSILSKHQRIHTGKKPYT*EDCGKSFSVHSYFIQHCKIHTREKPYECIKCGKAFSTHSSYVQHLKIHTGEKHHECNQCGKAFSHSSNLIHHQRIHSGEKPYKCKECGKAFNRQSHLIQHQRIHSGEKPYDCKKCGKAFSTQLSLIQHQIIHTGEKPYEYNECGKSFSLNRTLTVHQRIHTGEKPYRFNECGKSFSQCSQVIQHKRIHTGEKPYICNECGKSFGARLSLIQHQRIHTGEKPYGCSVCGKTFSQKGHLIQHQ


>ptZNF252krab-A
VSFEDVAVPLSQEEWDCLIPAQRGLYKDVMMETCGNLLSLG
>ptZNF252krab-B
GLQASKPDVISRLERGDEP*TPHILRTRGSWS

>ptZNF252krab (artificially combined)
VSFEDVAVPLSQEEWDCLIPAQRGLYKDVMMETCGNLLSLGGLQASKPDVISRLERGDEP*TPHILRTRGSWS

>ptZNF252art
IVVSFEDVAVPLSQEEWDCLIPAQRGLYKDVMMETCGNLLSLGGLQASKPDVISRLERGDEP*TPHILRTRGSWSWRHKGEGCDSRIEKEEMIPKQDISEELESQRAKSEDHVRNIFKETEEISKTEGKLENCWRKYAVEGVKNSFSQRSNFREITMRYVKTLSRENGQKFNAVGENCITDSNPAKHLRGSREESLHPSVSSVENLQQHEDLINLQSFQLGERAYQTDVLVKVPRQSSVLSENQRMNNPERWFESTGHGKT*NQNRAFNQHQRFHSGEKPYEHNECGKAFSQPSILSKHQRIHTGKKPYT*EDCGKSFSVHSYFIQHCKIHTREKPYECIKCGKAFSTHSSYVQHLKIHTGEKHHECNQCGKAFSHSSNLIHHQRIHSGEKPYKCKECGKAFNRQSHLIQHQRIHSGEKPYDCKKCGKAFSTQLSLIQHQIIHTGEKPYEYNECGKSFSLNRTLTVHQRIHTGEKPYRFNECGKSFSQCSQVIQHKRIHTGEKPYICNECGKSFGARLSLIQHQRIHTGEKPYGCSVCGKTFSQKGHLIQHQ*IHTGEKPYECSECGKAFSQSFNLIHHQRTHNGQKPYECNECDKAFSVLSSLVQHQRIHNGEKPYECHKCGKAFSQVSHLIQHQRSHTGEKP


Amino acid sequences of rhesus monkey (Macaca mulatta; one M. fascicularis) ZNF genes from the chromosomal region syntenic to human 8q24.3:


>mmul_ZNF251_XM_001105603alt
DSEFGTKKELSILNQKFSEEVKTPEFVSRSLLRDNAQAAEFREAWCHEGKLEEHLGNSAGQSLNKPNIHKRVLTEATMGRERSLGERPQEGSAFDRNLNLNQNVVRLQRNKTGERVFKCDICSKTFKYNSDLSRHQRSHTGEKPYQCGRCGRAFTHSSNLVLHHHIHTGNKPFKCDECGKTFGLNSHLRLHRRIHTGEKPFGCGECGKAFSRSSTLIQHRIIHTGEKPYKCNECGRGFSQSPQLTQHQRIHTGEKPHECRHCGKAFSRSSSLIQHERIHTGEKPHKCNQCGKAFSQSSSLFLHHRVHTGEKPYVCNECGRAFGFNSHLTEHVRIHTGEKPYVCNECGKAFRRSSTLVQHRRVHTGEKPYQCVECGKAFSQSSQLTLHQRVHTGEKPYECGDCGKAFSRRSTLIQHQKIHSGETRKCRKRGPAFVHGSSLTPDGQIPTGEKHSRAFSHGANLILRWTVHTGEKSFGCNEYGKAFSPTSQPTEDQKMHAGEKPYKCQECGNAFSGNSALIRHQVTHTGGKPCHCSVCGKAFSQSSQLTPPQQTHVGEKPALNDGSKRYFIQIKKIFQERHF


>mmulZNF34_ENSMMUP00000031827
MAAWFLSAPPQAEVTFEDVAVFLSREEWGRLGPAQRGLYRDVMLETYGNLVSLGVGPAGPKPGVISQLERGDEPWVLDVQGTSGREHLKVNSPALGTRTEYKELTSRETFGKEELGQLRGIFPQGSEPVEACDHIRKAEGSLEKLEQRGPRAVTLTNGESSRGSGGSLRLVSRPVPDQSPHKCDICEQSFEQRSYLNNHKRIHRSKKTKTVRDSGEILSANLVVKEDQKIPTGKKLHYCSYCGKTFRYSANLVKHQRLHSEEKPYKCDECGKAFSQSCEFINHRRMHSGEIPYRCDECGKTFTRRPNLMKHQRIHTGEKPYKCGECGKHFSAYSSLIYHQRIHTGEKPYKCNDCGKAFSDGSILIRHRRTHTGEKPFECKECGKGFTQSSNLIQHQRIHTGEKPYKCNECEKAFIQKTKLVEHQRSHTGEKPYECNDCGKVFSQSTHLIQHQRIHTGEKPYKCSECGKAFHNSSRLIHHQRLHHGEKPYKCSDCKKAFSQSTYLIQHRRIHTGEKPYKCSECGKAFRHSSNMCQHQRIHLREDFSV

>mmul_ZNF517_ENSMMUP00000017611
MTMALPMPGPQEAVVFEDVAVYFTRIEWSCLAPDQQALYRDVMLENYGNLASLGFLVAKPALISLLEQGEEPGALILQVAEQSVAKASLCTDSRMEAGIVESPLQRKLSRQAGLPGTVWGRLPGGHPAPPHPRGSPEDGSDKLTRPRARERSASPRVLQEDPGRPVGSSAPRYRCVCGKAFRYNSLLLRHQVIHTGAKPFQCTECGKAFKQSSILLRHQLIHTEEKPFQCGECGKAFRQSTQLAAHHRVHTRERPYVCGECGKAFSRSSRLLQHQKFHTGEKPFACTECGKAFCRRFTLNEHGRIHSGERPYRCLRCGQRFIRGSSLLKHHRLHAEEGPQDGGAGQGALLRAAQRPQVGDPPHECPVCGRPFRHNSLLLLHLRLHTGEKPFECAECGKAFGRKSNLTLHQKIHTKEKPFACTECGKAFRRSYTLNEHYRLHSGERPYRCRACGRACSRLSTLIQHQKVHGRERQEDTEGRRAPRWAS

>mmulZNF7_XM_001106092
MAVRSSPTHSRASISRTSVPVPLCVCAAATTAVPKTSVPWLLWRSWVSVWHIAQVGPAVCPGPLLLSLGPQPRPSSDLTPPLLRSLGGGAENHRDGRASEPPRAAGSLRGGAGQTRAAKARFRRRRAFAILGWSSGRVSRPEHVDAHPPLSLMEVVTFGDVAVHFSREEWQCLDPGQRALYREVMLENHSSVAGLAGFLVFKPELISRLEQGEEPWVLDLQGAEGTEAPRTSKTDSTIRTENEQSCEDMDILKSESYGTVIRISPQDFPQNPGFGDVSDSEVWLDSHLGSRGLRMTGSTFQNNCLNEETVVPKTFIKDAAQGCKELGSSILDCQPPESQRESAEGTSQRCEGCGKGFRATSDIALHWEINTQKISRCQECQKKLSDCLQGKHPNNCHGEKPYECAECGKVFRLCSQLNQHQRIHTGEKPFKCTECGKAFRLSSKLIQHQRIHTGEKPYRCEECGKAFGQSSSLIHHQRIHTGERPYGCCECGKAFSQQSQLVRHQRTHTGERPYPCKECGKAFSQSSTLAQHQRMHTGEKAQILRASDSPSLVAHQRIHAVEKPFKCDECGKAFRWISRLSQHQLIHTGEKPYKCNKCTKAFGCSSRLIRHQRTHTGEKPFKCDECGKGFVQGSHLIQHQRIHTGEKPYVCNDCGKAFSQSSSLIYHQRIHKGEKPYECLQCGKAFSMSTQLTIHQRVHTGERPYKCNECGKAFSQNSTLFQHQIIHAGVKPYECSECGKAFSRSSYLIEHQRIHTRAQWFYEYGNALEGSTFVSRKKVNTIKKLHQCEDCEKIFRWRSHLIIHQRIHTGEKPYKCNDCGKAFNRSSRLTQHQKIHTG

>mfasZNF7_AB169247
MRQCQKGAGTQGFLGCWCMSFQEVVTFGDVAVHFSREEWQCLDPGQRALYREVMLENHSSVAGLAGFLVFKPELISRLEQGEEPWVLDLQGAEGTEAPRTSKTDSTIRTENEQSCEDMDILKSESYGTVIRISPQDFPQNPGFGDVSDSEVWLDSHLGSRGLRMTGSTFQNNCLNEETVVPKTFIKDAAQGCKELGSSILDCQPPESQRESAEGTSQRCEGCGKGFRATSDIALHWEINTQKINRCQECQKKLSDCLQGKHPNNCHGEKPYECAECGKVFRLCSQLNQHQRIHTGEKPFKCTECGKAFRLSSKLIQHQRIHTGEKPYRCEECGKAFGQSSSLIHHQRIHTGERPYGCCECGKAFSQQSQLVRHQRTHTGERPYPCKECGKAFSQSSTLAQHQRMHTGEKAQILRASDNPSLVAHQRIHAVEKPFKCDECGKAFRWISRLSQHQLIHTGEKPYKCNKCTKAFGCSSRLIRHQRTHTGEKPFKCDECGKGFVQGSHLIQHQRIHTGEKPYVCNDCGKAFSQSSSLIYHQRIHKGEKPYECLQCGKAFSMSTQLTIHQRVHTGERPYKCNECGKAFSQNSTLFQHQIIHAGVKPYECSECGKAFSRSSYLIEHQRIHTRAQWFYEYGNALEGSTFVSRKKVNTIKKLHQCEDCEKIFRWRSHLIIHQRIHTGEKPYKCNDCGKAFNRSSRLTQHQKIHTG


>MMulZNF250
AKLTFEDVAVLLSQDEWDRLCPAQRGLYRNVMMETYGNVVSLGLPGSKPDIISQLERGEDPWVLDRKGAKKSQGLWSDYSDNLKCDHTTACTQDSLSCPWECETKGENQNTDLSPKPLISEETVILGKTPLGRIDQENNETKRSFFLSPNSVDHREVQGLSQSVPLTPHQAVPSGERPYMCVECGKCFGRSSHLLQHQRIHTGEKPYVCNVCGKAFSQSSVLSKHRRIHTGEKPYECNECGKAFRVSSDLAQHHKIHTGEKPHECLECRKAFTQLSHLIQHQRIHTGERPYVCPLCGKAFNHSTVLRSHQRVHTGEKPHRCNECGKTFSVKRTLLQHQRIHTGEKPYTCSECGKAFSDRSVLIQHHNVHTGEKPYECSECGKTFSHRSTLMNHERIHTEEKPYACYECGKAFVQHSHLIQHQRVHTGEKPYVCGECGHAFSARRSLIQHERIHTGEKPFQCTECGKAFSLKATLIVHLRTHTGEKPYECNSCGKAFSQYSVLIQHQRIHTGEKPYECGECGRAFNQHGHLIQHQKVHRKL


>MmulZNF16_ENSMMUP00000021155
MPSLRTRREEAEMELSAPGPSPWTPAAQAPVNDAPAVTHPGSAACGPPCCSDTELEAICPHYQQPDCNTRTEDKEFLHKEDVHEDMESQTEISEHCAGDVSQVPELGDLCDDASERDWGVSEGRRLPQSLSQEGDFTPAALGLLRGALEEKDLACNGFDSCFSLSPNLMSCQEIPTEERPHPYDMDGQSFQHCVDLTGHEEVPTAESPLICNECGKTFQGNPDLIQHQIFHIGEASFMCNGCGKTFSQNSVLKSCHRSHMSEKACQCSECGKALRGCSDFSRHQSHHSSERPYMCNECGKAFSQNSSLKKHQKSHMSEKPYECNECGKAFRRSSNLIQHQRIHSGEKPYVCSECGKAFRRSSNLIKHHRTHTGEKPFECGECRKAFSQSAHLRKHQRVHTGEKPYECNRGKPFSRVSNLINPHRVHTGEKPYKCSDCGKAFSQSSSLIQHRRTHTGEKPHVCTVCGKAFSYSSVLRKHQIIHTGEKPYRCSVCGKAFSHSSALIQHQGVHTGDKPYECHECGKTFGRSSNLILHQRVHTGEKPYECTECGKTFSQSSTLIQHQRIHNGLKPHECSQCGKAFNRSSNLIHHQKVHTGEKPYTCVECGKGFSQSSHLIQHQIIHTGERPYKCSECGKAFSQRSVLIQHQRIHTGVKPYDCAACGKAFSQRSKLIKHQLIHTRE

>MmulZNF252
MVSFEDVAVLLSQEEWDCLIPAQRGLYRDVMLETYGNLVSLGLQASKPDVISRLEQGDKPWTPHILRTQISWSWRHKRECYDSMIEKEELIPKQEISEELESQRAKSEDHVRNIFKETEEMSKTEGKLENCWRKYAVEGVKNSFSRKNNFRAITMRCVKTLSRENGHKFNTVGENCITDSNLDKHVRMSREKSLHPRVSSVENLKQHEDLTNLQSFQLGERACQTDVLVKVPRQSSVLSENQRMNNPDRPFERTGHGKTFNQNTAFNQHQRIHSGEKPYECNECGKAFSRPSILSKHQRIHNGKKPYTCEDCGKSFSAHSYFIQHCKIHTGQKPYECIKCGKAFSTHSSYIRHLKIHTGEKPHESNQCGKAFSHSSNLIHHQRIHSGEKPYKCRECGKAFNRKSHLIQHQRIHSGEKPYDCEECGKAFSTRLSLIQHQRVHTGEKPYECNECGKSFSLNRTLTVHQRIHTGEKPYRCNECGKSFSQRSQVIQHKRIHTGEKPYISNECGKSFGARLSLIQHQIVHTGEKPYGCSVCGKTFSQKGHLIQHQRIHTGEKPHECSECGKAFSQSFNLIHHQRTHNGEKPYECNECDKAFSVLSSLVQHQRIHNGKKPYECHKCGKAFSQGSHLIQHQRSHTGEKPYECNECGKTFGQISTLIKHKRTHNGEKPYECSDCGKAFSQSAHLIRHRRIHTGENPYECSDCGKAFNIRSSLIQHQRIHTGEKPYECSECGKAFSQHSQFIQHQRIHTGEKPYVCTECGKSFRQRSHLTRHQRIHSGERP


Amino acid sequences of dog (Canis familiaris) ZNF genes from the chromosomal region syntenic to human 8q24.3:


>cfZNF34_ENSCAFP00000002444
QAEVTFEDVAVLFSREEWGRLGPSQRGLYRDVMLETYRNLVSLGAGPAGPKPGVITQLERGDEPWDLDAQGAKGTERLRVSVSGHGARTEFKELSSEEMLDREELDQLQGAVPQGRDPEEIPAWSREPEESLDKHMEQRDLRPVPLTSEDSIQESGGGLRFRSSPVSDQRPHKCDICEQSFEQRSYLNNHKRVHRSKKTNIVHDSGEFFSANLVVKEDQKIPLGKKLHYCGYCGKAFRYSANLVKHQRLHSEEKPYKCDECGKAFSQSCEFINHRRMHSGEIPYRCGECGKTFNQRPNLMKHQRIHTGEKPYKCGDCGKHFSAYSSLIYHQRIHTGEKPYKCNDCGKAFSDGSILIRHRRTHTGEKPFECKECGKGFTQSSNLIQHQRIHTGEKPYKCNECEKAFIQKTKLVEHQRSHTGEKPYECNDCGKVFSQSTHLIQHQRIHTGEKPYKCSECGKAFHNSSRLIHHQRSHHGEKPYKCSDCKKAFSQGTYLIQHRRIHTGEKPYKCSKCGKAFRHSSNMCQHQRIHLREDFS

>cfZNF517_XM_539227
MLLDRQHLKGASSPDPCRLWRDSPRQQGLWLQEAVVFEDVAVYFTRIEWSCLAPDQRALYRDVMLENYGHVASLGFLVAKPALISLLEQGEEPGALILQVTEERGSATSRCPDSRMEAGIKQSPLRRVSSKQLGLLGTIWGRLPAGRPKLTELNGSPEDGLDKVPLPLQAGGPGGVLSTSLGVLEDKQRASGRAGTVGQRVYRCACGKAFKYNSLLLRHQVIHTGAKPYQCTECGKAFKQSSILLRHQLIHTEEKPYQCSECGKAFRQSTQLTAHHRVHTREKPYKCGECGKAFGRSSRLRQHQKFHTGEKPYECGECGKAFCRRFTLNEHCRIHSGERPYTCLQCGQRFIRGSSLLKHHRLHARESPRDDSGCPNTLLGAAQKAAAGDKLYQCSVCQRLFKHNSLLLLHQRLHTGEKPFECRECGKAFSRKSNLTLHQKTHTKEKPFACTECGKAFRRSYTLNEHYRLHSGERPYRCRACGRACSRLSALIQHQKVHGPECSREGGEHRRVGKSQRLGCLTLDFGSGHDLGV

>cfZNF7_XM_532359
MRGSPRVLKFLESTGSVPPPGHMDANPPSLMEAVTFGDVAVHFSREEWQCLDPGQRALYKEVMLENHSSVAGLAGFLVFKPELISRLEQGQEPWVLDLKGVEGREGARTFLTDSAGGIASEQAGEDVDVLKSEPCAAMVRSPPPAFPQSSSFSDPSDGAVWSESKPGSLQRNRLSTGTVAPRKTFTREGAQGCGELESSGGLGCQPGESPGGAEGTSRRCDVCGRSFRSASDIALRREIDTRKKPNTCPECKTKVPDCLQGKPRGNCHGEKPYECEECGKVFRLCSQLNQHQRIHTGEKPFKCIECGKAFRLSSKLIQHQRIHTGEKPYRCEECGKAFGQSSSLIHHQRVHTGERPYGCRECGKAFSQQSQLVRHQRTHTGERPYQCQECGKAFSQSSTLAQHQRMHAGDKPQLPRNPDSPSLVAHQRIHATEKPFKCDECGKAFRWVSRLSQHQLTHTGEKPYKCNKCAKAFGCSSRLIRHQRTHTGEKPFKCEECGKGFVQGSHLIQHQRIHTGEKPYECSDCGKAFSQSSSLIYHQRIHKGEKPYECLECGKAFSMSTQLTIHQRVHTGERPYKCTECGKAFSQNSTLFQHQIIHAGVKPYGCSECGKAFSRSSYLIEHQRIHTRAQWYHEYGNTLEASTHVSRKKVSTVKKLHKCNECEKIFRWRSHLIIHQRIHTGEKPYKCNECGKAFNRSSRLTQHQKIHMG


>cfZNF250_ENSCAFP00000002448
MAAARLLPPPAGPQPLSFQAKVTFEDVAVLLSQEEWDRLGPAQRGLYRHVMMETYGNVVSLGLPGSKPNVICQLERGEEPWVLDGQGTKETGGLGSGHSDNYRHDHMPACMGQDSSPCPWECENQGENQERDLGVKPGVSEDGSVVPGEGRPGWRDQGRPRNAPPPRAPAAAGAERPYKCTECGKCFGRSSHLLQHQRTHTGEKPYVCGVCGKAFSQSSVLSKHRRIHTGEKPYECNECGKAFRVSSDLAQHHKIHTGEKPHECLECRKAFTQLSHLIQHQRIHTGERPYVCALCGKAFNHSTVLRSHQRVHTGEKPHECAQCGRAFSVKRTLLQHQRVHTGEKPYTCSECGRAFSDRSVLIQHHNVHTGEKPYECGECGKAFSHRSTLMNHERIHTEEKPYGCYACGKAFVQHSHLTQHQRVHTGEKPYVCGECGHAFSARRSLVQHERIHTGERPFRCAQCGKAFSLKATLIVHLRTHTGERPYECSRCGKAFSQYSVLIQHQRIHTGERPYECGECGRAFNQHGHLIQHQKVHRKL


>cfZNF16_XM_532361alt
MHLPAPAVLGLSVPRAAARSSAAAARKSGEGFPAPPLGRAGSPTAARGDRSDPRAGRGARAFRPTAPVPEAPGLAREPCVRVGNGSGNDTGHAGTPSAGSPPPTVPYARVGGLRSCTRRSKAFRLPQVRDGEDDFKNFKIKRVNIANVHLCGEDPRPAEATPALSCCYRPGTLVRVLLALTIVRTIPGSTGLFVLIQKRGPCATVKQLRPRLYPTTLLLRMLFSDNPQACVSDAPAVTHAGSTLRDPHCCGYTEPGTTPPHHQQPDWDTRTKSKEFLQKKEVSEDLESQEEISENYTSDFSQVPELGELCDDVLERDWGAPDSKRRVQSLYQKGGFTPMAVLLRSPLEKELGCDDFVRSFSLSPNPTASQGIPTEERPHLYDMCGHSFQHSMDLGSHEEHHIAESPLICNDCGKTFRGNPDLIQHQIIHTGQKSFVCNECGKSFSQNSFLKNHQRSHVSEKPYQCSECRKTFSVHSNLIRHQINHSGEKPYVCSECGKAFSQNSSLKKHQKSHMSEKPYECSECGKAFRRSSNLIQHQRIHSGEKPYVCNECGKAFRRSSNLIKHHRTHTGEKPFQCNECGKAFSQSSHLRKHQRVHTGERPYECNECGKPFSRVSNLIKHHRVHTGEKPYKCSDCGKAFSQSSSLIQHRRIHTGEKPHVCNVCGKAFSYSSVLRKHQIIHTGEKPYECSICGKAFSHSSALIQHQGVHTGDKPYECRECGKTFGRSSNLILHQRVHTGEKPYECTECGKTFSQSSTLIQHQRIHNGLKPHECSQCGKAFNRSSNLIHHQKVHTGEKPYTCVECGKGFSQSSHLIQHQIIHTGERPYKCSECGKAFSQRSVLIQHQRIHTGVKPYDCSACGKAFSQRSKALPSAYPSCSHNL


>cfZNF252_NP_001002954
MAVKQLLPAGSQVLVSFEDVAVLLSREEWGRLGPAQRGLYSDVMLETYRNLISLGLQGSKPDVISRLEKGEEPWAPYSAKIEESWIRSHESESFQSLMEKKGLTPKQEISKAMGFRRAKSEYVRNVSKESEFEEMNKTKGKLKNYRKKSAEEELKKSFSQKNSSRPVTLTHVKSPVSGKGQKSSSLEVDYTVDASPVRFHRASTGGSLHQNVPCVNDFQQSQDLINLQCLHLGERACQTDLFMKAPRQSSVLSENQRVNNPEKSFECTECRRLFSPSKALSQHQRSHTGEIPCESGGCGRTSHHCSVLSQHQEVHHGGESHTCAECGKAFKAHSYFIQQHNTHTGERPYECSECAHLSYSQHLQIHSGQKPHECSQCGKAFSHSSNLFHHQRIHSGEKPYECKECGKAFGRHSHLLQHKRIHSGEKPYDCTECGKAFSARLSLIQHQRTHTGEKPYECNECGKSFSLNRTLIVHQRIHTGEKPYRCNECGKSFSQRAQVIQHKRIHTGEKPYVCNECGKSFSARLSLIQHQRIHTGEKPYGCSECGKTFSQKGHLIQHQRIHTGEKPYECNECGKAFSQSFNLIHHQRTHNGEKPYECNECDKAFSVLSSLVQHQRVHNGEKPYECHKCGKAFSQGSHLIQHQRSHTGEKPYECNECGKTFGQISTLIKHERTHNGEKPYECGDCGKAFSQSAHLVRHRRIHTGENPYECSDCGKAFNVRSSLVQHHRIHTGEKPYECEKCGKAFSQHSQFIQHQRIHTGEKPYICNECEKAFSARLSLIQHKRIHTGEKPYKCTECGKSFRQSSHLIRHQRVHSGERPYMCNECGKTFSQRITLTSHEKTHTREQAYKCVKREDLLTAQSASIQHHKVHNGE


Amino acid sequences of bovine (Bos taurus) ZNF genes from the chromosomal region syntenic to human 8q24.3:

>btZNF34_XM_867433
MAALDLCALPQAEVTFEDVAVFLSQEEWGLLGPAQKGLYREVMLETYRNLVSLGAGLAGPKPEVIAQLEQGDELWVLDMHGAEQPSVDGSAHGTRTENQEVTSGEMLFGRELDPLRGSVLRGPEPGEVHERVREPEGRLDRPGEQRGPRLVTLANEECGLESGGNLRSRSRPVPDQRPHKCDICEQSFEQRSYLNNHKRVHRCKKTNIVHDSGEIFAANLVKEDQKIPVGKRLYYCGCCGKAFRYSANLVKHQRLHSEEKPYKCEECGKAFHQSCELISHRRMHSGEIPYRCDECGKTFNQRPNLMKHQRIHTGEKPYKCSECGKHFSAYSSLIYHQRIHTGEKPYKCSDCGKAFSDGSILIRHRRTHTGEKPYECKECGKGFTQSSNLIQHQRIHTGEKPYKCNECEKAFIQKTKLVEHQRSHTGEKPYECNDCGKVFSQSTHLIQHQRIHTGEKPYKCSECGKAFHNSSRLIHHQRSHHGEKPYKCADCKKAFSQGTYLLQHRRIHTGEKPYTCGECGKAFRHSSNMSQHQRIHLREDFSL


>btZNF7_ENSBTAP00000024564
QEAVTFGDVAVHFSREEWQCLDPGQRALYKEVMLENHSSVAGLAGFLVFKPELISRLEQGQDPWVLDLQGAEGREEARTTRTDSTVGTDGEQDMDSFKSESGGVMVKTLPQNFPQSPGFGNTSDPEVCSQRQPTSLFHKNFLNMGTMGPRKAFTEDEFQGHGEWGSSGRLGCQPDQSQWSFGRCDVCGRSLRSPSDAALHQEVNTQQKPNRCQECQKQLSDCFQGRPLSTFPGEKPYECRECGKVFRLCSQLTQHQRIHTGEKPFKCTDCGKAFRLSSKLIQHQRIHTGEKPYRCEECGKAFGQSSSLIHHQRVHTGERPYGCRECGKAFSQQSQLARHQRTHTGERPYPCRECGKAFSQSSTLAQHQRMHAAEKLELPRTPESPSLGARQRMHVPEKPFKCDECGKAFRWVSRLSQHQLTHTGEKPYKCNKCSKAFGCSSRLIRHQRTHTGEKPFKCDECGKGFVQGSHLIQHQRIHTGEKPYECSDCGKAFSQSSSLIYHQRIHKGEKPYECLECGKAFSMSTQLTIHQRVHTGERPYKCSECGKAFSQNSTLFQHQIIHAGVKPYGCSECGKAFSRSSYLIEHQRIHTRAQWYREYGSTLEASTHTSRRRVNTVKKLHKCNECEKIFRWRSHLIIHQRIHTGEKPYKCNECDKAFNRSSRLTQHQKIHMG


>btZNF16_XM_591561alt
MDSEGAGEPSQTWVPSTSQRRGEGWSRVRSGAAESLLLPQPRAATLGRAGGSASRKLRRDFRSTSRPSRLGISSLRSSPSRSRRRLFALWRLRVADRSTYDSDAPAVGAGPGRPVSGARGAVESWAACSLGGPQTGGRVRDALWTLPDRAEQSLPWSRAAGPLGEAAAERPAGWDTRTKGQEFVQKEEVSEDLESQVEISDSSGSDAPQTPELGGLHCGVEERDCGLPEDERQMHRPHQEGAFTLAQMLLRSPSGEREVDCDDIKSSCSWSPSPVECQGPPAEGRPHPLGIRSLPCSLDLPSCTGLHVAESPFICSECGKTFEGDPGLTQHQTGHTGHKSFICNECGRLFSTHTGFLQHQLTHHGEKLHMCSECGKAFCQSSSLKKHQKSHVSEKPYECSECGKTFRRSSNLIQHQRIHSGEKPYVCHACGKAFRRSSNLVKHQRVHTGEKPFECTECGRAFSQSSHMRKHQRVHTGERPYSCSECGKPFSRVSNLIKHHRVHTGEKPYKCSECGKAFSQSSSLIQHRRIHTGEKPHVCAVCGKAFSYSSVLRKHQIIHTGEKPYECGVCGKAFSHSSALVQHQGVHTGDKPYECRECGKTFGRSSNLILHQRVHTGEKPYECTECGKTFSQSSTLIQHQRIHNGLKPHECNQCGKAFNRSSNLIHHQKVHTGEKPYTCVECGKGFSQSSHLIQHQIIHTGERPYQCSECGKSFSQRSVLIQHQRIHTGVKPYDCTACGKAFSQRSHLLQHQRTHTGERPYVCGVCGKAFSQSSVLSKHKRIHTGEKP


Amino acid sequences of mouse (Mus musculus) ZNF genes from the chromosomal region syntenic to human 8q24.3:

>mmZNF251/Zfp251_NM_001007568
MADKSCSPTPGETPLTFQDVAVYFSRAEGQQLSPQERALYRDVMLENYGNVASLGFPGPKPELISQLEQEEELWVLDLLGAEEPEVLRRCQTDSEIEAEKELSILNQKCFEEVKTPEFISPKFPRAYSQASEPQEACAHEGQGDGSHGSSATQGLKSIAKKDAAVCREQFPKNAQTSVFDKHLNPSQSVVTVQRNKAGQRIFKCDICNKTFKYNSDLSRHRRSHTGEKPYECGPCGRAFTHSSNLILHQRIHTGNKPFKCEECGKTFGLNSYLRLHQRIHTGEKPFGCKECGKAFSRSSSLIQHRIIHTGEKPYKCDECGKAFSQSPQLTQHQRIHTGEKPHGCTWCGKAFSRNASLIQHQRIHTGEKPHKCAQCGKAFSQSSSLFLHHRVHTGEKPYVCGECGRAFGFNSHLTEHVRIHTGEKPYVCGECGKAFSRSSTLMQHRRVHTGEKPYQCAECGKAFIQSSQLTLHQRVHTGEKPYECGLCGKAFSRRSALTQHQRVHMGENPQEFECGPDFVYDSSHLSAGERHGRAFSHSAKLVLQWTIRSDEKSRGCHECGKTYSTSSQSMDYQKSQAGEKPYKCQECGGKAGSGVSPLTPHHVTRVGEKPQLKDGSERYLIQIKKIFQERDF

>mmZNF7/Zfp7_NM_145916
MEAVTFGDVAVHFSREEWQCLDSGQRALYKEVMLENHSSVAGLAGFLVFKPELISRLEQGQEPWVLDLQGAEGTEAPRICQTDSAIRTDRKQTCEYTSLLQRQIPGFGDNLDSKVWSENCPRSLGLSVSGSLFQKHRLNSEAVMPKNSTKDAVQERKELQATDVGYRPDDQRDHLSSKLIRRQSVPTGENRYPCEECGKAFRWRSRLNQHKLSHTGEKSYQCNKCTKVFASSSRLIRHQRAHTGEKPFKCDQCGKRFVLASVLTQHQRIHTGERPFKCAECGKGFHLSAKLVQHQRIHTGEKPYRCEECGKTFGQSSSLVHHQRIHTGERPFICQECGKAFCQRSQLSRHRRTHTGERPYSCQECGKAFCQRATLAQHQKMMHTAEKSQMPRASESPSLIACQGNTTEEKPFKCEQCGKAFRWLSRLNQHQVVHSGEKPYQCNKCSKAFGCTSRLIRHQRTHTGEKPFKCDECGKRFVQSSHLIQHQRIHTGEKPYVCDDCGRAFTQSSSLIYHQRIHKGEKPYKCSQCGKAFSMSTQLTSHQRTHTGERPYTCNECGKTFRQNSTLFQHQIIHVRVKPYECNECGKAFSRSSYLIEHQRIHTRAQGGHEFGDTPESPTVLNHKKVHTVKKLYQCDDCDKVFRWRSYLIIHQRIHTGERPYKCNACGKAFHQIAKLTQHQKLHMR


>mmZNF250/Zfp647_NM_172817
MAAAGLLPLPAAPQAKVTFEDVAVLLSQEEWARLGPAQRGLYRHVMMETYGNVVSLGLPGSKPVVISQLERGEDPWVLDGQGTELSQSLGSDHSECKAKEENQNTDLNVPPLISDEASATLTETPLRKVAEERYKTEPKVCPSPKPIGPQNAHGLNPSVPVARPQTAPSVARPYICIECGKCFGRSSHLLQHQRIHTGEKPYVCHVCGKAFSQSSVLSKHRRIHTGEKPYECNECGKAFRVSSDLAQHHKIHTGEKPHECLECGKAFTQLSHLIQHQRIHTGERPYVCPLCGKAFNHSTVLRSHQRVHTGEKPHGCSECGKTFSVERTLLQHQRVHTGEKPYTCSECGKAFSDRSVLIQHHNVHTGEKPYECSECGKTFSHRSTLMNHERIHTQEKPYACYECGKAFVQHSHLIQHQRVHTGEKPYVCGECGHAFSARRSLIQHERIHTGEKPFQCTECGKAFSLKATLIVHLRTHTGEKPYECNSCGKAFSQYSVLIQHQRIHTGEKPYECGECGRAFNQHGHLIQHQKVHKKL


Amino acid sequences of rat (Rattus norvegicus) ZNF genes from the chromosomal region syntenic to human 8q24.3:

>rnZNF251_XM_00107545alt
MAAKSCSRTPGEMPLTFQDVAVYFSRAEGQQLSPQERALYRDVMLENYGNVASLGFPGPKPELISQLEQEEELWVLDLLGAEEPEVLRSCGTDSEIKSEKERSILNQKCSEEVKTPELSQKFPRANSQASESKEACAHESQGDGSQGSSATQGLKGVAKKDSTVCREHLPENTQASSAFDKHLNPSQSGVTIQRNKTGQRIFKCDICNKMFKYNSDLSRHRRSHTGEKPYECGPCGRAFTHSSNLILHQRIHTGNKPFKCDECGKTFGLNSYLRLHQRIHTGEKPFGCNECGKAFSRSSSLIQHRIIHTGEKPYKCNECGKAFSQSPQLTQHQRIHTGEKPHGCSWCGKAFSRNASLIQHQRIHTGEKPHKCTQCGKAFSQSSSLFLHHRVHTGEKPYVCGECGRAFGFNSHLTEHVRIHTGEKPYVCGECGKAFSRSSTLMQHRRVHTGEKPYQCAECGKAFIQSSQLTLHQRVHTGEKPYECGLCGKAFSRRSALTQHRRVHMGESPQEFECGPDFVYDSSSHLSAGERHGGTFSHSAKLVLQWTIRSEEKPRGCRECGKTYSSSSQSVDYQRIQAGEKPSTCQECGGKAGSGVSPLTPHHVTRVGEKPQLKDGSERYLIQIKKIFQERHF


>rnZNF7_XM_235457alt
METVTFGDVAVHFSREEWQCLDSGQRALYREVMLENHSSVAGLAGFLVFKPELISRLEQGQEPWALDLQGAEGTEAPRICQTDSNTRTDCRQTCEYTNLLKRQIPDFGGNLDSKVWSENCPRSLGLRVSGSLFQEHHLKNETMMPKNFTKDAVKECKELQATDLGYQPDDLRDHVKGTSENCEVCGRVVRPVLNLDPYEMNGQERPYRCQECQETSSDCKQEKHTGDCHGKKPYGCEECGKVFRLCSQLNQHQRIHTGEKPFKCVDCGKGFRLSSKLIQHQRIHTGEKPYRCEECGKTFGQSSSLIHHQRIHTGERPYSCQECGKSFSQQSQLVRHQRTHTGERPYPCQECGKAFSQSSTLAQHQRMHTGEKSQMPRASGSPSLLARQRNNTVEKPFKCEECGKAFRWISRLNQHQLIHTGEKPYKCNKCTKAFGCSSRLIRHQRTHTGEKPFKCEECGKGFVQGSHLIQHQRIHTGEKPYVCDDCGKAFSQSSSLIYHQRIHKGEKPYECIQCGKAFSMSTQLTSHQRIHTGERPYKCSECGKAFSQNSTLFQHQIIHAGVKPYECSECGKAFSRSSYLIEHQRIHTRAQWYYEFGNTTESSTFLNHKKVNPVKKLHQCDDCDKIFRWRSHLVIHQRIHTGEKPYRCNACGKAFNRSSRLTQHQKIHMR


>rnZNF250_XM_343279alt
MAAAGLLPLPAAPQAKVTFEDVAVLLSQEEWARLGPAQRGLYRNVMMETYGNVVSLGLPGSKPVVISQLERGEDPWVLDGQETELSQGLGSDHSECKAKEENQNTDSNAQPLISDEASAMLAETPLRKVDEHYKTEPNFCPSPKSVGPQNAHVLNPSVPVARPQMAPSGERPYICIECGKCFGRSSHLLQHQRIHTGEKPYVCHVCGKAFSQSSVLSKHRRIHTGEKPYECNECGKAFRVSSDLAQHHKIHTGEKPHECLECGKAFTQLSHLIQHQRIHTGERPYVCPLCGKAFNHSTVLRSHQRVHTGEKPHGCSECGKTFSVKRTLLQHQRVHTGEKPYTCSECGKAFSDRSVLIQHHNVHTGEKPYECSECGKTFSHRSTLMNHERIHTQEKPYACYECGKAFVQHSHLIQHQRVHTGEKPYVCGECGHAFSARRSLIQHERIHTGEKPFQCTECGKAFSLKATLIVHLRTHTGEKPYECNSCGKAFSQYSVLIQHQRIHTGEKPYECGECGRAFNQHGHLIQHQKVHKKL

>"rnZNF252_krab_B"
SQGSKPSISSRLEQGSEPWAPHLSRVSEESKLVTLFP


Amino acid sequences of opossum (Monodelphis domestica) ZNF genes orthologues to human 8q24.3:


>mondomZNF252_ENSMODP00000005301
MFKDVAVYLTREEWDCLGPAQRSLYRDVMLENYRNLVSLGFSVSKPDVISHLEKGEEPWVLDKQGAREREILKREIADIMSWLRERRGDLKISPLLPSLLSQVALPNLLLSLLSPLHYSRPKGRFKGPEFGEPCKIENQTEKHWEKPVVGVLRKSLSLERNYKPVTVISMKTSTRVKGQECNEFGRRLNLTSKCVKHQRVPRSEKIHPCATCGQTFKKNSDLINHQSIHTREKAHKLNIYGEMPRQSSISIEHQRLHNTEKLYECNECGKAFSQNRTLIQHERIHTREKPYECGECGKTFNRSSILTKHQRIHTGERPYKCNECGKAFSARSYFFQHRKIHTGEKPYECNDCGKSFSTRSSFTQHGKIHTGEKPHECHQCGKAFSHSSNLIHHQRIHTGEKPYKCKECQKAFSRHSHLIQHQRIHTGEKPYECNDCGKAFSARLSLIQHQRIHTGEKPYECNECGKTFSLNRTLIVHQRIHTGEKPYECNECGKSFSQRSQVIQHKRIHTGEKPYICNECGKSFSARLSLIQHQRIHTGEKPYECNECGKTFSQKGHLIQHQRIHTGEKPYECNECGKAFSQSFNLIHHQRTHNGEKPYECNECDKAFSVLSSLVQHQRVHNGEKPYECNKCGKAFSQGSHLIQHQRSHTGEKPYECNECGKTFGQISTLIKHERTHNGEKPYECNECGKAFSQSAHLIRHRRIHTGENPYECNVCGKAFNVRSSLIQHQRIHTGEKPYECNECGKAFSQHSQFIQHQRIHTGEKPYVCDECDKSFSARLSLIQHKRIHTGEKPYECSECGKSFRQSSHLIRHQRIHSGERPFICDECGKTFSQRITLISHEKIHTRE


Other proteins used as outliers and outgroups in alignments:

>ZNF10
MDAKSLTAWSRTLVTFKDVFVDFTREEWKLLDTAQQIVYRNVMLENYKNLVSLGYQLTKPDVILRLEKGEEPWLVEREIHQETHPDSETAFEIKSSVSSRSIFKDKQSCDIKMEGMARNDLWYLSLEEVWKCRDQLDKYQENPERHLRQVAFTQKKVLTQERVSESGKYGGNCLLPAQLVLREYFHKRDSHTKSLKHDLVLNGHQDSCASNSNECGQTFCQNIHLIQFARTHTGDKSYKCPDNDNSLTHGSSLGISKGIHREKPYECKECGKFFSWRSNLTRHQLIHTGEKPYECKECGKSFSRSSHLIGHQKTHTGEEPYECKECGKSFSWFSHLVTHQRTHTGDKLYTCNQCGKSFVHSSRLIRHQRTHTGEKPYECPECGKSFRQSTHLILHQRTHVRVRPYECNECGKSYSQRSHLVVHHRIHTGLKPFECKDCGKCFSRSSHLYSHQRTHTGEKPYECHDCGKSFSQSSALIVHQRIHTGEKPYECCQCGKAFIRKNDLIKHQRIHVGEETYKCNQCGIIFSQNSPFIVHQIAHTGEQFLTCNQCGTALVNTSNLIGYQTNHIRENAY


>ZNF136
CRTWFRFASPRGPEWLAWSLCGAVSCTCLGIRREEAGTPGSQEMDSVAFEDVDVNFTQEEWALLDPSQKNLYRDVMWETMRNLASIGKKWKDQNIKDHYKHRGRNLRSHMLERLYQTKDGSQRGGIFSQFANQNLSKKIPGVKLCESIVYGEVSMGQSSLNRHIKDHSGHEPKEYQEYGEKPDTRNQCWKPFSSHHSFRTHEIIHTGEKLYDCKECGKTFFSLKRIRRHIITHSGYTPYKCKVCGKAFDYPSRFRTHERSHTGEKPYECQECGKAFTCITSVRRHMIKHTGDGPYKCKVCGKPFHSLSSFQVHERIHTGEKPFKCKQCGKAFSCSPTLRIHERTHTGEKPYECKQCGKAFSYLPSLRLHERIHTGEKPFVCKQCGKAFRSASTFQIHERTHTGEKPYECKECGEAFSCIPSMRRHMIKHTGEGPYKCKVCGKPFHSLSPFRIHERTHTGEKPYVCKHCGKAFVSSTSIRIHERTHTGEKPYECKQCGKAFSYLNSFRTHEMIHTGEKPFECKRCGKAFRSSSSFRLHERTHTGQKPYHCKECGKAYSCRASFQRHMLTHAEDGPPYKCMWESL

>ZNF248
MNKSQEQVSFKDVCVDFTQEEWYLLDPAQKILYRDVILENYSNLVSVGYCITKPEVIFKIEQGEEPWILEKGFPSQCHPERKWKVDDVLESSQENEDDHFWELLFHNNKTVSVENGDRGSKTFNLGTDPVSLRNYPYKICDSCEMNLKNISGLIISKKNCSRKKPDEFNVCEKLLLDIRHEKIPIGEKSYKYDQKRNAINYHQDLSQPSFGQSFEYSKNGQGFHDEAAFFTNKRSQIGETVCKYNECGRTFIESLKLNISQRPHLEMEPYGCSICGKSFCMNLRFGHQRALTKDNPYEYNEYGEIFCDNSAFIIHQGAYTRKILREYKVSDKTWEKSALLKHQIVHMGGKSYDYNENGSNFSKKSHLTQLRRAHTGEKTFECGECGKTFWEKSNLTQHQRTHTGEKPYECTECGKAFCQKPHLTNHQRTHTGEKPYECKQCGKTFCVKSNLTEHQRTHTGEKPYECNACGKSFCHRSALTVHQRTHTGEKPFICNECGKSFCVKSNLIVHQRTHTGEKPYKCNECGKTFCEKSALTKHQRTHTGEKPYECNACGKTFSQRSVLTKHQRIHTRVKALSTS

>ZNF25
MNKFQGPVTLKDVIVEFTKEEWKLLTPAQRTLYKDVMLENYSHLVSVGYHVNKPNAVFKLKQGKEPWILEVEFPHRGFPEDLWSIHDLEARYQESQAGNSRNGELTKHQKTHTTEKACECKECGKFFCQKSALIVHQHTHSKGKSYDCDKCGKSFSKNEDLIRHQKIHTRDKTYECKECKKIFYHLSSLSRHLRTHAGEKPYECNQCEKSFYQKPHLTEHQKTHTGEKPFECTECGKFFYVKAYLMVHQKTHTGEKPYECKECGKAFSQKSHLTVHQRMHTGEKPYKCKECGKFFSRNSHLKTHQRSHTGEKPYECKECRKCFYQKSALTVHQRTHTGEKPFECNKCGKTFYYKSDLTKHQRKHTGEKPYECTECGKSFAVNSVLRLHQRTHTGEKPYACKECGKSFSQKSHFIIHQRKHTGEKPYECQECGETFIQKSQLTAHQKTHTKKRNAEK

>ZNF439
MLSLSPILLYTCEMFQDPVAFKDVAVNFTQEEWALLDISQKNLYREVMLETFWNLTSIGKKWKDQNIEYEYQNPRRNFRSVTEEKVNEIKEDSHCGETFTPVPDDRLNFQKKKASPEVKSCDSFVCEVGLGNSSSNMNIRGDTGHKACECQEYGPKPWKSQQPKKAFRYHPSLRTQERDHTGKKPYACKECGKNIIYHSSIQRHMVVHSGDGPYKCKFCGKAFHCLSLYLIHERTHTGEKPYECKQCGKSFSYSATHRIHERTHIGEKPYECQECGKAFHSPRSCHRHERSHMGEKAYQCKECGKAFMCPRYVRRHERTHSRKKLYECKQCGKALSSLTSFQTHIRMHSGERPYECKTCGKGFYSAKSFQRHEKTHSGEKPYKCKQCGKAFTRSGSFRYHERTHTGEKPYECKQCGKAFRSAPNLQLHGRTHTGEKPYQCKECGKAFRSASQLRIHRRIHTGEKPYECKKCGKAFRYVQNFRFHERTQTHKNALWRKTL

>ZNF101
MDSVAFEDVAVNFTQEEWALLSPSQKNLYRDVTLETFRNLASVGIQWKDQDIENLYQNLGIKLRSLVERLCGRKEGNEHRETFSQIPDCHLNKKSQTGVKPCKCSVCGKVFLRHSFLDRHMRAHAGHKRSECGGEWRETPRKQKQHGKASISPSSGARRTVTPTRKRPYECKVCGKAFNSPNLFQIHQRTHTGKRSYKCREIVRAFTVSSFFRKHGKMHTGEKRYECKYCGKPIDYPSLFQIHVRTHTGEKPYKCKQCGKAFISAGYLRTHEIRSHALEKSHQCQECGKKLSCSSSLHRHERTHSGGKLYECQKCAKVFRCPTSLQAHERAHTGERPYECNKCGKTFNYPSCFRRHKKTHSGEKPYECTRCGKAFGWCSSLRRHEMTHTGEKPFDCKQCGKVFTFSNYLRLHERTHLAGRSQCFGRRQGDHLSPGV

>ZNF20
MMFQDSVAFEDVAVSFTQEEWALLDPSQKNLYRDVMQETFKNLTSVGKTWKVQNIEDEYKNPRRNLSLMREKLCESKESHHCGESFNQIADDMLNRKTLPGITPCESSVCGEVGTGHSSLNTHIRADTGHKSSEYQEYGENPYRNKECKKAFSYLDSFQSHDKACTKEKLYDGKECTETFISHSCIQRHRVMHSGDGPYKCKFCGKAFYFLNLCLIHERIHTGVKPYKCKQCGKAFTRSTTLPVHERTHTGVNADECKECGNAFSFPSEIRRHKRSHTGEKPYECKQCGKVFISFSSIQYHKMTHTGEKPYECKQCGKAFRCGSHLQKHGRTHTGEKPYECRQCGKAFRCTSDLQRHEKTHTEDKPYGCKQCGKGFRCASQLQIHERTHSGEKPHECKECGKVFKYFSSLRIHERTHTGEKPRECKQCGKAFRYFSSLHIHERTHTGDKPYECKVCGKAFTCSSSIRYHERTHTGEKPYECKHCGKAFISNYIRYHERTHTGEKPYQCKQCGKAFILPVHVENMKELIPLIDEKSF

>ZNF23
GSRNLLFQKSVTFEDVAVYFTQAEWDGLSPAQRTLYRDVMLENYGNVASLGFPLLKPAVISQLEGGSELGGSSPLAAGTGLQGLQTDIQTDNDLTKEMYEGKENVSFELQRDFSQETDFSEASLLEKQQEVHSAGNIKKEKSNTIDGTVKDETSPVEECFFSQSSNSYQCHTITGEQPSGCTGLGKSISFDTKLVKHEIINSEERPFKCEELVEPFRCDSQLIQHQENNTEEKPYQCSECGKAFSINEKLIWHQRLHSGEKPFKCVECGKSFSYSSHYITHQTIHSGEKPYQCKMCGKAFSVNGSLSRHQRIHTGEKPYQCKECGNGFSCSSAYITHQRVHTGEKPYECNDCGKAFNVNAKLIQHQRIHTGEKPYECNECGKGFRCSSQLRQHQSIHTGEKPYQCKECGKGFNNNTKLIQHQRIHTGEKPYECTECGKAFSVKGKLIQHQRIHTGEKPYECNECGKAFRCNSQFRQHLRIHTGEKPYECNECGKAFSVNGKLMRHQRIHTGEKPFECNECGRCFTSKRNLLDHHRIHTGEKPYQCKECGKAFSINAKLTRHQRIHTGEKPFKCMECEKAFSCSSNYIVHQRIHTGEKPFQCKECGKAFHVNAHLIRHQRSHTGEKPFRCVECGKGFSFSSDYIIHQTVHTWKKPYMCSVCGKAFRFSFQLSQHQSVHSEGKS

>ZNF155
MTTFKEAVTFKDVAVVFTEEELGLLDPAQRKLYRDVMLENFRNLLSVGHQPFHQDTCHFLREEKFWMMGTATQREGNSGGKIQTELESVPEAGAHEEWSCQQIWEQIAKDLTRSQDSIINNSQFFENGDVPSQVEAGLPTIHTGQKPSQGGKCKQSFSDVPIFDLPQQSYSEEKSYTCDECGKSICYISALHVHQRVHVGEKLFMCDVCGKEFSQSSHLQTHQRVHTGEKPFKCEQCGKGFSRRSALNVHHKLHTGEKPYICEACGKAFIHDSQLKEHKRIHTGEKPFKCDICGKTFYFRSRLKSHSMVHTGEKPFRCDTCDKSFHQRSALNRHCMVHTGEKPYRCEQCGKGFIGRLDFYKHQVVHTGEKPYNCKECGKSFRWSSCLLNHQRVHSGEKSFKCEECGKGFYTNSQLSSHQRSHSGEKPYKCEECGKGYVTKFNLDLHQRVHTGERPYNCKECGKNFSRASSILNHKRLHCQKKPFKCEDCGKRLVHRTYRKDQPRDYSGENPSKCEDCGRRYKRRLNLDILLSLFLNDT

>ZNF221
MISPSLELLHSGLYKFPEVEGKMTTFKEAVTFKDVAVVFTEEELGLLDPAQRKLYRDVMLENFRNLLSVGNQPFHQDTFHFLGKEKFWKMKTTSQREGNSGGKIQIEMETVPEAGPHEEWSCQQIWEQIASDLTRSQNSIRNSSQFFKEGDVPCQIEARLSISHVQQKPYRCNECKQSISDVSVFDLHQQSHSGEKSHTCGECGKSFCYSPALHIHQRVHMGEKCYKCDVCGKEFNQSSHLQTHQRVHTGEKPFKRGQCGKGFHSRSALNVHCKLHTGEKPYNCEECGKAFIHDSQLQEHQRIHTGEKPFKCDICGKSFRVRSRLNRHSMVHTGEKPFRCDTCGKNFRQRSALNSHSMVHIEEKPYKCEQCGKGFICRRDFCKHQMVHTGEKPYNCKECGKTFRWSSCLLNHQQVHSGQKSFKCEECGKGFYTNSRRSSHQRSHNGEKPYNCEECGKDYKRRLDLEFHQRVHTGERPYNCKECGKSFGWASCLLKHQRLHSGEKPFKCEECGKRFTQSTQLHSHQTCHTGEKLYKCEQCEKGYNSKFNLDMHQRVHRGERPYNCKECGKSFGWASCLLKHQRLHSGEKPLKSGVWEEIYSEFTASFTSVSLCGRKAI

>ZNF223
MTMSKEAVTFKDVAVVFTEEELGLMDLAQRKLYRDVMLENFRNLLSVGHQPFHRDTFHFLREEKFWMMDIATQREGNSGGKIQPEMKTFPEAGPHEGWSCQQIWEEIASDLTRPQDSTIKSSQFFEQGDAHSQVEEGISIMHTGQKPSNCGKSKQSFSDMSIFDLPQQIRSAEKSHSCDECGKSFCYISALHIHQRVHLGEKLFKCDVCGKEFSQSLHLQTHQRVHTGEKPFKCEQCGRGFRCRSALTVHCKLHMGEKHYNCEACGRAFIHDFQLQKHQRIHTGEKPFKCEICGKSFCLRSSLNRHCMVHTAEKLYKSEKYGRGFIDRLDLHKHQMIHMGQKPYNCKECGKSFKWSSYLLVHQRVHTGEKPYKCEECGKGYISKSGLDLHHRAHTGERPYNCDDCGKSFRQASSILNHKRLHCRKKPFKCEDCGKKLVYRSYRKDQQKNHSGENPSKCEDCGKRYKRRLNLDIILSLFLNDT

>ZNF224
MTTFKEAMTFKDVAVVFTEEELGLLDLAQRKLYRDVMLENFRNLLSVGHQAFHRDTFHFLREEKIWMMKTAIQREGNSGDKIQTEMETVSEAGTHQEWSFQQIWEKIASDLTRSQDLVINSSQFSKEGDFPCQTEAGLSVIHTRQKSSQGNGYKPSFSDVSHFDFHQQLHSGEKSHTCDECGKNFCYISALRIHQRVHMGEKCYKCDVCGKEFSQSSHLQTHQRVHTGEKPFKCVECGKGFSRRSALNVHHKLHTGEKPYNCEECGKAFIHDSQLQEHQRIHTGEKPFKCDICGKSFCGRSRLNRHSMVHTAEKPFRCDTCDKSFRQRSALNSHRMIHTGEKPYKCEECGKGFICRRDLYTHHMVHTGEKPYNCKECGKSFRWASCLLKHQRVHSGEKPFKCEECGKGFYTNSQCYSHQRSHSGEKPYKCVECGKGYKRRLDLDFHQRVHTGEKLYNCKECGKSFSRAPCLLKHERLHSGEKPFQCEECGKRFTQNSHLHSHQRVHTGEKPYKCEKCGKGYNSKFNLDMHQKVHTGERPYNCKECGKSFGWASCLLKHQRLRSGEKPFKCEECGKRFTQNSQLHSHQRVHTGEKPYKCDECGKGFSWSSTRLTHQRRHSRETPLKCEQHGKNIVQNSFSKVQEKVHSVEKPYKCEDCGKGYNRRLNLDMHQRVHMGEKTWKCRECDMCFSQASSLRLHQNVHVGEKP


>ZNF225
MTTLKEAVTFKDVAVVFTEEELRLLDLAQRKLYREVMLENFRNLLSVGHQSLHRDTFHFLKEEKFWMMETATQREGNLGGKIQMEMETVSESGTHEGLFSHQTWEQISSDLTRFQDSMVNSFQFSKQDDMPCQVDAGLSIIHVKTETSEGRTCKKSFSDVSVLDLHQQLQSREKSHTCDECGKSFCYSSALRIHQRVHMGEKLYNCDVCGKEFNQSSHQQIHQRIHTGEKPFKCEQCGKGFSRRSGLYVHRKLHTGVKPHICEKCGKAFIHDSQLQEHQRIHTGEKPFKCDICCKSFRSRANLNRHSMVHMREKPFRCDTCGKSFGLKSALNSHRMVHTGEKRYKCEECGKRFIYRQDLYKHQIDHTGEKPYNCKECGKSFRWASGLSRHVRVHSGETTFKCEECGKGFYTNSQRYSHQRAHSGEKPYRCEECGKGYKRRLDLDFHQRVHRGEKPYNCKECGKSFGWASCLLNHQRIHSGEKPFKCEECGKRFTQNSQLYTHRRVHSGEKPFKCEECGKRFTQNSQLYSHRRVHTGVKPYKCEECGKGFNSKFNLDMHQRVHTGERPYNCKECGKSFSRASSILNHKRLHGDEKPFKCEECGKRFTENSQLHSHQRVHTGEKPYKCEKCGKSFRWASTHLTHQRLHSREKLLQCEDCGKSIVHSSCLKDQQRDQSGEKTSKCEDCGKRYKRRLNLDTLLSLFLNDT


>ZNF228
MTVSKEMVTFKDVAVVFTEEELGLLDSVQRKLYRDVMLENFRNLLLVAHQPFKPDLISQLEREEKLLMVETETPRDGCSGRKNQQKMESIQEVTVSYFSPKELSSRQTWQQSTGGLIRCQDFLKVFQGKNSQLQEQGNSLGQAWAGIPVQISEDKNYIFTHIGNGSNYIKSQGYPSWRAHHSWRKMYLKESHNYQCRCQQISMKNHFCKCDSVSWLSHHNDELEVHRKENYSCHDCGEDIMKVSLLNQESIQTEEKPYPCSGYRKAFSNDSSSEVHQQFHLEGKPYTYSSCGKGCNYSSLLHIHQNIEREDDIENSHLKSYQRVHTEEKPCKCGEYGENFNHCSPLNTYELIHTGEMSYRHNIYEKAFSHSLDLNSIFRVHTRDEPHEYEESENVFNQSSCLQVHQKIHTEEKLYTDIEYGKSFICSSNLDIQHRVHMEENSYNSQECGNGFSLASHFQDLQIVHTKEQPYKRYVCSNSFSHNLHLQGHPKIHIGEKPRKEHGNGFNWSSKLKDHQRVHTGQKPYKCNICGKGFNHRSVLNVHQRVHTGEKPYKCEECDKGFSRSSYLQAHQRVHTGEKPYKCEECGKGFSRNSYLQGHQRVHTGEKPYKCEECGKGFSRSSHLQGHQRVHTGEKPFKCEECGKGFSWSFNLQIHQRVHTGEKPYKCEECGKGFSKASTLLAHQRVHTGEKPYQCDECGKSFSQRSYLQSHQSVHSGERPYICEVCGKGFSQRAYLQGHQRVHTRVKPYKCEMCGKGFSQSSRLEAHRRVHTGGKPYKCEVCTKGFSESSRLQAHQRVHVEGRPYKCEQCGKAFSGYSSLQAHHRVHTGEKPYKCEVCGKGFSQRSNLQAHQRVHTGEKPYKCDACGKGFRWSSGLLIHQRVHSSDKFYKSEDYGKDYPSSENLHRNEDS
VLF

>ZNF226
MNMFKEAVTFKDVAVAFTEEELGLLGPAQRKLYRDVMVENFRNLLSVGHPPFKQDVSPIERNEQLWIMTTATRRQGNLGEKNQSKLITVQDRESEEELSCWQIWQQIANDLTRCQDSMINNSQCHKQGDFPYQVGTELSIQISEDENYIVNKADGPNNTGNPEFPILRTQDSWRKTFLTESQRLNRDQQISIKNELCQCKKGVDPIGWISHHDGHRVHKSEKSYRPNDYEKDNMKILTFDHNSMIHTGQKSYQCNECKKPFSDLSSFDLHQQLQSGEKSLTCVERGKGFCYSPVLPVHQKVHVGEKLKCDECGKEFSQGAHLQTHQKVHVIEKPYKCKQCGKGFSRRSALNVHCKVHTAEKPYNCEECGRAFSQASHLQDHQRLHTGEKPFKCDACGKSFSRNSHLQSHQRVHTGEKPYKCEECGKGFICSSNLYIHQRVHTGEKPYKCEECGKGFSRPSSLQAHQGVHTGEKSYICTVCGKGFTLSSNLQAHQRVHTGEKPYKCNECGKSFRRNSHYQVHLVVHTGEKPYKCEICGKGFSQSSYLQIHQKAHSIEKPFKCEECGQGFNQSSRLQIHQLIHTGEKPYKCEECGKGFSRRADLKIHCRIHTGEKPYNCEECGKVFRQASNLLAHQRVHSGEKPFKCEECGKSFGRSAHLQAHQKVHTGDKPYKCDECGKGFKWSLNLDMHQRVHTGEKPYKCGECGKYFSQASSLQLHQSVHTGEKPYKCDVCGKVFSRSSQLQSHQRVHTGEKPYKCEICGKSFSWRSNLTVHHRIHVGDKSYKSNRGGKNIRESTQEKKSIK

>Xfin_X06021_alt_EU277665 [Xenopus laevis]
]
MVPTFDDVAVYFSRSEWKSLSASQREMYKSVMTENYQCVLSLGYPIRKPEIVSMMEVGEELWSKNDSARPGQKEVEGETPKESDWAAENCKRAQMHKEVLDLDTLAAVKSEPVEEGSNSAKKSHICSHYGKLFSCYAAVVRHQRMHQLQKSHHCPHCKKSFVQRSDFIKHQRTHTGERPYQCVECQKKFTERSALVNHQRTHTGERPYTCLDCQKTFNQRSALTKHRRTHTGERPYRCSVCSKSFIQNSDLVKHLRTHTGEKPYECPLCVKRFAESSALMKHKRTHSTHRPFRCSECSRSFTHNSDLTAHMRKHTEFRNVLNLDSVVGTDPLSSQNVASSPYSCSKCRKTFKRWKSFLNHQQTHSREKPYLCSHCNKGFIQNSDLVKHFRTHTGERPYQCAECHKGFIQKSDLVKHLRTHTGEKPFKCSHCDKKFTERSALAKHQRTHTGEKPYKCSDCGKEFTQRSNLILHQRIHTGERPYKCTLCDRTFIQNSDLVKHQKVHANLPLSDPHTANSPHKCSKCDLTFSHWSTFMKHSKLHSGEKKFQCAECKKGFTQKSDLVKHIRVHTGEKPFKCLLCKKSFSQNSDLHKHWRIHTGEKPFPCYTCDKSFTERSALIKHHRTHTGERPHKCSVCQKGFIQKSALTKHSRTHTGEKPYPCTQCGKSFIQNSDLVKHQRIHTGEKPYHCTECNKRFTEGSSLVKHRRTHSGEKPYRCPQCEKTFIQSSDLVKHLVVHNGENPPAATAFHEILIRRENLTRSEPDPYPCTECGKVFHQRPALLKHLRTHKTEKRYPCNECDKSFFQTSDLVKHLRTHTGERPYHCPECNKGFIQNSDLVKHQRTHTGERPYTCSQCDKGFIQRSALTKHMRTHTGEKPYKCEQCQKCFIQNSDLVKHQRIHTGEKPYHCPDCDKRFTEGSSLIKHQRIHSRIKPYPCGVCGKSFSQSSNLLKHLKCHSEQNPPVALSSELGFVAETQTHPDPVDHIVYGDTASYISPEAAGERSFKCNDCGKCFAHRSVLIKHVRIHTGERPYKCSQCTRSFIQKSDLVKHYRTHTGERPYKCGLCERSFVEKSALSRHQRVHKNESPVLNSAMEQQQVTYWGESKDDPNSLVPQLHVIKEEESPHIVNAYSPLSILQSYFPPILEPKGTPRYSCSECGKCFTHRSVFLKHWRMHTGEQPYTCKECGKSFSQSSALVKHVRIHTGEKPYPCSTCGKSFIQKSDLAKHQRIHTGEKPYTCTVCGKKFIDRSSVVKHSRTHTGERPYKCNECTKGFVQKSDLVKHMRTHTGEKPYGCNCCDRSFSTHSASVRHQRMCNTGRPYQDEEYENSLFYSADITWKGDYAQLLQIPCGLEEPMKAIGWISEVAL
