# Supplementary material for: The ancient mammalian KRAB zinc finger gene cluster on human chromosome 8q24.3 illustrates principles of C2H2 zinc finger evolution associated with unique expression profiles in human tissues
Source: BMC Genomics. 2010 Mar 26;11:206. doi: 10.1186/1471-2164-11-206 (PMC2865497; doi:10.1186/1471-2164-11-206)
Supplement: Additional file 3 — Orthologs of human 8q24.3 ZNF genes at syntenic genomic regions in mouse and rat. Table summarizing accession numbers and genomic localization of the mouse and rat orthologs. [file 1471-2164-11-206-S3.PDF]

Additional file 3: Orthologs of human 8q24.3 ZNF genes at syntenic genomic regions in mouse and rat

	**Homo sapiens** (build 36.1, hg18)	**Mus musculus** (build 37, mm9)	**Rattus norvegicus** (build 3.4, rn4)
**Name**	**ZNF251**	**mmZNF251/Zfp251**	**rnZNF251**
Accession		NM_001007568; BC059071	XM_001075451alt
Entrez Gene ID		71591	366954
Localization	8(-)	15(-)76.682.561-76.701.865	7(-)114.897.080-114.926.635
Size		19305 bp	29556 bp
**Name**	**ZNF34**	**none**	**none**
**Name**	**ZNF517**	**none**	**none**
**Name**	**ZNF7**	**mmZNF7/Zfp7**	**rnZNF7**
Accession		NM_145916, BC011501	XM_235457alt; NM_001142757
Entrez Gene ID		223669	315101
Localization	8(+)	15(+)76.709.689-76.722.825	7(+)114.935.565-114.943.596
Size		13137 bp	8032 bp
**Name**	**ZNF250(ZNF647)**	**mmZNF250/Zfp647**	**rnZNF250**
Accession		NM_172817	XM_343279alt
Entrez Gene ID		239546	362948
Localization	8(-)	15(-)76.740.801-76.755.878	7(-)114.960.293-114.974.748
Size		15078 bp	14456 bp
**Name**	**ZNF16**	**mmZNF16 pseudogene**	**rnZNF16 pseudogene**
Accession		genomic AC157554	genomic NC_005106
Entrez Gene ID		-	-
Localization	8(-)	15(-)76.764.004-76.765.966	7(-)114.980.706-114.982.689
Size		1963 bp	1984 bp
**Name/accession**	**ZNF252**	**none**	**putative KRAB-B** 7(+)115.003.928-115.004.038 (111bp)

Assignment of mouse (mm= Mus musculus) and rat (rn = Rattus norvegicus) orthologs of human 8q24.3 cluster ZNF genes based on reciprocal BLAST searches and comparison of genomic loci between syntenic regions.

Accession: GenBank accession numbers for transcripts or genomic regions. The additon “alt” for rat accessions means that the predicted mRNA sequence was adjusted to result in an alternative model that better fits the syntenic models in mouse.
