# Supplementary material for: The ancient mammalian KRAB zinc finger gene cluster on human chromosome 8q24.3 illustrates principles of C2H2 zinc finger evolution associated with unique expression profiles in human tissues
Source: BMC Genomics. 2010 Mar 26;11:206. doi: 10.1186/1471-2164-11-206 (PMC2865497; doi:10.1186/1471-2164-11-206)
Supplement: Additional file 4 — Phylogeny of the human 8q24.3 ZNF genes and their mammalian orthologs. Extended phylogenetic trees of the human 8q24.3 ZNF genes and their mammalian orthologs that were constructed using cDNA, whole protein, zinc finger region and KRAB domain sequences. [file 1471-2164-11-206-S4.PDF]

Additional file 4: Phylogeny of the human 8q24.3 ZNF genes and their mammalian orthologs

Phylogenetic trees constructed with MEGA4 (Kumar et al., 2008. Brief Bioinform. 9:299-306) after alignments of the 8q24.3 ZNF gene sequences and all their orthologs with Clustal W 1.83 (Thompson et al. 1994. Nucleic Acids Res. 22:4673-80) with the neighbor-joining algorithm after 1000 bootstrap cycles (numbers on branch points indicate %). As operational taxonomic units the gene names are given (the human orthologs without affix). Species designation: Chimpanzee (*Pan troglodytes*, pt), rhesus monkey (*Macaca mulatta*, mmul; *Macaca fascicularis*, mfas), dog (*Canis familiaris*, cf), cow (*Bos taurus*, bt), mouse (*Mus musculus*, mm), rat (*Rattus norvegicus*, rn) and opossum (*Monodelphis domestica*, mondom). Outlier for rooting: Xfin (*Xenopus laevis*). Full-length cDNA and protein sequences are given in Additional files 1, 2. The suffix "art" indicates artificially combined sequences ignoring stop codons.

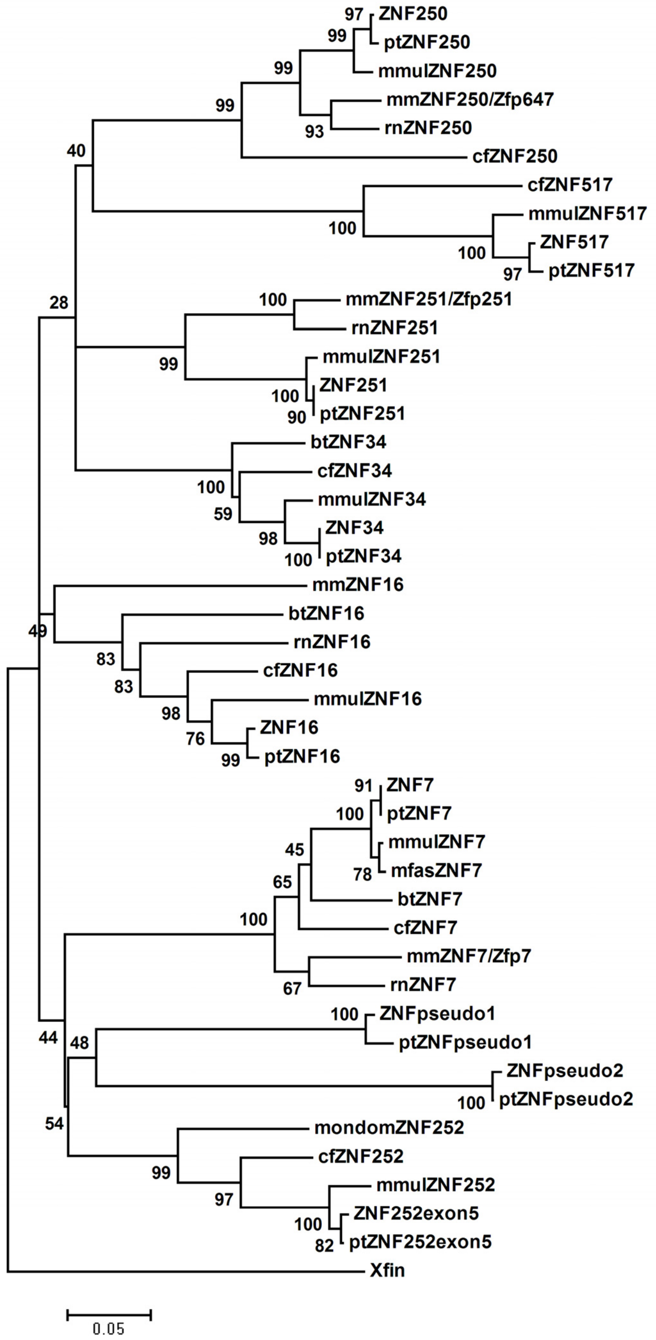

A: Input sequences: Full-length nucleotide sequences

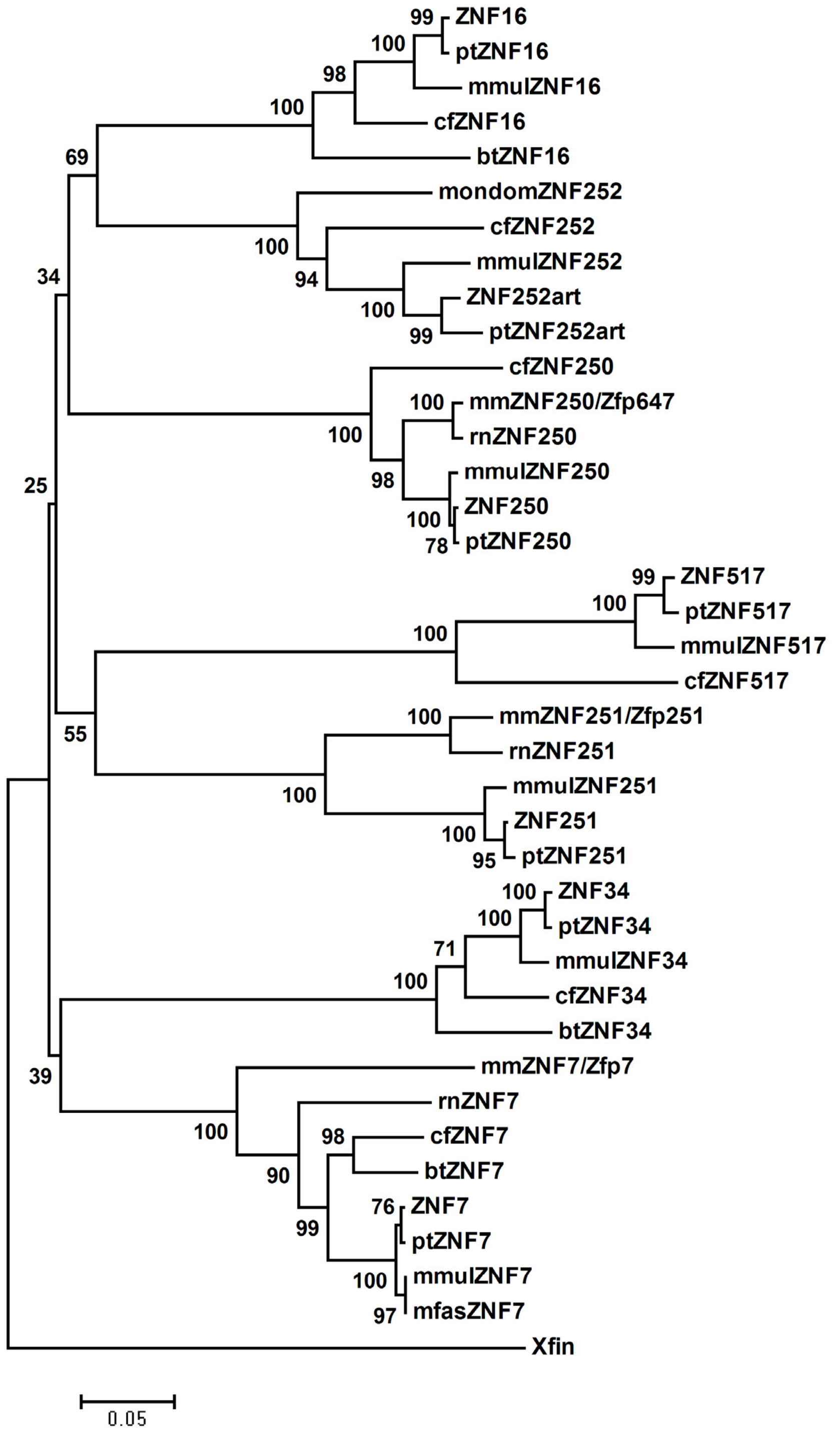

B: Input sequences: Full-length amino acid sequences

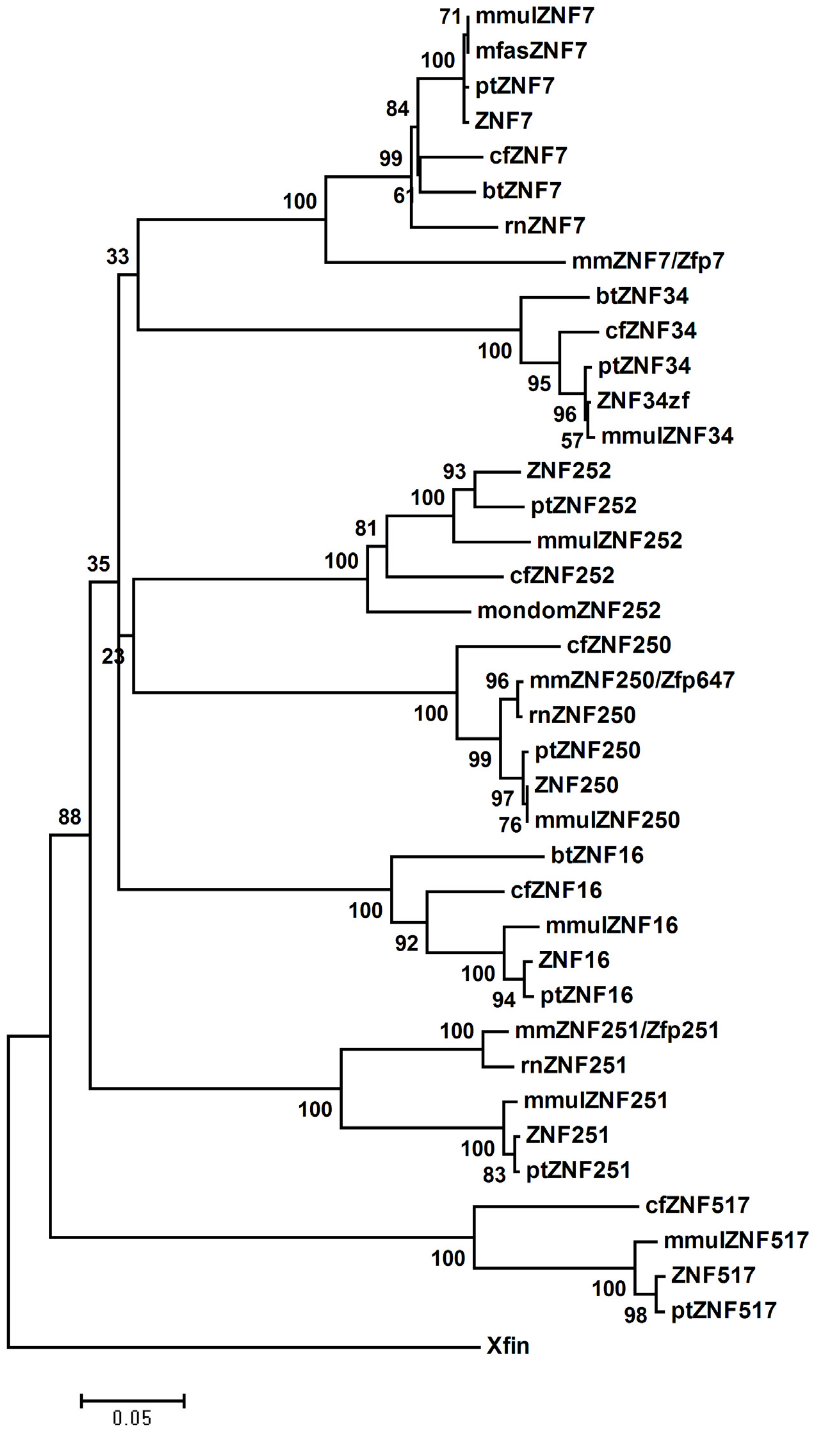

C: Input sequences: Amino acid sequences of the zinc finger regions (all aligned zinc fingers of each gene product

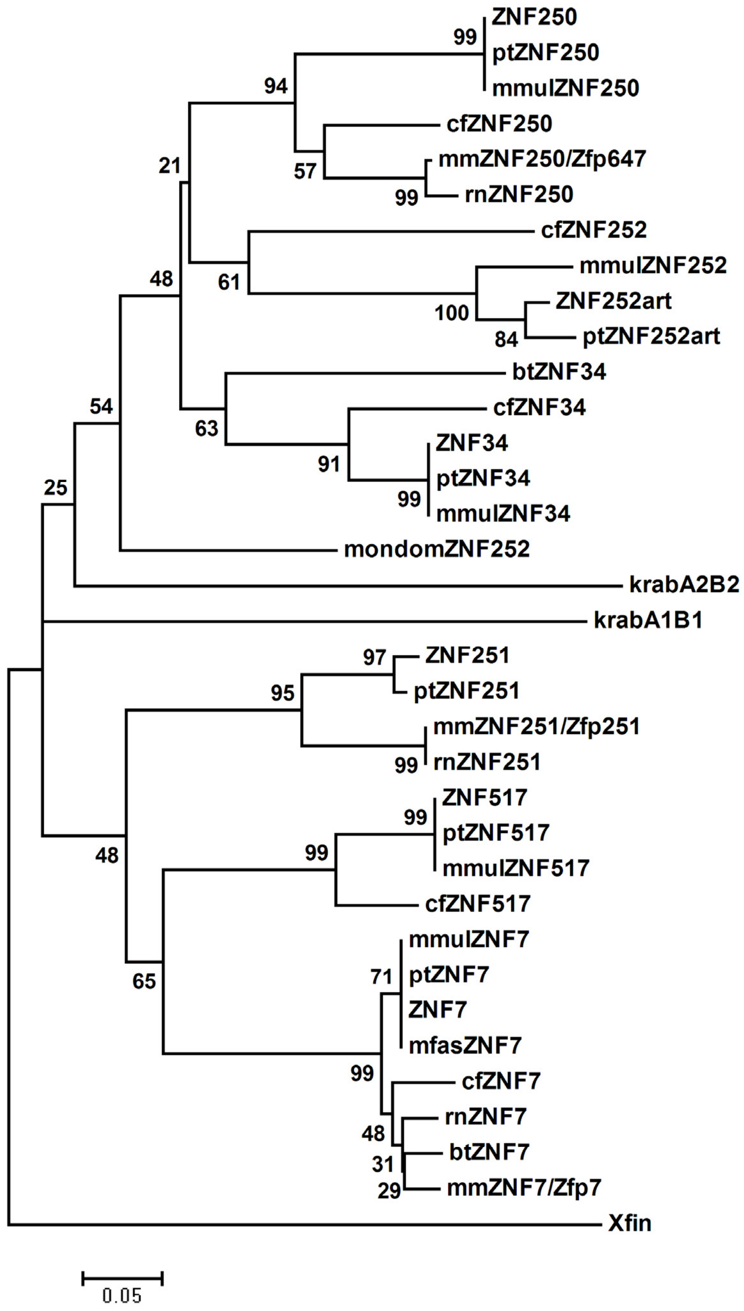

D: Input sequences: Amino acid sequences of the KRAB domains
