# Supplementary material for: The ancient mammalian KRAB zinc finger gene cluster on human chromosome 8q24.3 illustrates principles of C2H2 zinc finger evolution associated with unique expression profiles in human tissues
Source: BMC Genomics. 2010 Mar 26;11:206. doi: 10.1186/1471-2164-11-206 (PMC2865497; doi:10.1186/1471-2164-11-206)
Supplement: Additional file 5 — Calculations of synonymous and non-synonymous substitution rates. Table containing the values from the domain-wise calculations of synonymous and nonsynonymous substitution rates within an ortholog group of each 8q24.3 ZNF gene (done with the YN00 module of the PAML software package version 3.14 [106,54]). [file 1471-2164-11-206-S5.PDF]

Domain	Gene Fragment	Seq1	Seq2	S	N	t	Kappa	Omega	dN	dN_SE	dS	dS_SE	Z Score	P Value
KRAB_A	ZNF251krab_A	rnZNF251krab_A	ptZNF251krab_A	22.7	106.3	0.6422	6.3585	0.0318	0.0337	0.0182	1.0597	0.6776	-1.51362	0.0651
KRAB_A	ZNF251krab_A	hsZNF251krab_A	ptZNF251krab_A	17.5	111.5	0.1026	6.3585	0.0462	0.009	0.009	0.1952	0.1171	-1.58542	0.0564
KRAB_A	ZNF251krab_A	hsZNF251krab_A	rnZNF251krab_A	21.2	107.8	0.4058	6.3585	0.0713	0.0431	0.0206	0.6049	0.2601	-2.1532	0.0157
KRAB_A	ZNF251krab_A	mmZNF251krab_A	ptZNF251krab_A	27	102	1.0363	6.3585	0.0309	0.0457	0.0218	1.4788	1.5566	-0.92057	0.1786
KRAB_A	ZNF251krab_A	mmZNF251krab_A	rnZNF251krab_A	29.3	99.7	0.0984	6.3585	0.0916	0.0101	0.0101	0.1102	0.0649	-1.52403	0.0638
KRAB_A	ZNF251krab_A	mmZNF251krab_A	hsZNF251krab_A	25.4	103.6	0.5977	6.3585	0.0708	0.0555	0.0241	0.7842	0.3616	-2.01075	0.0222
KRAB_A	ZNF34krab_A	btZNF34krab_A	cfZNF34krab_A	31	95	0.5695	5.2413	0.1457	0.0777	0.0299	0.5337	0.2147	-2.10359	0.0177
KRAB_A	ZNF34krab_A	mmulZNF34krab_A	cfZNF34krab_A	29	97	0.9592	5.2413	0.0341	0.0426	0.0215	1.2472	1.3332	-0.90342	0.1832
KRAB_A	ZNF34krab_A	mmulZNF34krab_A	btZNF34krab_A	31.4	94.6	0.5378	5.2413	0.0994	0.055	0.0249	0.5535	0.2385	-2.07885	0.0188
KRAB_A	ZNF34krab_A	hsZNF34krab_A	cfZNF34krab_A	27.1	98.9	0.6475	5.2413	0.0688	0.0552	0.0244	0.802	0.5078	-1.46896	0.0709
KRAB_A	ZNF34krab_A	hsZNF34krab_A	btZNF34krab_A	29.8	96.2	0.5134	5.2413	0.1272	0.0653	0.027	0.5131	0.2236	-1.98824	0.0234
KRAB_A	ZNF34krab_A	hsZNF34krab_A	mmulZNF34krab_A	26.7	99.3	0.0742	5.2413	0.1289	0.0102	0.0102	0.0788	0.0565	-1.19484	0.1161
KRAB_A	ZNF34krab_A	ptZNF34krab_A	cfZNF34krab_A	27.1	98.9	0.6475	5.2413	0.0688	0.0552	0.0244	0.802	0.5078	-1.46896	0.0709
KRAB_A	ZNF34krab_A	ptZNF34krab_A	btZNF34krab_A	29.8	96.2	0.5134	5.2413	0.1272	0.0653	0.027	0.5131	0.2236	-1.98824	0.0234
KRAB_A	ZNF34krab_A	ptZNF34krab_A	mmulZNF34krab_A	26.7	99.3	0.0742	5.2413	0.1289	0.0102	0.0102	0.0788	0.0565	-1.19484	0.1161
KRAB_A	ZNF34krab_A	ptZNF34krab_A	hsZNF34krab_A	24.3	101.7	0	5.2413	99	0	0	0	0	#DIV/0!	#DIV/0!
KRAB_A	ZNF517krab_A	hsZNF517krab_A	mmulZNF517krab_A	26.9	99.1	0.1073	3.2888	0	0	0	0.1676	0.0876	-1.91324	0.0279
KRAB_A	ZNF517krab_A	cfZNF517krab_A	mmulZNF517krab_A	24.3	101.7	0.5194	3.2888	0.0391	0.0301	0.0175	0.7705	0.4641	-1.59421	0.0554
KRAB_A	ZNF517krab_A	cfZNF517krab_A	hsZNF517krab_A	26.5	99.5	0.3967	3.2888	0.0599	0.0308	0.0179	0.5136	0.2352	-2.0468	0.0203
KRAB_A	ZNF517krab_A	ptZNF517krab_A	mmulZNF517krab_A	27	99	0.0743	3.2888	0.1299	0.0102	0.0102	0.0784	0.0565	-1.18788	0.1174
KRAB_A	ZNF517krab_A	ptZNF517krab_A	hsZNF517krab_A	29.1	96.9	0.0741	3.2888	0.1436	0.0104	0.0104	0.0724	0.0521	-1.167	0.1216
KRAB_A	ZNF517krab_A	ptZNF517krab_A	cfZNF517krab_A	26.7	99.3	0.4375	3.2888	0.0774	0.0414	0.0209	0.5347	0.2614	-1.88114	0.0300
KRAB_A	ZNF7krab_A	cfZNF7krab_A	btZNF7krab_A	31.1	94.9	0.377	4.8889	0	0	0	0.5095	0.1918	-2.65641	0.0039
KRAB_A	ZNF7krab_A	ptZNF7krab_A	btZNF7krab_A	33.3	92.7	0.326	4.8889	0.0625	0.0219	0.0155	0.3504	0.1341	-2.43346	0.0075
KRAB_A	ZNF7krab_A	ptZNF7krab_A	cfZNF7krab_A	32	94	0.6126	4.8889	0.0291	0.0216	0.0153	0.7412	0.2995	-2.39954	0.0082
KRAB_A	ZNF7krab_A	mmZNF7krab_A	btZNF7krab_A	30.7	95.3	0.6117	4.8889	0.0131	0.0106	0.0106	0.8043	0.3441	-2.3055	0.0106
KRAB_A	ZNF7krab_A	mmZNF7krab_A	cfZNF7krab_A	29.2	96.8	0.5878	4.8889	0.0128	0.0104	0.0104	0.8115	0.3096	-2.58607	0.0049
KRAB_A	ZNF7krab_A	mmZNF7krab_A	ptZNF7krab_A	31.8	94.2	0.3661	4.8889	0.0842	0.0326	0.0189	0.3867	0.1576	-2.23084	0.0128
KRAB_A	ZNF7krab_A	hsZNF7krab_A	btZNF7krab_A	33.3	92.7	0.326	4.8889	0.0625	0.0219	0.0155	0.3504	0.1341	-2.43346	0.0075
KRAB_A	ZNF7krab_A	hsZNF7krab_A	cfZNF7krab_A	32	94	0.6126	4.8889	0.0291	0.0216	0.0153	0.7412	0.2995	-2.39954	0.0082
KRAB_A	ZNF7krab_A	hsZNF7krab_A	ptZNF7krab_A	34.5	91.5	0	4.8889	99	0	0	0	0	#DIV/0!	#DIV/0!
KRAB_A	ZNF7krab_A	hsZNF7krab_A	mmZNF7krab_A	31.8	94.2	0.3661	4.8889	0.0842	0.0326	0.0189	0.3867	0.1576	-2.23084	0.0128
KRAB_A	ZNF7krab_A	rnZNF7krab_A	btZNF7krab_A	30.8	95.2	0.7425	4.8889	0.0352	0.0322	0.0187	0.9134	0.4862	-1.81108	0.0351
KRAB_A	ZNF7krab_A	rnZNF7krab_A	cfZNF7krab_A	29.6	96.4	2.3569	4.8889	0.0098	0.0318	0.0185	3.2411	51.1425	-0.06275	0.4750
KRAB_A	ZNF7krab_A	rnZNF7krab_A	ptZNF7krab_A	32	94	0.3416	4.8889	0.0925	0.0326	0.0189	0.3526	0.1272	-2.4884	0.0064
KRAB_A	ZNF7krab_A	rnZNF7krab_A	mmZNF7krab_A	29.4	96.6	0.3028	4.8889	0.0577	0.021	0.0149	0.3639	0.1537	-2.22056	0.0132
KRAB_A	ZNF7krab_A	rnZNF7krab_A	hsZNF7krab_A	32	94	0.3416	4.8889	0.0925	0.0326	0.0189	0.3526	0.1272	-2.4884	0.0064
KRAB_A	ZNF7krab_A	mmulZNF7krab_A	btZNF7krab_A	33.3	92.7	0.326	4.8889	0.0625	0.0219	0.0155	0.3504	0.1341	-2.43346	0.0075
KRAB_A	ZNF7krab_A	mmulZNF7krab_A	cfZNF7krab_A	32	94	0.6126	4.8889	0.0291	0.0216	0.0153	0.7412	0.2995	-2.39954	0.0082
KRAB_A	ZNF7krab_A	mmulZNF7krab_A	ptZNF7krab_A	34.5	91.5	0	4.8889	99	0	0	0	0	#DIV/0!	#DIV/0!
KRAB_A	ZNF7krab_A	mmulZNF7krab_A	mmZNF7krab_A	31.8	94.2	0.3661	4.8889	0.0842	0.0326	0.0189	0.3867	0.1576	-2.23084	0.0128
KRAB_A	ZNF7krab_A	mmulZNF7krab_A	hsZNF7krab_A	34.5	91.5	0	4.8889	99	0	0	0	0	#DIV/0!	#DIV/0!
KRAB_A	ZNF7krab_A	mmulZNF7krab_A	rnZNF7krab_A	32	94	0.3416	4.8889	0.0925	0.0326	0.0189	0.3526	0.1272	-2.4884	0.0064
KRAB_A	ZNF7krab_A	mfasZNF7krab_A	btZNF7krab_A	33.3	92.7	0.326	4.8889	0.0625	0.0219	0.0155	0.3504	0.1341	-2.43346	0.0075
KRAB_A	ZNF7krab_A	mfasZNF7krab_A	cfZNF7krab_A	32	94	0.6126	4.8889	0.0291	0.0216	0.0153	0.7412	0.2995	-2.39954	0.0082
KRAB_A	ZNF7krab_A	mfasZNF7krab_A	ptZNF7krab_A	34.5	91.5	0	4.8889	99	0	0	0	0	#DIV/0!	#DIV/0!
KRAB_A	ZNF7krab_A	mfasZNF7krab_A	mmZNF7krab_A	31.8	94.2	0.3661	4.8889	0.0842	0.0326	0.0189	0.3867	0.1576	-2.23084	0.0128
KRAB_A	ZNF7krab_A	mfasZNF7krab_A	hsZNF7krab_A	34.5	91.5	0	4.8889	99	0	0	0	0	#DIV/0!	#DIV/0!
KRAB_A	ZNF7krab_A	mfasZNF7krab_A	rnZNF7krab_A	32	94	0.3416	4.8889	0.0925	0.0326	0.0189	0.3526	0.1272	-2.4884	0.0064
KRAB_A	ZNF250krab_A	rnZNF250krab_A	hsZNF250krab_A	35.4	90.6	0.6139	2.75	0.0748	0.0457	0.0231	0.6113	0.2199	-2.558	0.0053
KRAB_A	ZNF250krab_A	ptZNF250krab_A	hsZNF250krab_A	33.6	92.4	0.0243	2.75	0	0	0	0.0303	0.0305	-0.99344	0.1602
KRAB_A	ZNF250krab_A	ptZNF250krab_A	rnZNF250krab_A	34.9	91.1	0.5539	2.75	0.0831	0.0455	0.023	0.5472	0.1927	-2.58518	0.0049
KRAB_A	ZNF250krab_A	mmZNF250krab_A	hsZNF250krab_A	34.4	91.6	0.7119	2.75	0.0794	0.057	0.0259	0.7179	0.2666	-2.46738	0.0068
KRAB_A	ZNF250krab_A	mmZNF250krab_A	rnZNF250krab_A	35.8	90.2	0.1603	2.75	0.0698	0.0112	0.0112	0.16	0.0767	-1.91967	0.0274
KRAB_A	ZNF250krab_A	mmZNF250krab_A	ptZNF250krab_A	34	92	0.6418	2.75	0.0886	0.0567	0.0258	0.64	0.2278	-2.54431	0.0055
KRAB_A	ZNF250krab_A	mmulZNF250krab_A	hsZNF250krab_A	35.2	90.8	0.162	2.75	0	0	0	0.1931	0.0819	-2.35775	0.0092
KRAB_A	ZNF250krab_A	mmulZNF250krab_A	rnZNF250krab_A	36.8	89.2	0.8567	2.75	0.0536	0.0464	0.0235	0.8661	0.3704	-2.20857	0.0136
KRAB_A	ZNF250krab_A	mmulZNF250krab_A	ptZNF250krab_A	35	91	0.1321	2.75	0	0	0	0.1587	0.0732	-2.16803	0.0151
KRAB_A	ZNF250krab_A	mmulZNF250krab_A	mmZNF250krab_A	36.2	89.8	0.7663	2.75	0.078	0.0582	0.0264	0.7453	0.2779	-2.46139	0.0069
KRAB_A	ZNF250krab_A	cfZNF250krab_A	hsZNF250krab_A	30.9	95.1	0.5759	2.75	0.0891	0.0548	0.0249	0.6147	0.2504	-2.22505	0.0130
KRAB_A	ZNF250krab_A	cfZNF250krab_A	rnZNF250krab_A	32.1	93.9	0.5985	2.75	0.0301	0.0217	0.0154	0.7194	0.2797	-2.49069	0.0064
KRAB_A	ZNF250krab_A	cfZNF250krab_A	ptZNF250krab_A	30.3	95.7	0.5139	2.75	0.1007	0.0544	0.0247	0.5405	0.2133	-2.26382	0.0118
KRAB_A	ZNF250krab_A	cfZNF250krab_A	mmZNF250krab_A	30.2	95.8	0.5946	2.75	0.0133	0.0105	0.0106	0.7934	0.319	-2.45288	0.0071
KRAB_A	ZNF250krab_A	cfZNF250krab_A	mmulZNF250krab_A	33	93	0.8209	2.75	0.0633	0.0561	0.0255	0.8862	0.4394	-1.88599	0.0296
KRAB_A	ZNF252krab_A	mmulZNF252krab_A	cfZNF252krab_A	39.2	86.8	0.5329	7.3381	0.4385	0.127	0.0418	0.2897	0.11	-1.38263	0.0834
KRAB_A	ZNF252krab_A	ptZNF252krab_A	cfZNF252krab_A	38.4	87.6	0.706	7.3381	0.5142	0.1827	0.0516	0.3554	0.1379	-1.17293	0.1204
KRAB_A	ZNF252krab_A	ptZNF252krab_A	mmulZNF252krab_A	36.3	89.7	0.2002	7.3381	1.2292	0.0705	0.0294	0.0574	0.0411	0.259237	0.3977
KRAB_A	ZNF252krab_A	hsZNF252krab_A	cfZNF252krab_A	38.5	87.5	0.7057	7.3381	0.5171	0.183	0.0517	0.3539	0.1372	-1.16562	0.1219
KRAB_A	ZNF252krab_A	hsZNF252krab_A	mmulZNF252krab_A	36.4	89.6	0.2002	7.3381	1.2345	0.0706	0.0294	0.0572	0.0409	0.26603	0.3951
KRAB_A	ZNF252krab_A	hsZNF252krab_A	ptZNF252krab_A	35.6	90.4	0.0483	7.3381	99	0.0225	0.0159	0	0	1.415094	0.0785
KRAB_A	ZNF252krab_A	mondomZNF252krab_A	cfZNF252krab_A	35.6	90.4	1.0684	7.3381	0.3225	0.2234	0.0573	0.6927	0.2708	-1.69547	0.0450
KRAB_A	ZNF252krab_A	mondomZNF252krab_A	mmulZNF252krab_A	33.3	92.7	1.1051	7.3381	0.265	0.2124	0.0544	0.8016	0.3339	-1.74164	0.0408
KRAB_A	ZNF252krab_A	mondomZNF252krab_A	ptZNF252krab_A	32.7	93.3	1.19	7.3381	0.3456	0.266	0.0629	0.7695	0.3242	-1.52462	0.0637
KRAB_A	ZNF252krab_A	mondomZNF252krab_A	hsZNF252krab_A	32.8	93.2	1.1869	7.3381	0.3497	0.2665	0.063	0.7621	0.3185	-1.52647	0.0634

Domain	Gene Fragment	Seq1	Seq2	S	N	t	Kappa	Omega	dN	dN_SE	dS	dS_SE	Z Score	P Value
KRAB_B	ZNF251krab_B	rnZNF251krab_B	ptZNF251krab_B	35.7	78.3	0.591	3.2941	0.4432	0.1414	0.0464	0.319	0.1199	-1.3814	0.0836
KRAB_B	ZNF251krab_B	hsZNF251krab_B	ptZNF251krab_B	35.4	78.6	0.0268	3.2941	0	0	0	0.0288	0.0289	-0.99654	0.1595
KRAB_B	ZNF251krab_B	hsZNF251krab_B	rnZNF251krab_B	36	78	0.5472	3.2941	0.5259	0.142	0.0466	0.27	0.1043	-1.12048	0.1313
KRAB_B	ZNF251krab_B	mmZNF251krab_B	ptZNF251krab_B	38.4	75.6	0.7036	3.2941	0.2968	0.1304	0.0449	0.4395	0.1577	-1.88513	0.0297
KRAB_B	ZNF251krab_B	mmZNF251krab_B	rnZNF251krab_B	39.1	74.9	0.1645	3.2941	0.5097	0.0412	0.024	0.0808	0.0474	-0.74535	0.2280
KRAB_B	ZNF251krab_B	mmZNF251krab_B	hsZNF251krab_B	38.6	75.4	0.6477	3.2941	0.3421	0.1308	0.045	0.3823	0.137	-1.74409	0.0406
KRAB_B	ZNF34krab_B	btZNF34krab_B	cfZNF34krab_B	41.9	69.1	0.8374	10.8031	0.9597	0.2748	0.0785	0.2863	0.1026	-0.08902	0.4645
KRAB_B	ZNF34krab_B	mmulZNF34krab_B	cfZNF34krab_B	41.1	69.9	0.5576	10.8031	1.0642	0.1901	0.0599	0.1786	0.073	0.121783	0.4515
KRAB_B	ZNF34krab_B	mmulZNF34krab_B	btZNF34krab_B	41.6	69.4	1.059	10.8031	0.6762	0.2993	0.0834	0.4427	0.1417	-0.87215	0.1916
KRAB_B	ZNF34krab_B	hsZNF34krab_B	cfZNF34krab_B	41.2	69.8	0.5209	10.8031	0.9617	0.1711	0.0558	0.1779	0.0727	-0.0742	0.4704
KRAB_B	ZNF34krab_B	hsZNF34krab_B	btZNF34krab_B	41.6	69.4	1.0141	10.8031	0.6237	0.2757	0.0779	0.4421	0.1415	-1.03017	0.1515
KRAB_B	ZNF34krab_B	hsZNF34krab_B	mmulZNF34krab_B	39.3	71.7	0.0273	10.8031	99	0.0141	0.0141	0	0	1	0.1587
KRAB_B	ZNF34krab_B	ptZNF34krab_B	cfZNF34krab_B	41.2	69.8	0.5209	10.8031	0.9617	0.1711	0.0558	0.1779	0.0727	-0.0742	0.4704
KRAB_B	ZNF34krab_B	ptZNF34krab_B	btZNF34krab_B	41.6	69.4	1.0141	10.8031	0.6237	0.2757	0.0779	0.4421	0.1415	-1.03017	0.1515
KRAB_B	ZNF34krab_B	ptZNF34krab_B	mmulZNF34krab_B	39.3	71.7	0.0273	10.8031	99	0.0141	0.0141	0	0	1	0.1587
KRAB_B	ZNF34krab_B	ptZNF34krab_B	hsZNF34krab_B	39.4	71.6	0	10.8031	99	0	0	0	0	#DIV/0!	#DIV/0!
KRAB_B	ZNF517krab_B	hsZNF517krab_B	mmulZNF517krab_B	37.5	76.5	0.0269	2.7841	0	0	0	0.0272	0.0274	-0.9927	0.1604
KRAB_B	ZNF517krab_B	cfZNF517krab_B	mmulZNF517krab_B	36.1	77.9	0.4357	2.7841	0.9111	0.1409	0.0458	0.1546	0.0721	-0.16039	0.4363
KRAB_B	ZNF517krab_B	cfZNF517krab_B	hsZNF517krab_B	36.8	77.2	0.468	2.7841	0.7709	0.1423	0.0463	0.1846	0.0787	-0.46326	0.3216
KRAB_B	ZNF517krab_B	ptZNF517krab_B	mmulZNF517krab_B	36.8	77.2	0.0547	2.7841	0	0	0	0.0565	0.0405	-1.39506	0.0815
KRAB_B	ZNF517krab_B	ptZNF517krab_B	hsZNF517krab_B	37.5	76.5	0.0268	2.7841	0	0	0	0.0271	0.0273	-0.99267	0.1604
KRAB_B	ZNF517krab_B	ptZNF517krab_B	cfZNF517krab_B	36	78	0.5049	2.7841	0.6172	0.1407	0.0457	0.228	0.0919	-0.85058	0.1975
KRAB_B	ZNF7krab_B	mfasZNF7krab_B	mmulZNF7krab_B	34.5	91.5	0	4.8889	99	0	0	0	0	#DIV/0!	#DIV/0!
KRAB_B	ZNF7krab_B	btZNF7krab_B	cfZNF7krab_B	31.9	85.1	0.4975	2.1637	0.2322	0.0872	0.0335	0.3756	0.1477	-1.90424	0.0284
KRAB_B	ZNF7krab_B	mmZNF7krab_B	cfZNF7krab_B	33.1	83.9	1.1915	2.1637	0.0967	0.1091	0.0383	1.1276	0.8011	-1.26993	0.1021
KRAB_B	ZNF7krab_B	mmZNF7krab_B	btZNF7krab_B	34.3	82.7	1.0232	2.1637	0.1137	0.1038	0.0375	0.913	0.498	-1.62031	0.0526
KRAB_B	ZNF7krab_B	ptZNF7krab_B	cfZNF7krab_B	31.6	85.4	0.513	2.1637	0.4102	0.1232	0.0407	0.3003	0.1301	-1.29917	0.0969
KRAB_B	ZNF7krab_B	ptZNF7krab_B	btZNF7krab_B	33.4	83.6	0.5484	2.1637	0.2402	0.0961	0.0359	0.4	0.1638	-1.81229	0.0350
KRAB_B	ZNF7krab_B	ptZNF7krab_B	mmZNF7krab_B	34	83	0.6193	2.1637	0.0848	0.0499	0.0252	0.5883	0.2581	-2.07614	0.0189
KRAB_B	ZNF7krab_B	mfasZNF7krab_B	cfZNF7krab_B	30.9	86.1	0.5627	2.1637	0.3307	0.1222	0.0404	0.3695	0.1579	-1.5173	0.0646
KRAB_B	ZNF7krab_B	mfasZNF7krab_B	btZNF7krab_B	33	84	0.4965	2.1637	0.2779	0.0956	0.0357	0.3438	0.1413	-1.70303	0.0443
KRAB_B	ZNF7krab_B	mfasZNF7krab_B	mmZNF7krab_B	33.6	83.4	0.545	2.1637	0.0975	0.0496	0.0251	0.509	0.2148	-2.12428	0.0168
KRAB_B	ZNF7krab_B	mfasZNF7krab_B	ptZNF7krab_B	32.8	84.2	0.1433	2.1637	0	0	0	0.1702	0.0787	-2.16264	0.0153
KRAB_B	ZNF7krab_B	rnZNF7krab_B	cfZNF7krab_B	33.6	83.4	1.0277	2.1637	0.1393	0.1236	0.0411	0.8871	0.4895	-1.55429	0.0601
KRAB_B	ZNF7krab_B	rnZNF7krab_B	btZNF7krab_B	34.6	82.4	0.9187	2.1637	0.1568	0.1182	0.0403	0.7539	0.3578	-1.76553	0.0387
KRAB_B	ZNF7krab_B	rnZNF7krab_B	mmZNF7krab_B	34.7	82.3	0.1385	2.1637	0.0969	0.0123	0.0123	0.1265	0.0655	-1.71356	0.0433
KRAB_B	ZNF7krab_B	rnZNF7krab_B	ptZNF7krab_B	34.4	82.6	0.5705	2.1637	0.1278	0.0632	0.0286	0.4946	0.2086	-2.04891	0.0202
KRAB_B	ZNF7krab_B	rnZNF7krab_B	mfasZNF7krab_B	34.1	82.9	0.5699	2.1637	0.1264	0.063	0.0285	0.4982	0.2092	-2.06127	0.0196
KRAB_B	ZNF7krab_B	mmulZNF7krab_B	cfZNF7krab_B	30.9	86.1	0.5627	2.1637	0.3307	0.1222	0.0404	0.3695	0.1579	-1.5173	0.0646
KRAB_B	ZNF7krab_B	mmulZNF7krab_B	btZNF7krab_B	33	84	0.4965	2.1637	0.2779	0.0956	0.0357	0.3438	0.1413	-1.70303	0.0443
KRAB_B	ZNF7krab_B	mmulZNF7krab_B	mmZNF7krab_B	33.6	83.4	0.545	2.1637	0.0975	0.0496	0.0251	0.509	0.2148	-2.12428	0.0168
KRAB_B	ZNF7krab_B	mmulZNF7krab_B	ptZNF7krab_B	32.8	84.2	0.1433	2.1637	0	0	0	0.1702	0.0787	-2.16264	0.0153
KRAB_B	ZNF7krab_B	mmulZNF7krab_B	mfasZNF7krab_B	32.2	84.8	0	2.1637	99	0	0	0	0	#DIV/0!	#DIV/0!
KRAB_B	ZNF7krab_B	mmulZNF7krab_B	rnZNF7krab_B	34.1	82.9	0.5699	2.1637	0.1264	0.063	0.0285	0.4982	0.2092	-2.06127	0.0196
KRAB_B	ZNF7krab_B	hsZNF7krab_B	cfZNF7krab_B	31.6	85.4	0.513	2.1637	0.4102	0.1232	0.0407	0.3003	0.1301	-1.29917	0.0969
KRAB_B	ZNF7krab_B	hsZNF7krab_B	btZNF7krab_B	33.4	83.6	0.5484	2.1637	0.2402	0.0961	0.0359	0.4	0.1638	-1.81229	0.0350
KRAB_B	ZNF7krab_B	hsZNF7krab_B	mmZNF7krab_B	34	83	0.6193	2.1637	0.0848	0.0499	0.0252	0.5883	0.2581	-2.07614	0.0189
KRAB_B	ZNF7krab_B	hsZNF7krab_B	ptZNF7krab_B	33.4	83.6	0	2.1637	99	0	0	0	0	#DIV/0!	#DIV/0!
KRAB_B	ZNF7krab_B	hsZNF7krab_B	mfasZNF7krab_B	32.8	84.2	0.1433	2.1637	0	0	0	0.1702	0.0787	-2.16264	0.0153
KRAB_B	ZNF7krab_B	hsZNF7krab_B	rnZNF7krab_B	34.4	82.6	0.5705	2.1637	0.1278	0.0632	0.0286	0.4946	0.2086	-2.04891	0.0202
KRAB_B	ZNF7krab_B	hsZNF7krab_B	mmulZNF7krab_B	32.8	84.2	0.1433	2.1637	0	0	0	0.1702	0.0787	-2.16264	0.0153
KRAB_B	ZNF250krab_B	mmZNF250krab_B	mmulZNF250krab_B	33.9	80.1	0.8252	3.7217	0.2959	0.161	0.0495	0.5441	0.2331	-1.60765	0.0540
KRAB_B	ZNF250krab_B	hsZNF250krab_B	mmulZNF250krab_B	29.2	84.8	0.0269	3.7217	0	0	0	0.035	0.0352	-0.99432	0.1600
KRAB_B	ZNF250krab_B	hsZNF250krab_B	mmZNF250krab_B	33.4	80.6	0.757	3.7217	0.3363	0.1598	0.0491	0.4753	0.1994	-1.53635	0.0622
KRAB_B	ZNF250krab_B	cfZNF250krab_B	mmulZNF250krab_B	33.5	80.5	0.6892	3.7217	0.7528	0.2095	0.0585	0.2783	0.1142	-0.53619	0.2959
KRAB_B	ZNF250krab_B	cfZNF250krab_B	mmZNF250krab_B	37.4	76.6	0.7791	3.7217	0.4109	0.1766	0.0531	0.4299	0.1701	-1.42147	0.0776
KRAB_B	ZNF250krab_B	cfZNF250krab_B	hsZNF250krab_B	32.8	81.2	0.7365	3.7217	0.6099	0.2073	0.0579	0.3399	0.1369	-0.89208	0.1862
KRAB_B	ZNF250krab_B	ptZNF250krab_B	mmulZNF250krab_B	29.2	84.8	0.0269	3.7217	0	0	0	0.035	0.0352	-0.99432	0.1600
KRAB_B	ZNF250krab_B	ptZNF250krab_B	mmZNF250krab_B	33.4	80.6	0.757	3.7217	0.3363	0.1598	0.0491	0.4753	0.1994	-1.53635	0.0622
KRAB_B	ZNF250krab_B	ptZNF250krab_B	hsZNF250krab_B	28.4	85.6	0	3.7217	99	0	0	0	0	#DIV/0!	#DIV/0!
KRAB_B	ZNF250krab_B	ptZNF250krab_B	cfZNF250krab_B	32.8	81.2	0.7365	3.7217	0.6099	0.2073	0.0579	0.3399	0.1369	-0.89208	0.1862
KRAB_B	ZNF250krab_B	rnZNF250krab_B	mmulZNF250krab_B	35.2	78.8	0.948	3.7217	0.2498	0.1639	0.0504	0.6562	0.2799	-1.731	0.0417
KRAB_B	ZNF250krab_B	rnZNF250krab_B	mmZNF250krab_B	38.4	75.6	0.238	3.7217	0.1475	0.0269	0.0191	0.1825	0.0808	-1.87409	0.0305
KRAB_B	ZNF250krab_B	rnZNF250krab_B	hsZNF250krab_B	34.7	79.3	0.8674	3.7217	0.2817	0.1627	0.0501	0.5777	0.2361	-1.71944	0.0428
KRAB_B	ZNF250krab_B	rnZNF250krab_B	cfZNF250krab_B	37.7	76.3	0.9712	3.7217	0.286	0.1774	0.0533	0.6203	0.2513	-1.72408	0.0423
KRAB_B	ZNF250krab_B	rnZNF250krab_B	ptZNF250krab_B	34.7	79.3	0.8674	3.7217	0.2817	0.1627	0.0501	0.5777	0.2361	-1.71944	0.0428
KRAB_B	ZNF252krab_B	ptZNF252krab_B	cfZNF252krab_B	24.1	74.9	2.085	2.3103	1.249	0.7304	0.1628	0.5848	0.2204	0.531373	0.2976
KRAB_B	ZNF252krab_B	mmulZNF252krab_B	cfZNF252krab_B	23.6	75.4	0.9806	2.3103	1.0573	0.3312	0.0807	0.3132	0.139	0.11199	0.4554
KRAB_B	ZNF252krab_B	mmulZNF252krab_B	ptZNF252krab_B	23.9	75.1	1.5419	2.3103	1.2922	0.5436	0.1254	0.4207	0.1684	0.585346	0.2792
KRAB_B	ZNF252krab_B	hsZNF252krab_B	cfZNF252krab_B	23.7	75.3	2.0115	2.3103	1.1557	0.6929	0.156	0.5995	0.2273	0.338795	0.3674
KRAB_B	ZNF252krab_B	hsZNF252krab_B	ptZNF252krab_B	24.3	74.7	0.0306	2.3103	99	0.0135	0.0135	0	0	1	0.1587
KRAB_B	ZNF252krab_B	hsZNF252krab_B	mmulZNF252krab_B	23.4	75.6	1.5528	2.3103	1.454	0.5588	0.1291	0.3843	0.1596	0.850068	0.1976
KRAB_B	ZNF252krab_B	mondomZNF252krab_B	cfZNF252krab_B	26.7	72.3	3.5005	2.3103	0.3043	0.722	0.1611	2.3725	2.0593	-0.79904	0.2121
KRAB_B	ZNF252krab_B	mondomZNF252krab_B	ptZNF252krab_B	26.5	72.5	2.5704	2.3103	0.582	0.7185	0.1634	1.2345	0.6712	-0.74696	0.2275
KRAB_B	ZNF252krab_B	mondomZNF252krab_B	mmulZNF252krab_B	26	73	2.7172	2.3103	0.5623	0.7518	0.1732	1.3369	0.5642	-0.99138	0.1607
KRAB_B	ZNF252krab_B	mondomZNF252krab_B	hsZNF252krab_B	26.3	72.7	2.6582	2.3103	0.5917	0.7487	0.1678	1.2652	0.7126	-0.70551	0.2402

Domain	Gene Fragment	Seq1	Seq2	S	N	t	Kappa	Omega	dN	dN_SE	dS	dS_SE	Z Score	P Value
Link	ZNF251link	ptZNF251link	hsZNF251link	85	239	0.0093	2.7507	99	0.0042	0.0042	0	0	1	0.1587
Link	ZNF251link	rnZNF251link	hsZNF251link	86.5	237.5	1.3273	2.7507	0.5197	0.3549	0.048	0.6829	0.169	-1.86698	0.0310
Link	ZNF251link	rnZNF251link	ptZNF251link	86.4	237.6	1.3278	2.7507	0.5181	0.3547	0.048	0.6846	0.1697	-1.87063	0.0307
Link	ZNF251link	mmZNF251link	hsZNF251link	87.6	236.4	1.2704	2.7507	0.6734	0.3744	0.0503	0.5559	0.129	-1.31085	0.0950
Link	ZNF251link	mmZNF251link	ptZNF251link	87.5	236.5	1.2707	2.7507	0.6711	0.3741	0.0503	0.5574	0.1295	-1.31941	0.0935
Link	ZNF251link	mmZNF251link	rnZNF251link	88.7	235.3	0.3787	2.7507	0.6551	0.1103	0.0231	0.1684	0.0501	-1.05313	0.1461
Link	ZNF251link	mmulZNF251link	hsZNF251link	82.9	241.1	0.1339	2.7507	1.2669	0.0472	0.0144	0.0372	0.0217	0.383977	0.3505
Link	ZNF251link	mmulZNF251link	ptZNF251link	82.7	241.3	0.1439	2.7507	1.3832	0.0516	0.0151	0.0373	0.0218	0.539239	0.2949
Link	ZNF251link	mmulZNF251link	rnZNF251link	84.6	239.4	1.2567	2.7507	0.6546	0.3682	0.0491	0.5625	0.1355	-1.34817	0.0888
Link	ZNF251link	mmulZNF251link	mmZNF251link	85.7	238.3	1.2439	2.7507	0.7403	0.3794	0.0502	0.5125	0.1215	-1.01246	0.1557
Link	ZNF34link	btZNF34link	cfZNF34link	68.9	168.1	0.9951	4.4705	1.1374	0.3438	0.0565	0.3022	0.0843	0.409922	0.3409
Link	ZNF34link	hsZNF34link	cfZNF34link	66.9	170.1	0.9591	4.4705	1.6733	0.3607	0.0582	0.2155	0.0664	1.644467	0.0500
Link	ZNF34link	hsZNF34link	btZNF34link	71.2	165.8	0.8817	4.4705	1.2429	0.3122	0.0534	0.2512	0.0742	0.667267	0.2523
Link	ZNF34link	mmulZNF34link	cfZNF34link	66.5	170.5	0.995	4.4705	1.3864	0.3598	0.0581	0.2595	0.0753	1.054581	0.1458
Link	ZNF34link	mmulZNF34link	btZNF34link	71.1	165.9	0.9611	4.4705	1.1263	0.3315	0.0558	0.2944	0.083	0.370951	0.3553
Link	ZNF34link	mmulZNF34link	hsZNF34link	69.2	167.8	0.1579	4.4705	1.2459	0.0559	0.0189	0.0448	0.0262	0.343594	0.3656
Link	ZNF34link	ptZNF34link	cfZNF34link	66.6	170.4	0.9204	4.4705	1.7953	0.3504	0.057	0.1952	0.062	1.842794	0.0327
Link	ZNF34link	ptZNF34link	btZNF34link	71.1	165.9	0.8429	4.4705	1.3182	0.3029	0.0524	0.2298	0.0693	0.841384	0.2001
Link	ZNF34link	ptZNF34link	hsZNF34link	69.6	167.4	0.0255	4.4705	0.4139	0.006	0.006	0.0145	0.0145	-0.54167	0.2940
Link	ZNF34link	ptZNF34link	mmulZNF34link	69.2	167.8	0.1581	4.4705	0.8138	0.0494	0.0177	0.0607	0.031	-0.31655	0.3758
Link	ZNF517link	ptZNF517link	mmulZNF517link	69.2	170.8	0.1568	8.8173	1.2394	0.0554	0.0189	0.0447	0.026	0.332881	0.3696
Link	ZNF517link	cfZNF517link	mmulZNF517link	73.1	166.9	1.4827	8.8173	0.74	0.4465	0.0676	0.6034	0.1422	-0.9965	0.1595
Link	ZNF517link	cfZNF517link	ptZNF517link	72.6	167.4	1.5466	8.8173	0.856	0.4906	0.0731	0.5731	0.1335	-0.54204	0.2939
Link	ZNF517link	hsZNF517link	mmulZNF517link	68.5	171.5	0.1568	8.8173	1.2212	0.0551	0.0188	0.0451	0.0263	0.309325	0.3785
Link	ZNF517link	hsZNF517link	ptZNF517link	69.1	170.9	0.0252	8.8173	99	0.0118	0.0084	0	0	1.404762	0.0800
Link	ZNF517link	hsZNF517link	cfZNF517link	72.3	167.7	1.5216	8.8173	0.8288	0.4775	0.0713	0.5761	0.1345	-0.6477	0.2586
Link	ZNF7link	mmZNF7link	btZNF7link	64.8	178.2	2.0215	4.0656	0.8182	0.6361	0.0924	0.7774	0.2378	-0.55386	0.2898
Link	ZNF7link	mmulZNF7link	btZNF7link	64.1	178.9	1.2733	4.0656	0.8115	0.3999	0.0613	0.4928	0.1381	-0.61485	0.2693
Link	ZNF7link	mmulZNF7link	mmZNF7link	64.3	178.7	1.6268	4.0656	1.1322	0.5596	0.0832	0.4943	0.1263	0.43176	0.3330
Link	ZNF7link	mfasZNF7link	btZNF7link	64.1	178.9	1.2733	4.0656	0.8115	0.3999	0.0613	0.4928	0.1381	-0.61485	0.2693
Link	ZNF7link	mfasZNF7link	mmZNF7link	64.3	178.7	1.6268	4.0656	1.1322	0.5596	0.0832	0.4943	0.1263	0.43176	0.3330
Link	ZNF7link	mfasZNF7link	mmulZNF7link	62.3	180.7	0	4.0656	99	0	0	0	0	#DIV/0!	#DIV/0!
Link	ZNF7link	rnZNF7link	btZNF7link	64.7	178.3	2.0347	4.0656	0.9103	0.6609	0.0944	0.726	0.2191	-0.27287	0.3925
Link	ZNF7link	rnZNF7link	mmZNF7link	65.8	177.2	0.4261	4.0656	1.4542	0.1552	0.0329	0.1067	0.0431	0.894472	0.1855
Link	ZNF7link	rnZNF7link	mmulZNF7link	64.3	178.7	1.8555	4.0656	0.7526	0.569	0.0843	0.7561	0.2302	-0.76321	0.2227
Link	ZNF7link	rnZNF7link	mfasZNF7link	64.3	178.7	1.8555	4.0656	0.7526	0.569	0.0843	0.7561	0.2302	-0.76321	0.2227
Link	ZNF7link	hsZNF7link	btZNF7link	65.1	177.9	1.1119	4.0656	1.042	0.3747	0.0587	0.3596	0.1028	0.127557	0.4492
Link	ZNF7link	hsZNF7link	mmZNF7link	65.1	177.9	1.5972	4.0656	1.095	0.5451	0.0808	0.4978	0.1272	0.313883	0.3768
Link	ZNF7link	hsZNF7link	mmulZNF7link	63.9	179.1	0.184	4.0656	0.3356	0.0403	0.0154	0.1202	0.0473	-1.60623	0.0541
Link	ZNF7link	hsZNF7link	mfasZNF7link	63.9	179.1	0.184	4.0656	0.3356	0.0403	0.0154	0.1202	0.0473	-1.60623	0.0541
Link	ZNF7link	hsZNF7link	rnZNF7link	65.3	177.7	1.7835	4.0656	0.7678	0.5498	0.0814	0.7161	0.2089	-0.74175	0.2291
Link	ZNF7link	cfZNF7link	btZNF7link	69.3	173.7	1.2118	4.0656	1.0997	0.4147	0.0639	0.3771	0.0999	0.317063	0.3756
Link	ZNF7link	cfZNF7link	mmZNF7link	68	175	2.014	4.0656	1.032	0.6772	0.0998	0.6562	0.1723	0.105466	0.4580
Link	ZNF7link	cfZNF7link	mmulZNF7link	65.8	177.2	1.2913	4.0656	0.7096	0.3875	0.0615	0.546	0.1615	-0.91717	0.1795
Link	ZNF7link	cfZNF7link	mfasZNF7link	65.8	177.2	1.2913	4.0656	0.7096	0.3875	0.0615	0.546	0.1615	-0.91717	0.1795
Link	ZNF7link	cfZNF7link	rnZNF7link	66.3	176.7	2.0265	4.0656	1.0893	0.691	0.1018	0.6343	0.1722	0.283443	0.3884
Link	ZNF7link	cfZNF7link	hsZNF7link	67.8	175.2	1.212	4.0656	0.7337	0.3669	0.0588	0.5	0.1436	-0.85776	0.1955
Link	ZNF7link	ptZNF7link	btZNF7link	65.6	177.4	1.1323	4.0656	1.0819	0.3853	0.0599	0.3561	0.1016	0.247577	0.4022
Link	ZNF7link	ptZNF7link	mmZNF7link	65.7	177.3	1.6033	4.0656	1.1486	0.5538	0.0827	0.4821	0.1234	0.482669	0.3147
Link	ZNF7link	ptZNF7link	mmulZNF7link	64.6	178.4	0.1562	4.0656	0.3455	0.0346	0.0143	0.1002	0.0421	-1.47541	0.0701
Link	ZNF7link	ptZNF7link	mfasZNF7link	64.6	178.4	0.1562	4.0656	0.3455	0.0346	0.0143	0.1002	0.0421	-1.47541	0.0701
Link	ZNF7link	ptZNF7link	rnZNF7link	65.7	177.3	1.8617	4.0656	0.7618	0.5721	0.0856	0.7511	0.2263	-0.73983	0.2297
Link	ZNF7link	ptZNF7link	hsZNF7link	65.8	177.2	0.05	4.0656	1.118	0.0172	0.0099	0.0153	0.0154	0.103782	0.4587
Link	ZNF7link	ptZNF7link	cfZNF7link	68.3	174.7	1.2063	4.0656	0.8302	0.3802	0.0611	0.458	0.1299	-0.54196	0.2939
Link	ZNF250link	cfZNF250link	mmulZNF250link	51.5	116.5	2.3025	2.941	0.8097	0.7159	0.123	0.8842	0.2961	-0.5249	0.2998
Link	ZNF250link	rnZNF250link	mmulZNF250link	49.7	118.3	1.3273	2.941	0.5166	0.3465	0.0677	0.6709	0.2329	-1.33751	0.0905
Link	ZNF250link	rnZNF250link	cfZNF250link	52.9	115.1	2.7245	2.941	1.2057	0.9597	0.1762	0.796	0.2069	0.602367	0.2735
Link	ZNF250link	hsZNF250link	mmulZNF250link	48.4	119.6	0.1102	2.941	2.0602	0.0431	0.0195	0.0209	0.021	0.774667	0.2193
Link	ZNF250link	hsZNF250link	cfZNF250link	50.7	117.3	2.4581	2.941	0.882	0.7876	0.1358	0.893	0.3058	-0.31501	0.3764
Link	ZNF250link	hsZNF250link	rnZNF250link	49.4	118.6	1.3247	2.941	0.6608	0.3837	0.0741	0.5807	0.192	-0.95723	0.1692
Link	ZNF250link	ptZNF250link	mmulZNF250link	48.4	119.6	0.1102	2.941	2.0602	0.0431	0.0195	0.0209	0.021	0.774667	0.2193
Link	ZNF250link	ptZNF250link	cfZNF250link	50.7	117.3	2.4581	2.941	0.882	0.7876	0.1358	0.893	0.3058	-0.31501	0.3764
Link	ZNF250link	ptZNF250link	rnZNF250link	49.4	118.6	1.3247	2.941	0.6608	0.3837	0.0741	0.5807	0.192	-0.95723	0.1692
Link	ZNF250link	ptZNF250link	hsZNF250link	48.1	119.9	0	2.941	99	0	0	0	0	#DIV/0!	#DIV/0!
Link	ZNF250link	mmZNF250link	mmulZNF250link	51.9	116.1	1.3546	2.941	0.6497	0.387	0.0744	0.5957	0.1904	-1.02094	0.1536
Link	ZNF250link	mmZNF250link	cfZNF250link	53.1	114.9	2.7248	2.941	1.1751	0.9531	0.1748	0.8111	0.2112	0.517957	0.3022
Link	ZNF250link	mmZNF250link	rnZNF250link	53.6	114.4	0.446	2.941	0.4198	0.1032	0.0318	0.2458	0.0803	-1.65109	0.0494
Link	ZNF250link	mmZNF250link	hsZNF250link	51.7	116.3	1.4714	2.941	0.6776	0.4279	0.0818	0.6315	0.2062	-0.91781	0.1794
Link	ZNF250link	mmZNF250link	ptZNF250link	51.7	116.3	1.4714	2.941	0.6776	0.4279	0.0818	0.6315	0.2062	-0.91781	0.1794
Link	ZNF252link	ptZNF252link	cfZNF252link	77.3	234.7	0.9622	2.8126	0.8973	0.3119	0.0445	0.3476	0.0939	-0.34356	0.3656
Link	ZNF252link	hsZNF252link	cfZNF252link	77.7	234.3	0.981	2.8126	0.8445	0.3127	0.0446	0.3702	0.0994	-0.52778	0.2988
Link	ZNF252link	hsZNF252link	ptZNF252link	76.2	235.8	0.0097	2.8126	0	0	0	0.0132	0.0133	-0.99248	0.1605
Link	ZNF252link	mmulZNF252link	cfZNF252link	76	236	1.0757	2.8126	1.1361	0.3693	0.0505	0.3251	0.0912	0.423988	0.3358
Link	ZNF252link	mmulZNF252link	ptZNF252link	74.8	237.2	0.1933	2.8126	0.5129	0.0525	0.0153	0.1023	0.0404	-1.15277	0.1245
Link	ZNF252link	mmulZNF252link	hsZNF252link	75.3	236.7	0.2051	2.8126	0.4457	0.0526	0.0153	0.118	0.044	-1.40391	0.0802
Link	ZNF252link	mondomZNF252link	cfZNF252link	82.6	229.4	5.0566	2.8126	1.1754	1.7549	0.3723	1.493	0.5098	0.414877	0.3391
Link	ZNF252link	mondomZNF252link	ptZNF252link	80.7	231.3	4.5394	2.8126	1.2087	1.5839	0.3101	1.3104	0.3894	0.549429	0.2914
Link	ZNF252link	mondomZNF252link	hsZNF252link	81	231	4.5444	2.8126	1.229	1.5918	0.3148	1.2953	0.379	0.601803	0.2737
Link	ZNF252link	mondomZNF252link	mmulZNF252link	79.5	232.5	4.7024	2.8126	0.9168	1.532	0.275	1.671	0.8336	-0.15835	0.4371

Domain	Gene Fragment	Seq1	Seq2	S	N	t	Kappa	Omega	dN	dN_SE	dS	dS_SE	Z Score	P Value
ZnF	ZNF251ZnF	hsZNF251ZnF	mmZNF251ZnF	325.2	901.8	1.0923	3.3515	0.1239	0.1267	0.0127	1.0226	0.1635	-5.46305	2.3401E-08
ZnF	ZNF251ZnF	mmulZNF251ZnF	mmZNF251ZnF	326	901	1.2668	3.3515	0.101	0.1255	0.0126	1.2424	0.2557	-4.36272	6.4229E-06
ZnF	ZNF251ZnF	mmulZNF251ZnF	hsZNF251ZnF	321.3	905.7	0.0603	3.3515	0.2455	0.0111	0.0035	0.0453	0.0123	-2.67432	0.0037
ZnF	ZNF251ZnF	rnZNF251ZnF	mmZNF251ZnF	306.2	920.8	0.2206	3.3515	0.103	0.0232	0.0051	0.225	0.0321	-6.20873	2.6707E-10
ZnF	ZNF251ZnF	rnZNF251ZnF	hsZNF251ZnF	327.4	899.6	1.0832	3.3515	0.1366	0.1344	0.0132	0.9839	0.1567	-5.40205	3.2941E-08
ZnF	ZNF251ZnF	rnZNF251ZnF	mmulZNF251ZnF	327.8	899.2	1.1644	3.3515	0.1255	0.1356	0.0133	1.0808	0.1926	-4.89592	4.8923E-07
ZnF	ZNF251ZnF	ptZNF251ZnF	mmZNF251ZnF	325	902	1.0652	3.3515	0.1298	0.1279	0.0128	0.9854	0.1516	-5.63628	8.6882E-09
ZnF	ZNF251ZnF	ptZNF251ZnF	hsZNF251ZnF	323.2	903.8	0.0123	3.3515	0.5355	0.0033	0.0019	0.0062	0.0044	-0.60509	0.2726
ZnF	ZNF251ZnF	ptZNF251ZnF	mmulZNF251ZnF	321.1	905.9	0.0631	3.3515	0.1917	0.01	0.0033	0.0522	0.0133	-3.07955	0.0010
ZnF	ZNF251ZnF	ptZNF251ZnF	rnZNF251ZnF	327.2	899.8	1.0547	3.3515	0.1446	0.1364	0.0133	0.9432	0.1447	-5.55227	1.4099E-08
ZnF	ZNF34ZnF	mmulZNF34ZnF	ptZNF34ZnF	291.8	794.2	0.0946	4.4672	0.0632	0.0063	0.0028	0.1001	0.0199	-4.66759	1.5238E-06
ZnF	ZNF34ZnF	btZNF34ZnF	ptZNF34ZnF	277.6	808.4	0.5992	4.4672	0.0712	0.0461	0.0078	0.6471	0.0952	-6.29194	1.5676E-10
ZnF	ZNF34ZnF	btZNF34ZnF	mmulZNF34ZnF	279.6	806.4	0.6953	4.4672	0.0602	0.0462	0.0078	0.7672	0.1214	-5.92682	1.5443E-09
ZnF	ZNF34ZnF	hsZNF34ZnF	ptZNF34ZnF	290.9	795.1	0.0055	4.4672	0.3653	0.0013	0.0013	0.0034	0.0034	-0.57691	0.2820
ZnF	ZNF34ZnF	hsZNF34ZnF	mmulZNF34ZnF	292.5	793.5	0.0941	4.4672	0.0793	0.0076	0.0031	0.0958	0.0194	-4.48944	3.5706E-06
ZnF	ZNF34ZnF	hsZNF34ZnF	btZNF34ZnF	278.5	807.5	0.6103	4.4672	0.0723	0.0474	0.0079	0.6558	0.0969	-6.25788	1.9513E-10
ZnF	ZNF34ZnF	cfZNF34ZnF	ptZNF34ZnF	295.2	790.8	0.3566	4.4672	0.0611	0.0229	0.0055	0.3758	0.0499	-7.02957	1.0358E-12
ZnF	ZNF34ZnF	cfZNF34ZnF	mmulZNF34ZnF	295.5	790.5	0.3455	4.4672	0.0663	0.0238	0.0056	0.3595	0.0481	-6.93239	2.0690E-12
ZnF	ZNF34ZnF	cfZNF34ZnF	btZNF34ZnF	284.5	801.5	0.5504	4.4672	0.0572	0.0345	0.0067	0.6031	0.0852	-6.65317	1.4342E-11
ZnF	ZNF34ZnF	cfZNF34ZnF	hsZNF34ZnF	295.9	790.1	0.3538	4.4672	0.066	0.0243	0.0056	0.368	0.0489	-6.98299	1.4448E-12
ZnF	ZNF517ZnF	cfZNF517ZnF	mmulZNF517ZnF	111	795	1.5103	2.8017	0.0228	0.0805	0.0105	3.5323	4.6028	-0.74993	0.2266
ZnF	ZNF517ZnF	hsZNF517ZnF	mmulZNF517ZnF	86.9	819.1	0.1072	2.8017	0.048	0.0123	0.0039	0.2564	0.0687	-3.54742	0.0002
ZnF	ZNF517ZnF	hsZNF517ZnF	cfZNF517ZnF	114.2	791.8	1.559	2.8017	0.0231	0.0822	0.0107	3.5534	4.667	-0.74377	0.2285
ZnF	ZNF517ZnF	ptZNF517ZnF	mmulZNF517ZnF	85.5	820.5	0.1016	2.8017	0.034	0.0092	0.0034	0.2706	0.0719	-3.63155	0.0001
ZnF	ZNF517ZnF	ptZNF517ZnF	cfZNF517ZnF	112.7	793.3	1.5308	2.8017	0.0223	0.0792	0.0104	3.5438	4.6376	-0.74707	0.2275
ZnF	ZNF517ZnF	ptZNF517ZnF	hsZNF517ZnF	88.7	817.3	0.0237	2.8017	0.0787	0.0037	0.0021	0.0468	0.0237	-1.81147	0.0350
ZnF	ZNF7ZnF	hsZNF7ZnF	btZNF7ZnF	382.4	1087.6	0.9264	3.3098	0.0839	0.0804	0.009	0.9583	0.1437	-6.09731	5.3935E-10
ZnF	ZNF7ZnF	mfasZNF7ZnF	btZNF7ZnF	384.6	1085.4	0.9194	3.3098	0.0838	0.0794	0.009	0.9473	0.1387	-6.24426	2.1291E-10
ZnF	ZNF7ZnF	mfasZNF7ZnF	hsZNF7ZnF	388.3	1081.7	0.0705	3.3098	0.109	0.0074	0.0026	0.0683	0.014	-4.27687	9.4769E-06
ZnF	ZNF7ZnF	mmZNF7ZnF	btZNF7ZnF	371.1	1098.9	1.4704	3.3098	0.1458	0.1977	0.015	1.3559	0.2979	-3.88296	5.1596E-05
ZnF	ZNF7ZnF	mmZNF7ZnF	hsZNF7ZnF	389	1081	0.9567	3.3098	0.2502	0.1779	0.0141	0.7108	0.0883	-5.9596	1.2642E-09
ZnF	ZNF7ZnF	mmZNF7ZnF	mfasZNF7ZnF	390.8	1079.2	0.9416	3.3098	0.2593	0.1784	0.0142	0.688	0.0834	-6.02362	8.5277E-10
ZnF	ZNF7ZnF	ptZNF7ZnF	btZNF7ZnF	382.1	1087.9	0.9492	3.3098	0.0831	0.0818	0.0091	0.9843	0.1512	-5.95813	1.2757E-09
ZnF	ZNF7ZnF	ptZNF7ZnF	hsZNF7ZnF	388.3	1081.7	0.027	3.3098	0.1054	0.0028	0.0016	0.0264	0.0084	-2.7599	0.0029
ZnF	ZNF7ZnF	ptZNF7ZnF	mfasZNF7ZnF	389.1	1080.9	0.0824	3.3098	0.076	0.0065	0.0025	0.0857	0.0159	-4.92068	4.3122E-07
ZnF	ZNF7ZnF	ptZNF7ZnF	mmZNF7ZnF	388.8	1081.2	0.9431	3.3098	0.2547	0.1772	0.0141	0.6957	0.0854	-5.99033	1.0471E-09
ZnF	ZNF7ZnF	rnZNF7ZnF	btZNF7ZnF	371.9	1098.1	1.193	3.3098	0.0947	0.1163	0.011	1.2284	0.2405	-4.61929	1.9253E-06
ZnF	ZNF7ZnF	rnZNF7ZnF	hsZNF7ZnF	387	1083	0.737	3.3098	0.1188	0.0832	0.0092	0.7003	0.0892	-6.88166	2.9581E-12
ZnF	ZNF7ZnF	rnZNF7ZnF	mfasZNF7ZnF	388.5	1081.5	0.7546	3.3098	0.1198	0.0855	0.0093	0.7136	0.0917	-6.81455	4.7279E-12
ZnF	ZNF7ZnF	rnZNF7ZnF	mmZNF7ZnF	381.5	1088.5	0.617	3.3098	0.4209	0.1516	0.0128	0.3601	0.0415	-4.80092	7.8968E-07
ZnF	ZNF7ZnF	rnZNF7ZnF	ptZNF7ZnF	387.2	1082.8	0.7277	3.3098	0.1239	0.0847	0.0093	0.684	0.086	-6.92821	2.1310E-12
ZnF	ZNF7ZnF	mmulZNF7ZnF	btZNF7ZnF	384.3	1085.7	0.92	3.3098	0.0844	0.0799	0.009	0.9472	0.1394	-6.20874	2.6706E-10
ZnF	ZNF7ZnF	mmulZNF7ZnF	hsZNF7ZnF	388.2	1081.8	0.0664	3.3098	0.0901	0.006	0.0024	0.067	0.0139	-4.3245	7.6439E-06
ZnF	ZNF7ZnF	mmulZNF7ZnF	mfasZNF7ZnF	388.8	1081.2	0.0102	3.3098	0.2386	0.0019	0.0013	0.0078	0.0045	-1.2596	0.1039
ZnF	ZNF7ZnF	mmulZNF7ZnF	mmZNF7ZnF	390.5	1079.5	0.9568	3.3098	0.2535	0.179	0.0142	0.7059	0.087	-5.97723	1.1348E-09
ZnF	ZNF7ZnF	mmulZNF7ZnF	ptZNF7ZnF	389	1081	0.0785	3.3098	0.054	0.0046	0.0021	0.0859	0.016	-5.03804	2.3516E-07
ZnF	ZNF7ZnF	mmulZNF7ZnF	rnZNF7ZnF	388.3	1081.7	0.7494	3.3098	0.1218	0.086	0.0094	0.706	0.0899	-6.85916	3.4633E-12
ZnF	ZNF7ZnF	cfZNF7ZnF	btZNF7ZnF	371.6	1098.4	0.6949	3.3098	0.0657	0.0504	0.007	0.7673	0.0989	-7.23065	2.4036E-13
ZnF	ZNF7ZnF	cfZNF7ZnF	hsZNF7ZnF	389.2	1080.8	0.666	3.3098	0.0965	0.0638	0.008	0.6612	0.0804	-7.39384	7.1276E-14
ZnF	ZNF7ZnF	cfZNF7ZnF	mfasZNF7ZnF	391	1079	0.6878	3.3098	0.0932	0.0639	0.008	0.6857	0.085	-7.28311	1.6309E-13
ZnF	ZNF7ZnF	cfZNF7ZnF	mmZNF7ZnF	381.3	1088.7	1.1641	3.3098	0.2149	0.1993	0.0151	0.9271	0.1296	-5.57801	1.2164E-08
ZnF	ZNF7ZnF	cfZNF7ZnF	ptZNF7ZnF	389.1	1080.9	0.6997	3.3098	0.0943	0.0658	0.0081	0.6982	0.0871	-7.22943	2.4247E-13
ZnF	ZNF7ZnF	cfZNF7ZnF	rnZNF7ZnF	381.9	1088.1	0.9696	3.3098	0.1125	0.106	0.0105	0.9421	0.1412	-5.90508	1.7623E-09
ZnF	ZNF7ZnF	cfZNF7ZnF	mmulZNF7ZnF	390.7	1079.3	0.6831	3.3098	0.0949	0.0644	0.008	0.6787	0.0834	-7.33205	1.1335E-13
ZnF	ZNF250ZnF	ptZNF250ZnF	cfZNF250ZnF	171.5	914.5	1.9502	4.3816	0.0126	0.0484	0.0075	3.8584	5.7032	-0.66805	0.2521
ZnF	ZNF250ZnF	rnZNF250ZnF	cfZNF250ZnF	142.3	943.7	39.044	4.3816	0.0004	0.0441	0.007	99	5.7032	-17.3509	0
ZnF	ZNF250ZnF	rnZNF250ZnF	ptZNF250ZnF	240.2	845.8	0.8549	4.3816	0.0086	0.0107	0.0036	1.2506	0.3205	-3.8684	5.4776E-05
ZnF	ZNF250ZnF	mmZNF250ZnF	cfZNF250ZnF	134.9	951.1	37.0151	4.3816	0.0005	0.0465	0.0072	99	0.3205	-308.669	0
ZnF	ZNF250ZnF	mmZNF250ZnF	ptZNF250ZnF	235	851	1.4284	4.3816	0.0063	0.0136	0.004	2.1505	1.9469	-1.09759	0.1362
ZnF	ZNF250ZnF	mmZNF250ZnF	rnZNF250ZnF	211.8	874.2	0.2741	4.3816	0.0025	0.0011	0.0011	0.4637	0.0679	-6.81207	4.8104E-12
ZnF	ZNF250ZnF	hsZNF250ZnF	cfZNF250ZnF	172.8	913.2	1.967	4.3816	0.0126	0.0487	0.0075	3.864	5.7242	-0.66652	0.2525
ZnF	ZNF250ZnF	hsZNF250ZnF	ptZNF250ZnF	260.1	825.9	0.0055	4.3816	0.3144	0.0012	0.0012	0.0039	0.0039	-0.66169	0.2541
ZnF	ZNF250ZnF	hsZNF250ZnF	rnZNF250ZnF	241.2	844.8	0.8774	4.3816	0.0094	0.0119	0.0038	1.2752	0.3365	-3.754	8.7019E-05
ZnF	ZNF250ZnF	hsZNF250ZnF	mmZNF250ZnF	236.1	849.9	1.5343	4.3816	0.0065	0.0149	0.0042	2.2992	2.6279	-0.86925	0.1924
ZnF	ZNF250ZnF	mmulZNF250ZnF	cfZNF250ZnF	170.4	915.6	1.9396	4.3816	0.0129	0.0497	0.0076	3.8535	5.685	-0.66909	0.2517
ZnF	ZNF250ZnF	mmulZNF250ZnF	ptZNF250ZnF	258.8	827.2	0.0747	4.3816	0.025	0.0024	0.0017	0.0968	0.0211	-4.45948	4.1079E-06
ZnF	ZNF250ZnF	mmulZNF250ZnF	rnZNF250ZnF	239.5	846.5	0.842	4.3816	0.0087	0.0107	0.0036	1.2348	0.312	-3.92314	4.3702E-05
ZnF	ZNF250ZnF	mmulZNF250ZnF	mmZNF250ZnF	234.3	851.7	0.76	4.3816	0.0121	0.0136	0.004	1.1246	0.1443	-7.69628	6.9944E-15
ZnF	ZNF250ZnF	mmulZNF250ZnF	hsZNF250ZnF	259.6	826.4	0.0686	4.3816	0.0132	0.0012	0.0012	0.0919	0.0204	-4.43841	4.5314E-06
ZnF	ZNF16ZnF	ptZNF16ZnF	ZNF16ZnF	353.4	1065.6	0.0258	3.209	0.1086	0.0028	0.0016	0.026	0.0087	-2.62268	4.3620E-03
ZnF	ZNF16ZnF	btZNF16ZnF	ZNF16ZnF	306	1113	0.9198	3.209	0.1032	0.1067	0.0104	1.0339	0.1126	-8.19956	0
ZnF	ZNF16ZnF	btZNF16ZnF	ptZNF16	305.2	1113.8	0.9329	3.209	0.1023	0.1077	0.0104	1.0529	0.1155	-8.15057	0
ZnF	ZNF16ZnF	Mmul_ZNF16ZnF	ZNF16ZnF	349.4	1069.6	0.1602	3.209	0.1073	0.0175	0.0041	0.1633	0.025	-5.75512	4.3290E-09
ZnF	ZNF16ZnF	Mmul_ZNF16ZnF	ptZNF16ZnF	349.3	1069.7	0.1729	3.209	0.1189	0.0204	0.0044	0.1717	0.026	-5.73765	4.8000E-09
ZnF	ZNF16ZnF	Mmul_ZNF16ZnF	btZNF16ZnF	295.8	1123.2	0.9401	3.209	0.1086	0.1156	0.0108	1.0646	0.1191	-7.93553	0
ZnF	ZNF16ZnF	cfZNF16ZnF	ZNF16ZnF	359.7	1059.3	0.5629	3.209	0.1411	0.0738	0.0087	0.5229	0.0637	-6.98539	1.4204E-12
ZnF	ZNF16ZnF	cfZNF16ZnF	ptZNF16ZnF	359.9	1059.1	0.5792	3.209	0.1343	0.0733	0.0087	0.5456	0.0666	-7.03185	1.0191E-12
ZnF	ZNF16ZnF	cfZNF16ZnF	btZNF16ZnF	319.3	1099.7	1.0854	3.209	0.0892	0.1097	0.0106	1.2301	0.1411	-7.91816	0
ZnF	ZNF16ZnF	cfZNF16ZnF	Mmul_ZNF16ZnF	356.5	1062.5	0.6176	3.209	0.1373	0.0798	0.0091	0.5815	0.0722	-6.89421	2.7083E-12
ZnF	ZNF252ZnF	ptZNF252ZnF	cfZNF252ZnF	258.5	893.5	0.6101	2.6847	0.2319	0.1166	0.0122	0.5031	0.0717	-5.31414	5.3582E-08
ZnF	ZNF252ZnF	hsZNF252ZnF	cfZNF252ZnF	256.6	895.4	0.6283	2.6847	0.2302	0.12	0.0124	0.5214	0.0749	-5.28718	6.2108E-08
ZnF	ZNF252ZnF	hsZNF252ZnF	ptZNF252ZnF	243.6	908.4	0.0581	2.6847	1.7095	0.0212	0.0049	0.0124	0.0072	1.010427	0.1561
ZnF	ZNF252ZnF	mondomZNF252ZnF	cfZNF252ZnF	250.3	901.7	1.8074	2.6847	0.0419	0.101	0.0112	2.409	3.1905	-0.72339	0.2347
ZnF	ZNF252ZnF	mondomZNF252ZnF	ptZNF252ZnF	233.9	918.1	0.7938	2.6847	0.0981	0.0923	0.0106	0.9409	0.178	-4.75898	9.7285E-07
ZnF	ZNF252ZnF	mondomZNF252ZnF	hsZNF252ZnF	231.2	920.8	0.8085	2.6847	0.0961	0.0933	0.0106	0.9711	0.1903	-4.60558	2.0566E-06
ZnF	ZNF252ZnF	mmulZNF252ZnF	cfZNF252ZnF	396.7	1184.3	0.541	3.7226	0.2307	0.0982	0.0097	0.4256	0.0488	-6.580	2.3478E-11
ZnF	ZNF252ZnF	mmulZNF252ZnF	mondomZNF252ZnF	331.8	1264.2	1.0133	2.5508	0.056	0.075	0.008	1.339	0.3141	-4.023	2.8744E-05
ZnF	ZNF252ZnF	mmulZNF252ZnF	hsZNF252ZnF	393.3	1202.7	0.1751	4.1806	0.7513	0.054	0.0069	0.0718	0.0144	-1.115	0.1325
ZnF	ZNF252ZnF	ptZ252ZnF	mmulZ252ZnF	264.9	908.1	0.1276	3.3447	0.5113	0.035	0.0063	0.0684	0.0171	-1.833	0.0334

Domain	Gene Fragment	Seq1	Seq2	S	N	t	Kappa	Omega	dN	dN_SE	dS	dS_SE	Z Score	P Value
7AA DBD	ZNF251_7AA	hsZNF251_7AA	mmZNF251_7AA	69.8	161.2	0.7234	3.2073	0.1068	0.0684	0.0214	0.6402	0.1589	-3.566	0.0002
7AA DBD	ZNF251_7AA	mmulZNF251_7AA	mmZNF251_7AA	70.1	160.9	0.8488	3.2073	0.0884	0.0685	0.0215	0.7753	0.2071	-3.395	0.0003
7AA DBD	ZNF251_7AA	mmulZNF251_7AA	hsZNF251_7AA	72.1	158.9	0.0401	3.2073	0	0	0	0.0428	0.0249	-1.719	0.0428
7AA DBD	ZNF251_7AA	rnZNF251_7AA	mmZNF251_7AA	66.8	164.2	0.1314	3.2073	0	0	0	0.1514	0.053	-2.857	0.0021
7AA DBD	ZNF251_7AA	rnZNF251_7AA	hsZNF251_7AA	69.8	161.2	0.6942	3.2073	0.1125	0.0684	0.0214	0.6077	0.152	-3.513	0.0002
7AA DBD	ZNF251_7AA	rnZNF251_7AA	mmulZNF251_7AA	70.1	160.9	0.8147	3.2073	0.0928	0.0685	0.0215	0.7381	0.1973	-3.374	0.0004
7AA DBD	ZNF251_7AA	ptZNF251_7AA	mmZNF251_7AA	69.8	161.2	0.6873	3.2073	0.114	0.0684	0.0214	0.6	0.1468	-3.583	0.0002
7AA DBD	ZNF251_7AA	ptZNF251_7AA	hsZNF251_7AA	71.9	159.1	0.0131	3.2073	0	0	0	0.014	0.0141	-0.993	0.1604
7AA DBD	ZNF251_7AA	ptZNF251_7AA	mmulZNF251_7AA	71.9	159.1	0.054	3.2073	0	0	0	0.0578	0.0292	-1.979	0.0239
7AA DBD	ZNF251_7AA	ptZNF251_7AA	rnZNF251_7AA	69.7	161.3	0.6602	3.2073	0.1197	0.0683	0.0214	0.5711	0.1413	-3.518	0.0002
7AA DBD	ZNF34_7AA	mmulZNF34_7AA	ptZNF34_7AA	58.5	193.5	0.1062	3.4424	0	0	0	0.1524	0.056	-2.721	0.0033
7AA DBD	ZNF34_7AA	btZNF34_7AA	ptZNF34_7AA	53.4	198.6	0.6057	3.4424	0.0559	0.0441	0.0153	0.7893	0.2935	-2.536	0.0056
7AA DBD	ZNF34_7AA	btZNF34_7AA	mmulZNF34_7AA	51.6	200.4	0.6478	3.4424	0.0494	0.0437	0.0151	0.8853	0.34	-2.473	0.0067
7AA DBD	ZNF34_7AA	hsZNF34_7AA	ptZNF34_7AA	59.6	192.4	0	3.4424	99	0	0	0	0	#DIV/0!	#DIV/0!
7AA DBD	ZNF34_7AA	hsZNF34_7AA	mmulZNF34_7AA	58.5	193.5	0.1062	3.4424	0	0	0	0.1524	0.056	-2.721	0.0033
7AA DBD	ZNF34_7AA	hsZNF34_7AA	btZNF34_7AA	53.4	198.6	0.6057	3.4424	0.0559	0.0441	0.0153	0.7893	0.2935	-2.536	0.0056
7AA DBD	ZNF34_7AA	cfZNF34_7AA	ptZNF34_7AA	63.2	188.8	0.3232	3.4424	0.0421	0.0161	0.0093	0.3818	0.1066	-3.418	0.0003
7AA DBD	ZNF34_7AA	cfZNF34_7AA	mmulZNF34_7AA	62.2	189.8	0.3767	3.4424	0.0348	0.016	0.0093	0.4601	0.1297	-3.415	0.0003
7AA DBD	ZNF34_7AA	cfZNF34_7AA	btZNF34_7AA	57.9	194.1	0.5529	3.4424	0.041	0.0289	0.0124	0.7051	0.2307	-2.927	0.0017
7AA DBD	ZNF34_7AA	cfZNF34_7AA	hsZNF34_7AA	63.2	188.8	0.3232	3.4424	0.0421	0.0161	0.0093	0.3818	0.1066	-3.418	0.0003
7AA DBD	ZNF517_7AA	cfZNF517_7AA	mmulZNF517_7AA	40.3	169.7	0.7996	5.8278	0.0323	0.0395	0.0157	1.2239	0.7895	-1.500	0.0668
7AA DBD	ZNF517_7AA	hsZNF517_7AA	mmulZNF517_7AA	32.8	177.2	0.0822	5.8278	0	0	0	0.1753	0.084	-2.087	0.0184
7AA DBD	ZNF517_7AA	hsZNF517_7AA	cfZNF517_7AA	40.3	169.7	0.8114	5.8278	0.0318	0.0396	0.0157	1.2422	0.7299	-1.647	0.0498
7AA DBD	ZNF517_7AA	ptZNF517_7AA	mmulZNF517_7AA	32.8	177.2	0.0822	5.8278	0	0	0	0.1753	0.084	-2.087	0.0184
7AA DBD	ZNF517_7AA	ptZNF517_7AA	cfZNF517_7AA	40.3	169.7	0.8114	5.8278	0.0318	0.0396	0.0157	1.2422	0.7299	-1.647	0.0498
7AA DBD	ZNF517_7AA	ptZNF517_7AA	hsZNF517_7AA	32.9	177.1	0	5.8278	99	0	0	0	0	#DIV/0!	#DIV/0!
7AA DBD	ZNF7_7AA	hsZNF7_7AA	btZNF7_7AA	82.1	211.9	0.5905	4.3504	0.0373	0.024	0.0108	0.6432	0.1579	-3.912	4.5705E-05
7AA DBD	ZNF7_7AA	mfasZNF7_7AA	btZNF7_7AA	82	212	0.5663	4.3504	0.0305	0.0191	0.0096	0.6276	0.1477	-4.111	1.9684E-05
7AA DBD	ZNF7_7AA	mfasZNF7_7AA	hsZNF7_7AA	88.1	205.9	0.0877	4.3504	0	0	0	0.0975	0.0354	-2.754	0.0029
7AA DBD	ZNF7_7AA	mmZNF7_7AA	btZNF7_7AA	75.5	218.5	0.7794	4.3504	0.2747	0.1548	0.029	0.5636	0.1364	-2.932	0.0017
7AA DBD	ZNF7_7AA	mmZNF7_7AA	hsZNF7_7AA	82.8	211.2	0.6605	4.3504	0.4675	0.1667	0.0308	0.3566	0.0876	-2.045	0.0204
7AA DBD	ZNF7_7AA	mmZNF7_7AA	mfasZNF7_7AA	83	211	0.697	4.3504	0.4184	0.1669	0.0308	0.3988	0.0961	-2.298	0.0108
7AA DBD	ZNF7_7AA	ptZNF7_7AA	btZNF7_7AA	81.2	212.8	0.5641	4.3504	0.0386	0.0239	0.0107	0.6183	0.1504	-3.942	4.0375E-05
7AA DBD	ZNF7_7AA	ptZNF7_7AA	hsZNF7_7AA	87.6	206.4	0.0103	4.3504	0	0	0	0.0115	0.0115	-1.000	0.1587
7AA DBD	ZNF7_7AA	ptZNF7_7AA	mfasZNF7_7AA	87.6	206.4	0.0759	4.3504	0	0	0	0.0849	0.0328	-2.588	0.0048
7AA DBD	ZNF7_7AA	ptZNF7_7AA	mmZNF7_7AA	81.9	212.1	0.6427	4.3504	0.4892	0.1659	0.0306	0.3392	0.0841	-1.936	0.0264
7AA DBD	ZNF7_7AA	rnZNF7_7AA	btZNF7_7AA	81.2	212.8	0.816	4.3504	0.0346	0.0312	0.0123	0.9033	0.263	-3.312	0.00046258
7AA DBD	ZNF7_7AA	rnZNF7_7AA	hsZNF7_7AA	87.7	206.3	0.4961	4.3504	0.0334	0.0172	0.0092	0.5137	0.1211	-4.088	2.1743E-05
7AA DBD	ZNF7_7AA	rnZNF7_7AA	mfasZNF7_7AA	87.9	206.1	0.5937	4.3504	0.0277	0.0172	0.0092	0.6215	0.1581	-3.816	0.0001
7AA DBD	ZNF7_7AA	rnZNF7_7AA	mmZNF7_7AA	81.7	212.3	0.6061	4.3504	0.4713	0.154	0.0293	0.3268	0.0844	-1.934	0.0265
7AA DBD	ZNF7_7AA	rnZNF7_7AA	ptZNF7_7AA	87	207	0.526	4.3504	0.031	0.0171	0.0092	0.5515	0.1325	-4.024	2.8667E-05
7AA DBD	ZNF7_7AA	mmulZNF7_7AA	btZNF7_7AA	81.4	212.6	0.5771	4.3504	0.0296	0.0191	0.0096	0.6451	0.1567	-3.987	3.3398E-05
7AA DBD	ZNF7_7AA	mmulZNF7_7AA	hsZNF7_7AA	87.8	206.2	0.0884	4.3504	0	0	0	0.0987	0.0361	-2.734	0.0031
7AA DBD	ZNF7_7AA	mmulZNF7_7AA	mfasZNF7_7AA	87.7	206.3	0.0103	4.3504	0	0	0	0.0115	0.0115	-1.000	0.1587
7AA DBD	ZNF7_7AA	mmulZNF7_7AA	mmZNF7_7AA	82.5	211.5	0.7019	4.3504	0.4089	0.1664	0.0307	0.4071	0.0999	-2.303	0.0106
7AA DBD	ZNF7_7AA	mmulZNF7_7AA	ptZNF7_7AA	87.2	206.8	0.0764	4.3504	0	0	0	0.0859	0.0334	-2.572	0.0051
7AA DBD	ZNF7_7AA	mmulZNF7_7AA	rnZNF7_7AA	87.5	206.5	0.5854	4.3504	0.0279	0.0172	0.0092	0.615	0.153	-3.900	4.8068E-05
7AA DBD	ZNF7_7AA	cfZNF7_7AA	btZNF7_7AA	76.6	217.4	0.4827	4.3504	0.0156	0.0093	0.0066	0.5916	0.1394	-4.173	1.5063E-05
7AA DBD	ZNF7_7AA	cfZNF7_7AA	hsZNF7_7AA	84.1	209.9	0.442	4.3504	0.0248	0.012	0.0076	0.4851	0.1135	-4.159	1.5984E-05
7AA DBD	ZNF7_7AA	cfZNF7_7AA	mfasZNF7_7AA	83.9	210.1	0.5306	4.3504	0.0204	0.012	0.0076	0.5897	0.1465	-3.938	4.1073E-05
7AA DBD	ZNF7_7AA	cfZNF7_7AA	mmZNF7_7AA	78.2	215.8	0.8032	4.3504	0.2736	0.1569	0.0294	0.5735	0.1401	-2.910	0.0018
7AA DBD	ZNF7_7AA	cfZNF7_7AA	ptZNF7_7AA	83.4	210.6	0.4688	4.3504	0.023	0.012	0.0076	0.5204	0.1232	-4.119	1.9043E-05
7AA DBD	ZNF7_7AA	cfZNF7_7AA	rnZNF7_7AA	83.7	210.3	0.548	4.3504	0.0371	0.0218	0.0103	0.5869	0.1443	-3.906	4.6878E-05
7AA DBD	ZNF7_7AA	cfZNF7_7AA	mmulZNF7_7AA	83.4	210.6	0.5243	4.3504	0.0204	0.012	0.0076	0.5857	0.1426	-4.017	2.9417E-05
7AA DBD	ZNF250_7AA	ptZNF250_7AA	cfZNF250_7AA	50.8	222.2	1.6885	5.4357	0.0062	0.0182	0.0092	2.9457	3.1443	-0.931	0.1759
7AA DBD	ZNF250_7AA	rnZNF250_7AA	cfZNF250_7AA	30.9	242.1	33.6233	5.4357	0.0001	0.0125	0.0072	99	3.1443	-31.481	0
7AA DBD	ZNF250_7AA	rnZNF250_7AA	ptZNF250_7AA	66.2	206.8	0.802	5.4357	0	0	0	1.1017	0.4542	-2.426	0.0076
7AA DBD	ZNF250_7AA	mmZNF250_7AA	cfZNF250_7AA	29.1	243.9	31.6864	5.4357	0.0002	0.0166	0.0084	99	0.4542	-217.892	0
7AA DBD	ZNF250_7AA	mmZNF250_7AA	ptZNF250_7AA	64.5	208.5	0.841	5.4357	0.0084	0.0097	0.0068	1.1556	0.286	-4.006	3.0942E-05
7AA DBD	ZNF250_7AA	mmZNF250_7AA	rnZNF250_7AA	47.5	225.5	0.426	5.4357	0.0056	0.0044	0.0045	0.7943	0.318	-2.484	0.0065
7AA DBD	ZNF250_7AA	hsZNF250_7AA	cfZNF250_7AA	50.8	222.2	1.6885	5.4357	0.0062	0.0182	0.0092	2.9457	3.1443	-0.931	0.1759
7AA DBD	ZNF250_7AA	hsZNF250_7AA	ptZNF250_7AA	78.7	194.3	0	5.4357	99	0	0	0	0	#DIV/0!	#DIV/0!
7AA DBD	ZNF250_7AA	hsZNF250_7AA	rnZNF250_7AA	66.2	206.8	0.802	5.4357	0	0	0	1.1017	0.4542	-2.426	0.0076
7AA DBD	ZNF250_7AA	hsZNF250_7AA	mmZNF250_7AA	64.5	208.5	0.841	5.4357	0.0084	0.0097	0.0068	1.1556	0.286	-4.006	3.0942E-05
7AA DBD	ZNF250_7AA	mmulZNF250_7AA	cfZNF250_7AA	47.9	225.1	1.5707	5.4357	0.0062	0.018	0.009	2.9013	3.0559	-0.944	0.1727
7AA DBD	ZNF250_7AA	mmulZNF250_7AA	ptZNF250_7AA	77.3	195.7	0.0831	5.4357	0	0	0	0.0978	0.0382	-2.560	0.0052
7AA DBD	ZNF250_7AA	mmulZNF250_7AA	rnZNF250_7AA	64	209	0.6358	5.4357	0	0	0	0.9041	0.3057	-2.957	0.0016
7AA DBD	ZNF250_7AA	mmulZNF250_7AA	mmZNF250_7AA	62.2	210.8	0.8297	5.4357	0.0081	0.0096	0.0068	1.1813	0.3007	-3.896	0.0000
7AA DBD	ZNF250_7AA	mmulZNF250_7AA	hsZNF250_7AA	77.3	195.7	0.0831	5.4357	0	0	0	0.0978	0.0382	-2.560	0.0052
7AA DBD	ZNF16_7AA	ptZNF16_7AA	ZNF16_7AA	81.1	230.9	0.0194	3.6784	0.3487	0.0043	0.0043	0.0125	0.0125	-0.620	0.2675
7AA DBD	ZNF16_7AA	btZNF16_7AA	ZNF16_7AA	60.6	251.4	1.0241	3.6784	0.0488	0.0712	0.0176	1.461	0.4315	-3.218	6.4503E-04
7AA DBD	ZNF16_7AA	btZNF16_7AA	ptZNF16	61.8	250.2	1.0031	3.6784	0.0552	0.0761	0.0183	1.3793	0.3861	-3.372	3.7379E-04
7AA DBD	ZNF16_7AA	Mmul_ZNF16_7AA	ZNF16_7AA	76.8	235.2	0.1605	3.6784	0.1045	0.0172	0.0086	0.1647	0.0534	-2.727	0.0032
7AA DBD	ZNF16_7AA	Mmul_ZNF16_7AA	ptZNF16	77.8	234.2	0.1718	3.6784	0.1321	0.0217	0.0098	0.1643	0.0539	-2.603	0.0046
7AA DBD	ZNF16_7AA	Mmul_ZNF16_7AA	btZNF16_7AA	56.1	255.9	0.8831	3.6784	0.0706	0.0874	0.0195	1.2386	0.3402	-3.378	3.6462E-04
7AA DBD	ZNF16_7AA	cfZNF16_7AA	ZNF16_7AA	86.1	225.9	0.31	3.6784	0.0801	0.0248	0.0106	0.3095	0.0803	-3.515	2.1991E-04
7AA DBD	ZNF16_7AA	cfZNF16_7AA	ptZNF16	87.1	224.9	0.2978	3.6784	0.0583	0.018	0.009	0.3091	0.0778	-3.717	1.0086E-04
7AA DBD	ZNF16_7AA	cfZNF16_7AA	btZNF16_7AA	68.4	243.6	1.0714	3.6784	0.054	0.0738	0.0183	1.3664	0.3612	-3.574	1.7576E-04
7AA DBD	ZNF16_7AA	cfZNF16_7AA	Mmul_ZNF16_7AA	83.4	228.6	0.4213	3.6784	0.105	0.0428	0.014	0.4079	0.1019	-3.550	1.9292E-04
7AA DBD	ZNF252_7AA	hsZNF252_7AA	cfZNF252_7AA	67.7	178.3	0.8394	3.4055	0.297	0.1695	0.0338	0.5706	0.1685	-2.334	0.0098
7AA DBD	ZNF252_7AA	mondomZNF252_7AA	cfZNF252_7AA	65.6	180.4	1.1731	3.4055	0.102	0.1168	0.0271	1.1456	0.2801	-3.656	1.2814E-04
7AA DBD	ZNF252_7AA	mondomZNF252_7AA	hsZNF252_7AA	63	183	1.0805	3.4055	0.1042	0.1125	0.0266	1.0797	0.4778	-2.021	0.0216
7AA DBD	ZNF252_7AA	mmulZNF252_7AA	cfZNF252_7AA	68.4	177.6	0.9041	3.4055	0.1947	0.1401	0.0303	0.7196	0.2297	-2.501	0.0062
7AA DBD	ZNF252_7AA	mmulZNF252_7AA	hsZNF252_7AA	67.9	178.1	0.1387	3.4055	1.0211	0.0465	0.0166	0.0455	0.0265	0.032	0.4872
7AA DBD	ZNF252_7AA	mmulZNF252_7AA	mondomZNF252_7AA	63.3	182.7	0.9368	3.4055	0.0679	0.0689	0.0202	1.0142	0.406	-2.325	0.0100
7AA DBD	ZNF252_7AA	ptZNF252_7AA	cfZNF252_7AA	67.9	178.1	0.8388	3.4055	0.2986	0.1697	0.0338	0.5681	0.1676	-2.330	0.0099
7AA DBD	ZNF252_7AA	ptZNF252_7AA	hsZNF252_7AA	68.3	177.7	0.1126	3.4055	1.3546	0.0405	0.0154	0.0299	0.0212	0.405	0.3429
7AA DBD	ZNF252_7AA	ptZNF252_7AA	mondomZNF252_7AA	64.1	181.9	1.0076	3.4055	0.0922	0.0942	0.0242	1.0218	0.4218	-2.196	0.0141
7AA DBD	ZNF252_7AA	ptZNF252_7AA	mmulZNF252_7AA	68.6	177.4	0.152	3.4055	0.5296	0.0406	0.0155	0.0767	0.0348	-0.948	0.1717

calculated with PAML version 3.14 (Yang 1997, Comput. Appl. Biosci 13:555-556; Yang and Nielsen, 2000, Mol. Biol. Evol. 17:32-43)

Legend

**krab_A, _B**	input sequences: KRAB-A or KRAB-B, respectively
**link**	input is non-conserved sequence between KRAB-B and zinc finger array
**ZnF**	input is whole sequence encoding the C2H2 zinc fingers
**7AA DBD**	based on nucleotide sequence specifying amino acids that are considered important for DNA binding specificity of a C2H2 zinc finger (amino acids -2 to 6 with respect ot start of the alpha helix; see also Supplement 8); however, the codon encoding the conserved hydrophobic amino acid, in the vast majority leucine, on position 4 was removed; this sequences are arteficially linked together for all C2H2 zinc fingers of a particular gene
**S**	Number of synonymous sites in a sequence
**N**	Number of non-synonymous sites in a sequence
**t**	Time (distance), measured by the expected number of nucleotide substitutons per codon (= dS x 3S/(S+N) +dN x 3N((S+N))
**Kappa**	ratio of transition/transversion (mutation) rates
**Omega**	ratio of non-synonymous/synonymous rates (= dN/dS)
**dN**	Number of non-synonymous substitutions per non-synonymous site
**dN_SE**	Standard error of the mean of the dN calculation
**dS**	Number of synonymous substitutions per synonymous site
**dS_SE**	Standard error of the mean of the dS calculation
**Z score**	$Z = (dN-dS)/ \mathrm{sqrt}(dN\_SE^2 + dS\_SE^2)$
**p-value**	p = 1-normsdist (abs(Z score))

Note: "99" or "#DIV/0" values reflect non-productive calculations, e.g. if two sequences do not show differences
