# Supplementary material for: The ancient mammalian KRAB zinc finger gene cluster on human chromosome 8q24.3 illustrates principles of C2H2 zinc finger evolution associated with unique expression profiles in human tissues
Source: BMC Genomics. 2010 Mar 26;11:206. doi: 10.1186/1471-2164-11-206 (PMC2865497; doi:10.1186/1471-2164-11-206)
Supplement: Additional file 6 — Zinc finger DNA-binding region comparison. "Choo & Klug plot" for the C2H2 zinc finger regions of the human 8q24.3 ZNF genes and all their orthologs. Zinc finger region alignment of the proteins in each ortholog group based on individual zinc fingers represented by residues -1, 3 and 6 that are thought to be essential in determining DNA binding specificity. [file 1471-2164-11-206-S6.PDF]

Additional file 6: Zinc finger DNA-binding region comparison

"Choo & Klug plot" for the C2H2 zinc finger regions of the human 8q24.3 ZNF genes and all their mammalian orthologs. For each individual zinc finger, residues -1, 3 and 6 with respect to the start of the α-helix (that were proposed to be especially important for DNA-binding specificity; see text) were extracted and consecutively written down from the N- to the C-terminal end of the whole array. For species designation see Additional file 1 or 2.

Upper case letters: Residues from canonical, complete C2H2 zinc fingers
Lower case letters: Residues from non-canonical, degenerate C2H2 zinc fingers
- separator of individual C2H2 zinc fingers
* indicates Stop codon within sequence
xxx further sequence (non C2H2 or very degenerate sequence with less than 2 out of the 4 C2H2 conserved residues at the right positions) triple x equals length of a typical C2H2 domain
gap no sequence in between but inserted to keep alignment of orthologs

ZNF251

```
>hsZNF251
YDR-HNL-LHL-RTQ-QQQ-RSQ-QSL-FHE-RTQ-QQL-RTQ-xxx-xx-pre-GTQ-xxx
>ptZNF251
YDR-HNL-LHL-RTQ-QQQ-RSQ-QSL-FHE-RTQ-QQL-RTQ-xxx-xx-pre-GTQ-qqp
>mmulZNF251
YDR-HNL-LHL-RTQ-QQQ-RSQ-QSL-FHE-RTQ-QQL-RTQ-xxx-xx-pqe-GAR-qqp
>mmZNF251/Zfp251
YDR-HNL-LYL-RSQ-QQQ-RSQ-QSL-FHE-RTQ-QQL-RAQ-yss-xx-tqd-sst
>rnZNF251
YDR-HNL-LYL-RSQ-QQQ-RSQ-QSL-FHE-RTQ-QQL-RAQ-xxx-xx-sqd-sst
```

ZNF34

```
>hsZNF34
QYN-xxx-YNK-QEN-RNK-ASQ-DIR-QNQ-QKE-QHQ-NRH-QYQ-HNQ
>ptZNF34
QYN-xxx-YNK-QEN-RNK-ASY-DIR-QNQ-QKE-QHQ-NRH-QYQ-HNQ
>mmulZNF34
QYN-xxx-YNK-QEN-RNK-ASY-DIR-QNQ-QKE-QHQ-NRH-QYQ-HNQ
>cfZNF34
QYN-xxx-YNK-QEN-QNK-ASY-DIR-QNQ-QKE-QHQ-NRH-QYQ-HNQ
>btZNF34
QYN-xxx-YNK-QES-QNK-ASY-DIR-QNQ-QKE-QHQ-NRH-QYQ-HNQ
```

ZNF517

```
>hsZNF517
ylr-QIR-QQA-RRQ-RTE-RSK-xxx-HLL-RNL-RTE-RTQ
>ptZNF517
ylr-QIR-QQA-RRQ-RTE-RSK-xxx-HLL-RNL-RTE-RTQ
>mmulZNF517
ylr-QIR-QQA-RRQ-RTE-RSK-xxx-HLL-RNL-RTE-RTQ
>cfZNF517
ylr-QIR-QQA-RRQ-RTE-RSK-xxx-HLL-RNL-RTE-RAQ
```

ZNF7

>hsZNF7
adl-dqh-LQQ-LKQ-QSH-QQR-QTQ-dsa-WRQ-CRR-QHQ-QSY-MQI-QTQ-RYE-xxx-WHI-RRQ
>ptZNF7
adl-dqh-LQQ-LKQ-QSH-QQR-QTQ-dsa-WRQ-CRR-QHQ-QSY-MQI-QTQ-RYE-xxx-WHI-RRQ
>mmulZNF7
adl-dqh-LQQ-LKQ-QSH-QQR-QTQ-dsa-WRQ-CRR-QHQ-QSY-MQI-QTQ-RYE-xxx-WHI-RRQ
>mfasZNF7
adl-dqh-LQQ-LKQ-QSH-QQR-QTQ-dsa-WRQ-CRR-QHQ-QSY-MQI-QTQ-RYE-xxx-WHI-RRQ
>cfZNF7
sdl-dqp-LQQ-LKQ-QSH-QQR-QTQ-dsa-WRQ-CRR-QHQ-QSY-MQI-QTQ-RYE-xxx-WHI-RRQ
>btZNF7
sdl-dqp-LQQ-LKQ-QSH-QQR-QTQ-esa-WRQ-CRR-QHQ-QSY-MQI-QTQ-RYE-xxx-WHI-RRQ
>mmZNF7/Zfp7
WRQ-SRR-LVQ-LKQ-QSH-QQR-QTQ-esa-WRQ-CRR-QHQ-QSY-MQS-QTQ-RYE-etn-WYI-QKQ
>rnZNF7
pnp-dqh-LQQ-LKQ-QSH-QQR-QTQ-xxx-WRQ-CRR-QHQ-QSY-MQS-QTQ-RYE-xxx-WHI-RRQ

ZNF250

>hsZNF250
RHQ-QVK-VDQ-QHQ-HVS-VTQ-DVQ-HTN-QHQ-ASQ-LTV-QVQ-QHQ
>ptZNF250
RHQ-QVK-VDQ-QHQ-HVS-VTQ-DVQ-HTN-QHQ-ASQ-LTV-QVQ-QHQ
>mmulZNF250
RHQ-QVK-VDQ-QHQ-HVS-VTQ-DVQ-HTN-QHQ-ASQ-LTV-QVQ-QHQ
>cfZNF250
RHQ-QVK-VDQ-QHQ-HVS-VTQ-DVQ-HTN-QHQ-ASQ-LTV-QVQ-QHQ
>mmZNF250/Zfp647
RHQ-QVK-VDQ-QHQ-HVS-VTQ-DVQ-HTN-QHQ-ASQ-LTV-QVQ-QHQ
>rnZNF250
RHQ-QVK-VDQ-QHQ-HVS-VTQ-DVQ-HTN-QHQ-ASQ-LTV-QVQ-QHQ

ZNF16

>hsZNF16
xxx-gdq-qvn-GDR-QSK-RNQ-RNK-QHK-RNK-QSQ-YVK-HAQ-RNL-QTQ-RNH-QHQ-QVQ-QKK
>ptZNF16
xxx-gdq-qvn-GDR-QSK-RNQ-RNK-QHK-RNK-QSQ-YVK-HAQ-RNL-QTQ-RNH-QHQ-QVQ-QKK
>mmulZNF16
xxx-GDQ-qvs-GDR-QSK-RNQ-RNK-QHK-vlp-QSQ-YVK-HAQ-RNL-QTQ-RNH-QHQ-QVQ-QKK
>cfZNF16
hdg-GDQ-QFN-VNR-QSK-RNQ-RNK-QHK-RNK-QSQ-YVK-HAQ-RNL-QTQ-RNH-QHQ-QVQ-qkp
>btZNF16
xxx-GGQ-TGQ   -QSK-RNQ-RNK-QHK-RNK-QSQ-YVK-HAQ-RNL-QTQ-RNH-QHQ-QVQ-QHQ-QVK

ZNF252

>hsZNF252
qaq-wik- VYQ-TSQ-HNH-RNQ-TFQ-LTV-QQQ-ASQ-qhq*-QNH-LSQ-QHQ-QTK-QHH-
VSQ-QQQ-ASQ-*qhq-*ts-l*tf
>ptZNF252
*qaq-qik-*vyq-TSQ-HNH-RHQ-TSQ-ltv-qqq-ASQ-qhq*-QNH-VSQ-QHQ
>mmulZNF252
qaq-RIK- AYQ-TSR-hnh-RHQ-TSQ-LTV-QQQ-asq-QHQ –QNH-VSQ-QHQ-QTK-QHH-
ISQ-QQQ-QHR
>cfZNF252
PAQ-hvq- ayq-asq-HNH-RHQ-ASQ-LTV-QQQ-ASQ-QHQ -QNH-VSQ-QHQ-QTK-QHR-
VSQ-QQQ-ASQ- QHR-QTS-aaq
>mondomZNF252
lkk-KDN-qie-QTQ-RIK- AYQ-TSQ-HNH-RHQ-ASQ-LTV-QQQ-ASQ-QHQ -QNH-VSQ-QHQ-QTK-QHR-
VSQ-QQQ-ASQ- QHR-QTS
