# Supplementary material for: The ancient mammalian KRAB zinc finger gene cluster on human chromosome 8q24.3 illustrates principles of C2H2 zinc finger evolution associated with unique expression profiles in human tissues
Source: BMC Genomics. 2010 Mar 26;11:206. doi: 10.1186/1471-2164-11-206 (PMC2865497; doi:10.1186/1471-2164-11-206)
Supplement: Additional file 9 — Tissue expression distances inferred from ZNF gene expression. Euclidian distance between 27 human tissues based on the expression analysis of 17 human ZNF genes (7 genes from 8q24.3, 10 from other loci). [file 1471-2164-11-206-S9.PDF]

Additional file 9: Euclidian distance between 27 human tissues based on the expression analysis of 17 human ZNF genes (7 genes from 8q24.3, 10 from other loci)

Euclidean distance	a Bone marrow	b Thymus	c Spleen	d Trachea	e Lung	f Kidney	g Bladder	h Prostate	i Testis	j Uterus	k Placenta	l Mammary gland	m Adrenal gland	n Thyroid	o Salivary gland	p Stomach	q Pancreas	r Colon	s Small intestine	t Liver	u Fetal liver	v Heart	w Skeletal muscle	x Brain	y Cerebellum	z Spinal cord	$ Fetal brain
a Bone marrow	0.0	114.1	41.2	34.8	75.7	128.2	49.6	160.7	631.6	60.9	75.6	51.7	49.1	249.6	64.2	44.2	52.9	36.8	34.4	87.3	82.0	87.2	80.3	69.9	155.2	52.7	999.6
b Thymus	114.1	0.0	134.7	134.1	179.8	141.4	122.1	142.5	571.1	148.9	179.0	136.2	144.6	203.1	169.8	121.0	159.7	131.1	126.7	191.8	185.9	191.6	182.2	172.5	152.3	118.7	922.0
c Spleen	41.2	134.7	0.0	25.5	45.9	128.0	50.7	187.7	658.7	32.2	45.4	38.1	35.6	270.8	37.3	58.8	33.0	26.4	29.5	58.7	52.3	58.4	58.1	46.4	154.6	60.6	1024.3
d Trachea	34.8	134.1	25.5	0.0	51.9	119.0	35.3	172.6	643.3	30.4	51.1	28.1	26.0	258.3	41.0	46.0	29.4	21.6	24.0	64.2	58.5	64.3	60.7	49.6	149.7	49.3	1013.2
e Lung	75.7	179.8	45.9	51.9	0.0	153.6	80.3	218.1	687.0	46.7	7.3	60.5	53.4	303.1	14.4	88.0	28.2	56.1	62.1	14.2	8.3	14.1	41.8	35.2	183.1	91.9	1060.4
f Kidney	128.2	141.4	128.0	119.0	153.6	0.0	91.9	143.1	597.3	112.6	153.2	103.3	112.8	177.4	144.5	96.8	134.9	124.2	118.0	165.3	159.8	166.0	140.6	141.4	103.4	95.2	949.7
g Bladder	49.6	122.1	50.7	35.3	80.3	91.9	0.0	146.1	622.4	42.3	79.3	32.3	33.4	227.9	69.7	31.4	57.9	46.2	41.7	93.2	87.2	93.3	76.8	67.5	121.5	24.1	983.5
h Prostate	160.7	142.5	187.7	172.6	218.1	143.1	146.1	0.0	497.6	186.8	217.4	165.5	170.2	112.3	206.5	136.2	192.0	183.0	176.9	229.4	224.6	229.8	206.8	201.7	162.9	140.5	865.7
i Testis	631.6	571.1	658.7	643.3	687.0	597.3	622.4	497.6	0.0	659.3	686.6	634.9	648.1	454.3	678.1	618.2	662.9	654.1	650.7	695.8	691.9	696.2	682.6	678.9	620.3	623.2	696.3
j Uterus	60.9	148.9	32.2	30.4	46.7	112.6	42.3	186.8	659.3	0.0	45.2	31.9	27.8	265.8	39.4	60.1	35.9	36.7	38.4	59.4	53.0	59.5	52.8	43.6	140.3	56.9	1022.3
k Placenta	75.6	179.0	45.4	51.1	7.3	153.2	79.3	217.4	686.6	45.2	0.0	59.7	52.2	302.7	14.7	87.2	28.1	55.6	61.4	16.1	9.9	16.0	42.5	35.6	181.3	91.1	1059.4
l Mammary gland	51.7	136.2	38.1	28.1	60.5	103.3	32.3	165.5	634.9	31.9	59.7	0.0	29.6	245.8	51.2	43.6	39.7	45.3	44.9	72.1	66.6	72.6	61.3	55.5	145.2	51.4	1010.2
m Adrenal gland	49.1	144.6	35.6	26.0	53.4	112.8	33.4	170.2	648.1	27.8	52.2	29.6	0.0	254.0	42.0	40.3	32.3	39.0	36.6	66.0	60.0	66.1	48.7	39.5	140.7	42.6	1011.0
n Thyroid	249.6	203.1	270.8	258.3	303.1	177.4	227.9	112.3	454.3	265.8	302.7	245.8	254.0	0.0	291.7	220.3	277.9	266.5	259.2	315.1	309.9	315.7	287.6	285.9	200.9	222.4	800.9
o Salivary gland	64.2	169.8	37.3	41.0	14.4	144.5	69.7	206.5	678.1	39.4	14.7	51.2	42.0	291.7	0.0	75.3	15.9	46.2	51.2	26.2	20.7	26.5	37.6	30.3	174.6	80.8	1049.5
p Stomach	44.2	121.0	58.8	46.0	88.0	96.8	31.4	136.2	618.2	60.1	87.2	43.6	40.3	220.3	75.3	0.0	62.1	56.4	49.4	100.3	94.8	100.7	77.9	72.8	133.9	31.6	981.3
q Pancreas	52.9	159.7	33.0	29.4	28.2	134.9	57.9	192.0	662.9	35.9	28.1	39.7	32.3	277.9	15.9	62.1	0.0	40.0	43.8	39.3	34.3	39.8	40.6	33.6	168.4	70.1	1037.3
r Colon	36.8	131.1	26.4	21.6	56.1	124.2	46.2	183.0	654.1	36.7	55.6	45.3	39.0	266.5	46.2	56.4	40.0	0.0	10.4	68.4	62.5	68.2	67.5	55.4	149.6	54.7	1014.9
s Small intestine	34.4	126.7	29.5	24.0	62.1	118.0	41.7	176.9	650.7	38.4	61.4	44.9	36.6	259.2	51.2	49.4	43.8	10.4	0.0	74.7	68.8	74.7	68.0	56.6	143.1	47.1	1007.8
t Liver	87.3	191.8	58.7	64.2	14.2	165.3	93.2	229.4	695.8	59.4	16.1	72.1	66.0	315.1	26.2	100.3	39.3	68.4	74.7	0.0	7.0	2.3	48.3	44.7	195.4	105.0	1072.8
u Fetal liver	82.0	185.9	52.3	58.5	8.3	159.8	87.2	224.6	691.9	53.0	9.9	66.6	60.0	309.9	20.7	94.8	34.3	62.5	68.8	7.0	0.0	7.0	45.2	40.4	189.3	99.0	1067.3
v Heart	87.2	191.6	58.4	64.3	14.1	166.0	93.3	229.8	696.2	59.5	16.0	72.6	66.1	315.7	26.5	100.7	39.8	68.2	74.7	2.3	7.0	0.0	48.6	44.3	195.2	104.9	1072.7
w Skeletal muscle	80.3	182.2	58.1	60.7	41.8	140.6	76.8	206.8	682.6	52.8	42.5	61.3	48.7	287.6	37.6	77.9	40.6	67.5	68.0	48.3	45.2	48.6	0.0	21.3	172.4	82.5	1046.3
x Brain	69.9	172.5	46.4	49.6	35.2	141.4	67.5	201.7	678.9	43.6	35.6	55.5	39.5	285.9	30.3	72.8	33.6	55.4	56.6	44.7	40.4	44.3	21.3	0.0	164.7	72.3	1037.9
y Cerebellum	155.2	152.3	154.6	149.7	183.1	103.4	121.5	162.9	620.3	140.3	181.3	145.2	140.7	200.9	174.6	133.9	168.4	149.6	143.1	195.4	189.3	195.2	172.4	164.7	0.0	112.9	912.0
z Spinal cord	52.7	118.7	60.6	49.3	91.9	95.2	24.1	140.5	623.2	56.9	91.1	51.4	42.6	222.4	80.8	31.6	70.1	54.7	47.1	105.0	99.0	104.9	82.5	72.3	112.9	0.0	970.4
$ Fetal brain	999.6	922.0	1024.3	1013.2	1060.4	949.7	983.5	865.7	696.3	1022.3	1059.4	1010.2	1011.0	800.9	1049.5	981.3	1037.3	1014.9	1007.8	1072.8	1067.3	1072.7	1046.3	1037.9	912.0	970.4	0.0

Note: increasing values denote increasing distances

distance range

0-20

20-100

100-400

>400
