# Supplementary material for: The ancient mammalian KRAB zinc finger gene cluster on human chromosome 8q24.3 illustrates principles of C2H2 zinc finger evolution associated with unique expression profiles in human tissues
Source: BMC Genomics. 2010 Mar 26;11:206. doi: 10.1186/1471-2164-11-206 (PMC2865497; doi:10.1186/1471-2164-11-206)
Supplement: Additional file 10 — Gene expression similarities based on tissue expression profiles. Pairwise distances between all genes based on their tissue expression profiles. Included are the seven 8q24.3 ZNF genes, ten ZNF genes from other loci, TRIM28 and twelve non-ZNF genes. Calculation: Distance = 1 - abs (Pearson correlation coefficient). [file 1471-2164-11-206-S10.PDF]

**Additional file 10:**

**Gene expression similarities based on the expression profiles in 27 tissues and calculated with:**
**Distance = 1 – abs (Pearson correlation coefficient)**

| | ZNF251 | ZNF34 | ZNF517 | ZNF7 | ZNF250 | ZNF16 | ZNF252 | ZNF10 | ZNF136 | ZNF25 | ZNF439 | ZNF101 | ZNF23 | ZNF20 | ZNF248 | ZNF224 | ZNF228 | TIF1b | TBP | Foxp3 | USP38 | Senp6 | Raf | Sox7 | BLZF1 | FGF18 | ITGB1BP2 | GAPDH | IL8 | KDM6A |
|---|---|---|---|---|---|---|---|---|---|---|---|---|---|---|---|---|---|---|---|---|---|---|---|---|---|---|---|---|---|---|
| ZNF251 | 1 | 0.91 | 0.82 | 0.73 | 0.94 | 0.88 | 0.88 | 0.8 | 0.92 | 0.85 | 0.92 | 0.55 | 0.93 | 0.8 | 0.93 | 0.89 | 0.91 | 0.67 | 0.57 | 0.078 | 0.77 | 0.86 | 0.89 | 0.098 | 0.92 | 0.18 | 0.022 | 0.14 | -0.035 | 0.91 |
| ZNF34 | 0.91 | 1 | 0.77 | 0.7 | 0.96 | 0.86 | 0.73 | 0.75 | 0.82 | 0.8 | 0.86 | 0.47 | 0.87 | 0.69 | 0.88 | 0.82 | 0.87 | 0.67 | 0.57 | 0.072 | 0.65 | 0.81 | 0.78 | 0.016 | 0.81 | 0.17 | 0.017 | 0.13 | -0.034 | 0.81 |
| ZNF517 | 0.82 | 0.77 | 1 | 0.79 | 0.74 | 0.87 | 0.7 | 0.72 | 0.75 | 0.45 | 0.62 | 0.49 | 0.66 | 0.72 | 0.62 | 0.68 | 0.57 | 0.75 | 0.66 | 0.15 | 0.54 | 0.46 | 0.67 | 0.12 | 0.73 | 0.21 | -0.019 | 0.1 | -0.15 | 0.62 |
| ZNF7 | 0.73 | 0.7 | 0.79 | 1 | 0.58 | 0.92 | 0.57 | 0.91 | 0.7 | 0.46 | 0.69 | 0.57 | 0.71 | 0.92 | 0.65 | 0.65 | 0.56 | 0.93 | 0.88 | 0.2 | 0.56 | 0.48 | 0.74 | 0.096 | 0.81 | 0.24 | 0.097 | 0.096 | -0.055 | 0.6 |
| ZNF250 | 0.94 | 0.96 | 0.74 | 0.58 | 1 | 0.77 | 0.84 | 0.65 | 0.88 | 0.86 | 0.87 | 0.45 | 0.87 | 0.61 | 0.91 | 0.88 | 0.92 | 0.52 | 0.4 | 0.045 | 0.72 | 0.86 | 0.81 | 0.074 | 0.8 | 0.1 | 0.014 | 0.15 | -0.022 | 0.87 |
| ZNF16 | 0.88 | 0.86 | 0.87 | 0.92 | 0.77 | 1 | 0.65 | 0.92 | 0.76 | 0.6 | 0.78 | 0.53 | 0.81 | 0.9 | 0.76 | 0.7 | 0.7 | 0.9 | 0.85 | 0.12 | 0.59 | 0.63 | 0.78 | 0.07 | 0.84 | 0.26 | 0.064 | 0.095 | -0.099 | 0.71 |
| ZNF252 | 0.88 | 0.73 | 0.7 | 0.57 | 0.84 | 0.65 | 1 | 0.62 | 0.96 | 0.82 | 0.82 | 0.4 | 0.84 | 0.65 | 0.87 | 0.89 | 0.87 | 0.39 | 0.3 | -0.042 | 0.87 | 0.82 | 0.83 | 0.12 | 0.88 | 0.063 | 0.095 | 0.22 | 0.026 | 0.85 |
| ZNF10 | 0.8 | 0.75 | 0.72 | 0.91 | 0.65 | 0.92 | 0.62 | 1 | 0.76 | 0.65 | 0.81 | 0.52 | 0.85 | 0.98 | 0.77 | 0.65 | 0.72 | 0.89 | 0.91 | 0.062 | 0.62 | 0.67 | 0.76 | 0.0015 | 0.89 | 0.36 | 0.04 | 0.067 | -0.048 | 0.7 |
| ZNF136 | 0.92 | 0.82 | 0.75 | 0.7 | 0.88 | 0.76 | 0.96 | 0.76 | 1 | 0.84 | 0.9 | 0.53 | 0.91 | 0.78 | 0.93 | 0.94 | 0.91 | 0.56 | 0.47 | 0.066 | 0.83 | 0.85 | 0.86 | 0.13 | 0.94 | 0.14 | 0.031 | 0.093 | 0.013 | 0.89 |
| ZNF25 | 0.85 | 0.8 | 0.45 | 0.46 | 0.86 | 0.6 | 0.82 | 0.65 | 0.84 | 1 | 0.92 | 0.39 | 0.92 | 0.61 | 0.95 | 0.82 | 0.98 | 0.37 | 0.31 | -0.037 | 0.82 | 0.99 | 0.81 | 0.031 | 0.84 | 0.13 | 0.14 | 0.22 | 0.029 | 0.89 |
| ZNF439 | 0.92 | 0.86 | 0.62 | 0.69 | 0.87 | 0.78 | 0.82 | 0.81 | 0.9 | 0.92 | 1 | 0.63 | 0.98 | 0.81 | 0.97 | 0.91 | 0.96 | 0.64 | 0.54 | 0.17 | 0.78 | 0.95 | 0.88 | -0.0085 | 0.93 | 0.18 | 0.018 | 0.094 | 0.068 | 0.95 |
| ZNF101 | 0.55 | 0.47 | 0.49 | 0.57 | 0.45 | 0.53 | 0.4 | 0.52 | 0.53 | 0.39 | 0.63 | 1 | 0.53 | 0.59 | 0.46 | 0.68 | 0.44 | 0.63 | 0.48 | 0.81 | 0.44 | 0.43 | 0.53 | -0.042 | 0.56 | 0.052 | -0.056 | -0.0085 | 0.063 | 0.64 |
| ZNF23 | 0.93 | 0.87 | 0.66 | 0.71 | 0.87 | 0.81 | 0.84 | 0.85 | 0.91 | 0.92 | 0.98 | 0.53 | 1 | 0.84 | 0.96 | 0.87 | 0.95 | 0.65 | 0.6 | 0.021 | 0.8 | 0.93 | 0.86 | -0.007 | 0.95 | 0.21 | -0.013 | 0.11 | 0.088 | 0.93 |
| ZNF20 | 0.8 | 0.69 | 0.72 | 0.92 | 0.61 | 0.9 | 0.65 | 0.98 | 0.78 | 0.61 | 0.81 | 0.59 | 0.84 | 1 | 0.75 | 0.69 | 0.69 | 0.88 | 0.89 | 0.13 | 0.63 | 0.64 | 0.76 | 0.033 | 0.9 | 0.34 | 0.02 | 0.033 | -0.013 | 0.71 |
| ZNF248 | 0.93 | 0.88 | 0.62 | 0.65 | 0.91 | 0.76 | 0.87 | 0.77 | 0.93 | 0.95 | 0.97 | 0.46 | 0.96 | 0.75 | 1 | 0.9 | 0.99 | 0.54 | 0.45 | 0.0018 | 0.79 | 0.96 | 0.87 | 0.084 | 0.92 | 0.16 | 0.065 | 0.11 | 0.016 | 0.93 |
| ZNF224 | 0.89 | 0.82 | 0.68 | 0.65 | 0.88 | 0.7 | 0.89 | 0.65 | 0.94 | 0.82 | 0.91 | 0.68 | 0.87 | 0.69 | 0.9 | 1 | 0.88 | 0.53 | 0.38 | 0.28 | 0.77 | 0.84 | 0.84 | 0.11 | 0.88 | 0.063 | -0.018 | 0.056 | 0.12 | 0.92 |
| ZNF228 | 0.91 | 0.87 | 0.57 | 0.56 | 0.92 | 0.7 | 0.87 | 0.72 | 0.91 | 0.98 | 0.96 | 0.44 | 0.95 | 0.69 | 0.99 | 0.88 | 1 | 0.47 | 0.39 | -0.02 | 0.79 | 0.99 | 0.84 | 0.061 | 0.89 | 0.15 | 0.049 | 0.12 | 0.014 | 0.93 |
| TRIM28 | 0.67 | 0.67 | 0.75 | 0.93 | 0.52 | 0.9 | 0.39 | 0.89 | 0.56 | 0.37 | 0.64 | 0.63 | 0.65 | 0.88 | 0.54 | 0.53 | 0.47 | 1 | 0.93 | 0.32 | 0.42 | 0.4 | 0.64 | 0.037 | 0.7 | 0.3 | 0.037 | 0.062 | -0.095 | 0.55 |
| TBP | 0.57 | 0.57 | 0.66 | 0.88 | 0.4 | 0.85 | 0.3 | 0.91 | 0.47 | 0.31 | 0.54 | 0.48 | 0.6 | 0.89 | 0.45 | 0.38 | 0.39 | 0.93 | 1 | 0.14 | 0.35 | 0.33 | 0.51 | -0.04 | 0.65 | 0.38 | -0.0054 | 0.026 | -0.063 | 0.41 |
| Foxp3 | 0.078 | 0.072 | 0.15 | 0.2 | 0.045 | 0.12 | -0.042 | 0.062 | 0.066 | -0.037 | 0.17 | 0.81 | 0.021 | 0.13 | 0.0018 | 0.28 | -0.02 | 0.32 | 0.14 | 1 | 0.011 | -0.019 | 0.11 | -0.077 | 0.065 | -0.045 | -0.016 | -0.043 | -0.053 | 0.18 |
| USP38 | 0.77 | 0.65 | 0.54 | 0.56 | 0.72 | 0.59 | 0.87 | 0.62 | 0.83 | 0.82 | 0.78 | 0.44 | 0.8 | 0.63 | 0.79 | 0.77 | 0.79 | 0.42 | 0.35 | 0.011 | 1 | 0.79 | 0.85 | 0.075 | 0.82 | 0.062 | 0.41 | 0.57 | 0.063 | 0.8 |
| Senp6 | 0.86 | 0.81 | 0.46 | 0.48 | 0.86 | 0.63 | 0.82 | 0.67 | 0.85 | 0.99 | 0.95 | 0.43 | 0.93 | 0.64 | 0.96 | 0.84 | 0.99 | 0.4 | 0.33 | -0.019 | 0.79 | 1 | 0.81 | 0.033 | 0.85 | 0.14 | 0.068 | 0.14 | 0.055 | 0.91 |
| Raf | 0.89 | 0.78 | 0.67 | 0.74 | 0.81 | 0.78 | 0.83 | 0.76 | 0.86 | 0.81 | 0.88 | 0.53 | 0.86 | 0.76 | 0.87 | 0.84 | 0.84 | 0.64 | 0.51 | 0.11 | 0.85 | 0.81 | 1 | 0.16 | 0.88 | 0.1 | 0.23 | 0.35 | 0.05 | 0.85 |
| Sox7 | 0.098 | 0.016 | 0.12 | 0.096 | 0.074 | 0.07 | 0.12 | 0.0015 | 0.13 | 0.031 | -0.0085 | -0.042 | -0.007 | 0.033 | 0.084 | 0.11 | 0.061 | 0.037 | -0.04 | -0.077 | 0.075 | 0.033 | 0.16 | 1 | 0.055 | -0.029 | 0.12 | -0.015 | -0.14 | 0.15 |
| BLZF1 | 0.92 | 0.81 | 0.73 | 0.81 | 0.8 | 0.84 | 0.88 | 0.89 | 0.94 | 0.84 | 0.93 | 0.56 | 0.95 | 0.9 | 0.92 | 0.88 | 0.89 | 0.7 | 0.65 | 0.065 | 0.82 | 0.85 | 0.88 | 0.055 | 1 | 0.23 | 0.064 | 0.1 | 0.042 | 0.88 |
| FGF18 | 0.18 | 0.17 | 0.21 | 0.24 | 0.1 | 0.26 | 0.063 | 0.36 | 0.14 | 0.13 | 0.18 | 0.052 | 0.21 | 0.34 | 0.16 | 0.063 | 0.15 | 0.3 | 0.38 | -0.045 | 0.062 | 0.14 | 0.1 | -0.029 | 0.23 | 1 | 0.2 | 0.077 | -0.086 | 0.087 |
| ITGB1BP2 | 0.022 | 0.017 | -0.019 | 0.097 | 0.014 | 0.064 | 0.095 | 0.04 | 0.031 | 0.14 | 0.018 | -0.056 | -0.013 | 0.02 | 0.065 | -0.018 | 0.049 | 0.037 | -0.0054 | -0.016 | 0.41 | 0.068 | 0.23 | 0.12 | 0.064 | 0.2 | 1 | 0.77 | -0.1 | 0.0077 |
| GAPDH | 0.14 | 0.13 | 0.1 | 0.096 | 0.15 | 0.095 | 0.22 | 0.067 | 0.093 | 0.22 | 0.094 | -0.0085 | 0.11 | 0.033 | 0.11 | 0.056 | 0.12 | 0.062 | 0.026 | -0.043 | 0.57 | 0.14 | 0.35 | -0.015 | 0.1 | 0.077 | 0.77 | 1 | -0.0006 | 0.12 |
| IL8 | -0.035 | -0.034 | -0.15 | -0.055 | -0.022 | -0.099 | 0.026 | -0.048 | 0.013 | 0.029 | 0.068 | 0.063 | 0.088 | -0.013 | 0.016 | 0.12 | 0.014 | -0.095 | -0.063 | -0.053 | 0.063 | 0.055 | 0.05 | -0.14 | 0.042 | -0.086 | -0.1 | -0.0006 | 1 | 0.088 |
| KDM6A | 0.91 | 0.81 | 0.62 | 0.6 | 0.87 | 0.71 | 0.85 | 0.7 | 0.89 | 0.89 | 0.95 | 0.64 | 0.93 | 0.71 | 0.93 | 0.92 | 0.93 | 0.55 | 0.41 | 0.18 | 0.8 | 0.91 | 0.85 | 0.15 | 0.88 | 0.087 | 0.0077 | 0.12 | 0.088 | 1 |

- 8q24.3 locus ZNF genes
- non-8q24.3 KRAB-ZNF genes
- TRIM28 (KRAB-interacting protein)
- non - (C2H2 ZNF) genes

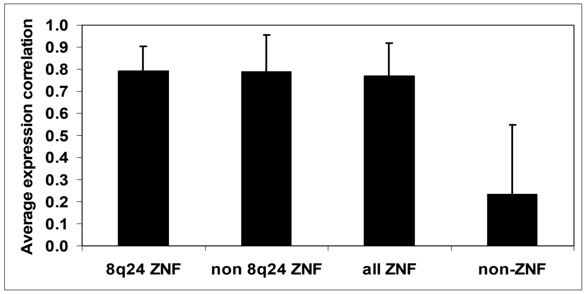


Group-wise calculation of average expression similarities (based on distance measure in table above). Error bars describe standard deviations. Note, that TRIM28 is not included in any group
