# Supplementary material for: The ancient mammalian KRAB zinc finger gene cluster on human chromosome 8q24.3 illustrates principles of C2H2 zinc finger evolution associated with unique expression profiles in human tissues
Source: BMC Genomics. 2010 Mar 26;11:206. doi: 10.1186/1471-2164-11-206 (PMC2865497; doi:10.1186/1471-2164-11-206)
Supplement: Additional file 11 — Proximal promoter sequences of the seven human 8q24.3 locus ZNF genes. Sequences of the proximal promoter regions of the seven human 8q24.3 ZNF genes extracted by Genomatix Gene2Promoter. [file 1471-2164-11-206-S11.RTF]

Proximal promoters of the seven human 8q24.3 locus ZNF genes predicted by Genomatix Gene2Promoter

>ZNF7_GXP_1506101
GGACAAGGCCCGAGCCAGGCGTCTCCGCGAGGCGGGGTCCATAGCGCGCCCAGCAGGCGCCTCCTGTCGCAGGGACGCGGGGGAGCGGCGCCTGAACAGCCTCGGTGGGAACCAGCGCCCGAAGCTGGTGATCCGGGGTGCGCAGGGCCCAACCGGGCGATCCCCTGGATCTCCGCCAGCCTCGCCCCGACTCCGCTCCTCCCACCCGCCAGCGAGTCCGCAACTCCCGGCCCCGGCCCGCGTCCCCAGCTCCAGGCCCCGGCCCCGCCCCGCCCCTGCTCAGGCACCGCCCACTCCGGGCTCTGGGCCCTCCTCTCCCTCGCGGCTCCGCCCCCTGCCCGCCTCTGACTGTACCCCGCCCCCAGGCGCCAGCCCCGCCTATCTGGGCTTCTGGTCCCGCCCAGGTTCTTGTCCCTCGGGCCCCAGCCTCGCCCCTCCTCGGCTCTGACTCCACCCCTTCTCCGGAGCCTGGGAGGCGGGGCTGAGAACCACAGAGATGGCCGGGCCTCCGAGCGGCCCAGGGCGGCCGGAAGTTTGCGGGGCGGGGCGGACGCGGGTGGCCAAGGCCCGTTTCCGGCGGCGTCGCGCGTTTGCGAGCCTCGGGTGGTCCTCAGGGAGGGTGAGTCGGCGCGGCGGGCGCGGACTCGGGTTGCCCTCGGTCCGAGTGATCCCTGGTCGCTTCCTTAGCCCTCC

>ZNF16_GXP_51450
TCGGCCTCCCAAGTGCTGGAATTACAGGCGTGGGCCACCGCACCCAGCCTTATTTGTATTCTTTATGTCCAAGTGTACAATTCATTTCCGTATTAGAAACGATCCATGCACAATCTCACGAAAACATGTTATCTACTGAAGGAATCTGAATTCTTCCATTTATCTATCTAATCACTTACTGATTGAATGATTCAGTTCTTCCAAAAAACGTATCCTTAGGTTTTTATCAAACTACCGTCTGTTCCGATTCTGGGGGCCTGTATATTAAAATCTCCCACTACACGAGTATATCTCATTATCTCTGCATTTCTATTAGTGTCCCCTTTTCTTCAGTTGCCGCCTTGTTCAGTGCACGGGTCAACTCTGAAAAAAAGGGGAACAGAAGGGTCTCTGGCCTCAGGGCTAGTGGGACCAGGTCTGGGGCTGAACCACAGAGTCGCCCCGGCCTCCCGCCATCTAGAGAGGCTACGCTGCCCCAGATAGAACGAGCGGCGGGCCGGAATTCGGGGCGGGACTTCCGGGGGTCAGCCGGCGTTGGCTGAGACGTCTTCGTGCCACGGTGCTGCCTCCTTTCCAAGCGCGACCCGTTGAGGTTAGTTCCGCGAAGCTCCACGCCTGGGACCCTGCGTCT

>ZNF34_GXP_51569
GGCCATGAGGGAAAGTTTGTTCCAGAAGCAGCTCAGGCACCCGAAAAACCCTTTCTCCAGAGTGGACACTGGGCCCCATTTACAGGAGTTAAGGATGAGGAGCAATAGGTTAAACCCAAGACAGGCTTCTTTGGCCAGTAAGCATCCTTTCTTGGCCCTAAGGAAGGGTGCGGTTCCCGTTTCTCTGTAAATCGAAGGGCCTAGTTCTTGTTTGCGGCGCCTTTCGTGCATCATTAGGGAGAGAATTTCTCAGAAAAAGGGAAAGACTAATATGTTTTCGCCTCTGACCATCGCCCCAGTTCTGAGGCCTCTGGGCCGTGGCTTCGGACTGCCACCCGGTGGCAGCATTCCCGCTTTACGGCTTTGCCATTCGCGGGTCCCCGGAGCCTTACTGCAAGCCAGGGAGGTGGGCAGCCGACACGCGGCGTCCTAGCTTCCCAGAGCGGCCCGGACGGCGCCTCGGACCCAGACAGTCGGCTGACGTCACTTCCGGATCGGTTCCGATTGGCGGGGAGGGAGGGCCGAACGGGCGAGGTTGGGCTGCCGTGCTGCTCGGCGGCGCTGAGGTGAGTGTGAGCCCGGCGGGGGCGGGCGCGCGGGGCGCAGACCCTCCCGGCGCAGACCCTCCCCTGCGCGTTGCCC

>ZNF250_GXP_51306
CGCACTGCAGCGGCGCAGGGACCAGCCAGGCGGCTGAGGGCAAGGTCGTTCCCAGCAGCGGGAACCGCAAGGGCAAAAGGCTCGGAGGGGGGAACGAGCTCGAGTTCCAGGACGGCCGAAGCCAGCGCAAGTCGAGGAGGGACTGCGGGGCCGGCAGGGGTGTCCTCCACAGGGCGACGGCGGCAGGAGCTCGGGAACCGGGACTGCGAGCGGGTAAGGCTCGGCGATGGGCTGTAACCTCCGCGCCCGGAAGTGCGGCCGGGGAGCCGGCCGGGCCCATTGCCCAGAGGTGTTCGGCCCCTCTAGGGATCGCGGAGGTCTGGCTCTCGGTGGCTCCTGAGAGCTCCCTGCAGTCGGTCACCGAGAAAGGCTTCGGGCGGGCAGGCGGGCCAAATGACGTAAACATGCTTGCCCTCCAGGATTGGCGAACTCGGCTGTGGGGGCGGGAACTGAGGGGCAGGAGCCCCCAGGGATTGGCGGCTGCGCAGACGGGGCGGGGCAGCCCTTTGTCTGAAGGTGCTGCGGGATGCCGTTCCTTCGCGCGTGAGGCTGCGGCTCTGACGGTGAGTGGGGGGCGCGTGTCGTCGCCGGCCAGGACCCTCTCCCATCACGGACACCGGAGCCGGTGCGTCCTCCACAC

>ZNF251_GXP_926503
CATCAGAGTCGTGTTTTTAGGTTTTGTGGCTGGCTTTGGGGGTCTGGCCTCTGGGACTCGCCTTGGGTAAAAGGGATTCACTCTGTGAGGAGCCTCGCGGGAGAAAAGAACTGAGACTGGAGGGCTGGGAAGGTCAGAGGGAAACTGCCTCCCAGGCCTTCATTTTGGGGCGTTGTGTTCTGAGCCCCGATAATTGTAACATCTTCATAAGCGACTTTCTGGTGGTTATGGTGAATCGGGACACACGATGTATGGTGACCGACGGGGGAGCCGGGAGCCCAGCGGGGTTAGGGTTCAGGTGAGGGTTTAGGGTCGGGGCGAGCGCGGCGCGGCCCGGATGCGCAGCGTCACCGCGCCAGACCCTCCGCTCAGCTGCCGTGGGCTCTCCCCCTCCCCGACCATAGACGGCGCAGGCCTCCAGTCATCCCAGAGCGGCCCCGGAAGAATCCGGGCGGAACCGCGGCTGGCTTCCGGTTCCCGGCGTTCTCGGAGGCGTACTGAGGCCCCGCAGGGGGCGGGGAAGCCTGCGTGTGCGCAGCTCGGCCCGGCCCGCCCTCTTCATCCTAGCCCGCCCCCTCCCCGGCTCTGGACCCGGTTCTGTGGGAGGGTCCGTTCCGGGCTCGGTGCGACTGCGCAGCTCCTCGGCGCTTCCTCGGTGGCTTCCCCGGGTCGAGCAAACAGGTGGGGCAGTGTCGGACCAGGAAGGAGCGCCAAGCG


>ZNF252_GXP_51420
AGCTCCACCGCAATAGGTCTGAAACCTCTCTGCACCTCGGCTTCCTCATTTAAGCGATGGCAGCAGCCTTCTAGAGGGGGCGATGAGGTCAAAAGACTACAGGTGGGAAGTGCTAGGTGCACAGCGGGCGCGAGCTGGATTCTGAGCTTGATCACTACTTGGAGGTGGGGGAAAAGTCGCCCGAGAGAGGGGGACAGTGCACCGCGAGACGTCCTAGGTGAGGAAGGGCCGGGTCCAGAGCACGGAGAACAACCCCCGGTTGAGGAGCGTCCGCAGGAACGCTCGGTGTCGGGGTCCCTTCCTTCGCAGCCGCACGCCGCCGGCGGCACCTCTGCACCTGCGCCCTCTCGCCCGCAGACTCCGCCAAGGGCTGGAAGGGACGTAACCGGGAAGGAGCAGGACTGCCGGAGCATTGAGTACTATCAGCCGCCTAGAGCGGACCCACGAAACCTGGAATTCAATTGGTTGGTGAGCTGAAGGGCAGGGCGGAAGTTCAGATTAGGCATTTCCGGCCCAGGGGGCTTGGCTCCCCGCGGCACGGGATTTAGCGTTCGCGCTCCTTCCCTTCCCGTGGTCGAGCCGAGTGAGTCCCGAGCTAGGGCGCCTGGTGCGGAGGTGCCGGAGTGGCGCTGGGGCGGGCAGGGGCGGTG

>ZNF517_GXP_137603
GAATCGCTTGAACCTGAGAGGCGGAGGTTGCAGTGAGCCCAGATCACACCATTGCACTCCAGCCTGGGGAACAAGAGCGAGACTCAGGCTCAAAAAAAAAAAAAAAAGTCATACTAAACAATGTCATAAATTTATATGAGACCGACCCTGGCCGGACGCCCCCGCTCCGCCCTTCCTGCGGGTCCGGGAGTCGATCCCCCACCGTGCTCCCCAACCCCCGCAGGCCACCCGGGGGCAGAGAGTGGGCGCAAGGGGTGAACGTTCGCTGCCTCCGATCCCGGGGGAAACGGAGCGCCGAGAACGTCGACGCCCTTCCGCGCCCGTCGGGCCTGGGACTGATACCCGGTCTGGCCCGGAACCCCCCAGGCGCGCCCCTAGCTGGGGCCCTGGCGACCGCCGCGCCGCAGGGGCTAGAAGCCGTCGAGACGCGAGCGCCATCGAGGCGCGCGGTCCTCCAGGCCTCCCAGAGCGGCGCAGCGGCCGTTTCCGGGGGCGGGGAAGGAACCGGAGCCTGAGAGCCGGGCGCCGTGCGCTCCTCCCCGCGCTGTCTCGGCGGCCCAGGGTGAGTCGGCCGCGGCCGCGGGGCGGGGACTGGGAGGGGCGCGGTCCTCCGAGCCCGGCCTGGTGG
