# Supplementary figures and images for: The ancient mammalian KRAB zinc finger gene cluster on human chromosome 8q24.3 illustrates principles of C2H2 zinc finger evolution associated with unique expression profiles in human tissues
Source: BMC Genomics. 2010 Mar 26;11:206. doi: 10.1186/1471-2164-11-206 (PMC2865497; doi:10.1186/1471-2164-11-206)

# UCSC Genome Browser Human (hg18)

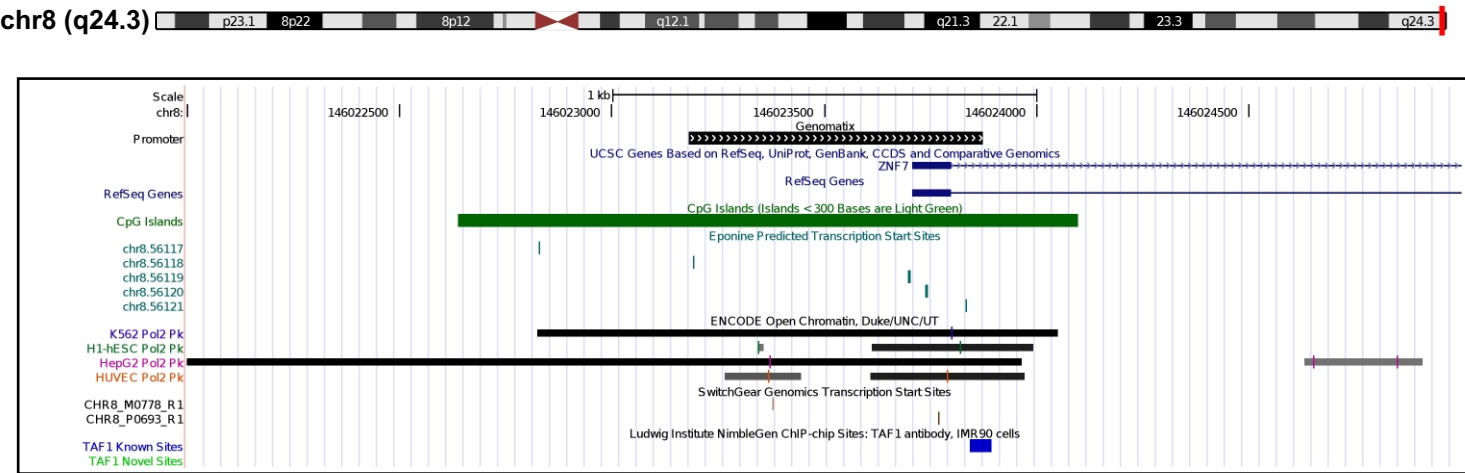

**ZNF7 (+)**

**chr8:146,022,000-146,025,000**

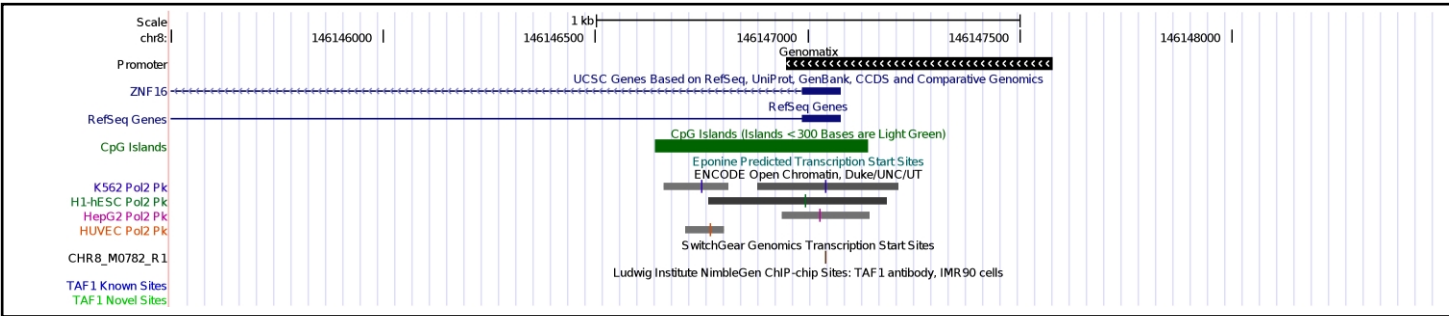

**ZNF16 (-)**

**chr8:146,145,500-146,148,500**

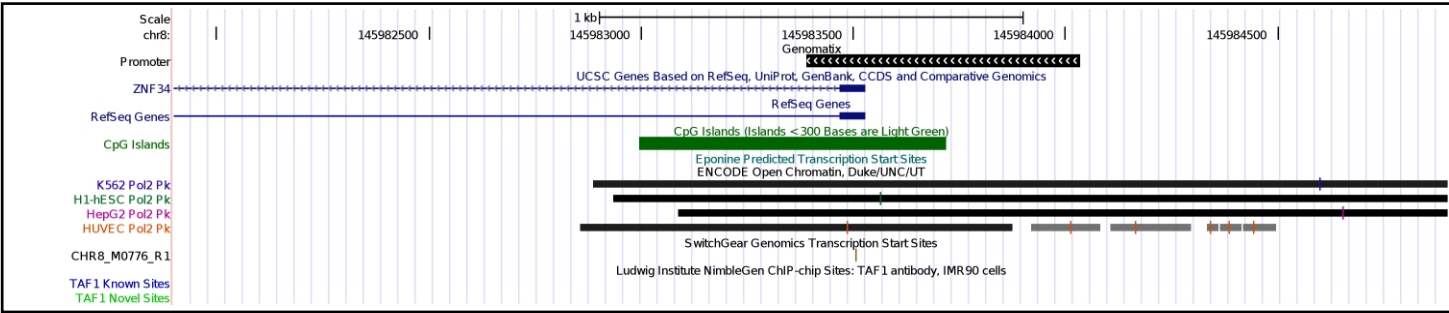

**ZNF34 (-)**

**chr8:145,981,700-145,984,700**

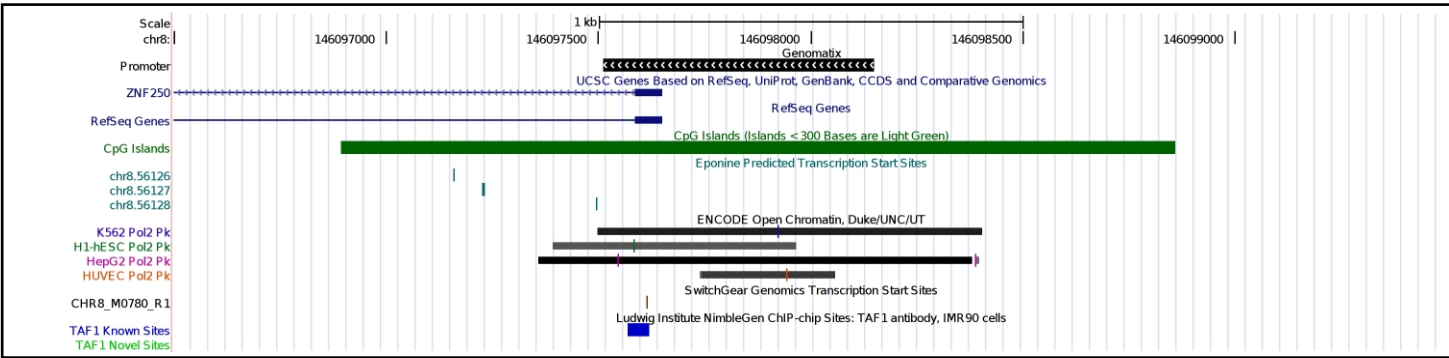

**ZNF250 (-)**

**chr8:146,096,500-146,099,500**

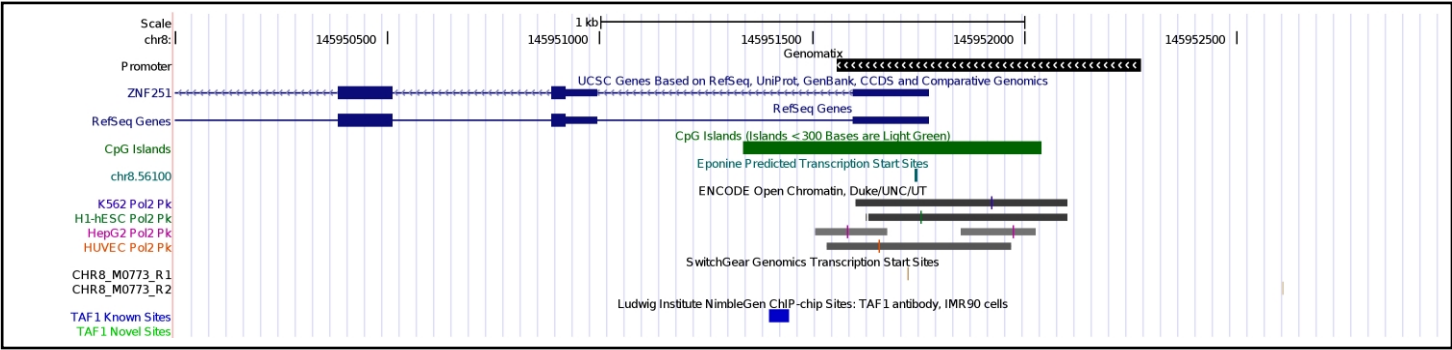

**ZNF251 (-)** **chr8:145,950,000-145,953,000**

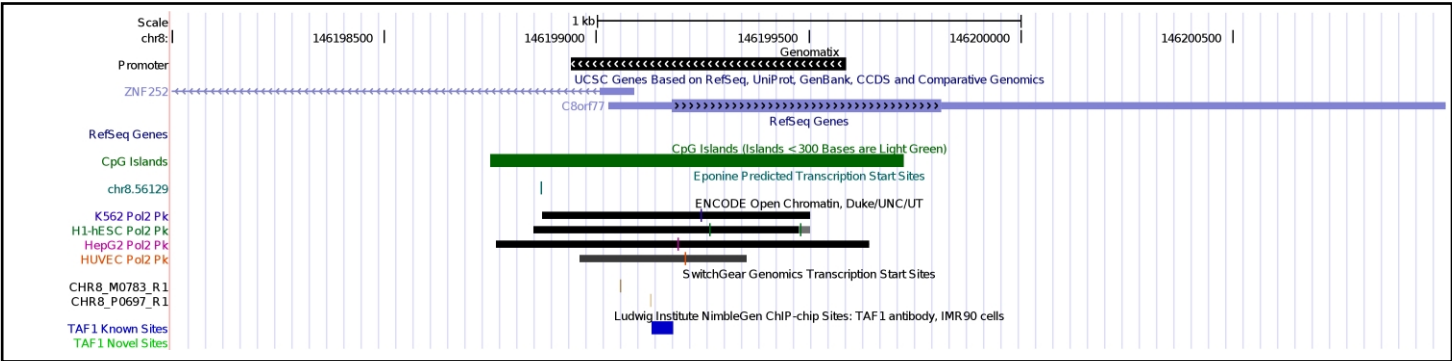

**ZNF252 (-)** **chr8:146,198,000-146,201,000**

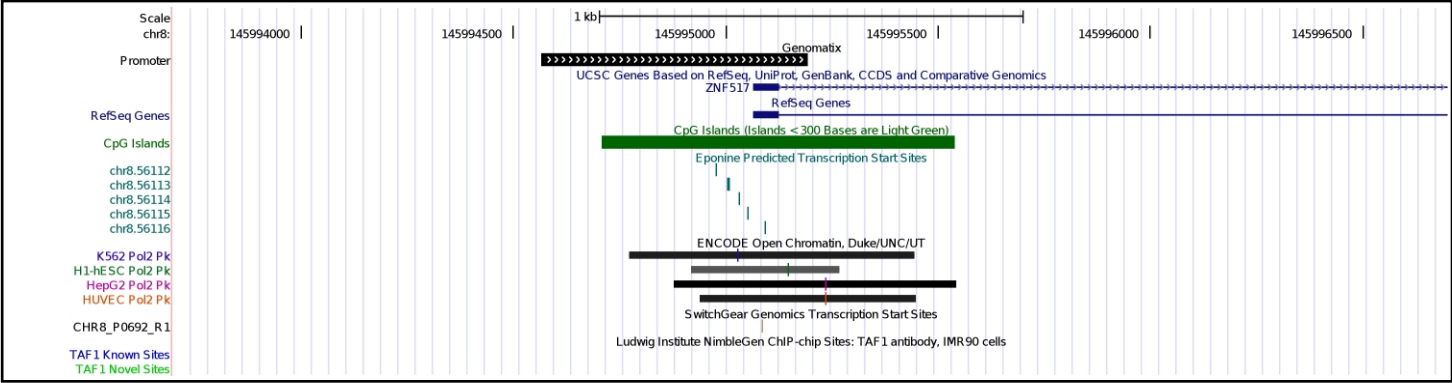

**ZNF517 (+)** **chr8:145,993,700-145,996,700**

Supplement: Additional file 12 — UCSC Genome Browser visualizations of the proximal promoter regions of the seven human 8q24.3 ZNF genes. Visualization along with associated features using the UCSC Genome Browser (human genome hg18). Genome Browser tracks included CpG islands, occupancy with RNA polymerase II core enzyme (Pol2) in four cell lines, occupancy with pre-initiation complex general transcription factor TAF1 and third party TSS predictions. Arrowheads in the depiction of the promoter regions indicate the direction of transcription. [file 1471-2164-11-206-S12.PDF]

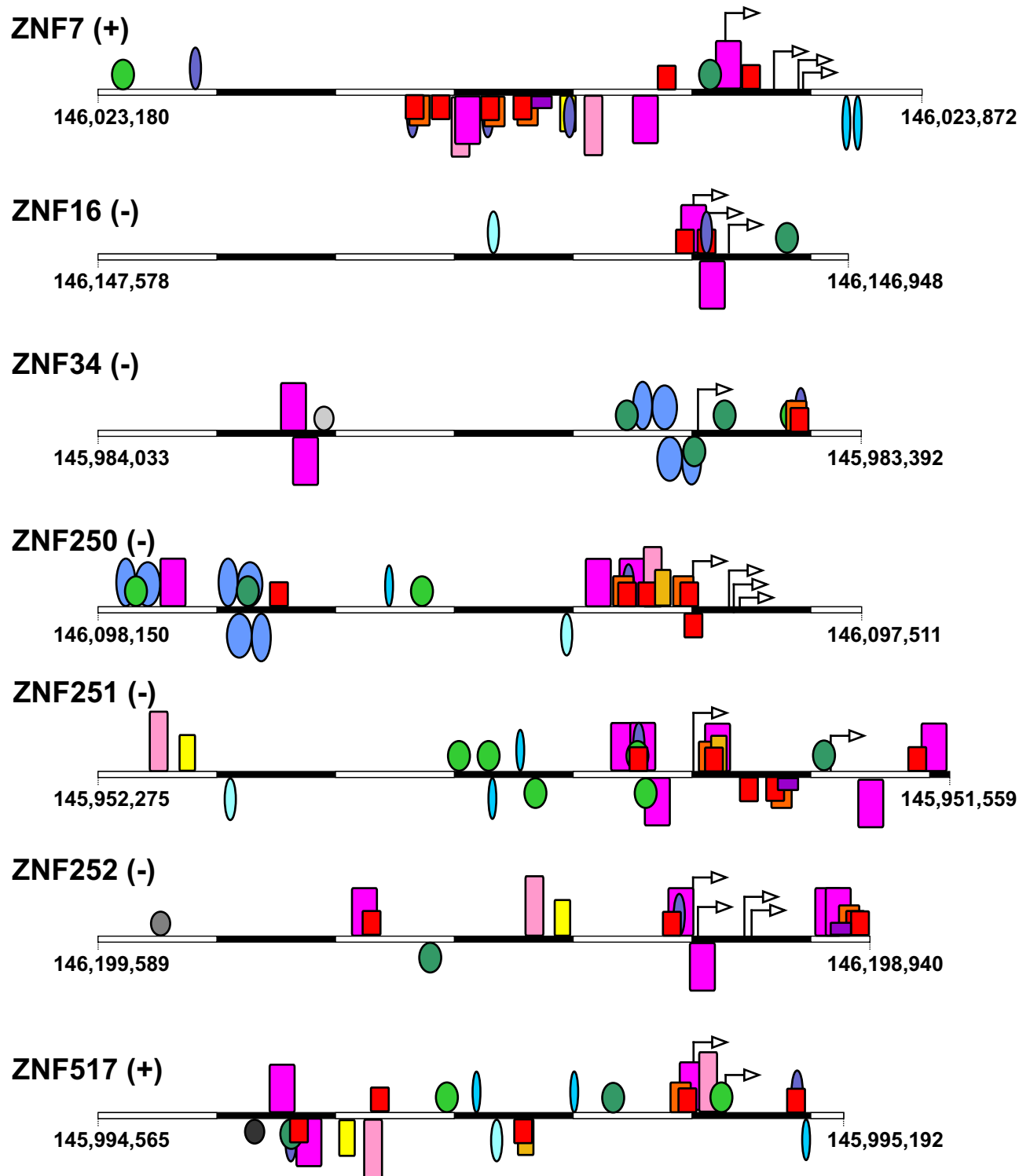

**Legend:**

**Symbols representing TFBS:**

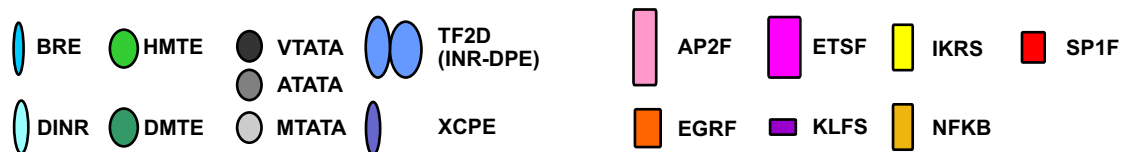

## Core promoter elements

## TFBS families

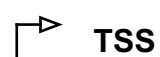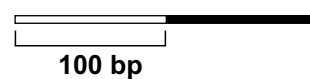

Supplement: Additional file 15 — Graphical view of TFBS and TSS within the proximal promoter regions of the seven human 8q24.3 ZNF genes. Visualization of core promoter elements and those TFBS that are derived from module families that occur in at least three promoters. The symbols representing core promoter elements have ovoid shapes, those depicting TFBS from modules are rectangles. Strand orientation is reflected in the placement of the symbols above ("sense" strand) or below ("antisense" strand) the promoter DNA depiction. The width of the symbols reflects the length of the respective DNA elements. [file 1471-2164-11-206-S15.PDF]
