# Supplementary material for: The ancient mammalian KRAB zinc finger gene cluster on human chromosome 8q24.3 illustrates principles of C2H2 zinc finger evolution associated with unique expression profiles in human tissues
Source: BMC Genomics. 2010 Mar 26;11:206. doi: 10.1186/1471-2164-11-206 (PMC2865497; doi:10.1186/1471-2164-11-206)
Supplement: Additional file 13 — Core promoter elements in the proximal promoter regions of the seven human 8q24.3 genes. Detailed listing and sequences of core promoter elements based on Genomatix MatInspector software. [file 1471-2164-11-206-S13.PDF]

Additional file 13: Core promoter elements in the proximal promoter regions of the seven human 8q24.3 genes

Gene	Individual Matrix	Matrix Family	Start	End	Strand	Sequence	Core similarity	Matrix similarity	Description
ZNF7	O$HMTE.01	O$MTEN	11	31	(+)	cgAGCCaggcgtctccgcgag	0.961	0.894	Core promoter motif ten elements (human)
ZNF7	O$XCPE1.01	O$XCPE	77	87	(+)	gcGGGGgagcg	0.761	0.847	Activator-, mediator- and TBP-dependent core promoter element for RNA polymerase II transcription from TATA-less promoters
ZNF7	O$XCPE1.01	O$XCPE	260	270	(-)	ggGCGGggccg	1.000	0.877	Activator-, mediator- and TBP-dependent core promoter element for RNA polymerase II transcription from TATA-less promoters
ZNF7	O$XCPE1.01	O$XCPE	323	333	(-)	ggGCGGagccg	1.000	0.804	Activator-, mediator- and TBP-dependent core promoter element for RNA polymerase II transcription from TATA-less promoters
ZNF7	O$XCPE1.01	O$XCPE	392	402	(-)	ggGCGGgacca	1.000	1.000	Activator-, mediator- and TBP-dependent core promoter element for RNA polymerase II transcription from TATA-less promoters
ZNF7	O$DMTE.01	O$MTEN	505	525	(+)	gcctccgAGCGgcccagggcg	1.000	0.823	Core promoter motif ten elements (Drosophila)
ZNF7	O$BRE.01	O$TF2B	627	633	(-)	ccgCGCC	1.000	1.000	RNA polymerase II transcription factor II B
ZNF7	O$BRE.01	O$TF2B	636	642	(-)	ccgCGCC	1.000	1.000	RNA polymerase II transcription factor II B
ZNF7	**TSS**		528						Transcriptional start site
ZNF7	**TSS**		569						Transcriptional start site
ZNF7	**TSS**		590						Transcriptional start site
ZNF7	**TSS**		593						Transcriptional start site
ZNF16	O$DINR.01	O$INRE	328	338	(+)	ctTCAGttgcc	1.000	0.957	Core promoter initiator elements (Drosophila)
ZNF16	O$DMTE.01	O$MTEN	570	590	(+)	ctttccaAGCGcgacccgttg	1.000	0.772	Core promoter motif ten elements (Drosophila)
ZNF16	O$XCPE1.01	O$XCPE	507	517	(+)	ggGCGGgactt	1.000	0.900	Activator-, mediator- and TBP-dependent core promoter element for RNA polymerase II transcription from TATA-less promoters
ZNF16	**TSS**		501						Transcriptional start site
ZNF16	**TSS**		514						Transcriptional start site
ZNF16	**TSS**		531						Transcriptional start site
ZNF34	O$MTATA.01	O$VTBP	183	199	(+)	ctctgTAAAtcgaaggg	1.000	0.879	Vertebrate TATA binding protein factor (muscle)
ZNF34	O$DMTE.01	O$MTEN	435	455	(+)	ttcccagAGCGgcccggacgg	1.000	0.773	Core promoter motif ten elements (Drosophila)
ZNF34	O$INR_DPE.01	O$TF2D	450	488	(+)	ggacggcgcctcggacccagacagtcggctGACGtcact	1.000	0.692	General transcription factor IID, GTF2D
ZNF34	O$INR_DPE.01	O$TF2D	470	508	(-)	ccaatcggaaccgatccggaagtgacgtcaGCCGactgt	0.848	0.722	General transcription factor IID, GTF2D
ZNF34	O$DMTE.01	O$MTEN	492	512	(-)	cccgccaATCGgaaccgatcc	0.875	0.851	Core promoter motif ten elements (Drosophila)
ZNF34	O$DMTE.01	O$MTEN	518	538	(+)	agggccgAACGggcgaggttg	0.938	0.804	Core promoter motif ten elements (Drosophila)
ZNF34	O$HMTE.01	O$MTEN	574	594	(+)	tgAGCCcggcgggggcgggcg	0.961	0.922	Core promoter motif ten elements (human)
ZNF34	O$XCPE1.01	O$XCPE	586	596	(+)	ggGCGGgcgcg	1.000	0.855	Activator-, mediator- and TBP-dependent core promoter element for RNA polymerase II transcription from TATA-less promoters
ZNF34	**TSS**		505						Transcriptional start site
ZNF250	O$INR_DPE.01	O$TF2D	15	53	(+)	gcagggaccagccaggcggctgagggcaagGTCGttccc	0.978	0.727	General transcription factor IID, GTF2D
ZNF250	O$HMTE.01	O$MTEN	22	42	(+)	ccAGCCaggcggctgagggca	0.961	0.931	Core promoter motif ten elements (human)
ZNF250	O$INR_DPE.01	O$TF2D	101	139	(+)	cgagttccaggacggccgaagccagcgcaaGTCGaggag	0.978	0.693	General transcription factor IID, GTF2D
ZNF250	O$INR_DPE.01	O$TF2D	108	146	(-)	gcagtccctcctcgacttgcgctggcttcgGCCGtcctg	0.848	0.783	General transcription factor IID, GTF2D
ZNF250	O$DMTE.01	O$MTEN	117	137	(+)	cgaagccAGCGcaagtcgagg	1.000	0.775	Core promoter motif ten elements (Drosophila)
ZNF250	O$BRE.01	O$TF2B	241	247	(+)	ccgCGCC	1.000	1.000	RNA polymerase II transcription factor II B
ZNF250	O$HMTE.01	O$MTEN	263	283	(+)	ggAGCCggccgggcccattgc	0.961	0.933	Core promoter motif ten elements (human)
ZNF250	O$DINR.01	O$INRE	389	399	(-)	cgTCATttggc	0.969	0.940	Core promoter initiator elements (Drosophila)
ZNF250	O$XCPE1.01	O$XCPE	441	451	(+)	ggGCGGgaact	1.000	0.953	Activator-, mediator- and TBP-dependent core promoter element for RNA polymerase II transcription from TATA-less promoters
ZNF250	**TSS**		501						Transcriptional start site
ZNF250	**TSS**		531						Transcriptional start site
ZNF250	**TSS**		535						Transcriptional start site
ZNF250	**TSS**		540						Transcriptional start site
ZNF251	O$DINR.01	O$INRE	106	116	(-)	tcTCAGttctt	1.000	0.970	Core promoter initiator elements (Drosophila)
ZNF251	O$HMTE.01	O$MTEN	274	294	(+)	ggAGCCcagcggggttagggt	0.961	0.965	Core promoter motif ten elements (human)
ZNF251	O$HMTE.01	O$MTEN	319	339	(+)	cgAGCGcggcgcggcccggat	1.000	0.959	Core promoter motif ten elements (human)
ZNF251	O$BRE.01	O$TF2B	326	332	(-)	ccgCGCC	1.000	1.000	RNA polymerase II transcription factor II B
ZNF251	O$BRE.01	O$TF2B	351	357	(+)	ccgCGCC	1.000	1.000	RNA polymerase II transcription factor II B
ZNF251	O$HMTE.01	O$MTEN	356	376	(-)	gcAGCTgagcggagggtctgg	0.839	0.933	Core promoter motif ten elements (human)
ZNF251	O$HMTE.01	O$MTEN	444	464	(+)	gaATCCgggcggaaccgcggc	0.761	0.913	Core promoter motif ten elements (human)
ZNF251	O$XCPE1.01	O$XCPE	450	460	(+)	ggGCGGaaccg	1.000	0.927	Activator-, mediator- and TBP-dependent core promoter element for RNA polymerase II transcription from TATA-less promoters
ZNF251	O$HMTE.01	O$MTEN	451	471	(-)	gaAGCCagccgcggttccgcc	0.961	0.890	Core promoter motif ten elements (human)
ZNF251	O$DMTE.01	O$MTEN	601	621	(-)	agcccggAACGgaccctccca	0.938	0.788	Core promoter motif ten elements (Drosophila)
ZNF251	**TSS**		501						Transcriptional start site
ZNF251	**TSS**		617						Transcriptional start site
ZNF252	O$ATATA.01	O$VTBP	44	60	(+)	cctcattTAAGcgatgg	1.000	0.796	Vertebrate TATA binding protein factor (avian)
ZNF252	O$DMTE.01	O$MTEN	270	290	(-)	gacaccgAGCGttcctgcgga	1.000	0.820	Core promoter motif ten elements (Drosophila)
ZNF252	O$XCPE1.01	O$XCPE	484	494	(+)	ggGCGGaagtt	1.000	0.806	Activator-, mediator- and TBP-dependent core promoter element for RNA polymerase II transcription from TATA-less promoters
ZNF252	**TSS**		501						Transcriptional start site
ZNF252	**TSS**		505						Transcriptional start site
ZNF252	**TSS**		544						Transcriptional start site
ZNF252	**TSS**		550						Transcriptional start site
ZNF517	O$VTATA.01	O$VTBP	123	139	(-)	tcataTAAAtttatgac	1.000	0.925	Vertebrate TATA binding protein factor
ZNF517	O$DMTE.01	O$MTEN	153	173	(-)	agggcggAGCGggggcgtccg	1.000	0.772	Core promoter motif ten elements (Drosophila)
ZNF517	O$XCPE1.01	O$XCPE	157	167	(-)	gaGCGGgggcg	1.000	0.801	Activator-, mediator- and TBP-dependent core promoter element for RNA polymerase II transcription from TATA-less promoters
ZNF517	O$HMTE.01	O$MTEN	284	304	(+)	gaAACGgagcgccgagaacgt	0.789	0.896	Core promoter motif ten elements
ZNF517	O$BRE.01	O$TF2B	315	321	(+)	ccgCGCC	1.000	1.000	RNA polymerase II transcription factor II B
ZNF517	O$DINR.01	O$INRE	331	341	(-)	taTCAGtccca	1.000	0.942	Core promoter initiator elements (Drosophila)
ZNF517	O$BRE.01	O$TF2B	397	403	(+)	ccgCGCC	1.000	1.000	RNA polymerase II transcription factor II B
ZNF517	O$DMTE.01	O$MTEN	424	444	(+)	agacgcgAGCGccatcgaggc	1.000	0.791	Core promoter motif ten elements (Drosophila)
ZNF517	O$HMTE.01	O$MTEN	515	535	(+)	agAGCCgggcgccgtgcgctc	0.961	0.916	Core promoter motif ten elements (human)
ZNF517	O$XCPE1.01	O$XCPE	583	593	(+)	ggGCGGggact	1.000	0.830	Activator-, mediator- and TBP-dependent core promoter element for RNA polymerase II transcription from TATA-less promoters
ZNF517	O$BRE.01	O$TF2B	600	606	(-)	ccgCGCC	1.000	1.000	RNA polymerase II transcription factor II B
ZNF517	**TSS**		501						Transcriptional start site
ZNF517	**TSS**		528						Transcriptional start site

Start / End **Position within promoter region relative to first nucleotide of the sequence**
strand orientation: **(+)** **Sense strand with respect to transcript**
(−) **Antisense strand with respect to transcript**

Genomatix Matrix Family Library Version 8.1
Prediction by Genomatix MatInspector 8.0
