# Supplementary material for: The ancient mammalian KRAB zinc finger gene cluster on human chromosome 8q24.3 illustrates principles of C2H2 zinc finger evolution associated with unique expression profiles in human tissues
Source: BMC Genomics. 2010 Mar 26;11:206. doi: 10.1186/1471-2164-11-206 (PMC2865497; doi:10.1186/1471-2164-11-206)
Supplement: Additional file 14 — TFBS modules occuring in at least three of the seven promoter regions of the human 8q24.3 ZNF genes. Listing of the module families, the modules, their associated individual TFBS and the respective nucleotide sequences. [file 1471-2164-11-206-S14.PDF]

Additional file 14: TFBS modules occuring in at least three of the seven promoter regions of the human 8q24.3 ZNF genes

Gene	TFBS module	1st TFBS	Start	End	Strand	Sequence	Core similarity	Matrix similarity	2nd TFBS	Start	End	Strand	Sequence	Core similarity	Matrix similarity
ZNF7	**EGRF_SP1F_01**	V$EGRF/EGR1.02	262	278	(-)	CAGGGGCGGGGCGGGGC	1.000	0.907	V$SP1F/SP1.01	259	273	(-)	GCGGGGCGGGGCCGG	1.000	1.000
ZNF7	**ETSF_SP1F_03**	V$ETSF/PEA3.01	301	321	(-)	GAGGGAGAGGAGGGCCCAGAG	1.000	0.836	V$SP1F/SP1.03	281	295	(-)	AGTGGGCGGTGCCTG	1.000	0.932
ZNF7	**SP1F_AP2F_01**	V$SP1F/GC.01	322	336	(-)	AGGGGGCGGAGCCGC	1.000	0.965	V$AP2F/AP2.02	297	311	(-)	AGGGCCCAGAGCCCG	0.905	0.795
ZNF7	**EGRF_SP1F_01**	V$EGRF/EGR1.02	325	341	(-)	CGGGCAGGGGGCGGAGC	1.000	0.924	V$SP1F/GC.01	322	336	(-)	AGGGGGCGGAGCCGC	1.000	0.965
ZNF7	**EGRF_SP1F_01**	V$EGRF/EGR1.02	353	369	(-)	GCGCCTGGGGGCGGGGT	1.000	0.903	V$SP1F/SP1.01	350	364	(-)	TGGGGGCGGGGTACA	1.000	0.997
ZNF7	**KLFS_SP1F_01**	V$KLFS/KLF6.01	364	380	(-)	AGGCGGGGCTGGCGCCT	1.000	0.932	V$SP1F/SP1.01	350	364	(-)	TGGGGGCGGGGTACA	1.000	0.997
ZNF7	**IKRS_AP2F_01**	V$IKRS/IK2.01	390	402	(-)	GGGCGGGACCAGA	1.000	0.919	V$AP2F/AP2.02	410	424	(-)	GGGGCCCGAGGGACA	0.905	0.910
ZNF7	**SP1F_ETSF_04**	V$SP1F/GC.01	471	485	(+)	GGGAGGCGGGGCTGA	1.000	0.930	V$ETSF/CETS1P54.01	451	471	(-)	CAGGCTCCGGAGAAGGGGTGG	1.000	0.843
ZNF7	**ETSF_SP1F_03**	V$ETSF/ELK1.02	521	541	(+)	GGGCGGCCGGAAGTTTGCGGG	1.000	0.980	V$SP1F/SP1.03	542	556	(+)	GCGGGGCGGACGCGG	1.000	0.924
ZNF16	**ETSF_SP1F_05**	V$ETSF/ELK1.02	491	511	(+)	GGCGGGCCGGAATTCGGGGCG	1.000	0.961	V$SP1F/GC.01	487	501	(+)	GAGCGGCGGGCCGGA	1.000	0.835
ZNF16	**SP1F_ETSF_01**	V$SP1F/SP1.01	504	518	(+)	TCGGGGCGGGACTTC	1.000	0.976	V$ETSF/ELK1.02	491	511	(+)	GGCGGGCCGGAATTCGGGGCG	1.000	0.961
ZNF16	**ETSF_ETSF_02**	V$ETSF/ELK1.02	507	527	(-)	TGACCCCCGGAAGTCCCGCCC	1.000	0.977	V$ETSF/ELK1.02	491	511	(+)	GGCGGGCCGGAATTCGGGGCG	1.000	0.961
ZNF34	**ETSF_ETSF_06**	V$ETSF/PEA3.01	154	174	(+)	GGCCCTAAGGAAGGGTGCGGT	1.000	0.930	V$ETSF/SPI1_PU1.02	165	185	(-)	GAGAAACGGGAACCGCACCCT	1.000	0.918
ZNF34	**EGRF_SP1F_01**	V$EGRF/EGR1.02	578	594	(+)	CCCGGCGGGGGCGGGCG	1.000	0.969	V$SP1F/SP1.01	583	597	(+)	CGGGGGCGGGCGCGC	1.000	0.976
ZNF250	**ETSF_SP1F_04**	V$ETSF/SPI1_PU1.02	53	73	(+)	CAGCAGCGGGAACCGCAAGGG	1.000	0.912	V$SP1F/SP1.03	145	159	(+)	GCGGGGCCGGCAGGG	1.000	0.885
ZNF250	**ETSF_SP1F_03**	V$ETSF/PDEF.01	411	431	(+)	GCCCTCCAGGATTGGCGAACT	1.000	0.888	V$SP1F/SP1.01	438	452	(+)	TGGGGGCGGGAACTG	1.000	0.973
ZNF250	**EGRF_SP1F_01**	V$EGRF/EGR1.02	433	449	(+)	GGCTGTGGGGGCGGGAA	1.000	0.987	V$SP1F/SP1.01	438	452	(+)	TGGGGGCGGGAACTG	1.000	0.973
ZNF250	**SP1F_AP2F_01**	V$SP1F/SP1.01	438	452	(+)	TGGGGGCGGGAACTG	1.000	0.973	V$AP2F/AP2.02	460	474	(+)	GGAGCCCCCAGGGAT	0.905	0.808
ZNF250	**SP1F_ETSF_01**	V$SP1F/SP1.03	452	466	(+)	GAGGGGCAGGAGCCC	1.000	0.897	V$ETSF/SPI1_PU1.02	438	458	(+)	TGGGGGCGGGAACTGAGGGGC	1.000	0.941
ZNF250	**NFKB_SP1F_04**	V$NFKB/NFKAPPAB.01	468	480	(+)	CAGGGATTGGCGG	1.000	0.810	V$SP1F/SP1.03	494	508	(-)	AAAGGGCTGCCCCGC	1.000	0.828
ZNF250	**EGRF_SP1F_01**	V$EGRF/EGR1.02	484	500	(+)	CGCAGACGGGGCGGGGC	1.000	0.909	V$SP1F/SP1.01	489	503	(+)	ACGGGGCGGGGCAGC	1.000	1.000
ZNF251	**IKRS_AP2F_01**	V$IKRS/IK2.01	69	81	(+)	AAAAGGGATTCAC	1.000	0.928	V$AP2F/AP2.02	44	58	(+)	CTGGCCTCTGGGACT	1.000	0.894
ZNF251	**ETSF_ETSF_04**	V$ETSF/ELK1.02	460	480	(-)	CGGGAACCGGAAGCCAGCCGC	1.000	0.987	V$ETSF/SPI1_PU1.02	511	531	(+)	GGGGGCGGGGAAGCCTGCGTG	1.000	0.956
ZNF251	**SP1F_ETSF_01**	V$SP1F/SP1.03	447	461	(+)	TCCGGGCGGAACCGC	1.000	0.901	V$ETSF/ELK1.02	432	452	(+)	GCGGCCCCGGAAGAATCCGGG	1.000	0.948
ZNF251	**ETSF_ETSF_02**	V$ETSF/ELK1.02	460	480	(-)	CGGGAACCGGAAGCCAGCCGC	1.000	0.987	V$ETSF/SPI1_PU1.02	446	466	(+)	ATCCGGGCGGAACCGCGGCTG	1.000	0.946
ZNF251	**EGRF_SP1F_01**	V$EGRF/EGR1.02	505	521	(+)	CCCGCAGGGGGCGGGGA	1.000	0.919	V$SP1F/SP1.01	510	524	(+)	AGGGGGCGGGGAAGC	1.000	0.997
ZNF251	**NFKB_SP1F_04**	V$NFKB/NFKAPPAB50.01	515	527	(+)	GCGGGGAAGCCTG	1.000	0.809	V$SP1F/SP1.03	540	554	(-)	GGCGGGCCGGGCCGA	1.000	0.903
ZNF251	**EGRF_SP1F_01**	V$EGRF/EGR1.02	566	582	(-)	CGGGGAGGGGGCGGGCT	1.000	0.966	V$SP1F/SP1.01	563	577	(-)	AGGGGGCGGGCTAGG	1.000	0.977
ZNF251	**KLFS_SP1F_01**	V$KLFS/KKLF.01	572	588	(-)	CAGAGCCGGGGAGGGGG	1.000	0.950	V$SP1F/SP1.01	563	577	(-)	AGGGGGCGGGCTAGG	1.000	0.977
ZNF251	**ETSF_ETSF_04**	V$ETSF/ETS1.01	640	660	(-)	GCCACCGAGGAAGCGCCGAGG	1.000	0.963	V$ETSF/ELF2.01	693	713	(+)	TCGGACCAGGAAGGAGCGCCA	1.000	0.941
ZNF251	**ETSF_SP1F_05**	V$ETSF/ELF2.01	693	713	(+)	TCGGACCAGGAAGGAGCGCCA	1.000	0.941	V$SP1F/SP1.03	682	696	(+)	GTGGGGCAGTGTCGG	1.000	0.872
ZNF252	**SP1F_ETSF_01**	V$SP1F/SP1.03	223	237	(+)	GAAGGGCCGGGTCCA	1.000	0.885	V$ETSF/SPI1_PU1.02	214	234	(+)	CTAGGTGAGGAAGGGCCGGGT	1.000	0.958
ZNF252	**IKRS_AP2F_01**	V$IKRS/IK2.01	384	396	(+)	AACCGGGAAGGAG	1.000	0.947	V$AP2F/AP2.02	360	374	(+)	TCCGCCAAGGGCTGG	0.823	0.866
ZNF252	**ETSF_ETSF_02**	V$ETSF/SPI1_PU1.02	480	500	(+)	GGCAGGGCGGAAGTTCAGATT	1.000	0.991	V$ETSF/ELK1.02	498	518	(-)	CCTGGGCCGGAAATGCCTAAT	1.000	0.984
ZNF252	**ETSF_SP1F_05**	V$ETSF/SPI1_PU1.02	480	500	(+)	GGCAGGGCGGAAGTTCAGATT	1.000	0.991	V$SP1F/SP1.03	476	490	(+)	GAAGGGCAGGGCGGA	1.000	0.896
ZNF252	**ETSF_SP1F_03**	V$ETSF/GABPB1.01	604	624	(+)	CCTGGTGCGGAGGTGCCGGAG	1.000	0.812	V$SP1F/SP1.01	630	644	(+)	CTGGGGCGGGCAGGG	1.000	0.955
ZNF252	**ETSF_SP1F_03**	V$ETSF/CETS1P54.01	613	633	(+)	GAGGTGCCGGAGTGGCGCTGG	1.000	0.817	V$SP1F/SP1.03	634	648	(+)	GGCGGGCAGGGGCGG	1.000	0.900
ZNF252	**KLFS_SP1F_01**	V$KLFS/GKLF.03	617	633	(+)	TGCCGGAGTGGCGCTGG	1.000	0.892	V$SP1F/SP1.01	630	644	(+)	CTGGGGCGGGCAGGG	1.000	0.955
ZNF252	**EGRF_SP1F_01**	V$EGRF/EGR1.02	625	641	(+)	TGGCGCTGGGGCGGGCA	1.000	0.879	V$SP1F/SP1.01	630	644	(+)	CTGGGGCGGGCAGGG	1.000	0.955
ZNF517	**ETSF_SP1F_04**	V$ETSF/CETS1P54.01	146	166	(+)	CCCTGGCCGGACGCCCCCGCT	1.000	0.924	V$SP1F/SP1.03	230	244	(+)	CGGGGGCAGAGAGTG	1.000	0.883
ZNF517	**SP1F_ETSF_01**	V$SP1F/SP1.02	161	175	(-)	GAAGGGCGGAGCGGG	1.000	0.938	V$ETSF/ETS1.01	164	184	(-)	GACCCGCAGGAAGGGCGGAGC	1.000	0.967
ZNF517	**IKRS_AP2F_01**	V$IKRS/IK2.01	203	215	(-)	GTTGGGGAGCACG	1.000	0.906	V$AP2F/AP2.02	225	239	(-)	TCTGCCCCCGGGTGG	0.905	0.894
ZNF517	**NFKB_SP1F_02**	V$NFKB/CREL.01	354	366	(-)	CTGGGGGGTTCCG	1.000	0.942	V$SP1F/TIEG.01	352	366	(-)	CTGGGGGGTTCCGGG	1.000	0.728
ZNF517	**EGRF_SP1F_01**	V$EGRF/EGR1.02	483	499	(+)	GTTTCCGGGGGCGGGGA	1.000	0.886	V$SP1F/SP1.01	488	502	(+)	CGGGGGCGGGGAAGG	1.000	0.997
ZNF517	**SP1F_AP2F_01**	V$SP1F/SP1.01	488	502	(+)	CGGGGGCGGGGAAGG	1.000	0.997	V$AP2F/AP2.02	507	521	(+)	GGAGCCTGAGAGCCG	1.000	0.793
ZNF517	**ETSF_SP1F_04**	V$ETSF/SPI1_PU1.02	489	509	(+)	GGGGGCGGGGAAGGAACCGGA	1.000	0.956	V$SP1F/SP1.01	580	594	(+)	GCGGGGCGGGGACTG	1.000	0.997

Individual colors denote different module families

Start / End **Position within promoter region relative to first nucleotide of the sequence**

strand orientation:

(+) **Sense strand with respect to transcript**

(−) **Antisense strand with respect to transcript**

Genomatix Matrix Family Library Version 8.1

Genomatix Module Library Version 5.2, Vertebrate Modules (June 2009)

Prediction by Genomatix ModelInspector 5.6.5
